# Noninnocent Deep Eutectic Solvents Enable Selective, Sustainable Nickel‐Catalyzed Cross‐Electrophile Couplings to Diarylmethanes

**DOI:** 10.1002/cssc.70970

**Published:** 2026-08-20

**Authors:** Andrea Nicola Paparella, Maristella Simone, Alessia Battezzato, Laura Chevet, Laetitia Chausset‐Boissarie, Muriel Durandetti, Filippo Maria Perna, Vito Capriati

**Affiliations:** ^1^ Dipartimento di Farmacia–Scienze del Farmaco Università degli Studi di Bari Aldo Moro Consorzio CINMPIS Bari Italy; ^2^ Université de Rouen Normandie INSA Rouen Normandie Université de Caen Normandie ENSICAEN CNRS Institut CARMeN UMR 6064 Rouen France

**Keywords:** cross‐electrophile coupling, deep eutectic solvents, diarylmethanes, nickel catalysis, sustainable chemistry

## Abstract

We report the first sustainable nickel‐catalyzed cross‐electrophile coupling (XEC) between aryl iodides and benzyl chlorides conducted in a deep eutectic solvent (DES), choline chloride/urea (1:2 mol mol^–1^), replacing the hazardous amide solvents traditionally used in this transformation. The reaction proceeds efficiently under mild (50 °C), aerobic, and heterogeneous conditions, using zinc as the terminal reductant, (dtbbpy)NiBr_2_ (2 mol%) as catalyst, and MePh_3_PBr (10 mol%) as additive, delivering a broad range of diarylmethanes in up to 95% yield with wide functional‐group tolerance and gram‐scale applicability within 3 h. Mechanistic and electrochemical studies support a radical/polar crossover pathway in which benzyl radicals, generated off‐cycle from benzyl chlorides, are selectively funneled into productive cross‐coupling through a predominantly Ni(I)/Ni(II)/Ni(III) catalytic manifold rather than diverted into homodimerization. The phosphonium salt appears to modulate nickel speciation, stabilize reduced Ni intermediates, and promote interfacial electron transfer between insoluble substrates and the fraction of nickel catalyst dissolved in the DES. The halide‐rich DES environment likely enhances the reducing power of Zn through Zn^2+^ chloride complexation and improved zinc surface activation. Overall, this study establishes DESs as noninnocent media for XEC, providing a sustainable platform beyond conventional volatile organic compound (VOC)‐based solvent systems.

## Graphical abstract

Ni‐catalyzed cross‐electrophile coupling (XEC) of aryl iodides with benzyl chlorides proceeds efficiently in the deep eutectic solvent (DES) choline chloride/urea, replacing hazardous amide solvents. Under mild, aerobic, heterogeneous conditions, diarylmethanes form in up to 95% yield. Mechanistic studies reveal solvent‐enabled control of radical pathways and Zn‐mediated redox processes, positioning DESs as noninnocent media for sustainable XEC.

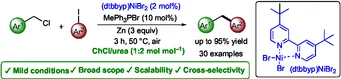

## 1 Introduction

Diarylmethanes constitute a privileged structural motif featured across a broad spectrum of pharmaceuticals, agrochemicals, and functional material [1, 2, 3]. Beyond their well‐established biological relevance, these scaffolds also serve as versatile molecular building blocks, with widespread applications as ligands, organocatalysts, and key intermediates in modern synthetic chemistry [4]. Structurally, diarylmethanes are characterized by a central methylene bridge flanked by two aryl rings—a configuration that offers considerable flexibility for downstream functionalization. The benzylic position is particularly reactive, enabling a broad range of transformations that make diarylmethanes highly valuable intermediates for accessing structurally complex and biologically relevant compounds [4, 5].

**SCHEME 1:**
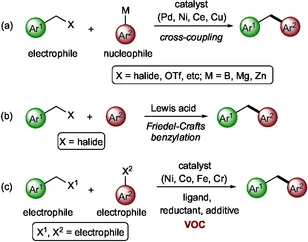
Representative strategies for the synthesis of diarylmethanes: (a) metal‐catalyzed cross‐coupling between nucleophilic and electrophilic partners; (b) Lewis acid‐catalyzed Friedel–Crafts benzylation; (c) XEC in VOCs.

Traditional synthetic approaches to diarylmethanes typically involve metal‐catalyzed cross‐couplings between nucleophilic and electrophilic partners (Scheme 1a) [6, 7, 8, 9, 10, 11, 12, 13, 14, 15, 16, 17]. A metal‐free carbon–carbon coupling between tosylhydrazones and boronic acids has also been reported, offering a general and functional‐group‐tolerant route to 1,1‐diarylalkanes [18]. Friedel–Crafts benzylation of arenes remains a classical route to 1,1‐diarylalkanes (Scheme 1b) [19, 20], with notable recent advances including catalyst‐ and additive‐free protocols—albeit typically requiring harsh conditions (e.g., 140 °C) [21], or the use of inexpensive iron‐based catalysts in volatile organic compounds (VOCs) such as CH_2_Cl_2_, CH_3_NO_2_ [22, 23]. More sustainable protocols have emerged employing FeCl_3_‐based deep eutectic solvents (DESs) as green reaction media, enabling benzylation with common electrophilic partners such as styrenes, alcohols, acetates, ethers, and chlorides under milder and more environmentally friendly conditions [24, 25]. Oxidative coupling reactions, including electrochemically and photochemically driven processes [26, 27, 28, 29, 30, 31], as well as the activation and cleavage of benzyl fluorides by electrophilic phosphonium cations [32], and Zn‐mediated Pd‐catalyzed on‐water cross‐coupling reactions between benzylic and (hetero)aryl halides [33, 34] have also been documented.

Cross‐electrophile coupling (XEC) has emerged as a powerful strategy for constructing diarylmethanes (Scheme 1c), offering distinct advantages over traditional approaches by avoiding the use of preformed organometallic nucleophiles—often limiting in terms of scope, availability, and substrate compatibility. XEC involves the selective coupling of two distinct *σ*‐electrophiles under reductive catalytic conditions, typically requiring a transition metal catalyst and a stoichiometric reductant to enable the turnover cycle. Achieving high cross‐selectivity over competing homocoupling pathways remains a central challenge and depends critically on the interplay between catalyst (typically a first‐row transition metal), ligand, additive, solvent, and reductant [35, 36, 37, 38, 39, 40]. Significant advances have been reported in the synthesis of diarylmethanes via C(sp^2^)–C(sp^3^) XEC. Representative examples include (a) Ni‐catalyzed cross‐coupling of aryl iodides with benzylpyridinium salts using homogeneous organic reductants [41]; (b) Ni‐catalyzed dynamic kinetic cross‐couplings of benzyl alcohols with (hetero)aryl electrophiles [42]; (c) Ni‐ and Co‐catalyzed systems employing benzyl alcohols via in situ mesylation [43]; (d) Co‐catalyzed reactions of aryl halides with benzylic electrophiles [44, 45]; (e) Ti‐assisted Ni catalysis for aryl halides/benzyl alcohol coupling [46]; (f) Ni‐catalyzed reactions of benzylic esters with aryl halides [47]; and (g) Ni‐catalyzed XEC of aryl bromides with benzyl chlorides, using tailored additive systems [48].

Despite these advances, most XEC protocols still rely heavily on toxic and hazardous VOCs such as amides, ethers, and nitriles, with *N*,*N*‐dimethylacetamide (DMA) and *N*,*N*‐dimethylformamide (DMF) accounting for nearly 50% of all solvents used in reported XEC reactions [40]. While these solvents have proven highly effective, their increasing restriction in industrial practice due to environmental and health concerns has stimulated the search for more sustainable alternatives [49]. In the context of the 12 Principle of Green Chemistry proposed by Anastas and Warner [50, 51], the development of XEC methodologies in benign and nonvolatile media represents an important, yet still underexplored direction [52, 53].

**SCHEME 2:**
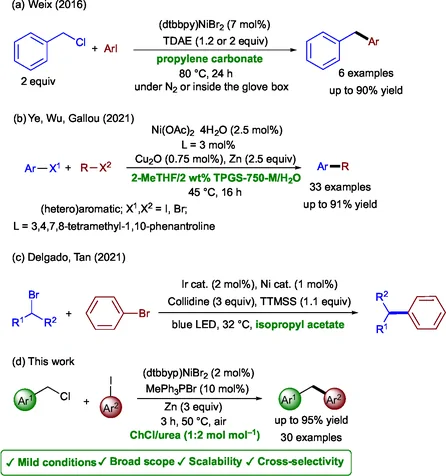
Sustainable XEC methodologies: (a) Ni‐catalyzed XEC in propylene carbonate; (b) Ni‐catalyzed XEC under micellar conditions in a 2‐MeTHF/aqueous medium; (c) Ir/Ni‐catalyzed photoredox XEC in isopropyl acetate; (d) Ni‐catalyzed XEC in DESs. (dtbbpy)NiBr_2_ = [4,4′‐bis(*tert*‐butyl)‐2,2′‐bipyridine]nickel dibromide. TDAE = tetrakis(dimethylamino)ethylene. TPGS‐750‐M = DL, *α*‐tocopherol methoxypolyethylene glycol succinate solution. TTMSS = tris(trimethylsilyl)silane. ChCl = choline chloride.

To date, only limited examples of XEC reactions in nonamide solvents have been reported. For instance, Weix et al. described in 2016 one of the first examples of XEC between benzyl chloride and aryl iodides in propylene carbonate, employing tetrakis(dimethylamino)ethylene (TDAE) as an organic reductant under homogeneous conditions (Scheme 2a). The reactions were conducted under a nitrogen atmosphere or in a glove box [54]. More recently, alternative approaches have been developed using aqueous micellar systems under Ni/Cu catalysis (Scheme 2b) [55] or lipophilic dual Ir/Ni catalysis in ester solvents such as isopropyl acetate (Scheme 2c) [56]. These studies highlight the feasibility of performing XEC under nonconventional conditions, although the scope of such methodologies remains relatively limited.

DESs are gaining significant momentum as promising sustainable alternatives to conventional VOCs in both catalytic and stoichiometric transformations. This growing interest is driven by their unique combination of favorable features: these media are typically composed of inexpensive, bio‐based components and are characterized by low toxicity, nonflammability, negligible vapor pressure, and tunable physicochemical properties [57, 58]. In addition to their environmental advantages, DESs can act as structured and highly interactive media, capable of influencing reactivity and selectivity. As part of our ongoing commitment to advancing safer and more sustainable chemical processes, our group has embarked on a program aimed at harnessing nonconventional solvents—such as DESs and water as noninnocent reaction media—to enable sustainable cross‐coupling reactions for the formation of carbon–carbon and carbon‐heteroatom bonds. This strategy also extends to key transformations aimed at synthesizing cornerstone functional groups, including amines, amides, ethers, and *α*‐ and *β*‐hydroxy phosphine oxides [59, 60, 61, 62, 63, 64, 65, 66, 67, 68]. Beyond polar and ionic processes, DESs have also recently demonstrated their ability to support radical chemistry. For example, isolated photosynthetic reaction centers have been shown to operate in DES mixtures, mediating electron and proton transfer in nonaqueous environments [69], while photocatalytic C—C bond formation via radical pathways has also been achieved [70].

Herein, we report the first example of a Ni‐catalyzed XEC performed in a DES composed of choline chloride (ChCl)/urea (1:2 mol mol^–1^), enabling the synthesis of diarylmethanes from aryl iodides and benzyl chlorides. The reaction proceeds under aerobic and heterogeneous conditions in the presence of Zn powder as a terminal reductant and methyltriphenylphosphonium bromide (MePh_3_PBr) as an additive. Key advantages of this protocol include: (a) mild and operationally simple conditions (50 °C, tolerance to air, and reaction times typically within 3 h), while avoiding the use of amide solvents, an inert atmosphere, or specialized equipments; (b) broad substrate scope, affording diarylmethanes in up to 95% isolated yield (30 examples); and (c) excellent scalability and chemoselectivity (Scheme 2d). These results demonstrate the feasibility of conducting XEC reactions in DESs and highlight their potential as sustainable, noninnocent media for reductive cross‐coupling chemistry.

## 2 Results and Discussion

### 2.1 Optimization Studies

Given that XEC catalysis typically proceeds via single‐electron redox pathways, inexpensive and readily available 3d transition metals are generally preferred. Among these, nickel has emerged as the catalyst of choice in the majority of reported systems [40]. As a model reaction, we selected the coupling between 1‐ethyl‐2‐iodobenzene (**1a**) and 4‐chlorobenzyl chloride (**2a**) to access 1‐(4‐chlorobenzyl)‐2‐ethylbenzene (**3a**). Our initial investigations employed (2,2′‐bipyridine)nickel(II) dibromide [(byp)NiBr_2_] (2 mol%) as the catalyst and elemental Zn(0) powder as the reductant (2 equiv). The catalyst was first dissolved in water, giving a green solution, which was then warmed to 50 °C. Upon the addition of zinc powder, the reaction mixture turned gray, and was stirred for 10 min before adding **2a** in a one‐shot fashion. However, mainly unreacted starting materials were recovered after 16 h, even in the presence of *N*,*N*,*N′*,*N′*‐tetramethylethylenediamine (TMEDA) (2 equiv) or various amphiphilic additives such as tetrabutylammonium bromide (TBAB, 5 mol%) and tetraoctylammonium bromide (TOAB, 5 mol%) (Table S1, entries 1–4). Upon switching the solvent to eutectic mixtures such as ChCl/ethylene glycol (EG) and ChCl/urea (1:2 mol mol^–1^), the desired cross‐coupled product **3a** was obtained in up to 17% yield in ChCl/EG. In contrast, the major byproduct was the homodimer of **2a**, namely 1,2‐bis(4‐chlorophenyl)ethane (**4a**), which formed in up to 80% yield in ChCl/urea (Table 1, entries 1,2). Notably, the amount of **4a** could be reduced to 40% by increasing the Zn loading to 3 equiv (Table 1, entry 3). Subsequent optimization studies were carried out in ChCl/urea, as the formation of additional side products—including Ullmann‐type C–O coupling derivatives, identified by ^1^H NMR and GC–MS analysis—was observed in ChCl/EG. These byproducts, likely arising from interactions between the substrates and the DES components, were highly soluble in ChCl/EG, thereby making both their extraction and accurate quantification challenging.

**TABLE 1:** Optimization of the reaction conditions.^a^

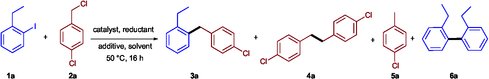								
Entry	Solvent	Catalyst, mol%	Reductant (equiv)	Additive, mol%	**3a** yield, %^b^	**4a** yield, %^b^	**5a** yield, %^b^	**6a** yield, %^b^
1	ChCl/EG^c^	(byp)NiBr_2_ (2)	Zn (2)	—	17	21	—	—
2	ChCl/urea	(byp)NiBr_2_ (2)	Zn (2)	—	10	80	—	—
3	ChCl/urea	(byp)NiBr_2_ (2)	Zn (3)	—	15	40	—	—
4	ChCl/urea^d^	NiBr_2_ (5)	Zn (3)	—	—	40	40	—
5	ChCl/urea^d^	Ni(OAc)_2_ (5)	Zn (3)	—	—	20	40	—
6	ChCl/urea^d^	(phe)NiBr_2_ (2)	Zn (3)	—	37	32	18	—
7	ChCl/urea^d^	(dtbbpy)NiBr_2_ (2)	Zn (3)	—	40	40	—	—
8	ChCl/urea^d^	(dtbbpy)NiBr_2_ (2)	Mn (3)	—	30	60	—	—
9	ChCl/urea^d^	(dtbbpy)NiBr_2_ (2)	Mg (3)	—	—	95	—	—
10	ChCl/urea^d^	(dtbbpy)NiBr_2_ (2)	Zn (3)^e^	—	—	48	—	—
11	ChCl/urea^d^, ^f^	(dtbbpy)NiBr_2_ (2)	Zn (3)	—	4	16	<5	24
12	ChCl/urea^d^	(dtbbpy)NiBr_2_ (2)	Zn (3)	(*n*‐Bu)_3_P (10)	<5	80	8	—
13	ChCl/urea^d^	(dtbbpy)NiBr_2_ (2)	Zn (3)	MePh_2_P (10)	8	58	—	—
14	ChCl/urea^d^	(dtbbpy)NiBr_2_ (2)	Zn (3)	(2‐furyl)_3_P (10)	11	80	10	—
15	ChCl/urea^d^	(dtbbpy)NiBr_2_ (2)	Zn (3)	Cy_3_P (10)	20	74	12	—
16	ChCl/urea^d^	(dtbbpy)NiBr_2_ (2)	Zn (3)	Ph_3_P (10)	40	52	—	10
17	ChCl/urea^d^	(dtbbpy)NiBr_2_ (2)	Zn (3)	TBAB (10)	—	—	—	—
18	ChCl/urea^d^	(dtbbpy)NiBr_2_ (2)	Zn (3)	LiBr (10)	7	79	10	10
19	ChCl/urea^d^	(dtbbpy)NiBr_2_ (2)	Zn (3)	MePh_3_PBr (10)	58	28	—	—
20	ChCl/urea^d^, ^g^	(dtbbpy)NiBr_2_ (2)	Zn (3)	MePh_3_PBr (10)	80^h^	20	—	—
21	ChCl/urea^d^, ^g^	NiBr_2_·3H_2_O (2) + dtbbpy (4)	Zn (3)	MePh_3_PBr (10)	80^h^	20	—	—
22	ChCl/urea^d^, ^g^, ^i^, ^j^	(dtbbpy)NiBr_2_ (2)	Zn (3)	MePh_3_PBr (10)	80^h^	20	—	—
23	ChCl/acetamide^d^, ^g^, ^i^	(dtbbpy)NiBr_2_ (2)	Zn (3)	MePh_3_PBr (10)	58	42	—	—
24	ChCl/urea^d^, ^g^, ^i^	—	Zn (3)	MePh_3_PBr (10)	—	24	<5	—
25	ChCl/urea^d^, ^g^, ^i^	(dtbbpy)NiBr_2_ (2)	—	MePh_3_PBr (10)	—	—	—	—
26	CH_3_CN^d^, ^g^, ^i^	(dtbbpy)NiBr_2_ (2)	Zn (3)	MePh_3_PBr (10)	15	40	15	—
27	Toluene^d^, ^g^, ^i^	(dtbbpy)NiBr_2_ (2)	Zn (3)	MePh_3_PBr (10)	30	25	—	—
28	THF^d^, ^g^, ^i^, ^k^	(dtbbpy)NiBr_2_ (2)	Zn (3)	MePh_3_PBr (10)	52	41	—	—
29	DMF^d^, ^g^, ^i^, ^k^	(dtbbpy)NiBr_2_ (2)	Zn (3)	MePh_3_PBr (10)	80	20	—	—

a Reaction conditions: 0.7 mL solvent per 0.5 mmol of **1a** and 0.75 mmol of **2a**; one‐shot addition of **2a** to the reaction mixture. Choline chloride (ChCl)/urea (1:2 mol mol^–1^); ChCl/ethylene glycol (EG) (1:2 mol mol^–1^); ChCl/acetamide (1:2 mol mol^–1^); (byp)NiBr_2_: (2,2′‐bipyridine)nickel(II) dibromide; (phe)NiBr_2_: 1,10‐phenantroline nickel(II) dibromide; (dtbbpy)NiBr_2_: [4,4′‐bis(*tert*‐butyl)‐2,2′‐bipyridine]nickel dibromide; Cy_3_P: tricyclohexylphosphine; TBAB: tetrabutylammonium bromide. All reactions were carried out under a confined headspace; this condition was found to be important for achieving optimal reactivity and selectivity in the optimized systems.

b Calculated by ^1^H NMR analysis of the crude reaction mixture using an internal standard technique (NMR internal standard: CH_2_Br_2_).

c Additional byproducts, likely resulting from interaction between the substrates and the DES components, were formed and found to be highly soluble in the eutectic mixture.

d Dropwise addition of **2a** to the reaction mixture.

e Zn granular.

f Temperature: 80 °C.

g DES or VOC: 0.5 mL.

h Isolated yield.

i Reaction time: 3 h.

j Product **3a** was also obtained in 70% yield when the reaction was carried out on a 10 mmol scale using **1a** (10 mmol) and **2a** (15 mmol) in 10 g of ChCl/urea.

k Zn powder was preactivated with trifluoroacetic acid (30 μL).

To minimize byproduct formation, **2a** (1.5 equiv) was added dropwise over 1 h in all subsequent screenings. Catalyst evaluation revealed that simple salts such as NiBr_2_ (5 mol%) and Ni(OAc)_2_ (5 mol%) suppressed the formation of **3a** and instead promoted the formation of **4a** and the hydrodehalogenated derivative of **2a**, that is 4‐chlorotoluene (**5a**), in yields of up to 40% (Table 1 entries 4,5). By contrast, using nickel complexes bearing chelating ligands—such as 1,10‐phenantroline nickel(II) dibromide [(Phe)NiBr_2_] (2 mol%) and [4,4′‐bis(*tert*‐butyl)‐2,2′‐bipyridine]nickel dibromide [(dtbbpy)NiBr_2_] (2 mol%)—afforded **3a** in up to 40% yield, although with notable amounts of **4a** and **5a** (Table 1, entries 6,7). Other Ni‐based catalysts—including nickel(II) gem‐dimethyl‐bis(oxazoline) diacetate [NIBOX(OAc)_2_] (2 mol%), nickel(II) *N*,*N*′‐bis(salicylidene)ethylenediamine [Nickel(II) salen] (2 mol%), dichlorobis[1,3‐bis(diphenylphoshino)propane]nickel (II) [(dppp)NiCl_2_] (2 mol%) as well as in situ combinations of NiBr_2_ (2 mol%) with terpyridine (4 mol%) or [1,3‐bis(2,4,6‐trimethylphenyl)imidazole‐2‐ylidene] (IMesNHC) (4 mol%)—proved completely ineffective under the same conditions (Table S2, entries 6–10). A screening of reductants with varying reduction potentials was also carried out. The use of Mn(0), Mg(0) powders, as well as granular Zn, resulted in a substantial increase in the formation of the homocoupled byproduct **4a**, reaching yields up to 95% at the expense of the desired product **3a** (Table 1, entries 8–10). Conversely, no reaction occurred when using other inorganic reductants such as Al(0) and Fe(0) powders or organic reductants including ascorbic acid, glycerol, and triethylsilane (Table S3, entries 3,4,7–9). At room temperature (25 °C), the catalytic cycle failed to initiate. However, increasing the reaction temperature to 80 °C led to a rise in the formation of byproducts such as **4a** and 2,2′‐diethyl‐1,1′‐biphenyl (**6a**), the homocoupling product of **1a**, again at the expense of **3a** (Table 1, entry 11) (see Table S4 for additional temperature screening).

The influence of various additives on the reaction outcome was systematically investigated. These additives could, in principle, exert multiple, potentially concurrent roles, including coordination to the catalyst, modulation of its speciation, substrate activation, facilitation of the reduction step, or promotion of halide exchange on the alkyl electrophile. However, the use of phosphine ligands did not lead to any improvement in the yield of the cross‐coupled product **3a**; in several cases, a detrimental effect was observed (Table 1, entries 12–16; see Table S5 for additional ligand screening). Likewise, replacement with an ammonium salt such as TBAB resulted in complete recovery of the starting materials, while simple bromide salts (e.g., LiBr) predominantly promoted homocoupling (up to 79% of **4a**; Table 1, entries 17,18). These results indicate that neither a generic ligand nor the presence of bromide alone accounts for productive reactivity.

In contrast, the use of a phosphonium salt–specifically MePh_3_PBr–led to a marked improvement, affording **3a** in 58% yield while suppressing the formation of the homocoupling product **4a** (28%; Table 1, entry 19). To the best of our knowledge, this represents the first example of a phosphonium salt employed in XEC reactions.

Although the origin of this effect is not yet fully understood, it likely reflects a more specific role of the phosphonium salt. Given the heterogeneous nature of the system—where both substrates and the reductant are largely insoluble in the eutectic phase—a contribution related to interfacial processes may be envisaged. However, at this stage, such an effect should be regarded as a possible contributing factor rather than a demonstrated mechanism. Further insight into the role of the phosphonium additive is provided by the electrochemical studies (*vide infra*).

Further improvement was achieved by decreasing the DES volume to 0.5 mL per 0.5 mmol of substrate, which increased the yield of **3a** to 80% while reducing the formation of **4a** to 20% (Table 1, entry 20). Thus, a higher local concentration of the aryl iodide promotes cross‐selectivity over benzyl chloride dimerization. It is worth noting that decreasing the volume of the eutectic mixture leads to increased viscosity of the reaction medium. Therefore, to ensure effective mixing of the reagents, magnetic stirring should be kept at a speed of no less than 900 rpm. Notably, the yield remained unchanged when the catalyst was generated in situ by sequential addition of NiBr_2_·3H_2_O (2 mol%) and dtbbpy (4 mol%) to the DES, followed by the phosphonium salt (Table 1, entry 21). Pleasingly, the reaction time could also be shortened from 16 to just 3 h without any loss in yield (Table 1, entry 22).

Finally, the influence of solvent nature was evaluated under the optimized reaction conditions. Water and aqueous/alcohol‐based DESs—such as ChCl/H_2_O (1:2 mol mol^−1^), ChCl/glycerol (Gly) (1:2 mol mol^−1^), and ChCl/EG (1:2 mol mol^−1^)—proved sluggish (up to 10% yield of **3a**) (Table S6, entries 1–4). The use of ChCl/acetamide (1:2 mol mol^−1^) afforded **3a** in 58% yield (Table 1, entry 23), whereas no reaction occurred in ChCl/*N*,*N*′‐dimethylurea (1:2 mol mol^−1^) (**4a**: 50% yield; Table S6, entry 7). Both of these mixtures are characterized by high viscosity, and maintaining a liquid state at 50 °C proved challenging. Hydrophobic DESs such as thymol/menthol (1:1 mol mol^−1^), as well as Lewis‐acid‐based mixtures like ZnCl_2_/acetamide (1:4 mol mol^−1^) and NiCl_2_/urea (1:3.5 mol mol^−1^), also failed to promote the reaction (Table S6, entries 8–10). Control experiments revealed that, in the absence of (dtbbpy)NiBr_2_, the starting materials remained largely unreacted, consistent with the lack of direct Zn insertion into the organohalide (Table 1, entry 24). We also established that Negishi cross‐coupling reactions do not operate under these conditions. The reaction of a freshly prepared solution of PhZnI (1 mmol, 1.0 M in THF solution) with **2a** (1.5 mmol) resulted exclusively in the formation of **4a** (>95% yield), whereas the reaction of commercially available benzylzinc bromide (1.5 mmol, 1.0 M in THF solution) with iodobenzene (1 mmol) led only to the detection of 1,2‐diphenylethane (5% yield) (as determined by ^1^H NMR and GC–MS analysis) (see the Supporting Information section). Conversely, omission of zinc completely suppressed the reaction (Table 1, entry 25). When benchmarked against conventional VOCs, acetonitrile, toluene, and THF proved largely ineffective, affording **3a** in only 15%–52% yield. Among the organic solvents examined, DMF was the only medium approaching the performance of ChCl/urea, delivering **3a** in 80% yield (Table 1, entries 26–29). Notably, the yields obtained in both THF and DMF were obtained only after preactivation of Zn powder with trifluoroacetic acid (30 μL). In the absence of this treatment, no reaction occurred, and the starting materials were quantitatively recovered. However, it should be noted that the observed reactivity strongly depends on the state of the zinc reductant. When freshly prepared zinc, properly stored under an inert atmosphere (e.g., in a glovebox), is employed, no acid activation is required, and high reactivity is observed under both aerobic and inert conditions. Therefore, the lack of reactivity in THF/DMF without acid activation can be attributed to the use of passivated zinc. This interpretation is consistent with literature reports [36, 71]. The use of this acid was unnecessary in the case of ChCl/urea, which efficiently removes the passivation layer of zinc, likely through solubilization of zinc oxide [72, 73, 74, 75]. Reactions of **1a** with benzyl bromide, benzyl mesylate, or benzyl azide as well as reactions of 1‐ethyl‐2‐bromo benzene or 1‐ethyl‐2‐chlorobenzene with **2a**, afforded **3a** in yields not exceeding 25%. In all cases, the formation of the dimeric product **4a** was significantly favored, reaching levels above 95% (Table S7). The robustness of the protocol was further underscored by its successful scale‐up to the gram level. The reaction between **1a** (10 mmol, 2.3 g) and **2a** (15 mmol, 2.4 g) proceeded smoothly under the optimized condition—3 h at 50 °C in a ChCl/urea eutectic mixture (10 mL)—affording the desired product **3a** in an excellent isolated yield of 70% (1.60 g) (Table 1, entry 22). These findings further reinforce the potential of the method for real‐world applications.

**FIGURE 1:**
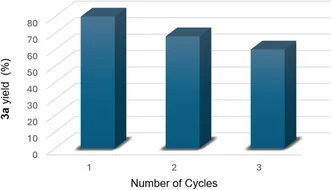
Reuse of (dtbbpy)NiBr_2_, MePh_3_PBr, and ChCl/urea in the synthesis of **3a** from **1a** and **2a**.

Reuse of the DES medium, the Ni catalyst, and the phosphonium additive was subsequently investigated in the model XEC reaction, leading to **3a**. The reaction was conducted under the optimized conditions (Table 1, entry 22) using **1a** (1 mmol), **2a** (1.5 mmol), (dtbbpy)NiBr_2_ (2 mol%), MePh_3_PBr (10 mol%), and Zn powder (3 equiv) in ChCl/urea (1 mL) at 50 °C for 3 h. Upon completion (TLC monitoring), product **3a** was isolated by extraction with EtOAc (1 mL) in 80% yield. The recovered reaction mixture was then reused by charging fresh electrophiles (**1a**, 1 mmol; **2a**, 1.5 mmol) together with a reduced amount of Zn (1.5 equiv). This adjustment was made to avoid further accumulation of residual zinc from the previous cycle and to limit the progressive increase in medium viscosity. Under otherwise identical conditions, the second cycle afforded **3a** in 68% yield, with 15% of unreacted **1a**. A third cycle was carried out following the same protocol. Owing to the further increase in viscosity, 400 μL of fresh DES were added to ensure efficient stirring and mass transfer. Under these conditions, product **3a** was obtained in 60% yield, along with 37% of unreacted starting material (Figure 1). Although these experiments clearly demonstrate the feasibility of reusing the DES medium together with the nickel catalyst and the phosphonium additive over consecutive cycles, a gradual erosion of the reaction efficiency was observed. This detrimental effect likely correlates with the progressive accumulation of zinc metal and zinc‐derived salts, which leads to a marked increase in medium viscosity and, consequently, to impaired mass transfer. Notably, partial dilution of the reaction mixture with fresh DES in the third cycle proved insufficient to restore the initial reactivity. Moreover, this intervention inevitably perturbed the optimized stoichiometry and concentration balance of the system, thereby preventing recovery of the yield obtained in the first run.

### 2.2 Reaction Scope

With the optimal conditions established, we next explored the substrate scope by coupling a variety of benzyl chlorides with structurally diverse aryl iodides. We first examined the reactivity of unsubstituted benzyl chloride (**2b**) with a selection of aryl iodides featuring varied electronic and steric properties. Both 1‐iodonaphtalene (**1b**) and 4‐iodo‐1,1′‐biphenyl (**1c**) participated efficiently in the transformation, affording the corresponding diarylmethanes **3b** and **3c** in excellent yields (74%–88%, Table 2). Electron‐deficient aryl iodides were also readily accommodated: substrates bearing a ketone (4′‐iodoacetophenone, **1d**), a potentially coordinating nitrile (4‐iodobenzonitrile, **1e**), an ester functionality (ethyl 4‐iodobenzoate, **1f**), as well as a stereodefined conjugated system [ethyl (*E*)‐3‐(4‐iodophenyl)acrylate], **1g**), all underwent smooth coupling with **2b**, affording products **3d**–**g** in moderate to excellent yields (55%–95%). Notably, an o*rtho*‐substituted, electron‐rich aryl iodide such as 2‐iodoanisole (**1h**) was well tolerated, providing adduct **3h** in 80% yield (Table 2). In contrast, 1‐iodo‐4‐nitrobenzene reacted with **2a** to give a complex mixture, likely due to competitive nitro‐group reduction, while 4‐iodobenzoic acid proved unreactive, presumably as a result of catalyst deactivation or unfavorable acid–base interactions (see the Supporting Information section for additional unreacted substrates).

**TABLE 2:** Ni‐catalyzed XECs between aryl iodides **1** and benzyl chlorides **2** to give diarylmethanes **3**.^a^

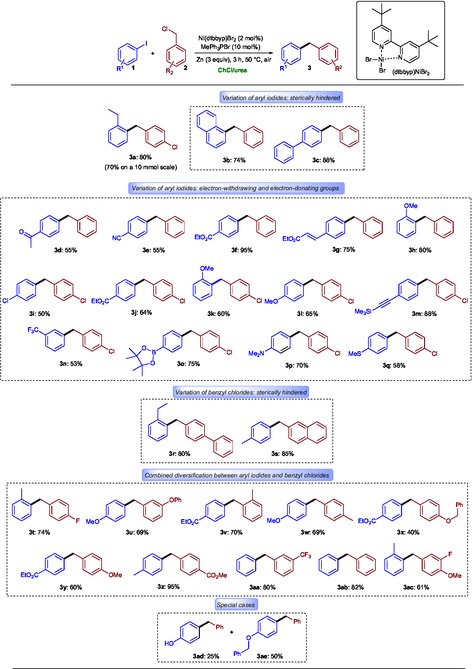	

a Reaction conditions: 0.5 mL DES per 0.5 mmol of **1** and 0.75 mmol of **2**; dropwise addition of **2** to the reaction mixture. ChCl: choline chloride. Isolated yields.

We then turned to benzyl chlorides featuring steric and electronic diversity. For instance, *p*‐chlorobenzyl chloride (**2a**) coupled efficiently with 1‐chloro‐4‐iodobenzene (**1i**), delivering dichlorinated diarylmethane **3i** in 50% yield with complete chemoselectivity. A broad array of aryl iodides was subsequently evaluated in combination with **2a**, including substrates bearing ester (**1f**), methoxy (**1h** and 4‐iodoanisole, **1j**), alkynylsilane [(4‐iodophenylethynyl)trimethylsilane, **1k**), trifluoromethyl [1‐iodo‐3‐(trifluoromethyl)benzene, **1l**], boronic ester [4‐iodophenylboronic acid pinacol ester, **1m**], dimethylamino (4‐iodo‐*N*,*N*‐dimethylaniline, **1n**), and thioether [4‐iodophenyl)(methyl)sulfane, **1o**] substituents. All these coupling partners were successfully engaged, furnishing diarylmethanes **3j**–**q** in 53%–88% yield (Table 2). Sterically more demanding benzyl electrophiles were also well tolerated. Thus, (chloromethyl)‐1,1′‐biphenyl (**2c**) and 2‐(chloromethyl)naphthalene (**2d**) reacted efficiently with **1a** and 4‐iodotoluene (**1p**), respectively, providing aryl(biphenyl)methane **3r** and aryl(2‐naphtyl)methane **3s** in 80%–85% yield.

Likewise, fluorinated and phenoxy‐substituted benzyl chlorides, such as 1‐(chloromethyl)‐4‐fluorobenzene (**2e**) and 1‐(chloromethyl)‐3‐phenoxybenzene (**2f**), as well as *o*‐ and *p*‐tolyl benzyl chlorides (**2g**, **2h**), proved to be competent coupling partners. Their reactions with **1f**, **1j**, and 2‐iodotoluene (**1q**) delivered products **3t**–**w** in 69%–74% yield. The reaction of *p*‐benzyloxybenzyl chloride (**2i**) and *p*‐methoxybenzyl chloride (**2j**) with the iodinated ester **1f** afforded diarylmethanes **3x** and **3y**, respectively, in 40%–60% yield. We next explored benzyl chlorides bearing electron‐withdrawing substituents. Methyl 4‐(chloromethyl)benzoate (**2k**) and 1‐(chloromethyl)‐3‐(trifluoromethyl)benzene (**2l**) reacted smoothly with **1p** and iodobenzene (**1r**), respectively, affording the corresponding diarylmethane derivatives **3z** and **3aa** in excellent yields (80%–95%). Unsubstituted diphenylmethane (**3ab**) was obtained in 82% yield from **1r** and **2b**. Moreover, the coupling of 4‐(chloromethyl)‐2‐fluoro‐1‐methoxybenzene (**2m**) with **1q** afforded polyfunctionalized diarylmethane **3ac** in 61% yield. A distinctive reactivity pattern was observed with 4‐iodophenol (**1s**). In its reaction with **2b**, the expected coupling product **3ad** was isolated in 25% yield, alongside a significant amount of diarylmethane **3ae** (50%). The latter likely arises from a subsequent nucleophilic substitution of the benzylic chloride in unreacted **2b** by the phenolic moiety of **3ad**.

**SCHEME 3:**
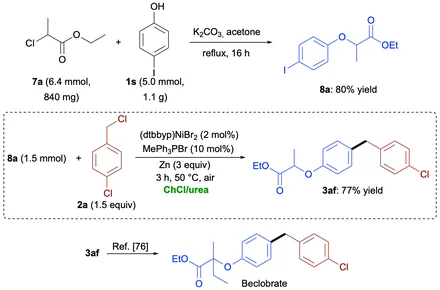
Modular synthesis of Beclobrate precursor **3af** via Ni‐catalyzed XEC between **2a** and iodophenoxy ester **8a**, the latter obtained from the reaction of 2‐chloropropanoate (**7a**) with 4‐iodophenol (**1s**).

The synthetic utility and strategic relevance of this XEC platform were further underscored by its application to the synthesis of key intermediate **3af**, en route to the lipid‐lowering drug Beclobrate (Scheme 3) [76]. Intermediate **3af** was accessed via a Ni‐catalyzed XEC reaction between benzyl chloride **2a** and ethyl 2‐(4‐iodophenoxy)propanoate (**8a**) in ChCl/urea. The iodophenoxy ester **8a** was in turn prepared in 80% yield through nucleophilic substitution of ethyl 2‐chloropropanoate (**7a**) with 4‐iodophenol (**1s**), following a slightly modified literature procedure [77, 78]. Under the optimized XEC conditions, the desired diarylmethane **3af** was isolated in 77% yield, thus enabling a direct and operationally convenient entry into Beclobrate scaffold [76] from readily available electrophiles in a green reaction medium (Scheme 3).

### 2.3 Mechanistic Studies: Evidence for Benzyl Radical Intermediates

**SCHEME 4:**
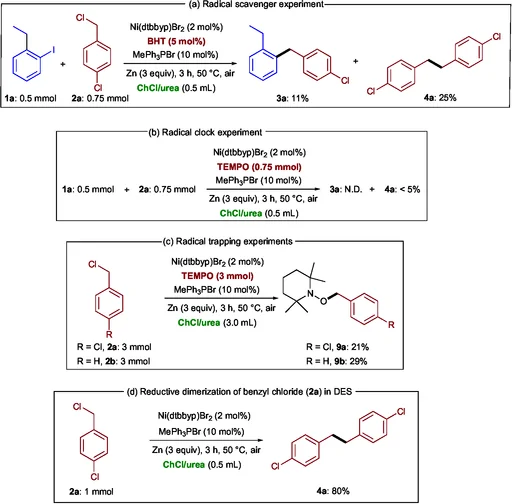
Mechanistic investigations. N.D. = not detected.

To gain deeper insight into the reaction mechanism, a series of control experiments were performed. The addition of the radical scavenger BHT [2,6‐bis(1,1‐dimethylethyl)phenol, 5 mol%] markedly suppressed the formation of **3a** (11% yield) from the reaction of **1a** with **2a**, with **4a** obtained in only 25% yield, as determined by ^1^H NMR and GC–MS analysis (Scheme 4a). Furthermore, the introduction of TEMPO (2,2,6,6‐tetramethylpiperidine‐1‐oxyl, 1.5 equiv), a well‐established radical trap and clock, completely inhibited product formation under the same conditions (Scheme 4b). These results are consistent with a radical pathway as opposed to a closed‐shell process [79]. Direct evidence for the generation of a benzyl radical was obtained when benzyl chlorides **2a**,**b** were reacted under standard conditions in the presence of TEMPO (1 equiv), leading to the isolation of stable alkoxyamine adducts **9a**,**b** in 21%–29% yield (Scheme 4c). Collectively, these findings not only provide compelling support for a radical‐mediated pathway but also deliver unambiguous proof of benzyl radicals as key intermediates. It is worth noting that, in the absence of both the aryl iodide and TEMPO, **2a** underwent clean homodimerization to furnish **4a** as the sole product in 80% yield (Scheme 4d). When the catalyst was also omitted and the reaction carried out with Zn alone, **4a** still formed, though in significantly lower yields (24%) (Table 1, entry 24). This underscores the crucial role of the catalyst in benzyl radical generation and, consequently, in the reductive dimerization of benzyl chloride, which is far from being trivial [80, 81].

### 2.4 Electrochemical Studies

To gain deeper insight into the mechanism of this nickel‐catalyzed cross‐coupling reaction, we carried out electrochemical studies. Cyclic voltammetry (CV) experiments were initially attempted in the DES medium; however, the very limited solubility of the Ni catalyst—even in the presence of the phosphonium salt—resulted in poorly defined and nonreproducible voltammograms, which precluded meaningful analysis (Figure S5). These limitations are exacerbated under CV conditions, where the absence of stirring imposes strict diffusion control.

To overcome these issues, the electrochemical investigations were conducted in DMF in the presence of the same phosphonium additive, ensuring full solubilization of the catalytic system and reliable measurements. Notably, the XEC proceeds with comparable efficiency in both DES and DMF (Table 1, entries 22,29), indicating that the key catalytic features are preserved across the two media. On this basis, the electrochemical data obtained in DMF are considered representative of the underlying redox processes operative under DES conditions.

**FIGURE 2:**
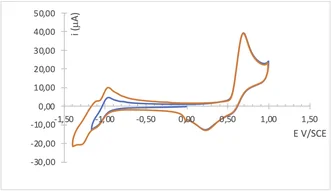
Cyclic voltammograms of (dtbbpy)NiBr_2_ 10^−2^ mol·L^−1^ in DMF + 0.1 mol·L^−1^ NBu_4_BF_4_, on GC electrode (*d* = 1 mm), scan rate 200 mV/s.

**FIGURE 3:**
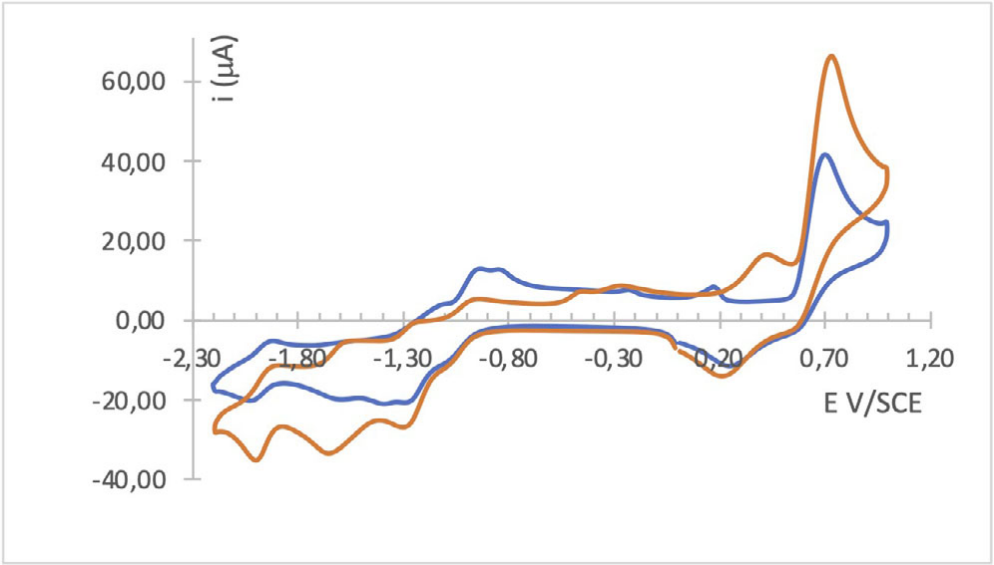
Cyclic voltammograms in DMF + 0.1 mol L^−1^ NBu_4_BF_4_, on GC electrode (*d* = 1 mm), scan rate 200 mV/s of (dtbbpy)NiBr_2_ 10^−2^ mol L^−1^ (blue curve); (dtbbpy)NiBr_2_ 10^−2^ mol·L^−1^ + MePh_3_PBr 10^−2^ mol·L^−1^ (orange curve).

The electrochemical behavior of (dtbbpy)NiBr_2_ was investigated by CV in DMF using a glassy carbon working electrode. In solution, the complex dissociates in (dtbbpy)NiBr^+^, releasing Br^–^, whose oxidation is observed at 0.68 V/SCE, consistent with the behavior reported for (bpy)NiBr_2_ [82]. Reduction of Ni(II) occurs in two distinct quasi‐reversible steps: the first reduction wave at Ep1 = –1.10 V/SCE corresponds to the one‐electron reduction to Ni(I), while the second wave at Ep2 = –1.25 V/SCE corresponds to the one‐electron reduction to Ni(0) (Figure 2). On the reverse scan, the reoxidation peaks of Ni(0) and Ni(I) appear at –1.10 and –0.95 V/SCE, respectively. A further quasi‐reversible reduction is also detected at Ep3 = –2.00 V/SCE, attributable to the reduction of dtbbpy ligand bound to Ni(0), giving Ni(0)dtbbpy/Ni(0)dtbbpy^•–^ (Equations I–IV) (Figure 3, blue curve).

Upon addition of 1 equiv of MePh_3_PBr to a DMF solution containing (dtbbpy)NiBr_2_, the CV profile changes markedly (Figure 3, orange curve). The Ni(II)/Ni(I) reduction at Ep1 remains unchanged, while the Ni(I)/Ni(0) reduction at Ep2 becomes sharper and more defined. Importantly, a new reduction feature appears at Ep4 = –1.65 V/SCE, which cannot be assigned to free phosphonium (Epc = –1.95 V/SCE; Figure S2). This new event was attributed to the reduction of a phosphonium‐associated Ni(0) complex, consistent with the absence of ligand‐free phosphonium reduction in this region, while retaining its characteristic oxidation at 0.45 V/SCE. The addition of 5 equiv of MePh_3_PBr relative to Ni(II) resulted in essentially identical electrochemical behavior, with the only notable difference being an increased cathodic signal at −1.95 V/SCE, attributable to the reduction of the free phosphonium species (Figure S6).

(I) $$(\mathrm{dtbbpy}){\mathrm{Ni}}^{\mathrm{II}}{\mathrm{Br}}_{2}\;\to \;(\mathrm{dtbbpy}){\mathrm{Ni}}^{\mathrm{II}}{\mathrm{Br}}^{+}\;\;\;\;+{\mathrm{Br}}^{-}$$

(II)

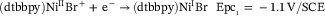

(III) $$(\mathrm{dtbbpy}){\mathrm{Ni}}^{I}\mathrm{Br}\;+\;e^{-}\;\to \;(\mathrm{dtbbpy}){\mathrm{Ni}}^{0}\;\;\;\;\;\;{\mathrm{Epc}}_{2}\;=\;-1.25\;V/\mathrm{SCE}$$

(IV) $$(\mathrm{dtbbpy}){\mathrm{Ni}}^{0}\;+\;e^{-}\;\;\;\to \;(\mathrm{dtbbpy}){{\mathrm{Ni}}^{0.}}^{-}\;\;\;\;{\mathrm{Epc}}_{3}=\;-2.0\;V/\mathrm{SCE}\;$$

**FIGURE 4:**
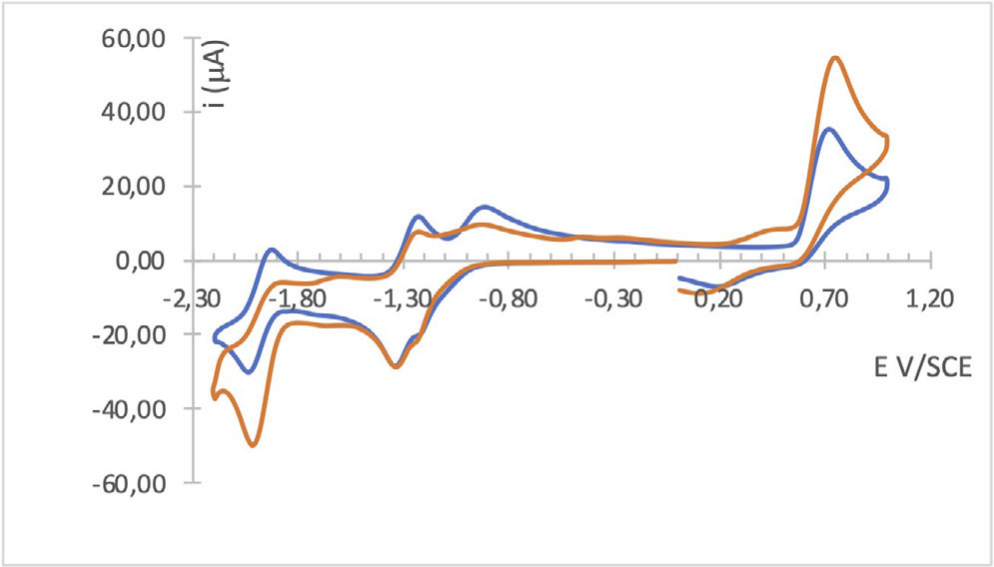
Cyclic voltammograms in DMF + 0.1 mol L^−1^ NBu_4_BF_4_, on GC electrode (*d* = 1 mm), scan rate 200 mV/s of (dtbbpy)_2_NiBr_2_ 10^−2^ mol L^−1^ (blue curve); (dtbbpy)_2_NiBr_2_ 10^−2^ mol L^−1^ + MePh_3_PBr 10^−2^ mol L^−1^ (orange curve).

Because (dtbbpy)Ni salts are weakly coordinating [83], addition of excess dtbbpy (2 equiv per Ni) leads to the formation of a more coordinated Ni complex, in which the three reduction steps are well resolved (Figure 4, blue curve). Under these conditions, the addition of 1 equiv of MePh_3_PBr does not significantly perturb the Ni(II)/Ni(I) and Ni(I)/Ni(0) redox couples. However, the Ep4 feature is markedly reduced in intensity, while a cathodic signal at –1.95 V/SCE, attributable to the reduction of free phosphonium species, becomes evident (Figure 4, orange curve).

**FIGURE 5:**
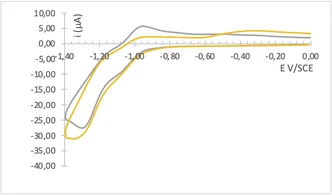
Cyclic voltammograms in DMF + 0.1 mol L^−1^ NBu_4_BF_4_, on GC electrode (*d* = 1 mm), scan rate 200 mV/s of (dtbbpy)NiBr_2_ 10^−2^ mol L^−1^ + MePh_3_PBr 10^−2^ mol L^−1^ (gray curve); (dtbbpy)NiBr_2_ 10^−2^ mol L^−1^ + MePh_3_PBr 10^−2^ mol L^−1^ + BnCl 10^−2^ mol L^−1^ (yellow curve).

Addition of 1 equiv benzyl chloride to the phosphonium/(dtbbpy)NiBr_2_ solution modifies the CV. The oxidation peak corresponding to Ni(I) reoxidation decreases in intensity during the reverse scan, indicating that electrogenerated Ni(I) reacts chemically with benzyl chloride (Figure 5, gray and yellow curves). We propose that Ni(I) species transfers an electron via a SET or a concerted halogen‐atom dissociation pathway to benzyl chloride to form a benzyl radical, regenerating Ni(II). Consistent with this assignment, a catalytic current develops in the nickel redox region. Extending the reverse scan to –1.90 V/SCE produces a further increase in current intensity without the appearance of a new redox couple (Figure S3).

**FIGURE 6:**
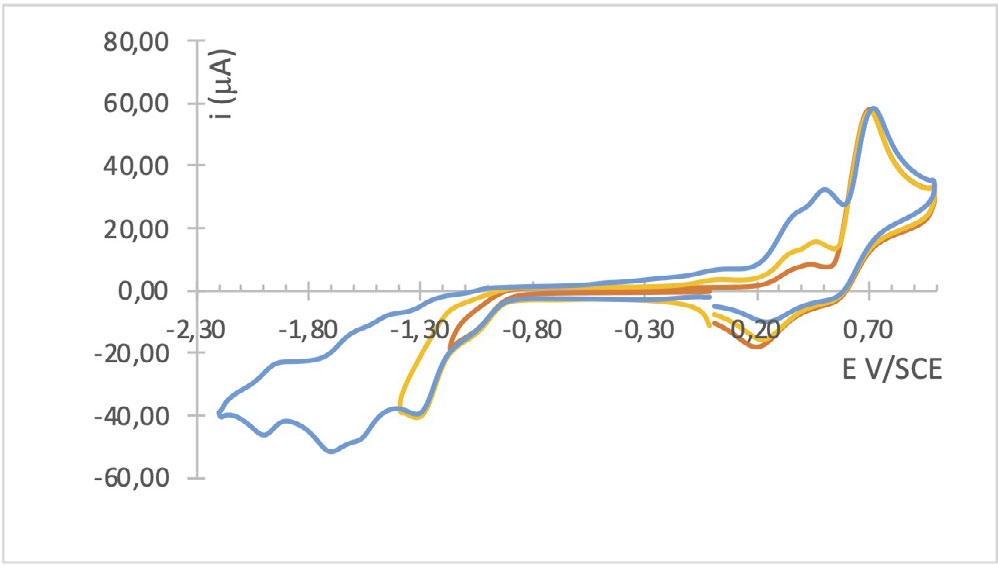
Cyclic voltammograms in DMF + 0.1 mol·L^−1^ NBu_4_BF_4_, on GC electrode (*d* = 1 mm), scan rate 200 mV/s of (dtbbpy)NiBr_2_ 10^−2^ mol·L^−1^ + MePh_3_PBr 10^−2^ mol·L^−1^ + ArI 10^−2^ mol·L^−1^: −1.15 V, orange curve; −1.4 V, yellow curve; −2.2 V, blue curve.

Addition of 1 equiv 2‐iodoanisol to the phosphonium/(dtbbpy)NiBr_2_ solution leads to disappearance of Ni(I) reoxidation during the reverse scan (Figure 6, orange curve), confirming oxidative addition of electrogenerated Ni(I) to 2‐iodoanisol. When the reverse scan extends to −1.40 V/SCE, reoxidation of Ni(0) also vanishes, while a catalytic current emerges in the Ni(I)/Ni(0) region along (Figure 6, yellow curve) with a new reduction process at −1.60 V/SCE (Figure 6, blue curve), consistent with the reduction of an arylnickel species formed by oxidative addition of Ni(I) [or Ni(0)] to aryl iodide. A corresponding oxidation event at 0.35V/SCE is attributed to reoxidation of arylnickel.

**FIGURE 7:**
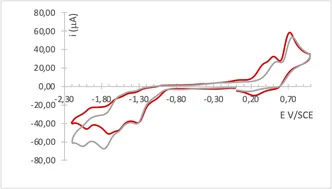
Cyclic voltammograms in DMF + 0.1 mol·L^−1^ NBu_4_BF_4_, on GC electrode (*d* = 1 mm), scan rate 200 mV/s of (dtbbpy)NiBr_2_ 10^−2^ mol·L^−1^ + MePh_3_PBr 10^−2^ mol·L^−1^ + ArI 10^−2^ mol·L^−1^ (red curve); (dtbbpy)NiBr_2_ 10^−2^ mol·L^−1^ + MePh_3_PBr 10^−2^ mol·L^−1^ + ArI 10^−2^ mol·L^−1^ + BnCl 10^−2^ mol·L^−1^ (gray curve).

Finally, addition of 1 equiv benzyl chloride to the phosphonium/(dtbbpy)NiBr_2_/aryl iodide system (Figure 7, red curve) further modifies the voltammogram (Figure 7, gray curve). The intensity of Ep4 at −1.65 V/SCE increases and the arylnickel oxidation at 0.35 V/SCE disappears, consistent with reaction between arylnickel species and benzyl radical generated following reduction of Ni(II) in the presence of benzyl chloride.

When the electrochemistry of (dtbbpy)_2_NiBr_2_ was reexamined in the presence of the phosphonium salt, two quasi‐reversible reductions corresponding to Ni(II)/Ni(I) and Ni(I)/Ni(0) were observed (Figure S4). As discussed above, the saturated Ni complex does not interact detectably with phosphonium, and Ep4 is almost absent. Addition of aryl iodide again led to disappearance of Ni(I) reoxidation while leaving peak height unchanged. The Ni(II)/Ni(I) system is slightly shifted toward more positive potentials, suggesting that a chemical reaction occurs after the electrochemical reduction. This clearly demonstrates that oxidative addition proceeds from Ni(I) using both (dtbbpy)NiBr^+^ and (dtbbpy)_2_NiBr_2_ precursors.

### 2.5 Proposed Mechanism

**FIGURE 8:**
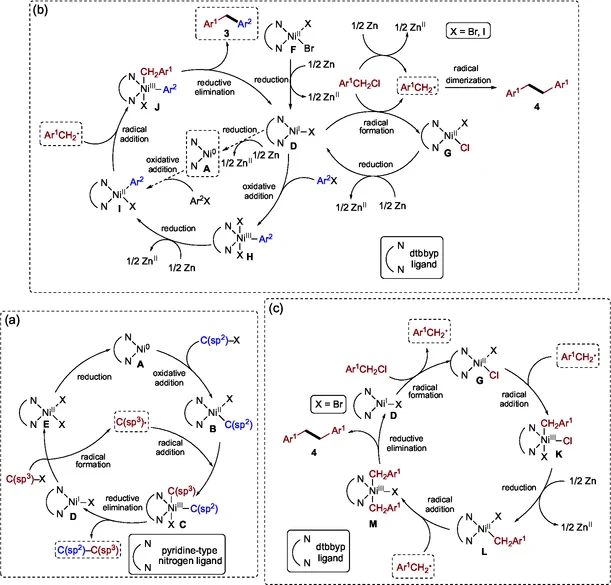
(a) Predominant mechanism for Ni‐catalyzed reductive couplings between aryl and alkyl halides in the presence of pyridine‐type nitrogen ligands and a reductant in conventional solvents; (b) proposed mechanism for Ni‐catalyzed reductive couplings between benzyl chlorides and aryl iodides in DESs; (c) proposed mechanism for Ni‐catalyzed reductive dimerization of benzyl chlorides.

The prevailing mechanistic paradigm for Ni(0)‐catalyzed reductive XEC between C(sp^3^) and C(sp^2^) electrophiles, in the presence of pyridine‐type nitrogen ligands and Zn (or Mn) as reductant, has been established through detailed mechanistic investigations [84, 85, 86]. This mechanism integrates polar and radical pathways and typically involves five elementary steps (Figure 8a): (a) oxidative addition of an aryl halide [C(sp^2^)–X] to Ni(0) species **A**, affording a Ni(II)–aryl complex **B**; (b) single‐electron transfer (SET) to the alkyl halide [C(sp^3^)–X], most commonly from a Ni(I) species, generating an alkyl radical species [C(sp^3^)•], which subsequently combines with **B** to form a diorgano‐nickel(III) intermediate **C**; (c) reductive elimination from **C**, delivering the cross‐coupled product [C(sp^2^)–C(sp^3^)] and a Ni(I) species **D**; (d) oxidation of **D** to a Ni(II) species **E** upon reaction with C(sp^3^)–X; and (e) reduction of **E** to regenerate Ni(0) species **A**, thus closing the catalytic cycle. Key features of this mechanism include: (i) aryl halides undergo oxidative addition to Ni(0) more readily than alkyl halides, owing to favorable *π*–metal interactions; (ii) alkyl halides are more prone to radical formation due to the higher thermodynamic stability of alkyl radicals; (iii) radical generation is commonly initiated at Ni(I) halide complexes, which are proposed to propagate C(sp^3^) radicals through halogen‐atom abstraction from the alkyl substrate. The resulting cage‐escaped radical then recombines with a distinct arylnickel species after oxidative addition.

Our mechanistic studies (Scheme 4) indicate that *benzyl radicals are primarily generated directly from benzyl chlorides*, *even in the absence of aryl iodides*. Of note, in the absence of nickel, the homodimerization product **4a** is still formed, albeit in significantly lower yield (24%; Table 1, entry 24), indicating that zinc alone can promote radical generation to a limited extent. The electrochemical signatures demonstrate that the Ni(II)/Ni(I)/Ni(0) redox manifold is accessible under the reducing conditions employed, and therefore do not exclude further reduction of Ni(I) to Ni(0) during turnover. Electrogenerated Ni(I) species react productively with both benzyl chloride and aryl iodide, as evidenced by the attenuation and shifting of redox waves upon addition of these electrophiles, together with the appearance of catalytic currents. Collectively, these observations are consistent with a sequence involving SET activation of benzyl chloride, oxidative addition of aryl iodide, and recombination of a transient benzyl radical with arylnickel intermediates. In addition, the emergence of new redox features upon introduction of the phosphonium salt suggests a specific interaction with reduced nickel species, consistent with a role in stabilizing Ni(I)/Ni(0) intermediates, modulating catalyst speciation, and sustaining catalytic turnover.

On this basis, we propose the mechanistic scenario depicted in Figure 8b. The initial Ni(II) precatalyst **F** would plausibly be reduced to a stabilized Ni(I) species **D** [87], which transfers an electron to benzyl chloride either via SET or through a concerted halogen‐atom dissociation pathway [88], thereby generating a benzyl radical and regenerating a Ni(II) species (**G**). Partial formation of benzyl radical via direct reduction of benzyl chloride by zinc cannot, however, be ruled out (Scheme 4d; Table 1, entry 24). The resulting Ni(II) species **G** can then be directly reduced back to Ni(I) **D**, which undergoes oxidative addition with aryl iodide to form a Ni(III)‐aryl intermediate **H**. Subsequent reduction of **H** furnishes a Ni(II) species **I**, which captures the benzyl radical to generate a diorgano‐Ni(III) complex **J**. Reductive elimination from **J** delivers the cross‐coupled product **3**, while regenerating the catalytically active Ni(I) species **D** [89]. A parallel pathway involving further reduction of Ni(I) **D** to Ni(0) **A**, followed by oxidative addition of the aryl iodide to directly form Ni(II) **I**, cannot be excluded.

The observed cross‐selectivity likely reflects competition between productive radical capture by **I** and self‐recombination of benzyl radicals leading to the homodimeric byproduct **4**. Alternatively, a nickel‐mediated pathway may also account for the formation of **4** (Figure 8c). Following initial benzyl radical generation, two such radicals could sequentially be captured by Ni(II) species **G** and **L.** This pathway would involve reduction of a transient Ni(III)(Bn)XCl intermediate **K** to Ni(II)(Bn)X **L**, ultimately affording a bis(benzyl)‐Ni(III) complex (**M**). Reductive elimination from **M** would then furnish bibenzyl **4** while regenerating the Ni(I)X catalyst **D**. This proposal is supported by several studies documenting radical generation via single‐electron oxidative activation of alkyl halides by Ni(I) species, as well as reductive elimination from diorgano‐Ni(III) intermediates [90, 91].

The distinctive feature of this XEC process, optimized in the ChCl/urea DES, lies in the efficient generation and selective control of benzyl radicals [most plausibly arising from Ni(I) species], whose reactivity can be effectively controlled to promote productive cross‐coupling while suppressing unselective pathways such as radical homodimerization. Such chemoselectivity was challenging to achieve in ealier Ni‐catalyzed systems conducted in conventional dipolar aprotic solvents such as DMA. Indeed, Weix reported that a bipyridine‐ligated nickel catalyst [(dtbbpy)NiBr_2_] in DMA with zinc as reductant afforded only 4% of the desired diarylmethane from benzyl bromide and iodobenzene, while bibenzyl formed in 48%. Under these conditions, benzyl electrophiles preferentially react with Ni(0), necessitating the use of benzyl mesylate and cobalt phthalocyanine co‐catalysis to bias the system toward aryl–Ni(II) formation [43].

In contrast, this limitation is effectively mitigated in the DES medium. As demonstrated by Stahl et al., the Zn^2+^/Zn redox potential is highly solvent‐dependent, shifting by several hundred millivolts across common amide solvents and correlating with solvent dielectric constant and donor ability [92]. Notably, the presence of halide additives (e.g., chloride salts) shifts the Zn^2+^/Zn couple to more negative potentials through stabilization of Zn^2+^ as halide complexes (e.g., ZnCl_4_^2−^), thereby enhancing the reducing power of zinc. In chloride‐rich environments such as ChCl/urea, an analogous—yet intrinsically amplified—effect is expected to operate. The high chloride activity promotes extensive coordination to Zn^2+^, effectively driving the redox potential to more negative values and thereby increasing zinc's reducing ability under operational conditions. In addition to these thermodynamic effects, DES exert a decisive kinetic contribution. Choline chloride‐based DESs have been shown to facilitate zinc activation through efficient removal of the passivating oxide layer and improved surface wetting, thus eliminating the need for external acidic activators typically required in conventional amide solvents. This behavior is fully consistent with prior literature reports [72, 73, 74, 75]. Taken together, these synergistic thermodynamic and kinetic effects—largely absent in conventional media—provide a compelling rationale for both the enhanced reactivity (complete conversion within 3 h) and the improved selectivity observed in this XEC protocol under DES conditions. More broadly, these findings highlight the capacity of nonconventional solvent systems to modulate metal redox properties and surface reactivity, thereby unlocking new reactivity paradigms in XEC chemistry.

## 3 Conclusion

In summary, we have developed a nickel‐catalyzed XEC between aryl iodides and benzyl chlorides in a deep eutectic solvent (DES) based on choline chloride/urea (1:2 mol/mol). The transformation proceeds under mild, aerobic, and heterogeneous conditions (3 h at 50 °C), providing a broad range of diarylmethanes in good to excellent yields (up to 95%), with high chemoselectivity, broad functional‐group tolerance, and scalability to gram quantities, while avoiding the use of conventional amide solvents or inert atmosphere. Optimal performance is achieved using a bipyridine‐ligated nickel catalyst [(dtbbpy)NiBr_2_, 2 mol%], zinc (3 equiv) as the terminal reductant, and a phosphonium salt additive (MePh_3_PBr, 10 mol%).

Mechanistic and electrochemical studies are consistent with a radical/polar crossover scenario, in which an accessible Ni(II)/Ni(I)/Ni(0) redox manifold enables sequential single‐electron activation of benzyl chlorides, oxidative addition of aryl iodides, and recombination of benzyl radicals with arylnickel intermediates, most likely operating through a Ni(I)/Ni(II)/Ni(III) catalytic cycle. The phosphonium additive appears to play a multifaceted role, contributing to the modulation of nickel speciation, stabilization of reduced Ni intermediates, and suppression of unproductive radical dimerization pathways. Given the heterogeneous nature of the reaction—where both zinc and organic substrates are largely insoluble in the eutectic phase—a contribution related to interfacial processes may be envisaged, potentially enhancing productive interactions between the solid reagents and the catalytically active fraction of nickel species dissolved in the DES. However, such an effect should be regarded as a possible contributing factor rather than a demonstrated mechanism.

Beyond serving as a benign solvent, the halide‐rich DES environment likely contributes to reactivity by enhancing the effective reducing power of zinc through stabilization of Zn^2+^ chloride complexes, as well as by promoting favorable surface activation and electron‐transfer kinetics relative to amide solvents. These combined thermodynamic and interfacial effects underpin the accelerated reaction rates and improved chemoselectivity observed in DESs, highlighting solvent‐dependent modulation as a powerful strategy for controlling radical reactivity in XEC.

Ongoing work in our laboratory is focused on expanding the electrophile scope, developing tailored DES formulations, and integrating electrochemical or photochemical redox control. Collectively, these advances position DESs as versatile and enabling platforms for Ni‐catalyzed XEC reactions and provide a foundation for the development of more sustainable and industrially relevant synthetic methodology.

## Funding

This work was supported by the Ministero dell'Università e della Ricerca (2022KMS84P), Ministero dell’Ambiente e della Sicurezza Energetica (RSH2A_000004), LabEx SynOrg (ANR‐11‐LABX‐0029), XL‐Chem Graduate School of Research (ANR‐18‐EURE‐0020 XL CHEM), Région Normandie, University of Rouen Normandy, INSA Rouen Normandy, Centre National de la Recherche Scientifique, European Regional Development Fund, and Carnot Institute I2 C.

## Conflicts of Interest

The authors declare no conflicts of interest.

## Supporting information

The authors have cited additional references within the Supporting Information section [93, 94, 95, 96, 97, 98, 99, 100, 101, 102, 103, 104, 105, 106, 107, 108, 109, 110, 111, 112].

## Data Availability

The data that supports the findings of this study are available in the supporting information of this article.
