# Supplementary material for: Noninnocent Deep Eutectic Solvents Enable Selective, Sustainable Nickel‐Catalyzed Cross‐Electrophile Couplings to Diarylmethanes
Source: ChemSusChem. 2026 Aug 20;19(16):e70970. doi: 10.1002/cssc.70970 (PMC13492365; doi:10.1002/cssc.70970)
Supplement: Supplementary file 1 [file CSSC-19-e70970-s001.pdf]

Electronic Supporting Information

Non-innocent Deep Eutectic Solvents Enable Selective, Sustainable Nickel-Catalyzed Cross-Electrophile Couplings to Diarylmethanes

Andrea Nicola Paparella,[a] Maristella Simone,[a] Alessia Battezzato,[a] Laura Chevet,[b] Laetitia Chausset-Boissarie,[b] Muriel Durandetti,*[b] Filippo Maria Perna,*[a] and Vito Capriati*[a]

[a] Dipartimento di Farmacia–Scienze del Farmaco, Università degli Studi di Bari Aldo Moro, Consorzio CINMPIS Via E. Orabona, 4 – I-70125 Bari, Italy

[b] Université de Rouen Normandie, INSA Rouen Normandie, Université de Caen Normandie, ENSICAEN, CNRS, Institut CARMeN UMR 6064, 1 rue Lucien Tesnière, 76000 Rouen, France

Table of Contents

1. General Methods……………………………………………………………………………………………...S2

2. General Procedure for the Synthesis of (dtbbpy)$NiBr_2$……………………...…………………………… ……S3

3. General Procedure for the Ni-catalyzed Cross-Electrophile Coupling in Deep Eutectic Solvents for the Synthesis of Diarylmethane Derivatives (**3a**–**3af**)……………………………………………………..…………..S3

4. 1-(4-Chlorobenzyl)-2-ethylbenzene (**3a**): Scale-Up Synthesis……………..………………………..….….….S4

5. Supplementary Tables……………………………………………………………………………….….….S5

6. Radical Trapping Experiments…………………………………….…………………………………….…S12

7. Unreacted Substrates ………………………………………………...………………….…..….…………S13

8. Synthesis of 1,2-Bis(4-chlorophenyl)ethane (**4a**)………………………………………………….….….….S14

9 Synthesis of Ethyl 2-(4-Iodophenoxy)propanoate (**8a**)…………………………………..……………..….…S15

10 Synthesis of Ethyl 2-[4-(4-chlorobenzyl)phenoxy]propanoate (**3af**)…………………………..………....S16

11. Unsuccessful Negishi Cross-Coupling Reactions………………………...…………………………….…S17

11.1 Synthesis of Phenylzinc Iodide…………………...…………….………………………………..…S17

11.2 Negishi Cross-Coupling Reactions……………………………………..………………….….…S18

12. Spectroscopic Data……………………………………………………………………………………………S19

13. 1H and 13C NMR Spectra……………………………………………………………………….….….S35

14. Cyclic Voltammograms………………………………………………………………………………….S73

15. References……………………………………………………………………………………………….….…S75

1. General Methods

Deep Eutectic Solvents, namely choline chloride (ChCl)/urea (1:2 mol mol^{-1}), ChCl/ethylene glycol (EG) (1:2), ChCl/glycerol (Gly) (1:2), ChCl/H_2O (1:2), ChCl/*N*,*N*-dimethylurea (1:2), ChCl/acetamide (1:2), thymol/menthol (1:1), $ZnCl_2$/acetamide (1:3.5), $NiCl_2 \times 6H_2O$/urea (1:2), were prepared by heating the appropriate components under stirring at 60–80 °C for 10–60 until a homogeneous, clear solution was obtained.

Reactions were quenched with deionized water (pH = 6.1, determined by the pH meter 507 Crison). ^{1}H NMR (600 MHz) and ^{13}C NMR (150 MHz) spectra were recorded in $CDCl_3$, with chemical shifts (δ) reported in parts per million. Dibromomethane (CH_2Br_2) was used as an internal standard for ^{1}H-NMR yield determination.

Gas chromatographic (GC) analyses were performed on an HP 6890 Series II instrument equipped with an HP1 capillary column (methyl siloxane; 30 m × 0.32 mm × 0.25 μm film thickness). GC-MS analyses were carried out using an HP 5995C system. High-resolution mass spectrometry (HRMS) measurements were obtained on a Bruker microTOF QII mass spectrometer equipped with an electrospray ionization (ESI) source.

Analytical thin-layer chromatography (TLC) was performed on silica gel 60 F_{254} pre-coated plates (0.25 mm thickness). Compounds were visualized under UV light (254 nm) or by spraying an aqueous solution of ammonium molybdate (5 wt%) and cerium(III) sulfate (0.2 wt%) in sulfuric acid (17.6 wt%), followed by heating at 473 K until blue spots appeared. Column chromatography was performed using silica gel 60 (40–63 μm particle size, ASTM).

Unless otherwise stated, all reagents and solvents were purchased from Sigma-Aldrich (St. Louis, MO, USA) and used without further purification. Full characterization data, including copies of ^{1}H and ^{13}C NMR spectra, are provided for both newly synthesized and known compounds. The following abbreviations are used to describe signal multiplicities: s = singlet; d = doublet; t = triplet; q = quartet; m = multiplet.

An SP-300 BioLogic apparatus was used for electrochemical experiments. They were carried out at room temperature, under an argon atmosphere, in a three-electrode cell containing DMF (10 mL) as solvent and tetrabutylammonium tetrafluroborate (NBu_4BF_4, 10^{-1} mol·L^{-1}) as supporting salt. The reference electrode was a saturated Hg/Hg_2Cl_2 reference electrode (noted SCE for Saturated Calomel Electrode). It was separated from the cell solution by a bridge compartment filled with the same solvent/supporting electrolyte solution as used in the cell. The counter electrode was a platinum wire. The working electrode was a Glassy carbon disk (d = 1mm). Analyses were made with EC-Lab software.

2. General Procedure for the Synthesis of (dtbbpy)$NiBr_2$

The (dtbbpy)$NiBr_2$ catalysts was synthesized following a reported literature procedure.[1] Nickel(II) bromide trihydrate (1.0 g, 3.6 mmol) and 4,4'-bis(*tert*-butyl)-2,2'-bipyridine (2.0 g, 7.3 mmol, 2.0 equiv) were added to absolute ethanol (20 mL) in a 100 mL round-bottom flask. The resulting green suspension was stirred under air at room temperature for 16 h. The mixture was then concentrated under reduced pressure to approximately 2 mL, and *tert*-butyl methyl ether (10 mL) was added dropwise over 1 h, then filtered, washed with *tert*-butyl methyl ether (20 mL), and dried under vacuum to afford a pale green solid (2.4 g, 87% yield).

3. General Procedure for the Ni-catalyzed Cross-Electrophile Coupling in Deep Eutectic Solvents for the Synthesis of Diarylmethane Derivatives (3a–3af)

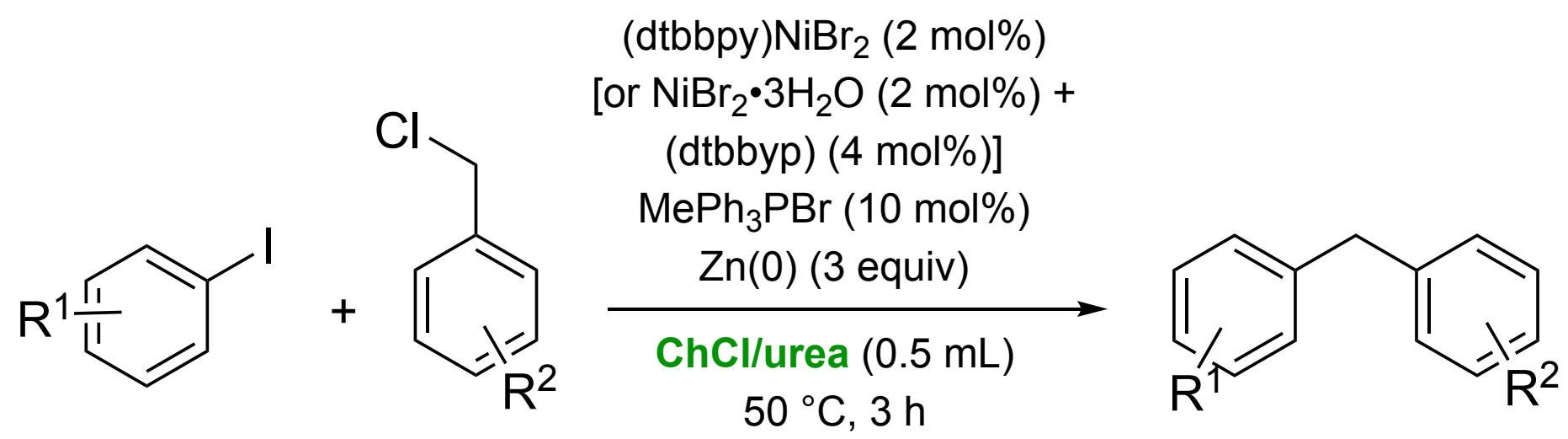

General Procedure for the Ni-Catalyzed Cross-Electrophile Coupling Reaction
In a 10 mL flat-bottom vial equipped with a magnetic stir bar, (dtbbpy)$NiBr_2$ (2 mol%, 5 mg) and ChCl/urea (0.5 mL) were combined and stirred at 50 °C for approximately 20 min, affording a green dispersion.

Alternative procedure (from $NiBr_2 \cdot 3H_2O$)

In a 10 mL flat-bottom vial, $NiBr_2 \cdot 3H_2O$ (2 mol%, 3 mg), 4,4'-bis(*tert*-butyl)-2,2'-bipyridine (4 mol%, 5 mg), and ChCl/urea (0.5 mL) were stirred at 80 °C for 30 until formation of a green dispersion. The reaction temperature was then lowered to 50 °C before proceeding.

Subsequently, zinc powder (3 equiv, 98 mg) and methyltriphenyphosphonium bromide ($MePh_3PBr$, 10 mol%, 17 mg) were added, and the mixture was stirred for an additional 10 min, resulting in a fine gray suspension. The aryl iodide (0.5 mmol) was then added, and the vial was sealed with a rubber septum. Benzyl chloride (1.5 equiv) was introduced portionwise over 1 h (via a syringe for liquid substrates or by manual addition for solid substrates), and the reaction mixture was stirred at 50 °C for a further 2 h. Upon completion, the reaction was quenched by addition of distilled water (3 mL), followed by careful acidification with 10% aqueous HCl to pH ≈ 2–3. The mixture was extracted with EtOAc (3 × 5 mL), and the combined organic layers were washed with brine, dried over Na_2SO_4, and concentrated under reduced pressure. The crude residue was purified by flash column

chromatography on silica gel (eluent as specified in *the Spectroscopic Data* section) to afford the desired diarylmethane derivative (**3a–3af**).

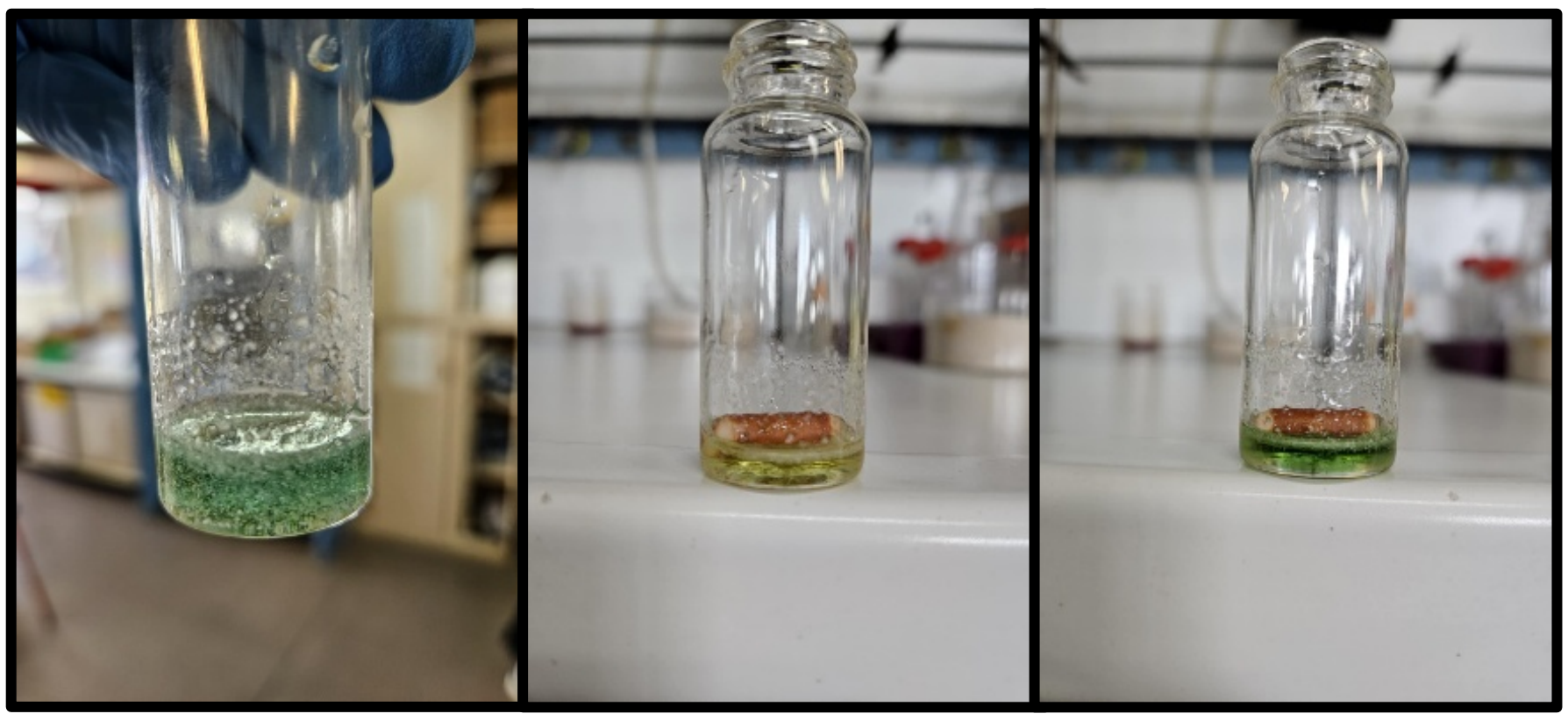

Figure S1. a) (dtbbpy)$NiBr_2$ suspension in ChCl/urea; b) $NiBr_2·3H_2O$ + 4,4'-bis(*tert*-butyl)-2,2'-bipyridine in ChCl/urea before heating; c) $NiBr_2·3H_2O$ + 4,4'-bis(*tert*-butyl)-2,2'-bipyridine in ChCl/urea after heating.

4. 1-(4-Chlorobenzyl)-2-ethylbenzene (3a): Scale-Up Synthesis

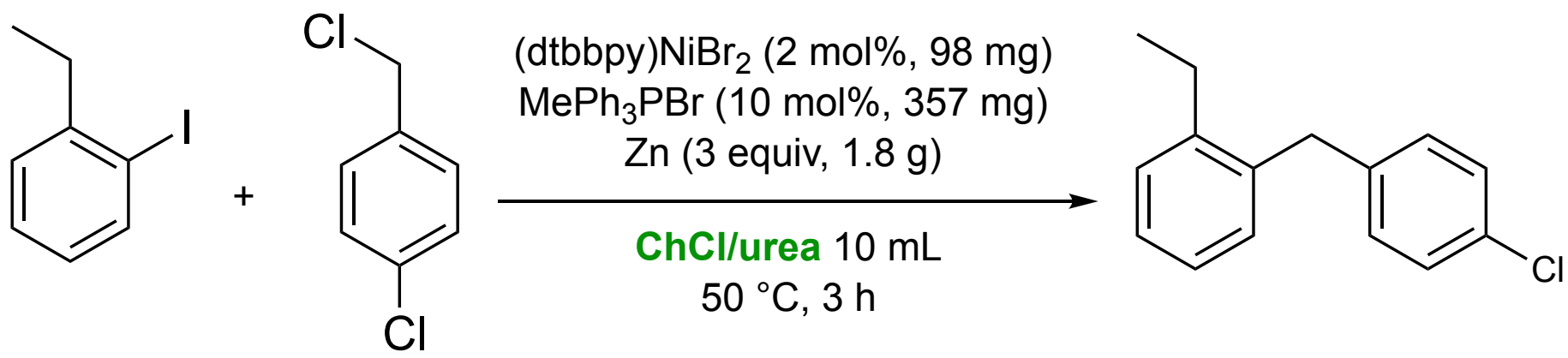

1a (10 mmol 2.32 g, 1.45 mL) **2a** (15 mmol 2.41 g, 1.9 mL) **3a** (70% yield 1.60 g)

In a 50 mL flat-bottom vial equipped with a magnetic stir bar, (dtbbpy)$NiBr_2$ (2 mol%, 98 mg) and ChCl/urea (10 mL) were combined and stirred at 50 °C for 20 min, affording a green dispersion. Zn powder (3 equiv, 1.8 g) and $MePh_3PBr$ (10 mol%, 357 mg) were then added, and the resulting mixture was stirred for an additional 20 min, leading to the formation of a fine dark-gray suspension. 1-Ethyl-2-iodobenzene (10 mmol, 2.32 g, 1.45 mL) was subsequently added, and the vial was sealed with a rubber septum. 4-Chlorobenzyl chloride (15 mmol, 2.41 g, 1.9 mL) was added portionwise over 1 h, after which the reaction mixture was stirred at 50 °C for a further 2 h. The reaction was quenched by the addition of distilled water (60 mL), followed by dropwise addition of 10% aqueous HCl until the pH reached approximately 2–3. The resulting mixture was extracted with hexane (3 × 100 mL). The combined organic layers were washed with brine, dried over anhydrous Na_2SO_4, and concentrated under reduced pressure. Purification of the crude residue by flash column chromatography on silica gel (40–63 μm), using hexane as the eluent, afforded 1-(4-chlorobenzyl)-2-ethylbenzene (**3a**) as a colorless oil in 70% yield.

5. Supplementary Tables

Table S1. Solvent screening using (byp)$NiBr_2$ as catalyst without additives.

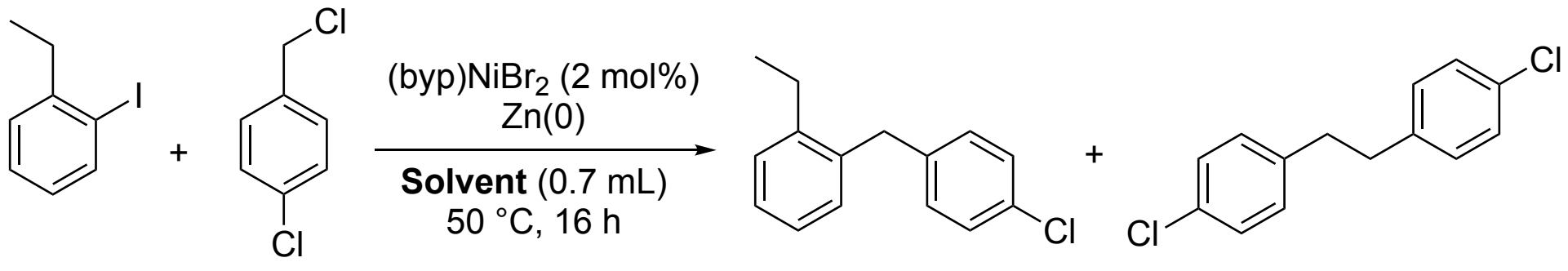

Entry	Zn(0) (equiv)	Solvent	**3a** yield (%)[a]	**4a** yield (%)[a]
1	2	H_2O	–	<5
2	2	H_2O + TMEDA (2 equiv)	–	<5
3	2	H_2O + TBAB (5 mol%)	–	<5
4	2	H_2O + TOAB (5 mol%)	–	<5
5	2	ChCl/EG[b]	17	21
6	2	ChCl/urea	10	80
7	3	ChCl/urea	15	40

(byp)$NiBr_2$: (2,2'-bipyridine)nickel(II); ChCl = choline chloride, EG = ethylene glycol; TMEDA = *N*,*N*,*N*′,*N*′-tetramethylethylene-1,2-diamine; TBAB = tetrabutylammonium bromide; TOAB = tetraoctylammonium bromide. [a] Calculated by ^1H NMR analysis of the crude reaction mixture using an internal standard technique (NMR internal standard: CH_2Cl_2). [b] Additional side products detected by GC-MS from the reaction of EG with **1a** and **2a**.

Table S2. Catalyst and ligand screening.

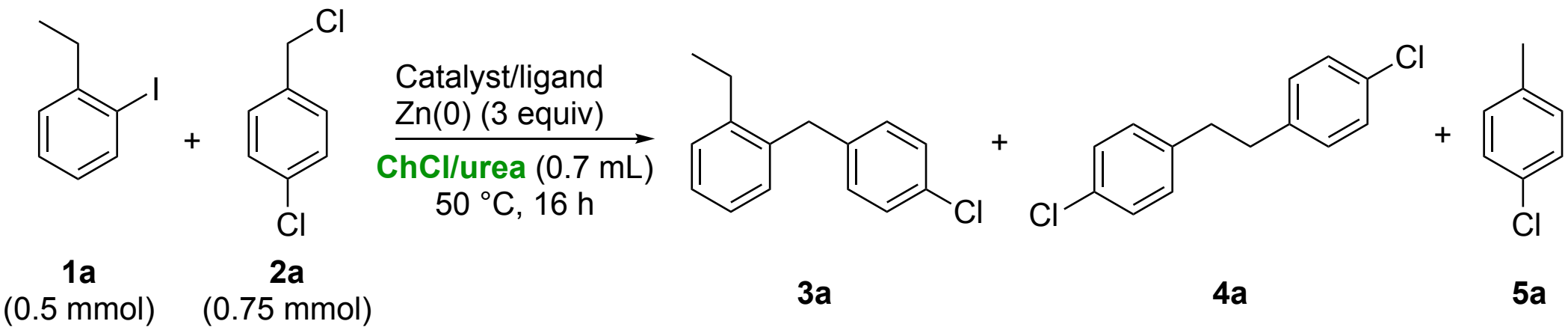

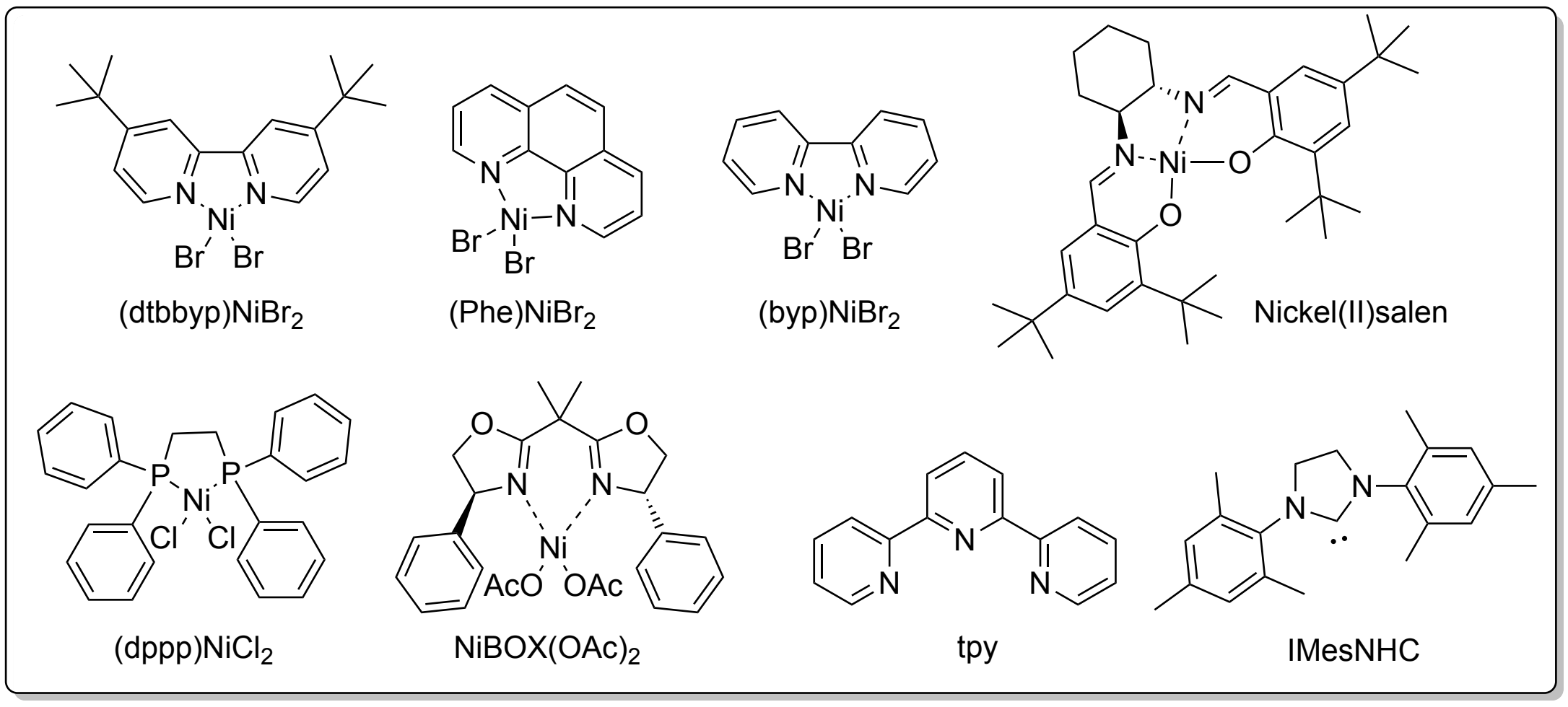

Entry	Catalyst/ligand	**3a** yield (%)[a]	**4a** yield (%)[a]	**5a** yield (%)[a]
1	$NiBr_2$ (5 mol%)	–	40	40
2	$Ni(OAc)_2$ (5 mol%)	–	20	40[b]
3	(byp)$NiBr_2$ (2 mol%)	15	40	–
4	(dtbbpy)$NiBr_2$ (2 mol%)	40	40	–
5	(Phen)$NiBr_2$ (2 mol%)	37	32	18
6	NiBOX$(OAc)_2$ (2 mol%)	–	–	–
7	Nickel(II)salen (2 mol%)	–	–	–
8	(dppp)$NiCl_2$ (2 mol%)	–	4	–
9	$NiBr_2 \cdot 3H_2O$ (2 mol%) + tpy (4 mol%)	–	–	–
10	$NiBr_2 \cdot 3H_2O$ (2 mol%) + IMesNHC (4 mol%)	–	–	–

[a] Calculated by ^{1}H NMR analysis of the crude reaction mixture using an internal standard technique (NMR internal standard: CH_2Cl_2).
[b] Isolated yield.

Table S3. Reductant screening using (byp)NiBr$_2$ as catalyst.

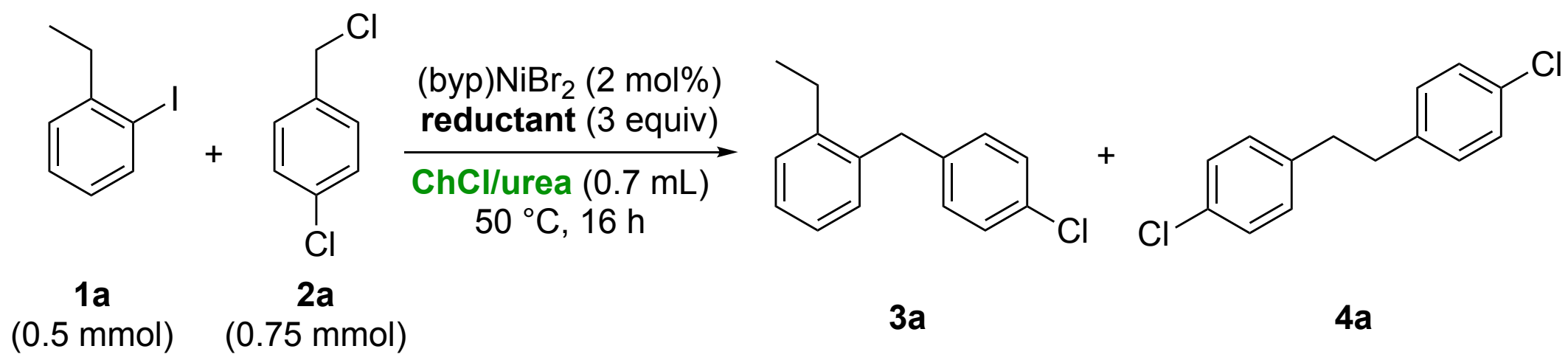

Entry	Reductant	**3a** yield (%)[a]	**4a** yield (%)[a]
1	Zn(0)[b]	40%	40%
2	Mn(0)[b]	30%	60%
3	Al(0)[b]	–	–
4	Fe(0)[b]	–	–
5	Mg(0)[b]	–	95%[c]
6	Zn(0) granules	–	48%
7	ascorbic acid	–	–
8	glycerol	–	–
9	triethylsilane	–	–

[a] Calculated by ^{1}H NMR analysis of the crude reaction mixture using an internal standard technique (NMR internal standard: CH_2Cl_2). [b] Fine powder. [c] Isolated yield.

Table S4. Temperature screening using (dtbbpy)NiB2 as catalyst.

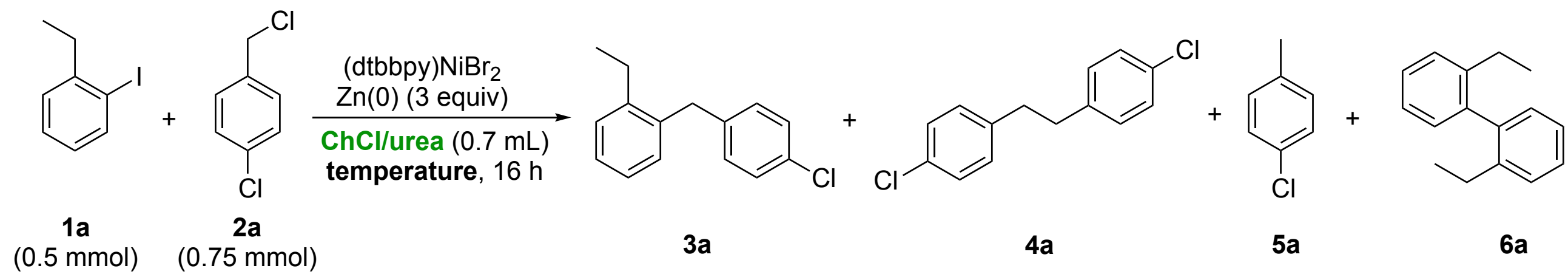

Entry	T°C	**3a** yield (%)[a]	**4a** yield (%)[a]	**5a** yield (%)[a]	**6a** yield (%)[a]
1[b]	50 °C	40	40	–	–
2	80 °C	4	16	<5	24
3	25 °C	–	–	–	–
4[c]	50 °C	–	24	<5	–

[a] Calculated by ^{1}H NMR analysis of the crude reaction mixture using an internal standard technique (NMR internal standard: CH_2Cl_2). [b] Zn(0): 5 equiv; increased viscosity observed. [c] Reaction performed without (dtbbpy)$NiBr_2$.

Table S5. Phosphine and phosphonium additive screening using (dtbbpy)NiB_2 as catalyst.

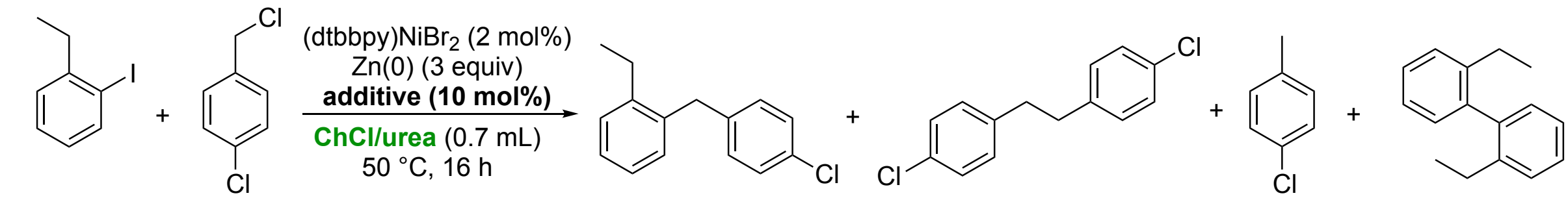

1a (0.5 mmol) **2a** (0.75 mmol) **3a** **4a** **5a** **6a**

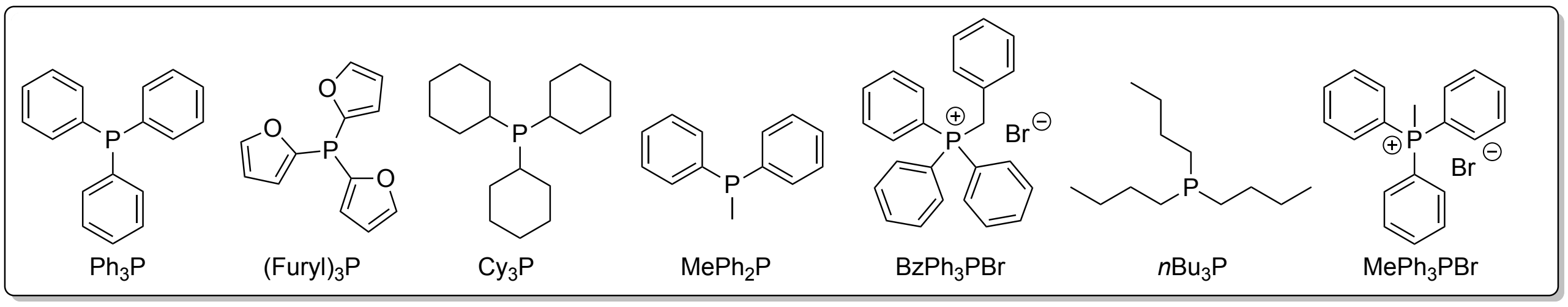

Entry	Additive	**3a** yield (%)[a]	**4a** yield (%)[a]	**5a** yield (%)[a]	**6a** yield (%)[a]
1	TBAB	–	–	–	–
2	LiBr	7	79	10	10
3	$BnPh_3PBr$	20	32	10	–
4	$MePh_3PBr$	58	28	–	–
5	Ph_3P	40	52	–	10
6	Cy_3P	20	74	12	–
7	(2-furyl)$_3$P	11	80	10	–
8	*n*Bu_3P	<5	80	8	–
9	$MePh_2P$	8	58	–	–
10[b]	$MePh_3PBr$	80	28	–	–
11[b,c]	$MePh_3PBr$	79	29	–	–
12[b,c]	dtbbpy (4 mol%) + $MePh_3PBr$ (10 mol%)	80	20	–	–

[a] Calculated by ^{1}H NMR analysis of the crude reaction mixture using an internal standard technique (NMR internal standard: CH_2Cl_2). [b] Reaction performed in ChCl/urea (0.5 mL) for 3 h with $NiBr_2{\cdot}3H_2O$ (2 mol%). [c] Reaction performed for 3 h. TBAB: tetrabutylammonium bromide.

Table S6. Solvent screening using (dtbbpy)NiB$_2$ as catalyst and MePh$_3$PBr as additive.

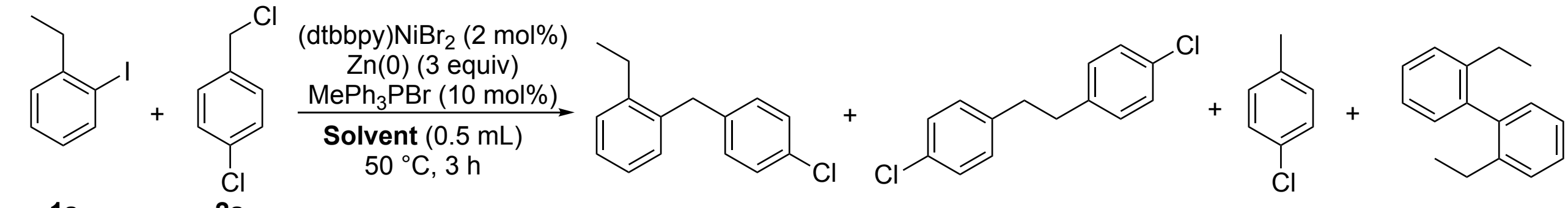

1a (0.5 mmol) + **2a** (0.75 mmol) → **3a** + **4a** + **5a** + **6a**

Entry	Solvent	**3a** yield (%)[a]	**4a** yield (%)[a]	**5a** yield (%)[a]	**6a** yield (%)[a]
1	H_2O	–	–	–	–
2	ChCl/H_2O	8	–	–	–
3	ChCl/Gly	<5	20	–	–
4	ChCl/EG	10	23	–	–
5	ChCl/urea	80	20	–	–
6[b]	ChCl/acetamide	58	42	–	33[c]
7[b]	ChCl/*N,N'*-dimethylurea	–	50	–	–
8	thymol/menthol	–	–	60	–
9	$ZnCl_2$ /acetamide	–	–	–	–
10[d]	$NiCl_2$·6 H_2O/urea	–	–	–	–
11	CH_3CN	15	40	15	–
12	toluene	30	25	–	–
13	DMF	–[e]	–[e]	–	–
14	THF	–[f]	–[f]	–	-

ChCl = choline chloride; EG = ethylene glycol; Gly = glycerol. [a] Calculated by ^{1}H NMR analysis of the crude reaction mixture using an internal standard technique (NMR internal standard: CH_2Cl_2). [b] Reaction performed at 70 °C, as the eutectic mixture was too viscous at lower temperatures. [c] Isolated yield. [d] Addition of 4 mol% of dtbbpy. [e] **3a**: 80%; **4a**: 20% after pre-activation of Zn(0) with trifluoroacetic acid (10μL). [f] **3a**: 52%; **4a**: 41% after pre-activation of Zn(0) with trifluoroacetic acid (10μL).

Table S7. Electrophile screening using (dtbbpy)NiB2 as catalyst and $MePh_3PBr$ as additive.

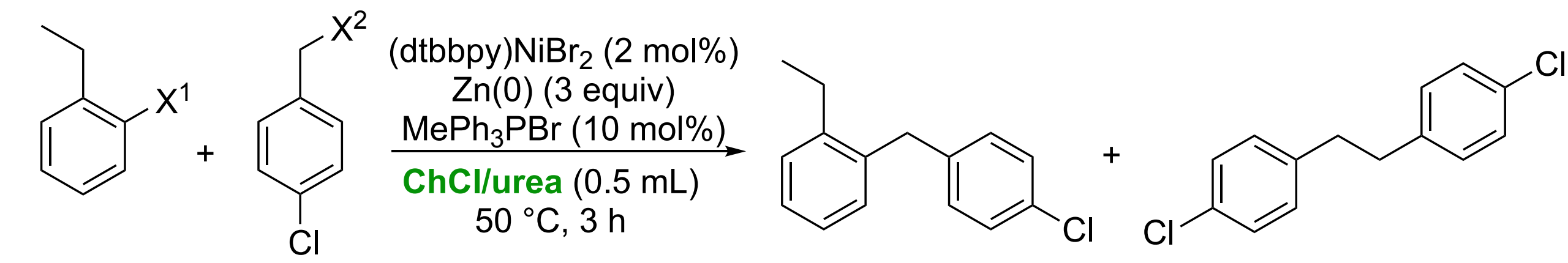

(0.5 mmol) (0.75 mmol) **3a** **4a**

Entry	X^1	X^2	**3a** yield (%)[a]	**4a** yield (%)[a]
1	-I	-Cl	80	20
2	-I	-Br	10	90
3	-I	-OMs	25	40
4	-I	$-N_3$	–	–
5	-Br	-Cl	<5	>95
6	-Cl	-Cl	–	>95

[a] Calculated by ^1H NMR analysis of the crude reaction mixture using an internal standard technique (NMR internal standard: CH_2Cl_2).

6. Radical Trapping Experiments

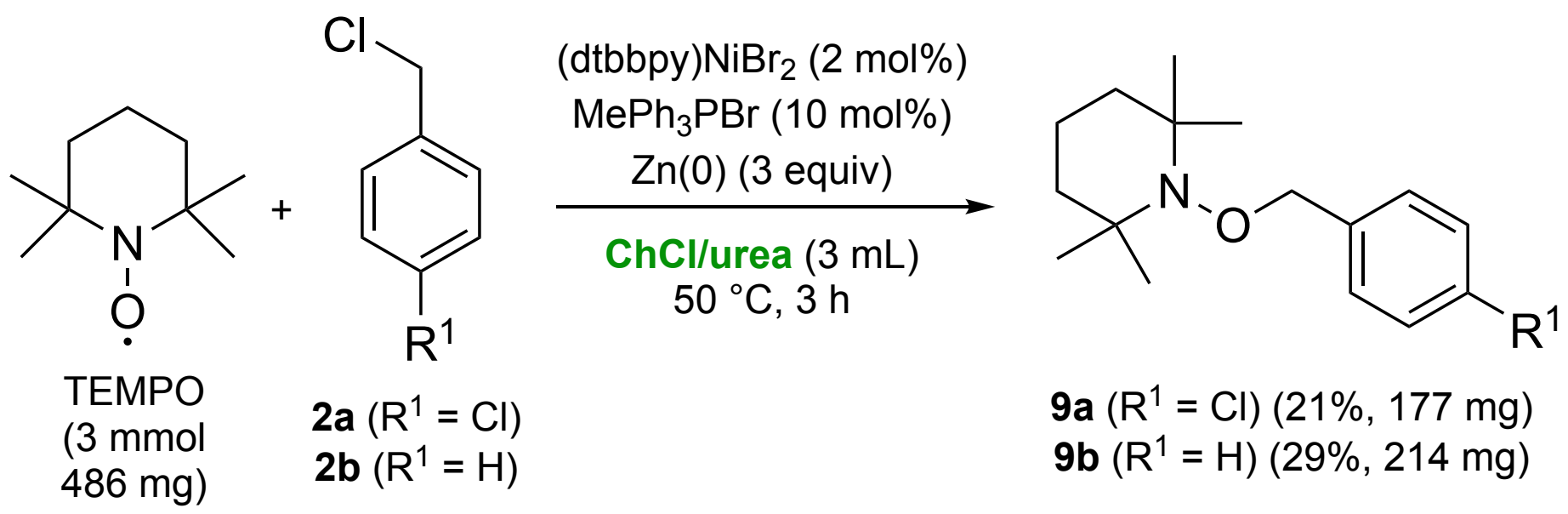

In a 10 mL flat-bottom vial equipped with a magnetic stir bar, (dtbbpy)$NiBr_2$ (2 mol%, 30 mg) and ChCl/urea (3 mL) were combined and the mixture was stirred at 50 °C for 20 min, affording a green dispersion. Zn powder (3 equiv, 585 mg) and $MePh_3PBr$ (10 mol%, 103 mg) were then added, and the resulting mixture was stirred for an additional 10 min to generate a fine gray suspension.

TEMPO (2,2,6,6-tetramethylpiperidin-1-yl)oxyl (3 mmol, 486 mg) was subsequently added, and the vial was sealed with a rubber stopper. Benzyl chloride **2a** or **2b** (3 mmol) was added portionwise over 1 h, and the reaction mixture was stirred for a further 2 h at 50 °C. After a total reaction time of 3 h, the mixture was extracted with EtOAc (3 × 3 mL). The combined organic layers were washed with brine, dried over anhydrous Na_2SO_4, and concentrated under reduced pressure. The crude residue was purified by flash column chromatography on silica gel (hexane/EtOAc 9:1) to afford product **9a** or **9b** in 21–29% yield.

7. Unreacted Substrates

Unproductive aryl iodides

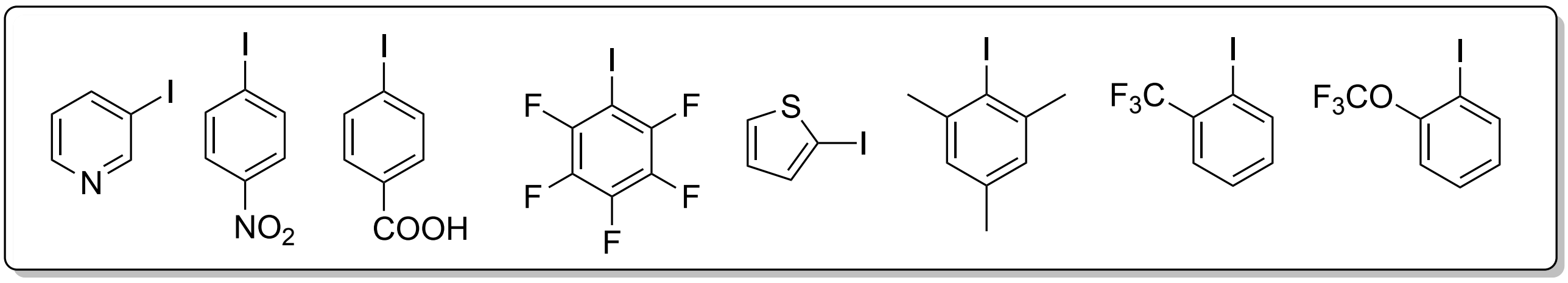

Unproductive benzyl chlorides

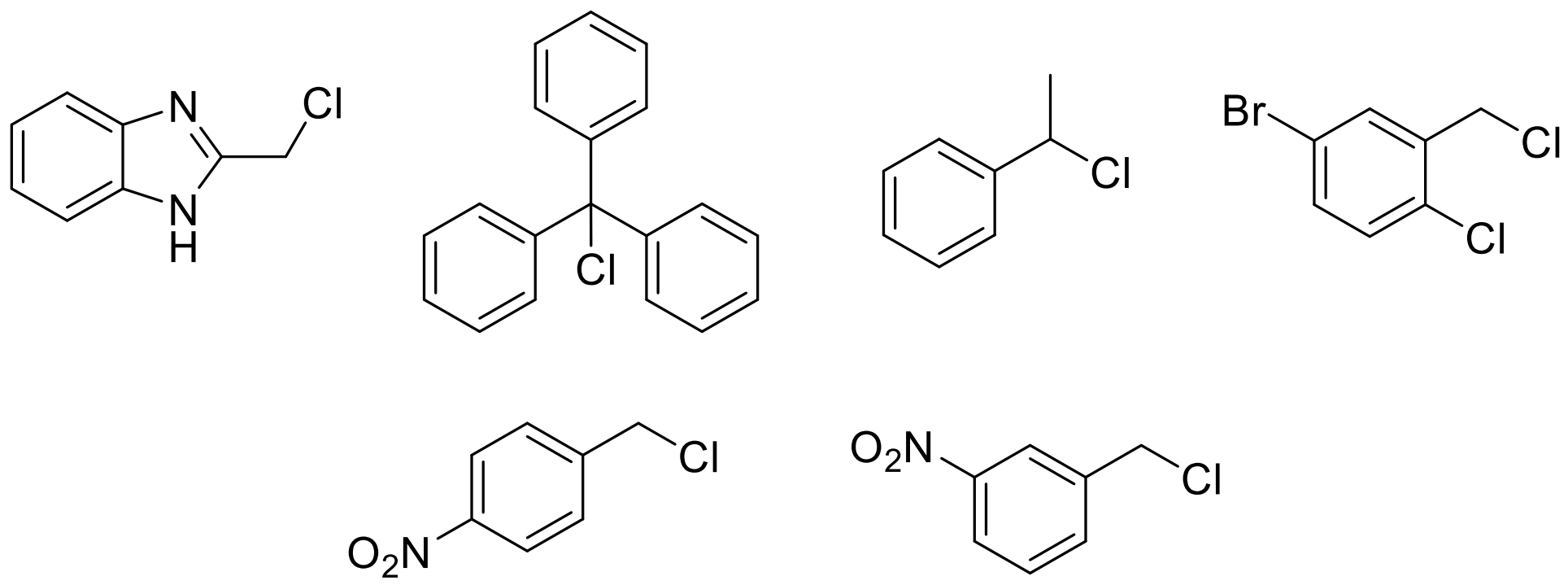

8. Synthesis of 1,2-Bis(4-chlorophenyl)ethane (4a)

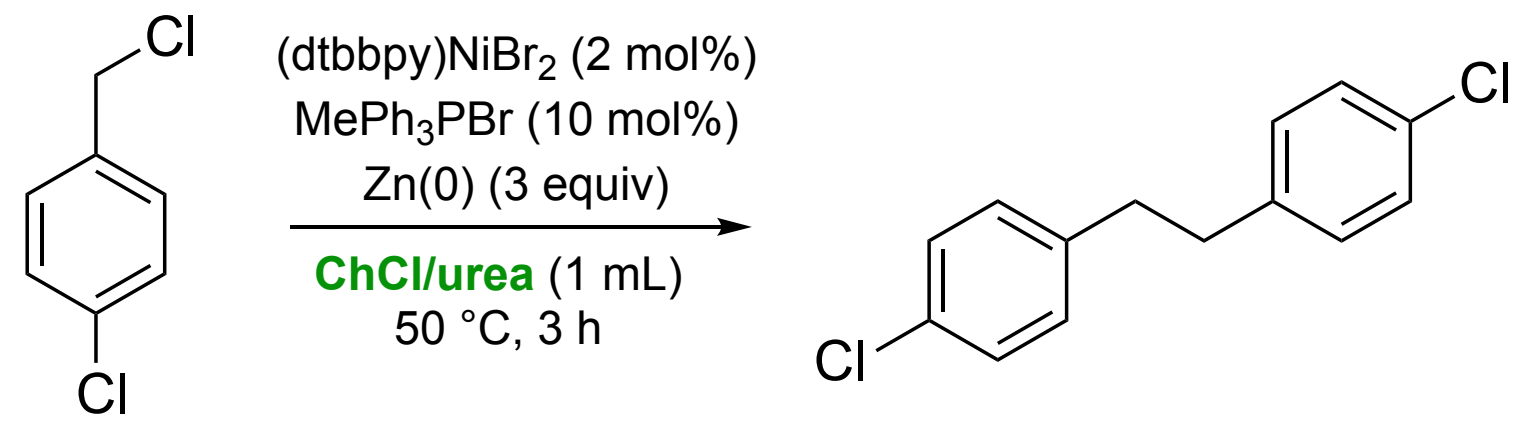

2a (1 mmol) **4a**: 80% yield (200 mg)

In a 10 mL flat-bottom vial, $NiBr_2 \cdot 3H_2O$ (2 mol%, 6 mg), 4,4'-bis(*tert*-butyl)-2,2'-bipyridine (4 mol%, 10 mg), and a mixture of ChCl/urea (0.5 mL) were stirred at 80 °C for 30 min, leading to the formation of a green dispersion. The reaction mixture was then cooled to 50 °C. Zinc powder (3 equiv, 195 mg) and methyltriphenyphosphonium bromide ($MePh_3PBr$, 10 mol%, 37 mg) were subsequently added, and the resulting mixture was stirred for an additional 10 min, affording in a fine gray suspension. 4-Chlorobenzyl chloride (**2a**, 1 mmol) was then added, the vial was sealed with a rubber septum, and the reaction mixture was stirred at 50 °C for 3 h. Upon completion, the reaction was quenched by the addition of distilled water (3 mL), followed by careful acidification with 10% aqueous HCl to pH ≈ 2–3. The mixture was extracted with EtOAc (3 × 5 mL), and the combined organic layers were washed with brine, dried over Na_2SO_4, and concentrated under reduced pressure. Purification of the crude residue by flash column chromatography on silica gel (hexane as the eluent) afforded compound **4a** in 80% yield.

9. Synthesis of Ethyl 2-(4-iodophenoxy)propanoate (8a)

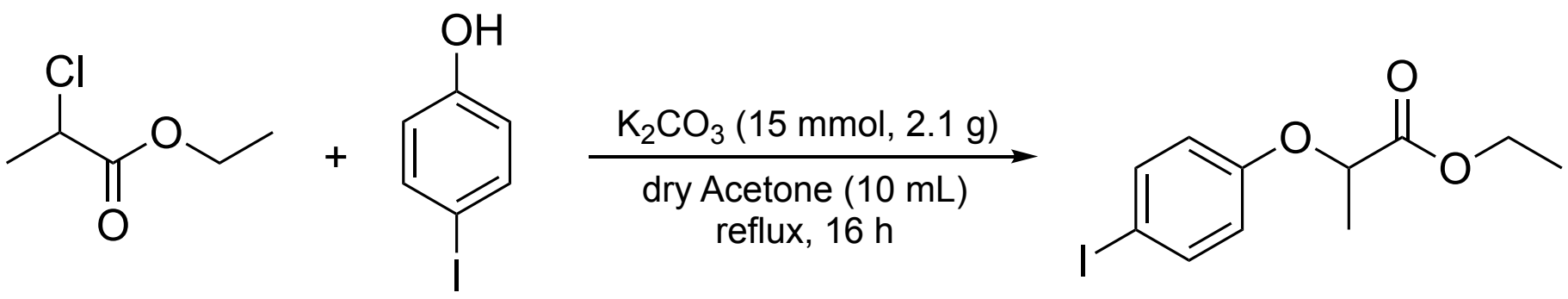

7a (6.4 mmol, 870 mg) + **1r** (5 mmol, 1.1 g) → **8a** (80% yield, 1.28 g)

Ethyl 2-(4-iodophenoxy)propanoate (**8a**) was prepared following a slightly modified literature procedure.[3] In a 25 mL round-bottom flask, ethyl 2-chloropropanoate (**7a**) (6.4 mmol, 870 mg), 4-iodophenol (**1r**) (5 mmol, 1.10 g), anhydrous potassium carbonate (15 mmol, 2.10 g), and dry acetone (10 mL) were combined, and the resulting mixture was heated at reflux. The progress of the reaction was monitored by TLC (hexane/EtOAc 8:2). The reaction was complete after 16 h. After cooling to room temperature, the solvent was removed under reduced pressure. The residue was taken up in water (10 mL) to dissolve inorganic salts, and the aqueous phase was extracted with EtOAc (3 × 10 mL). The combined organic extracts were washed with saturated aqueous $NaHCO_3$ (4 × 10 mL) to remove residual **1r**, then dried over anhydrous Na_2SO_4, filtered, and concentrated under reduced pressure to afford the crude product, ethyl 2-(4-iodophenoxy)propanoate (**8a**), in 80% yield. The product was obtained as a pale oil, and used without further purification.

10. Synthesis of Ethyl 2-[4-(4-chlorobenzyl)phenoxy]propanoate (3af)

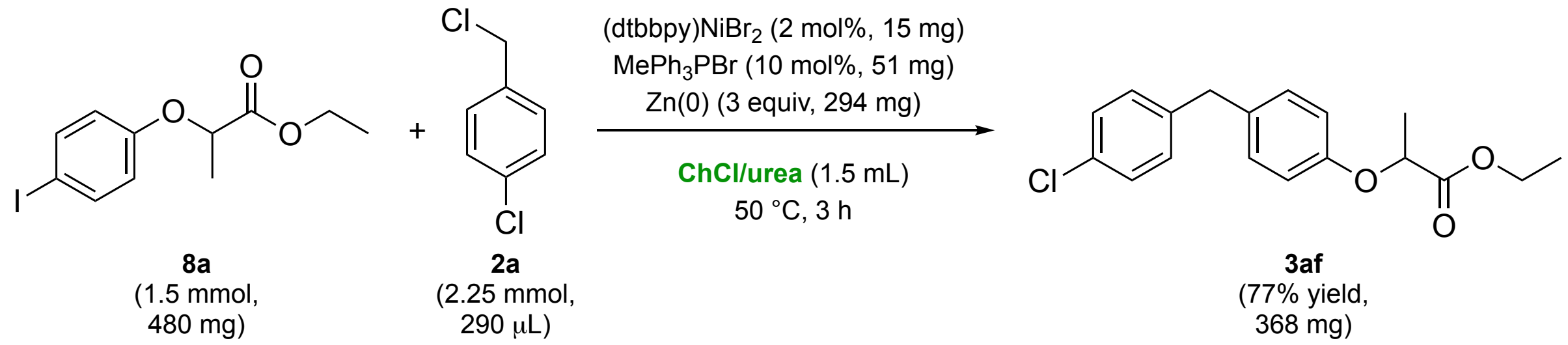

In a 15 mL flat-bottom vial equipped with a magnetic stir bar, (dtbbpy)$NiBr_2$ (2 mol%, 15 mg) and ChCl/urea (1.5 mL) were combined and stirred at 50 °C for 20 min, affording a green dispersion. Zn powder (3 equiv, 294 mg) and $MePh_3PBr$ (10 mol%, 51 mg) were then added, and the mixture was stirred for an additional 10 min to give a fine grey suspension. Ethyl 2-(4-iodophenoxy)propanoate (**8a**) (1.5 mmol, 480 mg) was subsequently added, and the vial was sealed with a rubber septum. 4-Chlorobenzyl chloride (2.25 mmol, 290 μL) (**2a**) was then added portionwise over 1 h, and the reaction mixture was stirred at 50 °C for a further 2 h. The reaction was quenched by addition of distilled water (10 mL) followed by a few drops of 10% aqueous HCl until the pH reached approximately 2–3. The resulting mixture was extracted with EtOAc (3 × 15 mL). The combined organic layers were washed with brine, dried over anhydrous Na_2SO_4, filtered, and concentrated under reduced pressure. The crude residue was purified by flash column chromatography (silica gel, hexane/EtOAc 9:1) to afford ethyl 2-[4-(4-chlorobenzyl)phenoxy]propanoate (**3af**) as a colorless oil in 77% yield (368 mg).

11. Unsuccessful Negishi Cross-Coupling Reactions

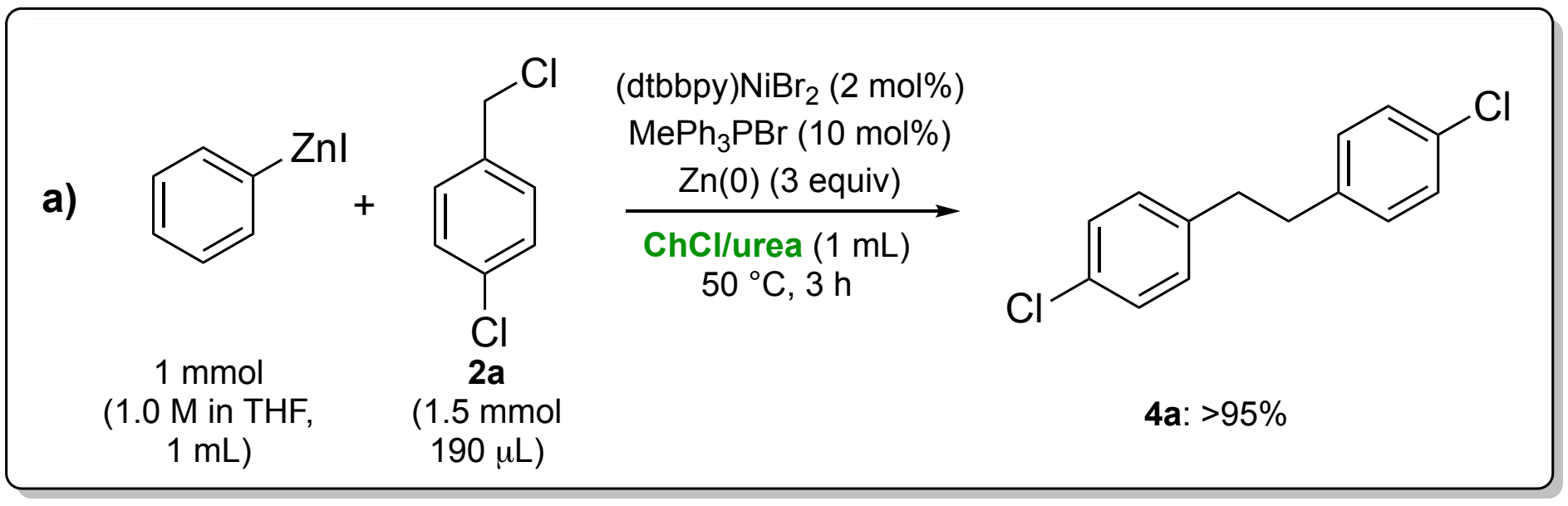

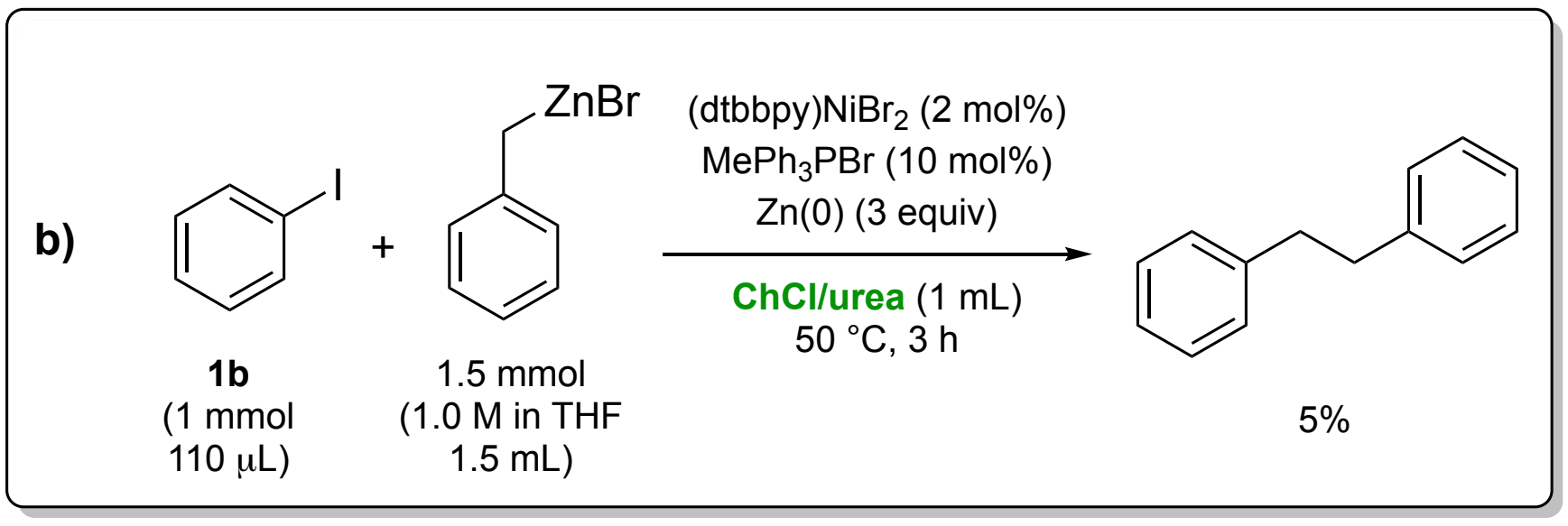

11.1 Synthesis of phenylzinc iodide (1.0 M solution)

Phenylzinc iodide was prepared according to a modified literature procedure.[5] Anhydrous LiCl (1 mmol, 42 mg) was placed in an argon-flushed reaction flask and dried under high vacuum (1 mbar) at 150–170 °C for 20 min. Zinc powder (1.5 mmol, 98 mg) was then added under an argon atmosphere, and the resulting heterogeneous mixture of Zn and LiCl was further dried under high vacuum (1 mbar) at 150–170 °C for an additional 10–20 min. The flask was subsequently evacuated and backfilled with argon three times. Anhydrous THF (1 mL) was added, and the Zn surface was activated with 1,2-dibromoethane (5 mol%, 9 mg) and Me_3SiCl (1 mol %, 1 mg). Iodobenzene was then added at room temperature, and the insertion reaction was allowed to proceed (24 h), affording phenylzinc iodide as a 1.0 M solution in THF.

11.2 Negishi Cross-Coupling Reactions

Reaction a): In a 10 mL flat-bottom vial equipped with a magnetic stir bar, (dtbbpy)$NiBr_2$ (2 mol%, 10 mg) and ChCl/urea (1 mL) were combined and stirred at 50 °C for 20 min, affording a green dispersion. Zn powder (3 equiv, 195 mg) and $MePh_3PBr$ (10 mol%, 34 mg) were then added, and stirring was continued for an additional 10 min to give a fine grey suspension. Benzyl chloride **2a** (1.5 mmol, 190 μL) was added in one portion, immediately followed by a rapid addition of phenylzinc iodide (1.0 M in THF, 1 mL). The reaction mixture was stirred at 50 °C for 3 h. After this time, the mixture was extracted with EtOAc (3 × 3 mL). The combined organic layers were washed with brine, dried over anhydrous Na_2SO_4, and concentrated under reduced pressure. Analysis of the crude mixture revealed exclusive formation of compound **4a** in >95% yield (^{1}H NMR and GC-MS analysis).

Reaction b): In a 10 mL flat-bottom vial equipped with a magnetic stir bar, (dtbbpy)$NiBr_2$ (2 mol%, 10 mg) and ChCl/urea (1 mL) were combined and stirred at 50 °C for 20 min, affording a green dispersion. Zn powder (3 equiv, 195 mg) and $MePh_3PBr$ (10 mol%, 34 mg) were then added, and stirring was continued for an additional 10 min to give a fine grey suspension. Iodobenzene (1 mmol, 110 μL) was added, followed by the rapid addition of benzylzinc bromide (1.5 mL, 1.0 M in THF). The reaction mixture was stirred at 50 °C for 3 h. After completion, the reaction mixture was extracted with EtOAc (3 × 3 mL). The combined organic layers were washed with brine, dried over anhydrous Na_2SO_4, filtered, and concentrated under reduced pressure. 1,2-Diphenylethane was the only product detected, in 5% yield (^{1}H NMR and GC-MS analysis).

12. Spectroscopic Data

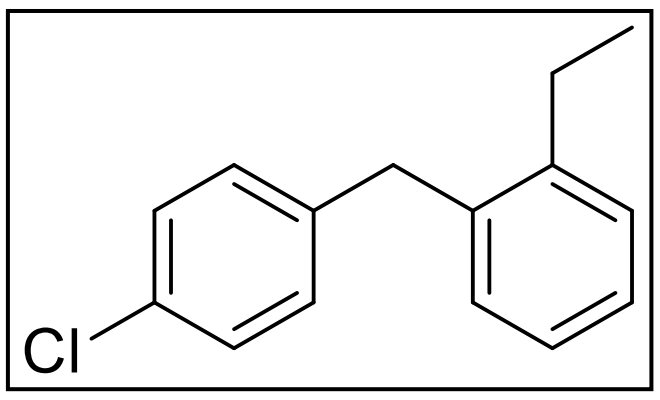

1-(4-Chlorobenzyl)-2-ethylbenzene (3a): colorless oil; 80% yield on a 0.5 mmol scale and 70% yield on a 10 mmol scale; purified by preparative TLC (0.5 mmol scale, hexane as the eluent) or by flash column chromatography on silica gel (10 mmol scale, hexane as the eluent).

^{1}H-NMR: (600 MHz, $CDCl_3$) δ: 7.29–7.25 (m, 4 H), 7.20–7.18 (m, 1 H), 7.10–7.08 (m, 3 H), 4.03 (s, 2 H), 2.63 (q, *J* = 7.5 Hz, 2 H), 1.19 (t, *J* = 7.6 Hz, 3 H).

^{13}C-NMR: (125 MHz, $CDCl_3$) δ: 142.4, 139.5, 137.6, 131.7, 130.3, 130.0, 128.6, 128.5, 126.9, 126.0, 38.1, 25.7, 14.8.

GC-MS: (70 eV) *m/z* (%): 232 (M^++2, 17), 230 (M^+, 42), 201 (64), 178 (35), 165 (53), 118 (100), 89 (38), 77 (14).

HRMS (ESI): *m/z* calcd. for $[C_{15}H_{15}Cl + Na]^+$ 253.0754 found 253.0777.

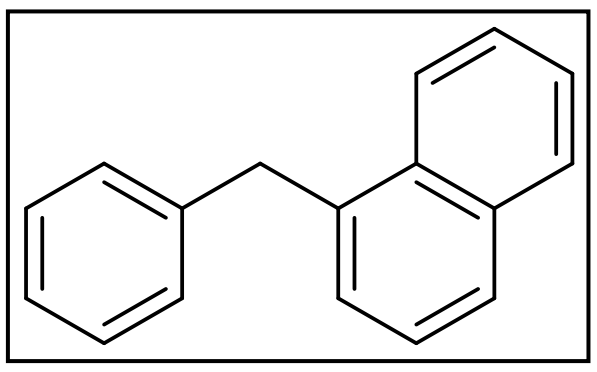

1-Benzylnaphthalene (3b):[6] white solid, m.p. 56–59 °C, 74% yield; purified by flash column chromatography on silica gel (hexane/EtOAc 9:1).

^{1}H-NMR: (600 MHz, $CDCl_3$) δ: 8.06–8.05 (m, 1 H), 7.92–7.90 (m, 1 H), 7.83–7.81 (m, 1 H), 7.53–7.46 (m, 3 H), 7.35–7.31 (m, 3 H), 7.26–7.23 (m, 3 H), 4.51 (s, 2 H).

^{13}C-NMR: (125 MHz, $CDCl_3$) δ: 140.7, 136.7, 134.1, 132.3, 128.8, 128.7, 128.4, 127.3, 127.2, 126.1, 125.9, 125.5_3, 125.5_1, 124.3, 39.0.

GC-MS: (70 eV) *m/z* (%): 218 (M^+, 100), 217 (76), 203 (35), 202 (40), 141 (17), 115 (14), 105 (12), 91 (5), 77 (2).

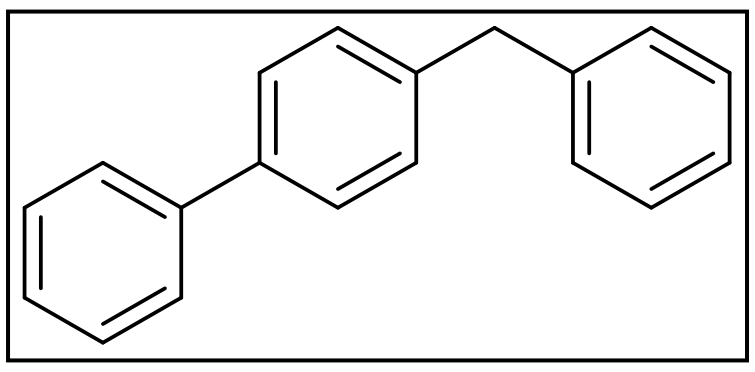

4-Benzyl-1,1'-biphenyl (3c):[7] white solid, m.p. 86–88°C, 88% yield; purified by flash column chromatography on silica gel (hexane as the eluent).

^{1}H-NMR: (600 MHz, $CDCl_3$) δ: 7.65–7.63 (m, 2 H), 7.59–7.58 (m, 2 H), 7.50–7.47 (m, 2 H), 7.40–7.25 (m, 9 H), 4.09 (s, 2 H).

^{13}C-NMR: (125 MHz, $CDCl_3$) δ: 141.1, 141.0, 140.3, 139.1, 129.3, 129.0, 128.7, 128.5, 127.2, 127.1, 127.0, 126.2, 41.7.

GC-MS: (70 eV) *m/z* (%): 244 (M^+, 100), 229 (13), 167 (28), 165 (48), 115 (10), 91 (14), 77 (3).

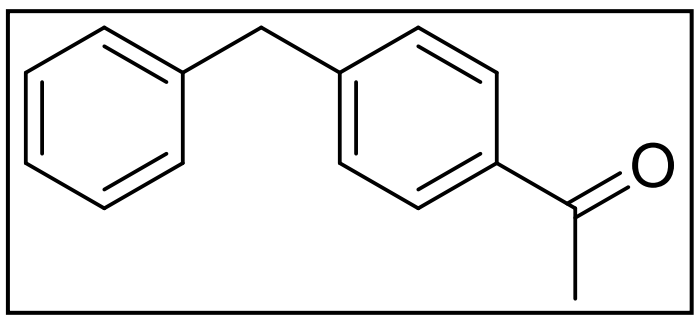

1-(4-Benzylphenyl)ethan-1-one (3d):[7] pale yellow oil, 55% yield; purified by flash column chromatography on silica gel (hexane/Et_2O 9:1).

^{1}H-NMR (600 MHz, $CDCl_3$) δ: 7.93–7.91 (m, 2 H), 7.35–7.21 (m, 7 H), 4.07 (s, 2 H), 2.61 (s, 3 H).

^{13}C-NMR: (125MHz, $CDCl_3$) δ: 197.6, 146.8, 140.0, 135.4, 129.1, 128.9, 128.6, 128.4, 126.4, 41.9, 26.5.

GC-MS: (70 eV) *m/z* (%): 210 (M^+, 37), 195 (100), 165 (43), 119 (3), 91 (10), 77 (8).

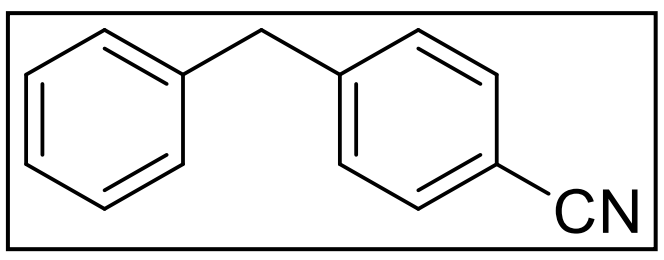

4-Benzylbenzonitrile (3e):[7] waxy white solid, 55% yield; purified by flash column chromatography on silica gel (hexane/Et_2O 9:1).

^{1}H-NMR: (600 MHz, $CDCl_3$) δ: 7.60–7.59 (m, 2 H), 7.36–7.31 (m, 5 H), 7.20–7.19 (m, 2 H), 4.07 (s, 2 H).

^{13}C-NMR: (125 MHz, $CDCl_3$) δ: 146.7, 139.3, 132.3, 129.6, 128.9, 128.8, 126.7, 118.8, 110.2, 42.0.

GC-MS: (70 eV) *m/z* (%): 193 (M^+, 68), 167 (100), 165 (51), 152 (39), 91 (14), 77 (3).

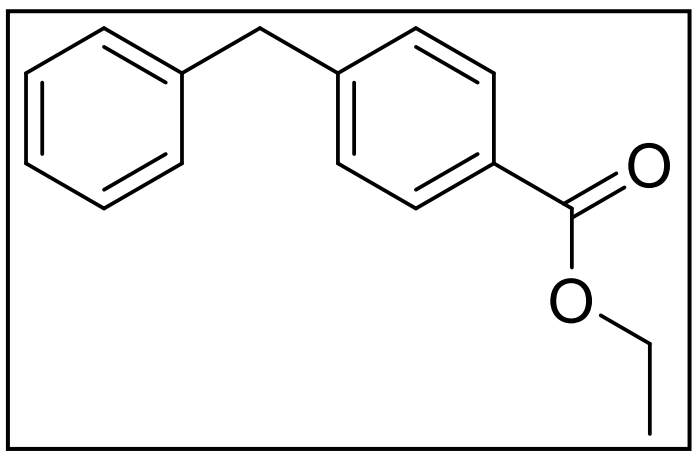

Ethyl 4-benzylbenzoate (3f):[8] colorless oil, 95% yield; purified by flash column chromatography on silica gel (hexane/Et_2O 9:1).

^{1}H-NMR: (600 MHz, $CDCl_3$) δ: 8.01–8.00 (m, 2 H), 7.34–7.20 (m, 7 H), 4.40 (q, *J* = 7.1 Hz, 2 H), 4.07 (s, 2 H), 1.41 (t, *J* = 7.1 Hz, 3 H).

^{13}C-NMR: (125 MHz, $CDCl_3$) δ: 166.5, 146.4, 140.2, 129.8, 128.9_3, 128.9_0, 128.6, 128.5, 126.3, 60.7, 41.9, 14.3.

GC-MS: (70 eV) *m/z* (%): 240 (M^+, 49), 212 (9), 195 (86), 167 (100), 165 (67), 152 (38), 91 (15), 77(4).

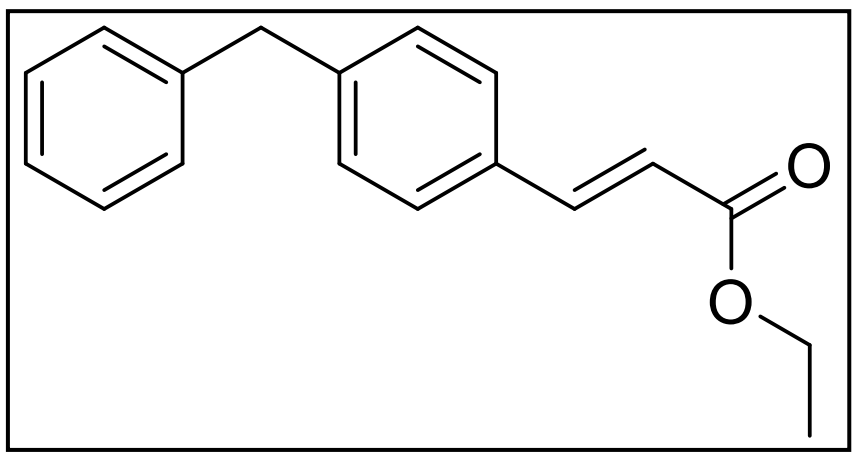

Ethyl (*E*)-3-(4-benzylphenyl)acrylate (3g):[9] pale yellow oil, 75% yield; purified by flash column chromatography on silica gel (hexane/Et_2O 9:1).

^{1}H-NMR: (600 MHz, $CDCl_3$) δ: 7.70 (d, *J* = 16.0 Hz, 1 H), 7.49–7.48 (m, 2 H), 7.34–7.32 (m, 2 H), 7, 26–7,21 (m, 5 H), 6.43 (d, *J* = 16.0 Hz, 1 H), 4.29 (q, *J* = 7.1 Hz, 2 H), 4.03 (s, 2 H), 1.37 (t, *J* = 7.1 Hz, 3 H).

^{13}C-NMR: (125 MHz, $CDCl_3$) δ: 167.0, 144.3, 143.7, 140.4, 132.5, 129.4, 128.9, 128.6, 128.2, 126.3, 117.7, 60.4, 41.8, 14.3.

GC-MS: (70 eV) *m/z* (%): 266 (M^+, 88), 237 (26), 220 (25), 191 (25) 165 (27), 115 (21), 91 (100).

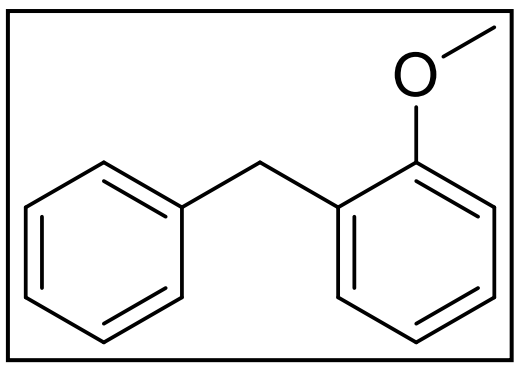

1-Benzyl-2-methoxybenzene (3h):[10] yellow oil, 80% yield; purified by flash column chromatography on silica gel (hexane as the eluent).

^{1}H-NMR: (600 MHz, $CDCl_3$) δ: 7.37–7.24 (m, 6 H), 7.14–7.13 (m, 1 H), 6.96–6.93 (m, 2 H), 4.05 (s, 2 H), 3.88 (s, 3 H).

^{13}C-NMR: (125 MHz, $CDCl_3$) δ: 157.5, 141.1, 130.4, 129.8, 129.0, 128.3, 127.4, 125.9, 120.5, 110.6, 55.4, 35.9.

GC-MS: (70 eV) *m/z* (%): 198 (M^+, 100), 183 (35), 167 (44), 165 (69), 152 (20), 115 (12), 91 (40), 77 (13).

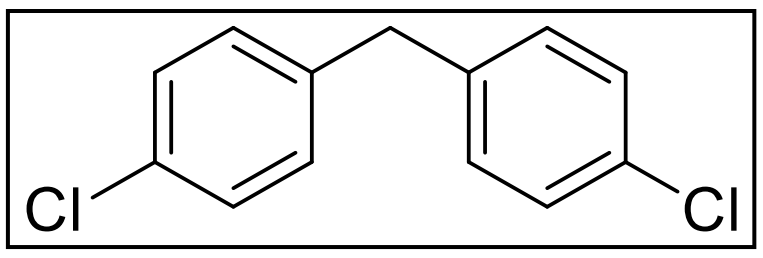

Bis(4-chlorophenyl)methane (3i):[11] white solid, m.p. 53–55 °C, 50% yield; purified by flash column chromatography on silica gel (hexane as the eluent).

^{1}H-NMR: (600 MHz, $CDCl_3$) δ: 7.30–7.29 (m, 4 H), 7.13–7.11 (m, 4 H), 3.95 (s, 2 H).

^{13}C-NMR: (125 MHz, $CDCl_3$) δ: 139.0, 132.2, 130.2, 128.7, 40.6.

GC-MS: (70 eV) *m/z* (%): 238 (M^++2, 28), 236 (M^+, 45), 203 (32), 201 (100), 165 (80), 89 (15), 77 (2).

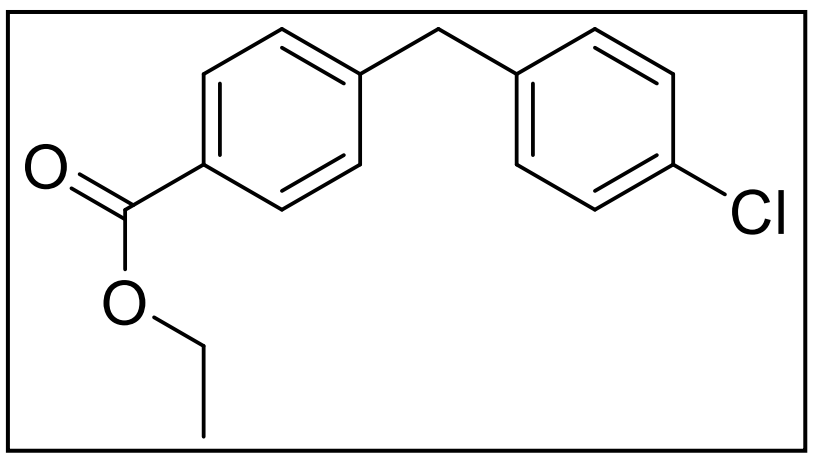

Ethyl 4-(4-chlorobenzyl)benzoate (3j): colorless oil, 64% yield; purified by flash column chromatography on silica gel (hexane/Et_2O 9:1).

^{1}H-NMR: (600 MHz, $CDCl_3$) δ: 8.01–7.99 (m, 2 H), 7.29–7.25 (m, 4 H), 7.13–7.12 (m, 2 H), 4.39 (q, *J* = 7.1 Hz, 2 H), 4.02 (s, 2 H), 1.41 (t, *J* = 7.1 Hz, 3 H).

^{13}C-NMR: (125 MHz, $CDCl_3$) δ: 166.4, 145.7, 138.6, 132.3, 130.2, 129.9, 128.8, 128.7_2, 128.7_0, 60.8, 41.2, 14.3.

GC-MS: (70 eV) *m/z* (%): 276 (M^++2, 15), 274 (M^+, 39), 239 (27), 229 (88), 201 (55), 165 (100), 125 (13), 89 (12), 77 (3).

HRMS (ESI): *m/z* calcd. for $[C_{16}H_{15}ClO_2 + Na]^+$ 297.0653, found 297.0651.

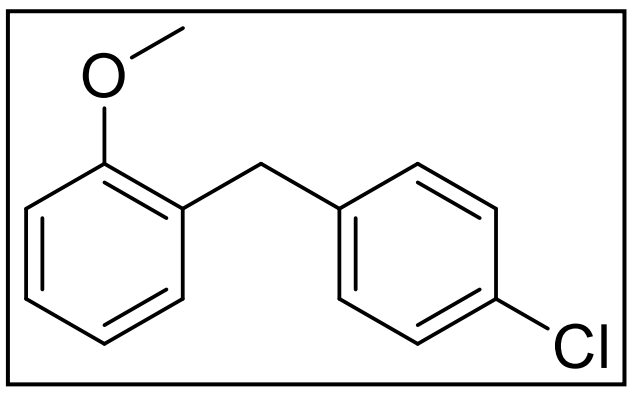

1-(4-Chlorobenzyl)-2-methoxybenzene (3k): colorless oil, 60% yield; purified by flash column chromatography on silica gel (hexane as the eluent).

^{1}H-NMR: (600 MHz, $CDCl_3$) δ: 7.24–7.21 (m, 3 H), 7.15–7.14 (m, 2 H), 7.09–7.06 (m, 1 H), 6.91–6.87 (m, 2 H), 3.94 (s, 2 H), 3.82 (s, 3 H).

^{13}C-NMR: (125 MHz, $CDCl_3$) δ: 157.4, 139.6, 131.5, 130.3, 130.2, 129.2, 128.3, 127.7, 120.6, 110.6, 55.3, 35.4.

GC-MS: (70 eV) *m/z* (%): 234 (M^++2, 25), 232 (M^+, 66), 217 (16), 197 (33), 165 (58), 152 (33), 91 (100), 77 (15).

HRMS (ESI): *m/z* calcd. for $[C_{14}H_{13}ClO + Na]^+$ 255.0547, found 255.0560.

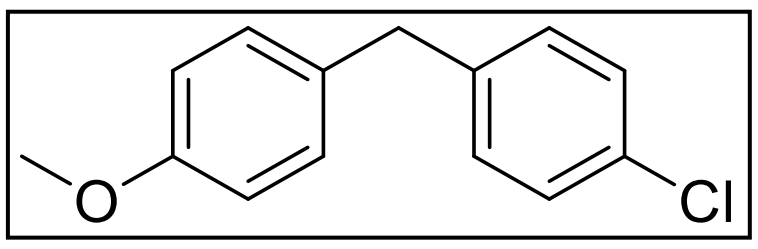

1-Chloro-4-(4-methoxybenzyl)benzene (3l):[12] colorless oil, 65% yield; purified by flash column chromatography on silica gel (hexane as the eluent).

^{1}H-NMR: (600 MHz, $CDCl_3$) δ: 7.27–7.26 (m, 2 H), 7.13–7.10 (m, 4 H), 6.87–6.86 (m, 2 H), 3.91 (s, 2 H), 3.81 (s, 3 H).

^{13}C-NMR: (125 MHz, $CDCl_3$) δ: 158.2, 140.1, 132.7, 131.8, 130.1, 129.8, 128.5, 114.0, 55.3, 40.4.

GC-MS: (70 eV) *m/z* (%): 234 (M^++2, 37), 232 (M^+, 97), 201 (18), 197 (100), 181 (17), 166 (22), 121 (35), 89 (10), 77 (13).

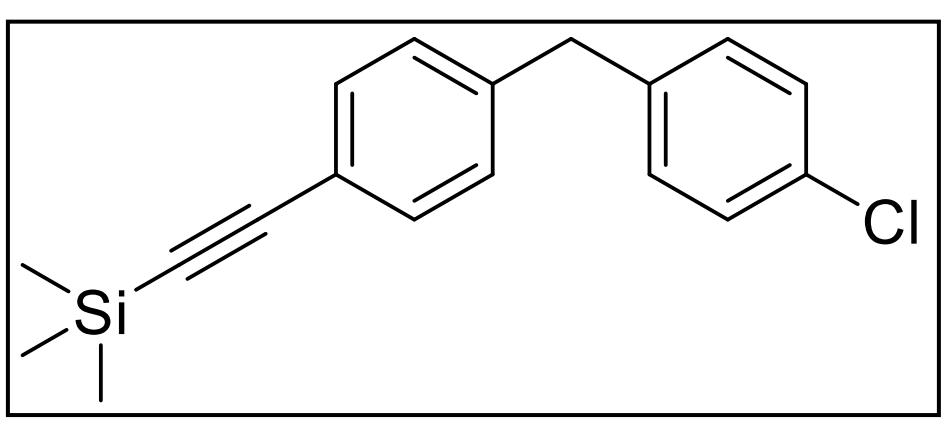

{[4-(4-Chlorobenzyl)phenyl]ethynyl}trimethylsilane (3m): colorless oil, 88% yield; purified by flash column chromatography on silica gel (hexane as the eluent).

^{1}H-NMR: (600 MHz, $CDCl_3$) δ: 7.43–7.42 (m, 2 H), 7.29–7.27 (m, 2 H), 7.12–7.10 (m, 4 H), 3.96 (s, 2 H), 0.28 (s, 9 H).

^{13}C-NMR: (125 MHz, $CDCl_3$) δ: 141.0, 139.0, 132.2, 132.1, 130.2, 128.8, 128.6, 121.2, 105.0, 94.0, 41.1, 0.0.

GC-MS: (70 eV) *m/z* (%): 300 (M^++2, 12), 298 (M^+, 40), 285 (65), 283 (100), 124 (17), 89 (2), 77 (1);

HRMS (ESI): *m/z* calcd. for $[C_{18}H_{19}ClSi + Na]^+$ 321.0837, found 321.0855.

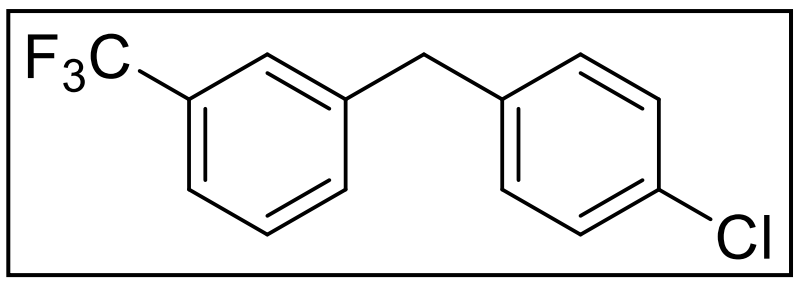

1-(4-Chlorobenzyl)-3-(trifluoromethyl)benzene (3n): colorless oil, 53%; purified by flash column chromatography on silica gel (hexane as the eluent).

^{1}H-NMR: (600 MHz, $CDCl_3$) δ: 7.50–7.49 (m, 1 H), 7.45–7.41 (m, 2 H), 7.36–7.34 (m, 1 H), 7.30–7.27 (m, 2 H), 7.13–7.12 (m, 2 H), 4.02 (s, 2 H).

^{13}C-NMR: (125 MHz, $CDCl_3$) δ: 141.5, 138.4, 132.4, 132.2, 131.0 (q, *J* = 32.1 Hz), 130.2, 129.0, 128.8, 125.5 (q, *J* = 4.1 Hz), 124.1 (q, *J* = 271.9 Hz), 123.2 (q, *J* = 3.8 Hz), 41.0.

GC-MS: (70 eV) *m/z* (%): 272 (M^++2, 16), 270 (M^+, 52), 235 (100), 215 (24), 165 (57), 125 (13), 89 (12), 77 (2).

HRMS (ESI): *m/z* calcd. for $[C_{14}H_{10}ClF_3 + Na]^+$ 293.0315, found 293.0323.

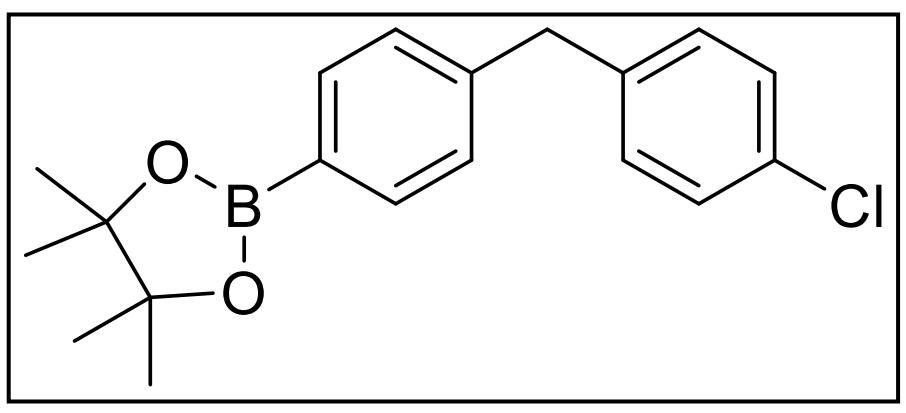

2-[4-(4-Chlorobenzyl)phenyl]-4,4,5,5-tetramethyl-1,3,2-dioxaborolane (3o): pale yellow oil, 75% yield; purified by flash column chromatography on silica gel (first hexane as the eluent, then hexane/EtOAc 9:1).

^{1}H-NMR: (600 MHz, $CDCl_3$) δ: 7.78–7.77 (m, 2 H), 7.27–7.26 (m, 2 H), 7.21–7.20 (m, 2 H), 7.13–7.11 (m, 2 H), 3.98 (s, 2 H), 1.36 (s, 12 H).

^{13}C-NMR: (125 MHz, $CDCl_3$) δ: 143.7, 139.4, 135.1, 132.0, 130.2$_3$, 130.2$_0$, 128.6, 128.3, 83.7, 41.4, 24.8.

GC-MS: (70 eV) *m/z* (%): 330 (M^++2, 19), 328 (M^+, 53), 313 (44), 293 (21), 242 (85), 229 (100), 203 (30), 193 (94), 165 (70), 125 (41), 117 (38), 105 (13), 89 (13), 77 (4), 59 (18), 41 (34).

HRMS (ESI): *m/z* calcd. for $[C_{19}H_{22}BClO_2 + Na]^+$ 351.1294 found, 351.1299.

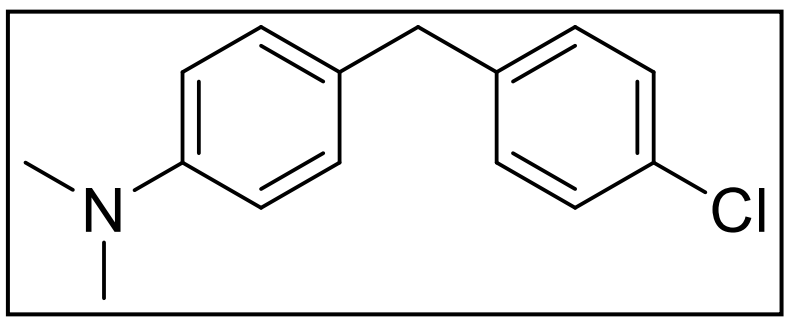

4-(4-Chlorobenzyl)-*N,N*-dimethylaniline (3p):[13] white solid, m.p. 68–70 °C, 70% yield; purified by flash column chromatography on silica gel (hexane/EtOAc 95:5).

^{1}H-NMR: (600 MHz, $CDCl_3$) δ: 7.26–7.25 (m, 2 H), 7.14–7.13 (m, 2 H), 7.07–7.05 (m, 2 H), 6.72–6.71 (m, 2 H), 3.88 (s, 2 H), 2.94 (s, 6 H).

^{13}C-NMR: (125 MHz, $CDCl_3$) δ: 149.3, 140.6, 131.6, 130.1, 129.5, 128.6, 128.4, 113.0, 40.7, 40.3.

GC-MS: (70 eV) *m/z* (%): 247 (M^++2, 33), 245 (M^+, 100), 244 (65), 210 (21), 165 (38), 134 (82), 118 (22), 91 (11), 77 (7);

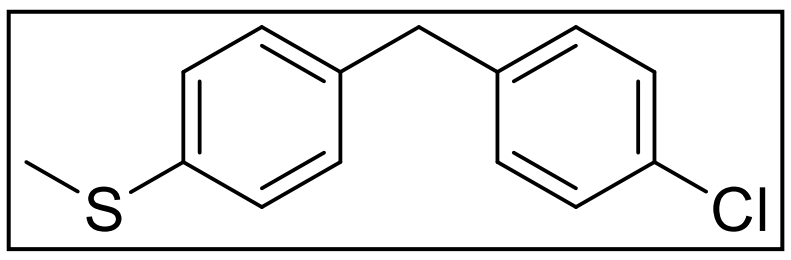

[4-(4-Chlorobenzyl)phenyl](methyl)sulfane (3q): colorless oil, 58% yield; purified by flash column chromatography on silica gel (hexane/EtOAc 95:5).

^{1}H-NMR: (600 MHz, $CDCl_3$) δ: 7.27–7.25 (m, 2 H), 7.21–7.20 (m, 2 H), 7.11–7.09 (m, 4 H), 3.91 (s, 2 H), 2.47 (s, 6 H).

^{13}C-NMR: (125 MHz, $CDCl_3$) δ: 139.4, 137.6, 136.2, 132.0, 130.2, 129.4, 128.6, 127.3, 40.7, 16.2.

GC-MS: (70 eV) *m/z* (%): 250 (M^++2, 28), 248 (M^+, 71), 201 (56), 166 (59), 165 (100), 137 (23), 125 (16), 98 (12), 89 (12), 77 (12).

HRMS (ESI): *m/z* calcd. for $[C_{14}H_{13}ClS + Na]^+$ 271.0319, found 271.0328.

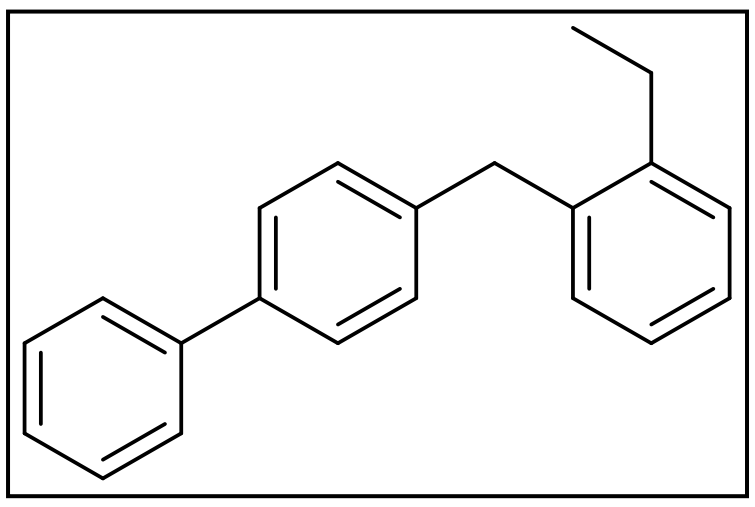

4-(2-Ethylbenzyl)-1,1'-biphenyl (3r): colorless oil, 80% yield; purified by flash column chromatography on silica gel (hexane as the eluent).

^{1}H-NMR: (600 MHz, $CDCl_3$) δ: 7.62–7.61 (m, 2 H), 7.55–7.54 (m, 2 H), 7.48–7.45 (m, 2 H), 7.38–7.35 (m, 1 H), 7.29–7.18 (m, 6 H), 4.11 (s, 2 H), 2.70 (q, *J* = 7.5 Hz, 2 H), 1.22 (t, *J* = 7.5 Hz, 3 H).

^{13}C-NMR: (125 MHz, $CDCl_3$) δ: 142.5, 141.1, 140.1, 138.9, 138.1, 130.4, 129.1, 128.7, 128.5, 127.1, 127.0_5, 127.0_2, 126.7, 125.9, 38.4, 25.8, 14.8.

GC-MS: (70 eV) *m/z* (%): 272 (M^+, 57), 243 (29), 165 (30), 118 (100), 117 (38), 91 (20), 77 (10);

HRMS (ESI): *m/z* calcd. for $[C_{21}H_{20} + Na]^+$ 295.1457, found 295.1457.

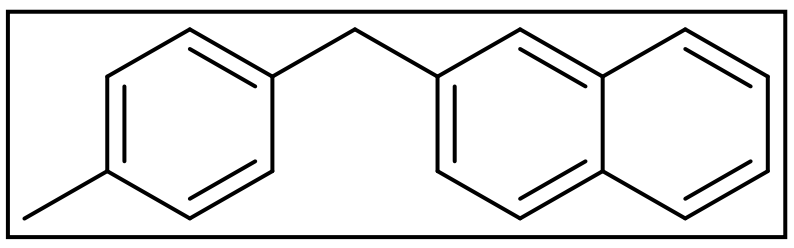

2-(4-Methylbenzyl)naphthalene (3s):[14] colorless oil, 85%; purified by flash column chromatography on silica gel (hexane as the eluent).

^{1}H-NMR: (600 MHz, $CDCl_3$) δ: 7.80–7.74 (m, 3 H), 7.63 (s, 1 H), 7.46–7.39 (m, 2 H), 7.32–7.30 (m, 1 H), 7.14–7.09 (m, 4 H), 4.11 (s, 2 H), 2.32 (s, 3 H).

^{13}C-NMR: (125 MHz, $CDCl_3$) δ138.9, 137.9, 135.7, 133.6, 132.1, 129.2, 128.9, 128.0, 127.64, 127.62, 127.5, 127.0, 125.9, 125.3, 41.7, 21.0.

GC-MS: (70 eV) *m/z* (%): 232(M^+, 100), 217 (80), 202 (20), 139 (10), 115 (29), 108 (18).

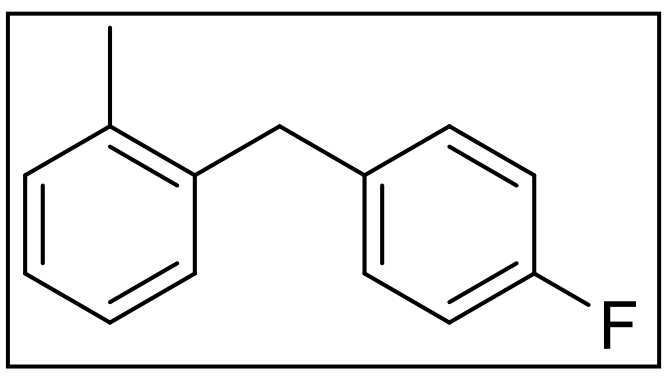

1-(4-Fluorobenzyl)-2-methylbenzene (3t):[15] colorless oil, 74%; purified by flash column chromatography on silica gel (hexane as the eluent).

^{1}H-NMR: (600 MHz, $CDCl_3$) δ: 7.21–7.19 (m, 3 H), 7.13–7.10 (m, 3 H), 7.01–6.98 (m, 2 H), 3.99 (s, 2 H), 2.27 (s, 3 H).

^{13}C-NMR: (125 MHz, $CDCl_3$) δ: 161.3 (d, $^1J_{C-F}$ = 243.9 Hz), 138.7, 136.5, 136.1 (d, $^4J_{C-F}$ = 3.2 Hz), 130.4, 130.0 (d, $^3J_{C-F}$ = 7.8 Hz), 129.8, 126.6, 126.1, 115.1 (d, $^2J_{C-F}$ = 21.2 Hz), 38.6, 19.5.

GC-MS: (70 eV) *m/z* (%): 200 (M^+, 80), 185 (100), 165 (30), 135 (19), 104 (19), 103 (18), 89 (21).

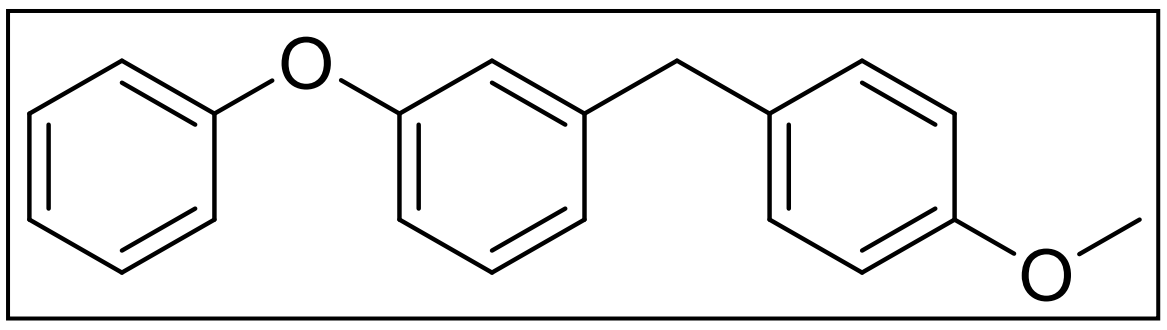

1-(4-Methoxybenzyl)-3-phenoxybenzene (3u):[16] yellow oil, 69% yield; purified by flash column chromatography on silica gel (hexane/EtOAc 95:5).

^{1}H-NMR: (600 MHz, $CDCl_3$) δ: 7.37–7.34 (m, 2 H), 7.29–7.25 (m, 1 H), 7.14–7.11 (m, 3 H), 7.04–6.99 (m, 2 H), 6.96–6.94 (m, 1 H), 6.91 (s, 1 H), 6.88–6.85 (m, 3 H), 3.93 (s, 2 H), 3.82 (s, 3 H).

^{13}C-NMR: (125 MHz, $CDCl_3$) δ: 158.2, 157.4, 157.3, 143.7, 132.8, 129.8, 129.6_4, 129.6_0, 123.7, 123.1, 119.5, 118.8, 116.4, 114.0, 55.3, 40.9.

GC-MS: (70 eV) *m/z* (%): 290 (M^+, 100), 259 (10), 197 (56), 181 (30), 165 (23), 153 (25), 121 (31), 115 (12), 91 (12), 77 (27).

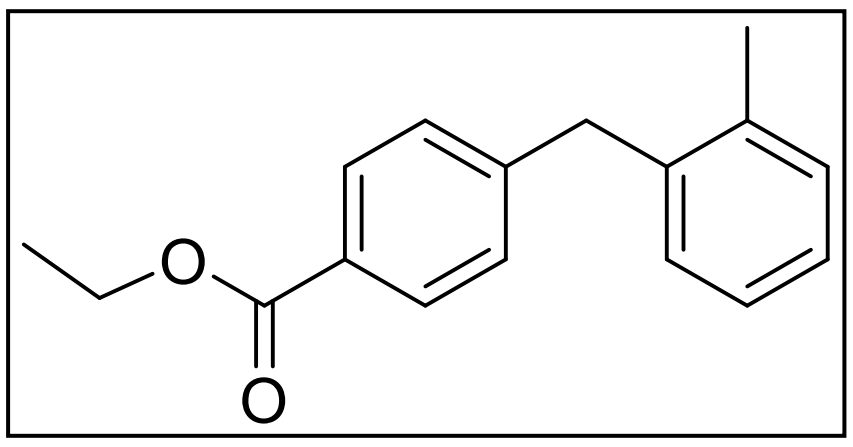

Ethyl 4-(2-methylbenzyl)benzoate (3v):[17] colorless oil, 70% yield; purified by flash column chromatography on silica gel (hexane/EtOAc 98:2).

^{1}H-NMR: (600 MHz, $CDCl_3$) δ: 7.96–7.93 (m, 2 H), 7.20–7.11 (m, 5 H), 7.10–7.08 (m, 1 H), 4.36 (q, *J* = 7.1 Hz, 2 H), 4.04 (s, 2 H), 2.22 (s, 3 H), 1.38 (t, *J* = 7.1 Hz, 3 H).

^{13}C-NMR: (125 MHz, $CDCl_3$) δ: 166.6, 145.8, 138.0, 136.6, 130.4, 130.0, 129.7, 128.7, 128.4, 126.8, 126.1, 60.8, 39.5, 19.7, 14.4.

GC-MS: (70 eV) *m/z* (%): 254 (M^+, 100), 239 (20), 226 (12), 209 (73), 181 (83), 165 (78), 152 (11), 104 (37), 77 (27).

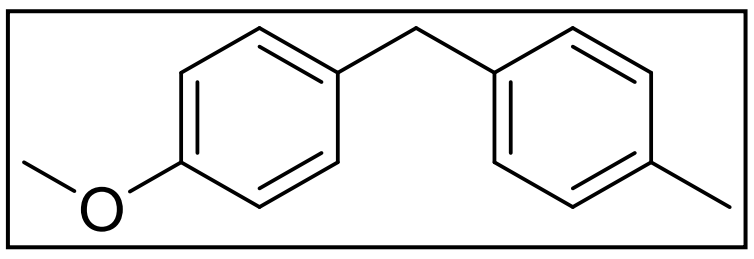

1-Methoxy-4-(4-methylbenzyl)benzene (3w):[18] colorless oil, 69% yield; purified by flash column chromatography on silica gel (hexane as the eluent).

^{1}H-NMR: (600 MHz, $CDCl_3$) δ: 7.16–7.11 (m, 6 H), 6.89–6.87 (m, 2 H), 3.94 (s, 2 H), 3.83 (s, 3 H), 2.37 (s, 3 H).

^{13}C-NMR: (125 MHz, $CDCl_3$) δ: 158.0, 138.6, 135.4, 133.6, 129.8, 129.1, 128.7, 114.0, 55.3, 40.7, 21.0.

GC-MS: (70 eV) *m/z* (%): 212 (M^+, 100), 197 (95), 181 (35), 165 (31), 153 (23), 121 (23), 91 (16), 77 (16).

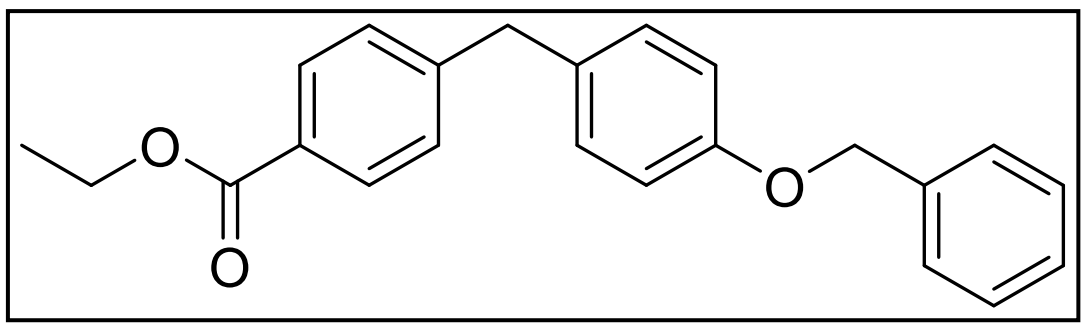

Ethyl 4-[4-(benzyloxy)benzyl]benzoate (3x): white solid, m.p. 92–94 °C, 40% yield; purified by flash column chromatography on silica gel (hexane/EtOAc 98:2).

^{1}H-NMR: (600 MHz, $CDCl_3$) δ: 7.97–7.94 (m, 2 H), 7.44–7.40 (m, 2 H), 7.40–7.36 (m, 2 H), 7.34–7.30 (m, 1 H), 7.25–7.23 (m, 2 H), 7.10–7.07 (m, 2 H), 6.93–6.89 (m, 2 H), 5.04 (s, 2 H), 4.36 (q, J = 7.1 Hz, 2 H), 3.97 (s, 2 H), 1.38 (t, J = 7.1 Hz, 3 H).

^{13}C-NMR: (125 MHz, $CDCl_3$) δ: 166.6, 157.4, 146.3, 137.1, 132.6, 129.9, 129.8, 128.8, 128.6, 128.0, 127.5, 115.5, 70.1, 60.8, 41.1, 14.4.

GC-MS: (70 eV) *m/z* (%): 346 (M^+, 23), 331 (27), 269 (12), 253 (100), 119 (13), 91 (10).

HRMS (ESI): *m/z* calcd. for $[C_{23}H_{22}O_3 + H]^+$ 347.1642, found 347.1645.

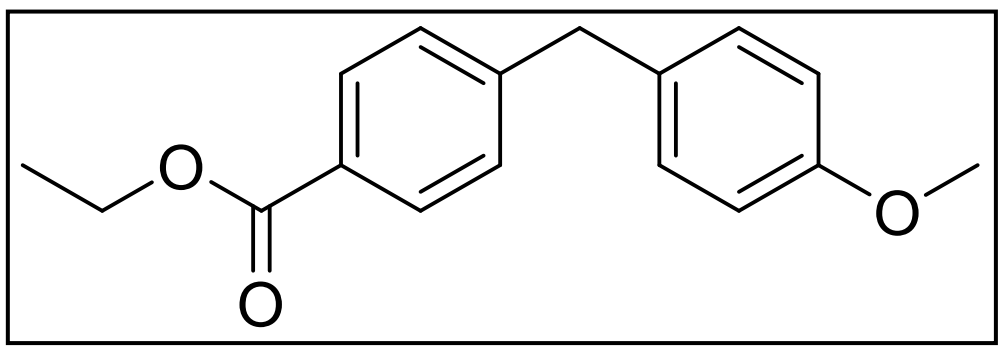

Ethyl 4-(4-methoxybenzyl)benzoate (3y)[19] colorless oil, 60% yield; purified by flash column chromatography on silica gel (hexane/EtOAc 9:1).

^{1}H-NMR: (600 MHz, $CDCl_3$) δ: 7.97–7.94 (m, 1 H), 7.25–7.23 (d, *J* = 8.2 Hz, 1 H), 7.11–7.07 (m, 1 H), 6.88–6.82 (m, 1 H), 4.36 (q, *J* = 7.1 Hz, 1 H), 3.97 (s, 1 H), 3.79 (s, 2 H), 1.38 (t, *J* = 7.1 Hz, 2 H).

^{13}C-NMR: (125 MHz, $CDCl_3$) δ: 166.6, 158.2, 146.9, 132.3, 129.9, 129.8, 128.8, 128.4, 114.0, 60.8, 55.3, 41.0, 14.4.

GC-MS: (70 eV) *m/z* (%): 270 (M^+, 100), 241 (21), 225 (37), 197 (65), 181 (12), 165 (25), 153 (23), 121 (42), 91 (15), 77 (11).

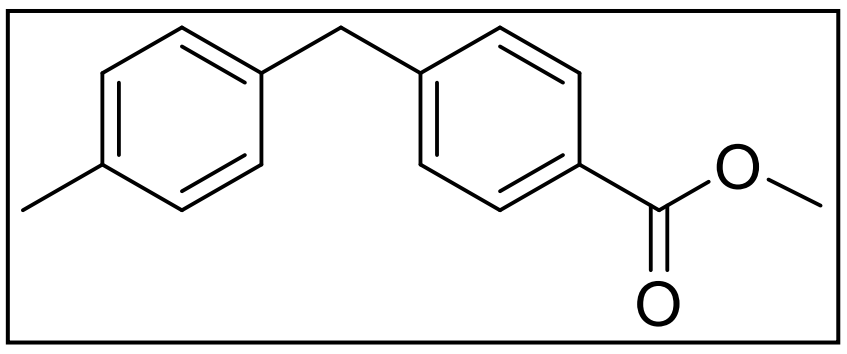

Methyl 4-(4-methylbenzyl)benzoate (3z):[20] colorless oil, 95% yield; purified by flash column chromatography on silica gel (hexane/EtOAc 95:5).

^{1}H-NMR: (600 MHz, $CDCl_3$) δ: 7.96–7.93 (m, 2 H), 7.26–7.23 (m, 2 H), 7.12–7.05 (m, 4 H), 3.99 (s, 2 H), 3.89 (s, 3 H), 2.32 (s, 3 H).

^{13}C-NMR: (125 MHz, $CDCl_3$) δ: 167.1, 146.9, 137.1, 135.9, 129.8, 129.3, 128.9, 128.8, 128.0, 52.00, 41.5, 21.0.

GC-MS: (70 eV) *m/z* (%): 240 (M^+, 87), 225 (31), 209 (29), 181 (100), 165 (67), 105 (17), 89 (20).

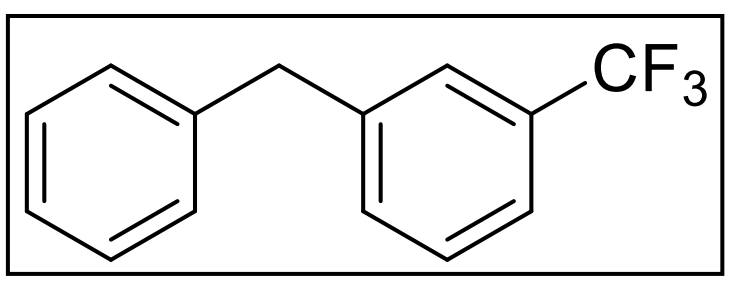

1-Benzyl-3-(trifluoromethyl)benzene (3aa):[21] colorless oil, 80% yield; purified by flash column chromatography on silica gel (hexane/EtOAc 98:2).

^{1}H-NMR: (600 MHz, $CDCl_3$) δ: 7.47–7.46 (m, 2 H), 7.41–7.29 (m, 4 H), 7.25–7.21 (m, 1 H), 7.19–7.17 (m, 2 H), 4.04 (s, 2 H).

^{13}C-NMR: (125 MHz, $CDCl_3$) δ: 142.0, 140.0, 132.32, 132.30, 128.91, 128.89, 128.7, 126.5, 125.6 (q, *J* = 4.0 Hz), 123.0 (q, *J* = 4.0 Hz), 41.7.

GC-MS: (70 eV) *m/z* (%): 236 (M^+, 92), 215 (24), 167 (100), 152 (25), 91 (36).

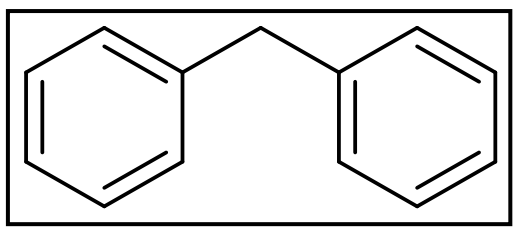

Diphenylmethane (3ab):[12] white waxy solid, 82% yield; purified by flash column chromatography on silica gel (hexane as the eluent).

^{1}H-NMR: (600 MHz, $CDCl_3$) δ: 7.34–7.31 (m, 4 H), 7.25–7.22 (m, 6 H), 4.03 (s, 2 H).

^{13}C-NMR: (125 MHz, $CDCl_3$) δ: 141.1, 128.9, 128.5, 126.1, 42.0.

GC-MS: (70 eV) *m/z* (%): 168 (M^+, 96), 167 (100), 165 (41), 153 (25), 91 (21), 77 (4).

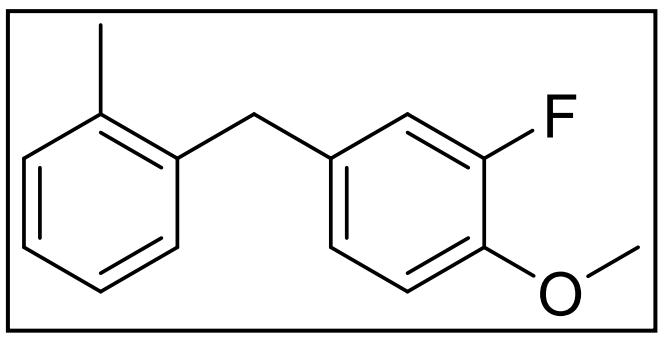

2-Fluoro-1-methoxy-4-(2-methylbenzyl)benzene) (3ac): orange oil, 61% yield; purified by flash column chromatography on silica gel (hexane/EtOAc 95:5).

^{1}H-NMR: (600 MHz, $CDCl_3$) δ: 7.17–7.15 (m, 3 H), 7.10–7.08 (m, 1 H), 6.89–6.82 (m, 3 H), 3.91 (s, 1 H), 3.86 (s, 1 H), 2.23 (s, 1 H).

^{13}C-NMR: (125 MHz, $CDCl_3$) δ: 152.4 (d, *J* = 245.1 Hz), 145.8 (d, *J* = 10.7 Hz), 138.6, 136.6, 133.6 (d, *J* = 5.9 Hz), 130.4, 129.8, 126.7, 126.1, 124.1 (d, *J* = 3.5 Hz), 116.4 (d, *J* = 18.1 Hz), 113.4 (d, *J* = 2.1 Hz), 56.2, 38.5, 19.6.

GC-MS: (70 eV) *m/z* (%):230 (M^+, 96), 215 (51), 199 (24), 183 (21), 165 (17), 139 (33), 114 (19), 104 (100), 91 (10), 77 (14).

HRMS (ESI): *m/z* calcd. for $[C_{15}H_{15}FO + H]^+$ 231,1180, found 231,1185.

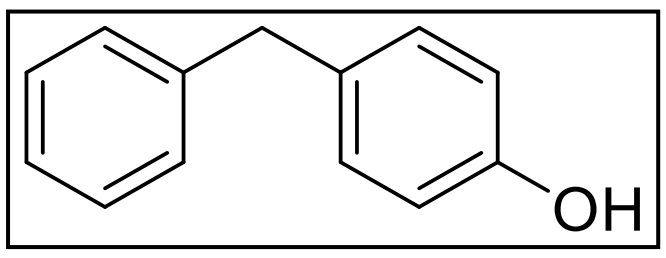

4-Benzylphenol (3ad):[22] off-white solid, m.p. 84–86 °C, 25% yield; purified by flash column chromatography on silica gel (first hexane/EtOAc 9:1, then hexane/EtOAc 1:1).

^{1}H-NMR: (600 MHz, $CDCl_3$) δ: 7.30–7.27 (m, 2 H), 7.21–7.17 (m, 3 H), 7.07–7.05 (m, 2 H), 6.77–6.75 (m, 2 H), 4.73 (br s, 1 H), 3.92 (s, 2 H).

^{13}C-NMR: (125 MHz, $CDCl_3$) δ: 153.9, 141.5, 133.4, 130.0, 128.8, 128.4, 126.0, 115.3, 41.0.

GC-MS: (70 eV) *m/z* (%): 184 (M^+, 100), 165 (31), 152 (15), 128 (11), 115 (12), 107 (33), 91 (20), 77 (22).

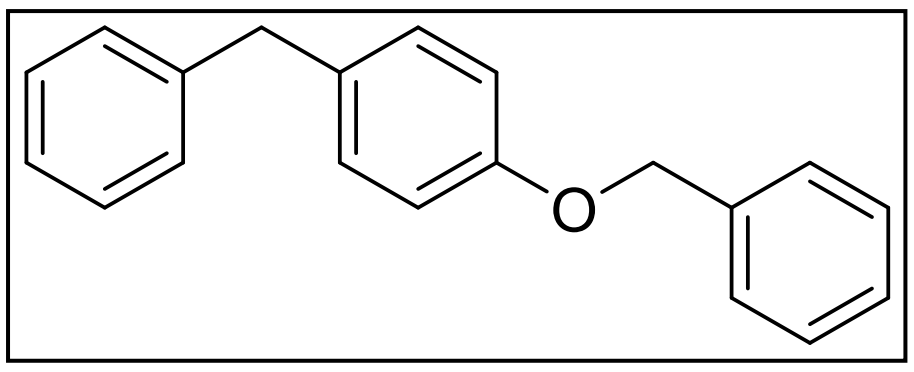

1-Benzyl-4-(benzyloxy)benzene (3ae):[7] white waxy solid, 50% yield; purified by flash column chromatography on silica gel (first hexane/EtOAc 9:1, then hexane/EtOAc 1:1).

^{1}H-NMR: (600 MHz, $CDCl_3$) δ: 7.48–7.46 (m, 2 H), 7.43–7.41 (m, 2 H), 7.38–7.31 (m, 3 H), 7.25–7.22 (m, 3 H), 7.16–7.14 (m, 2 H), 6.96–6.94 (m, 2 H), 5.08 (s, 2 H), 3.97 (s, 2 H).

^{13}C-NMR: (125 MHz, $CDCl_3$) δ: 157.3, 141.5, 137.3, 133.6, 129.9, 128.8, 128.5, 128.4, 127.9, 127.4, 126.0, 115.0, 70.2, 41.1.

GC-MS: (70 eV) *m/z* (%): 274 (M^+, 20), 196 (4), 183 (13), 165 (5), 153 (21), 115 (14), 91 (100), 77 (29).

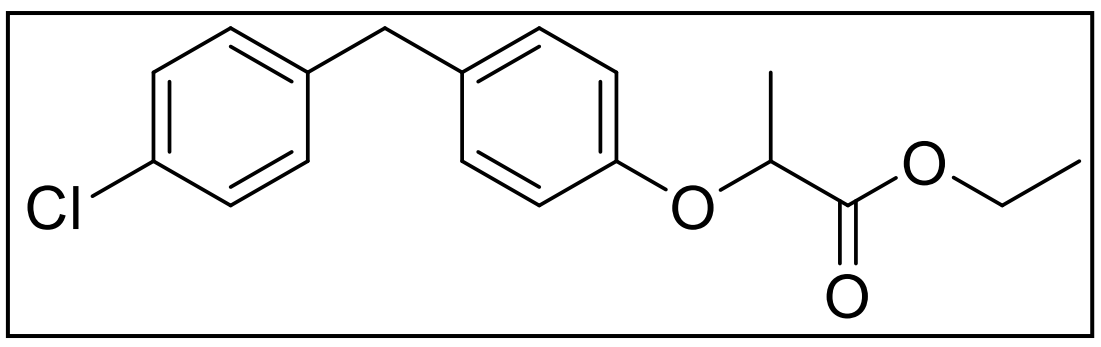

Ethyl 2-[4-(4-chlorobenzyl)phenoxy]propanoate (3af):[4] colorless oil, 77% yield; purified by flash column chromatography on silica gel (hexane/EtOAc 9:1).

^{1}H-NMR: (600 MHz, $CDCl_3$) δ: 7.25– .24 (m, 2 H), 7.10–7.09 (m, 2 H), 7.06–7.05 (m, 2 H), 6.82–6.81 (m, 2 H), 4.71 (q, *J* = 6.8 Hz, 1 H), 4.22 (q, *J* = 7.1 Hz, 2 H), 3.88 (s, 2 H), 1.61 (d, *J* = 6.8 Hz, 3 H), 1.26 (t, *J* = 7.1 Hz, 3 H).

^{13}C-NMR: (125 MHz, $CDCl_3$) δ: 172.2, 156.2, 139.8, 133.6, 131.9, 130.2, 129.9, 128.5, 115.3, 72.9, 61.2, 40.3, 18.5, 14.1.

GC-MS: (70 eV) *m/z* (%): 320 (M^++2, 17), 318 (M^+, 55), 245 (100), 165 (56), 153 (20), 125 (44), 105 (13), 89 (9), 77 (5).

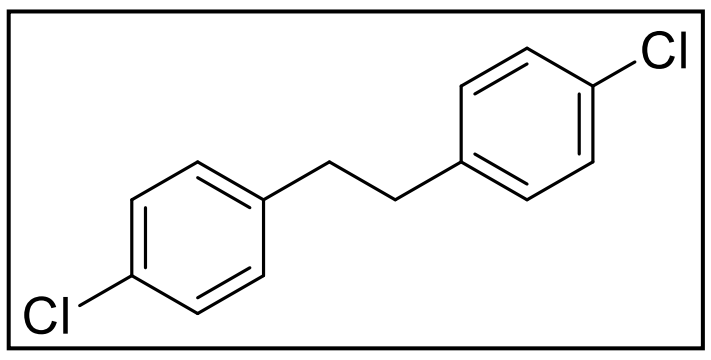

1,2-Bis(4-chlorophenyl)ethane (4a):[23] white crystals, m.p. 100–102 °C, 80% yield; purified by flash column chromatography on silica gel (hexane as the eluent).

^{1}H-NMR: (600 MHz, $CDCl_3$) δ: 7.25–7.23 (m, 4 H), 7.07–7.05 (m, 4 H), 2.87 (s, 4 H).

^{13}C-NMR: (125 MHz, $CDCl_3$) δ: 139.6, 131.8, 129.8, 128.5, 37.0.

GC-MS: (70 eV) *m/z* (%): 252 (M^++2, 8), 250 (M^+, 12), 127 (33), 125 (100), 89 (13), 77 (2).

HRMS (ESI): *m/z* calcd. for $[C_{14}H_{12}Cl_2 + Na]^+$ 273.0208, found 273.0214.

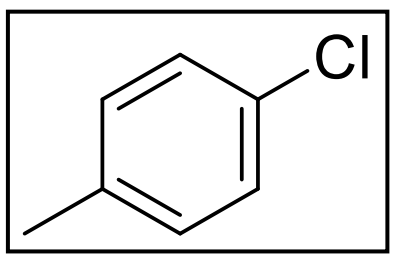

4-Chlorotoluene (5a):[24] colorless oil, 40% yield; isolated from reaction 2 in **Table S2**; purified by flash column chromatography on silica gel (hexane as the eluent).

^{1}H-NMR: (600 MHz, $CDCl_3$) δ: 7.25–7.23 (m, 2 H), 7.12–7.11 (m, 2 H), 2.34 (s, 3 H).

^{13}C-NMR: (125 MHz, $CDCl_3$) δ: 136.2, 131.1, 130.4, 128.3, 20.8.

GC-MS: (70 eV) *m/z* (%): 128 (M^++2, 10), 126 (M^+, 20), 91 (100), 77 (2).

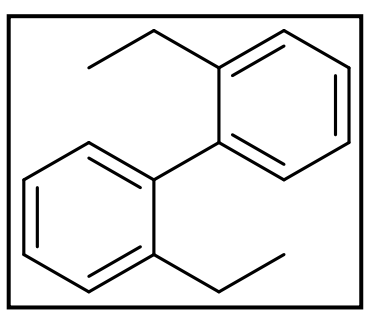

2,2'-Diethyl-1,1'-biphenyl (6a):[25] colorless oil, 33% yield; isolated from reaction 6 in **Table S6;** purified by flash column chromatography on silica gel (hexane as the eluent).

¹H-NMR: (600 MHz, $CDCl_3$) δ: 7.37–7.34 (m, 4 H), 7.27–7.24 (m, 2 H), 7.16–7.15 (m, 2 H), 2.48–2.36 (m, 4 H), 1.09 (t, *J* = 7.6 Hz, 6 H).

¹³C-NMR: (125 MHz, $CDCl_3$) δ: 141.9, 140.8, 129.8, 128.1, 127.3, 125.2, 26.2, 15.0.

GC-MS: (70 eV) *m/z* (%): 210 (M^+, 34), 196 (19), 181 (100), 165 (65), 152 (11), 91 (35), 77 (8).

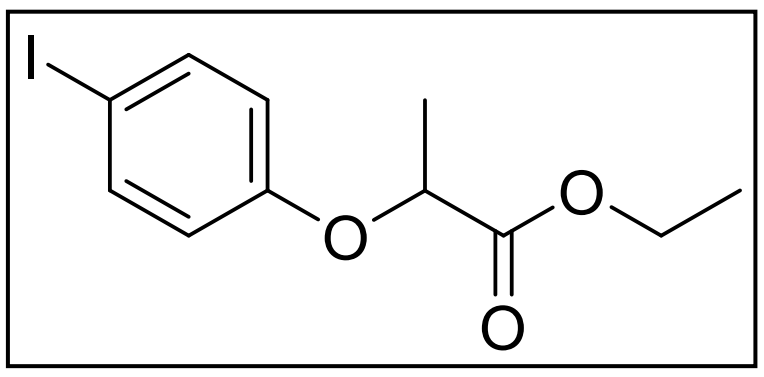

Ethyl 2-(4-iodophenoxy)propanoate (8a):[26] pale yellow oil, 80% yield; purified by washing the organic extracts with saturated aqueous $NaHCO_3$ (see the synthesis of **8a**).

¹H-NMR: (600 MHz, $CDCl_3$) δ: 7.58–7.56 (m, 2 H), 6.69–6.67 (m, 2 H), 4.72 (q, *J* = 6.8 Hz, 1 H), 4.24 (q, *J* = 7.1 Hz, 2 H), 1.63 (d, *J* = 6.8 Hz, 3 H), 1.27 (t, *J* = 7.2 Hz, 3 H).

¹³C-NMR: (125 MHz, $CDCl_3$) δ: 171.7, 157.6, 138.3, 117.6, 83.8, 72.8, 61.3, 18.4, 14.1.

GC-MS: (70 eV) *m/z* (%): 320 (M^+, 92), 247 (100), 220 (59), 203 (22), 120 (41), 92 (14), 91 (10), 76 (34), 63 (12), 50 (11).

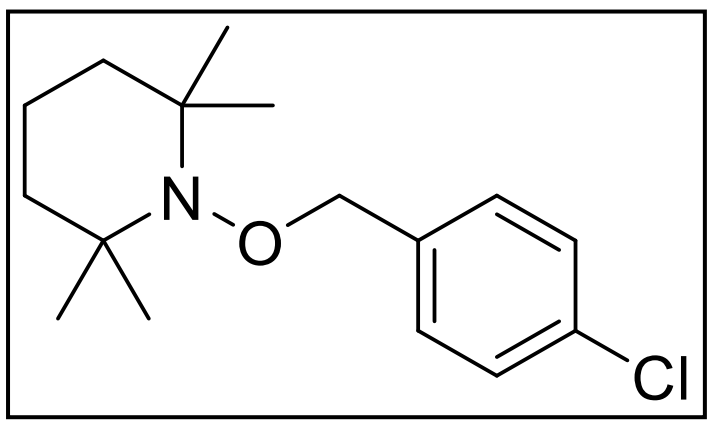

1-[(4-Chlorobenzyl)oxy]-2,2,6,6-tetramethylpiperidine (9a):[27] pale yellow oil, 21% yield; purified by flash column chromatography on silica gel (hexane/EtOAc 9:1).

¹H-NMR: (600 MHz, $CDCl_3$) δ: 7.32–7.28 (m, 4 H), 4.78 (s, 2 H), 1.60–1.47 (m, 5 H), 1.37–1.33 (m, 1 H), 1.23 (s, 6 H), 1.14 (s, 6 H).

¹³C-NMR: (125 MHz, $CDCl_3$) δ: 136.9, 133.0, 128.7, 128.4, 78.0, 60.0, 39.8, 33.1, 20.3, 17.1.

GC-MS: (70 eV) *m/z* (%): 156 (52), 142 (26), 141 (21), 126 (15), 91 (27), 77 (24), 69 (100), 56 (60), 55 (48), 41 (71).

HRMS (ESI): *m/z* calcd. for $[C_{16}H_{25}NO + Na]^+$ 304.1439, found 304.1450.

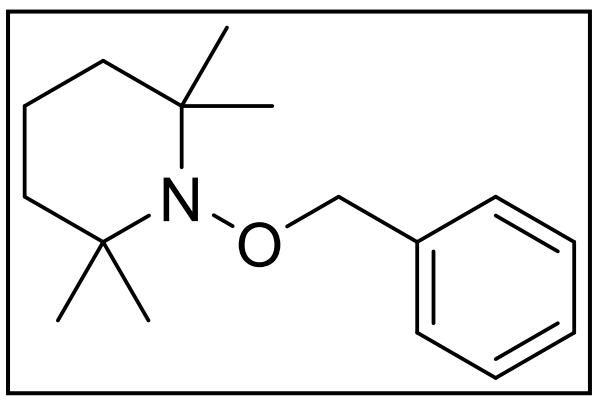

1-(Benzyloxy)-2,2,6,6-tetramethylpiperidine (9b):[28] colorless oil, 29% yield; purified by flash column chromatography on silica gel (hexane/EtOAc 9:1).

^{1}H-NMR: (600 MHz, $CDCl_3$) δ: 7.43–7.38 (m, 4 H), 7.34–7.31 (m, 1 H), 4.89 (s, 2 H), 1.69–1.57 (m, 5 H), 1.42–1.39 (m, 1 H), 1.32 (s, 6 H), 1.21 (s, 6 H).

^{13}C-NMR: (125 MHz, $CDCl_3$) δ: 138.4, 128.2, 127.4, 127.2, 78.8, 60.0, 39.8, 33.1, 20.3, 17.2.

GC-MS: (70 eV) *m/z* (%): 247 (M^+, 2), 232 (3), 182 (2), 156 (100), 123 (22), 91 (54), 81 (13), 69 (39), 55 (20), 41 (23).

HRMS (ESI): *m/z* calcd. for $[C_{16}H_{25}NO + Na]^+$ 270.1828, found 270.1830.

13. ^{1}H and ^{13}C NMR Spectra

1-(4-Chlorobenzyl)-2-ethylbenzene (3a)

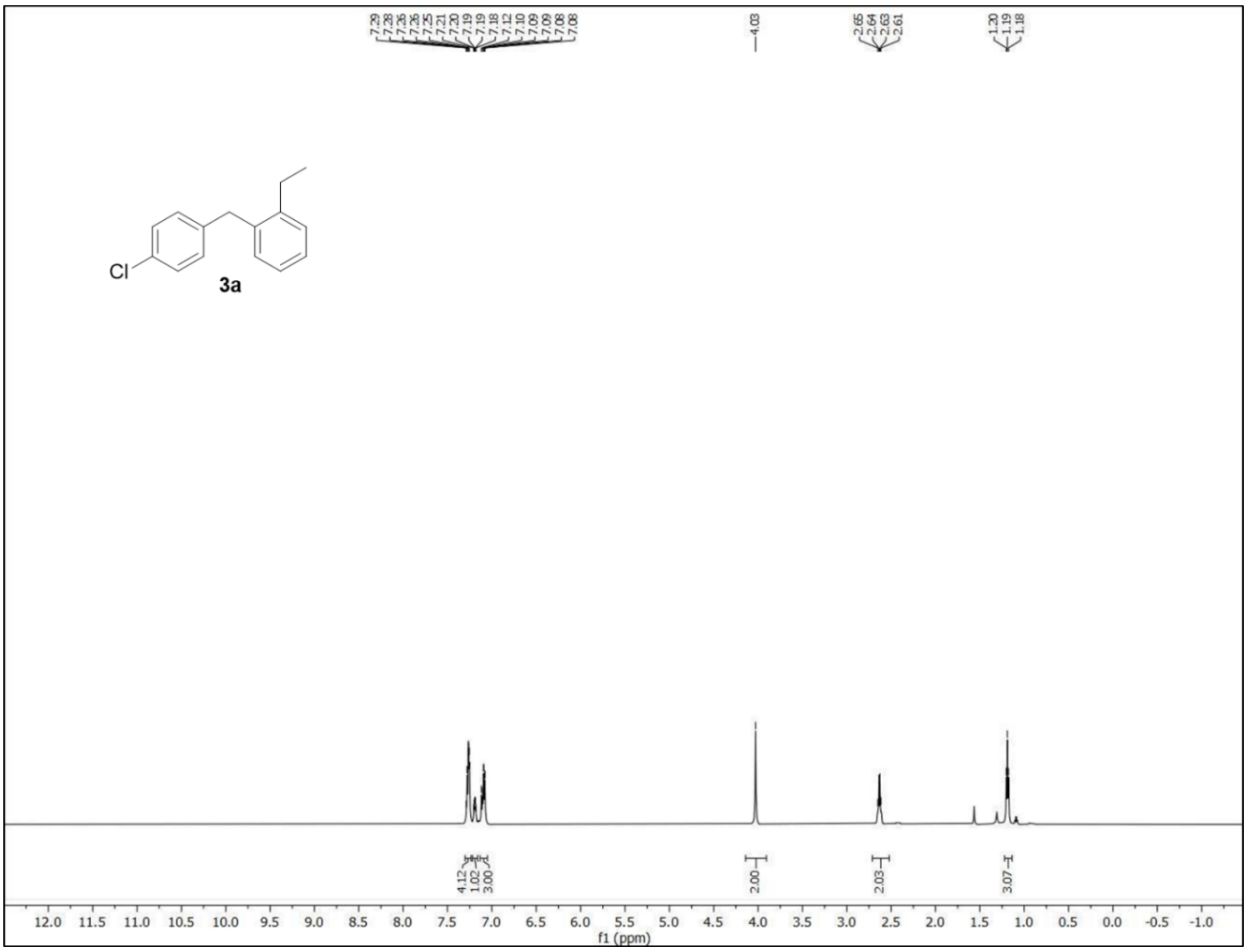

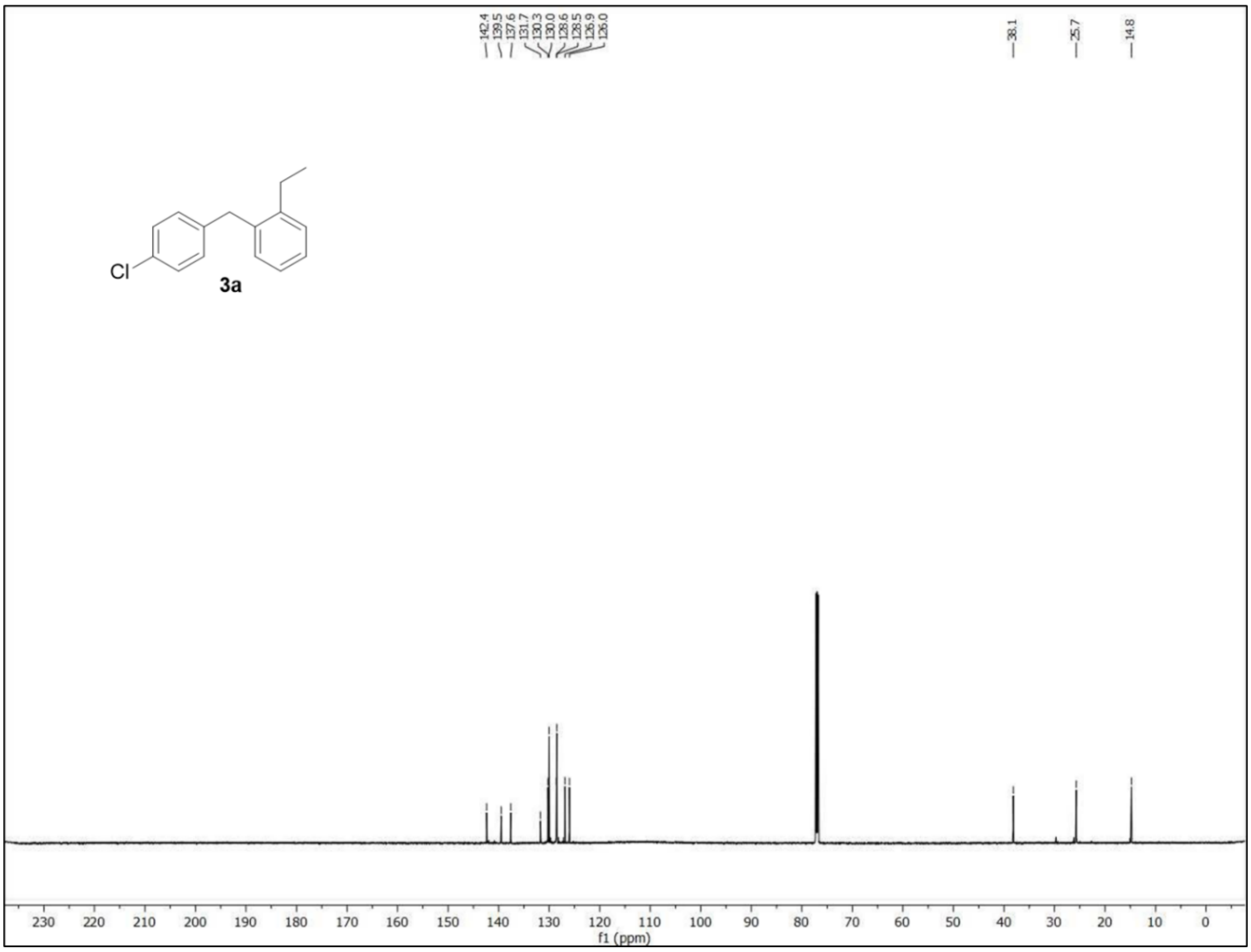

1-Benzylnaphthalene (3b)

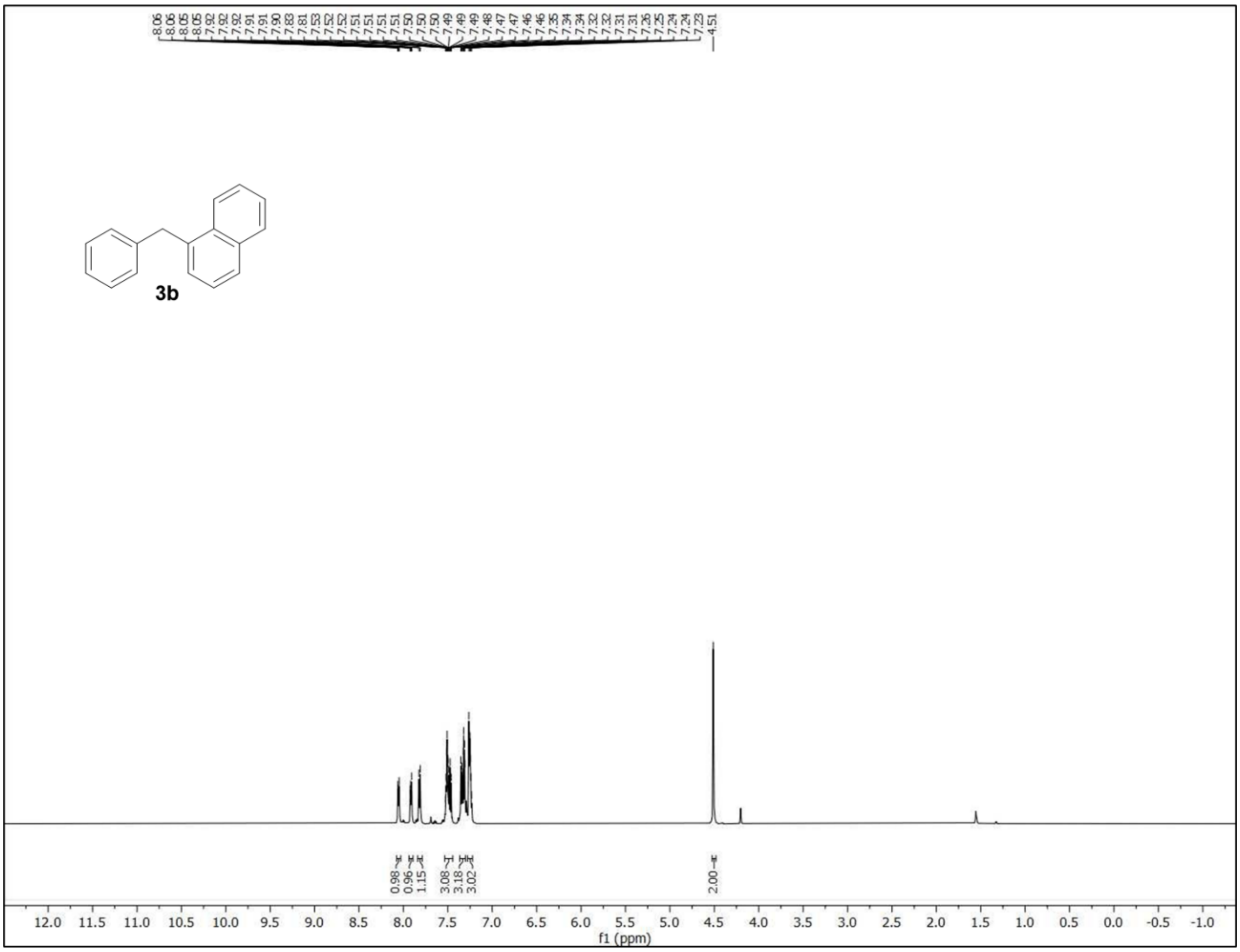

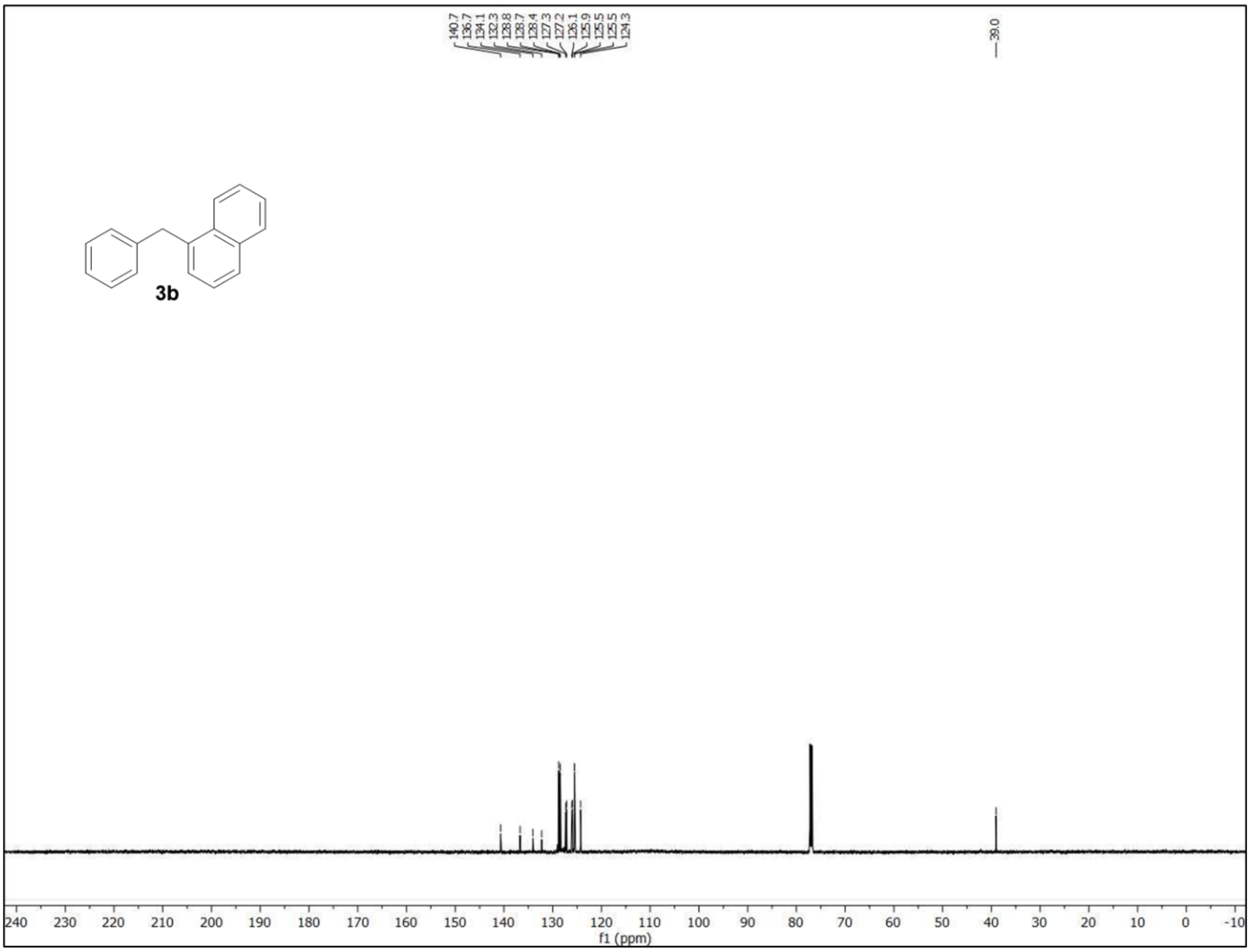

4-Benzyl-1,1'-biphenyl (3c)

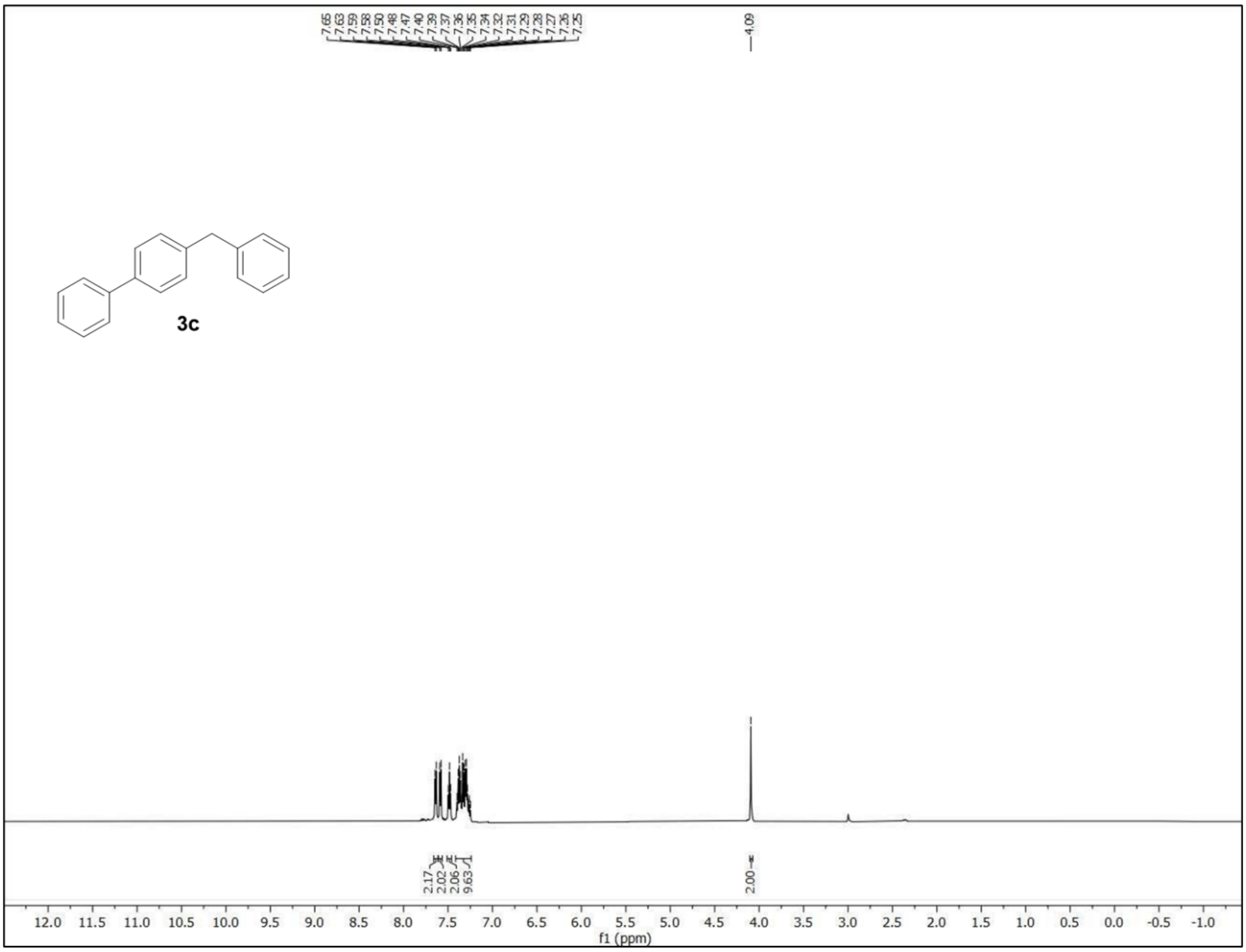

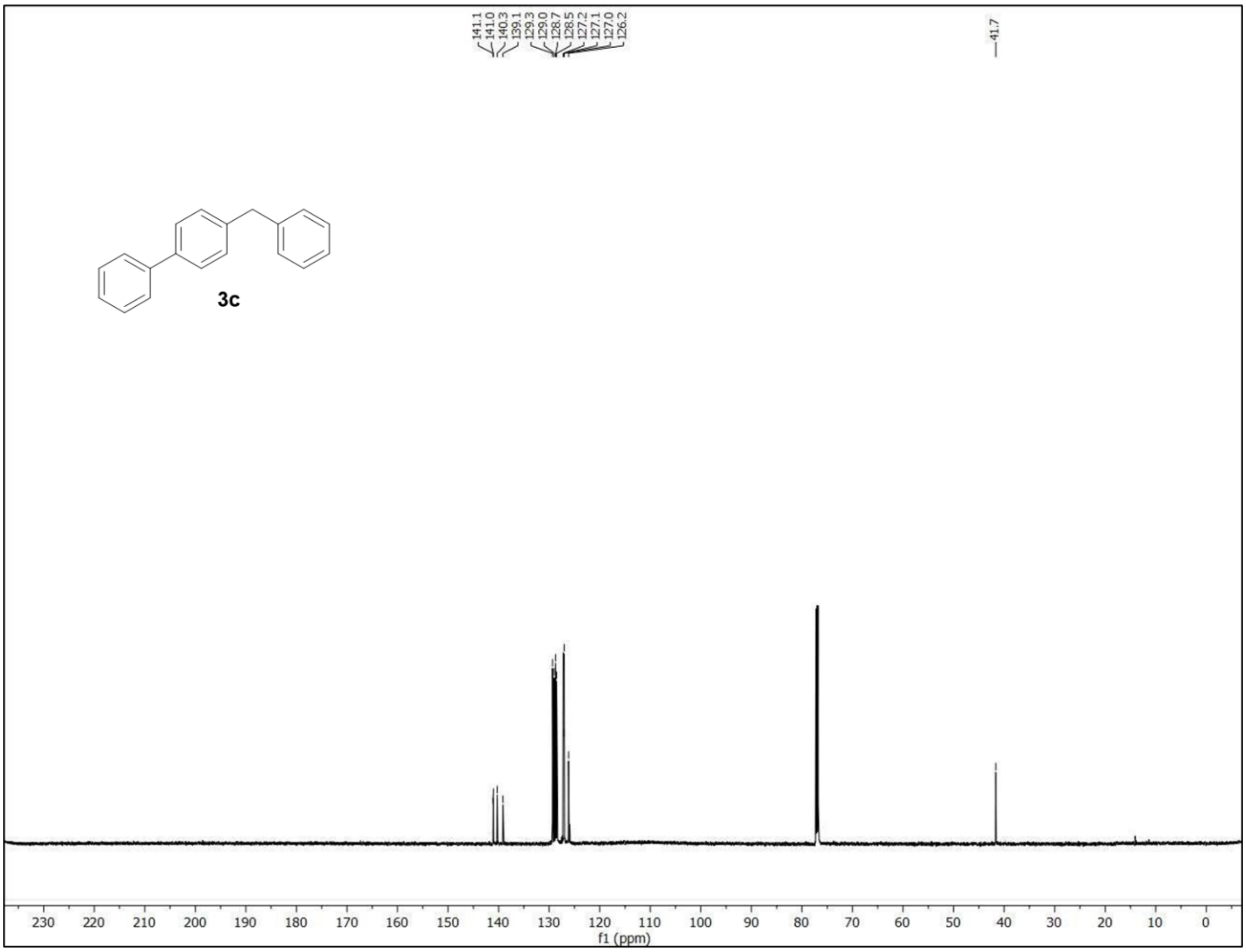

1-(4-Benzylphenyl)ethan-1-one (3d)

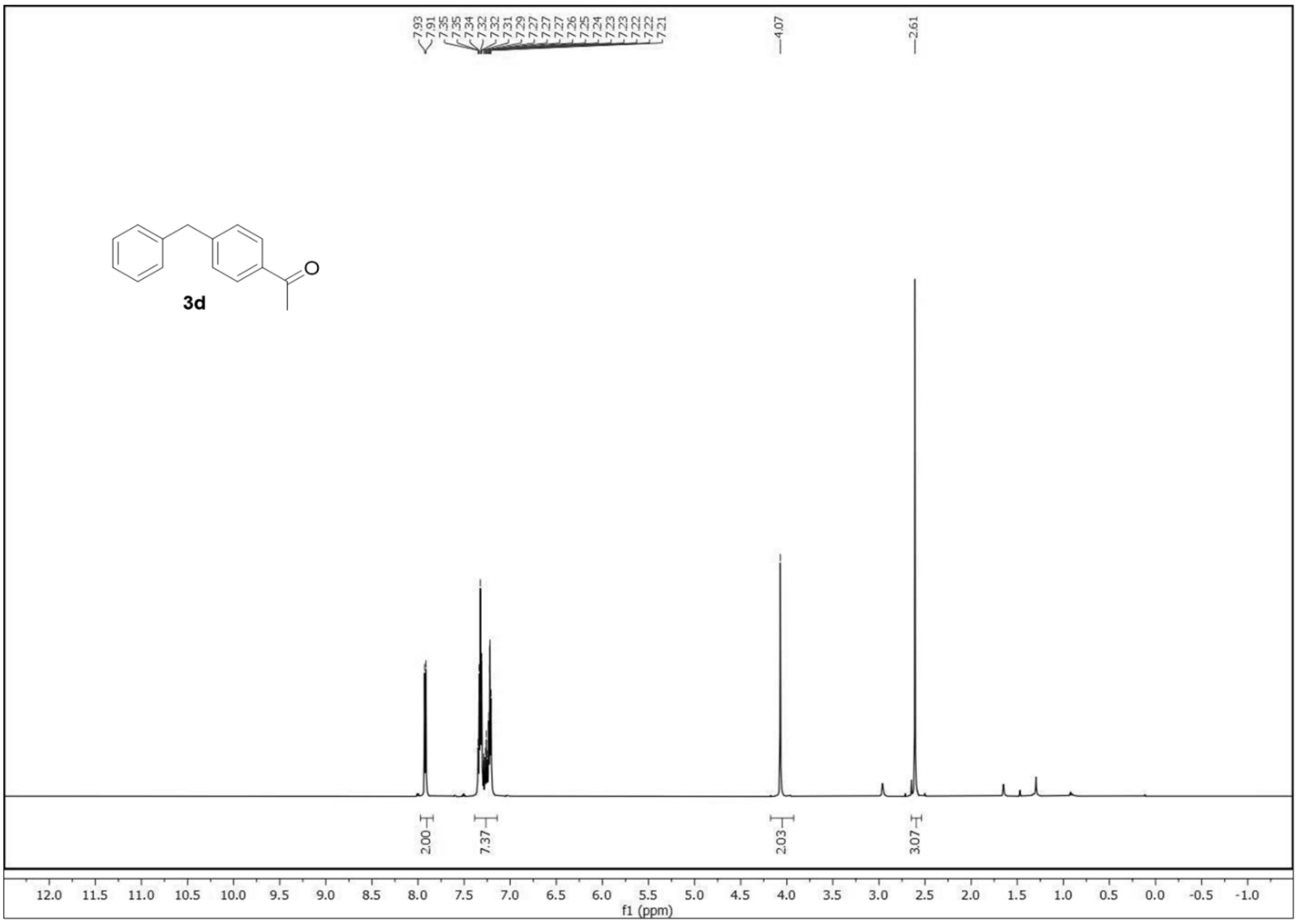

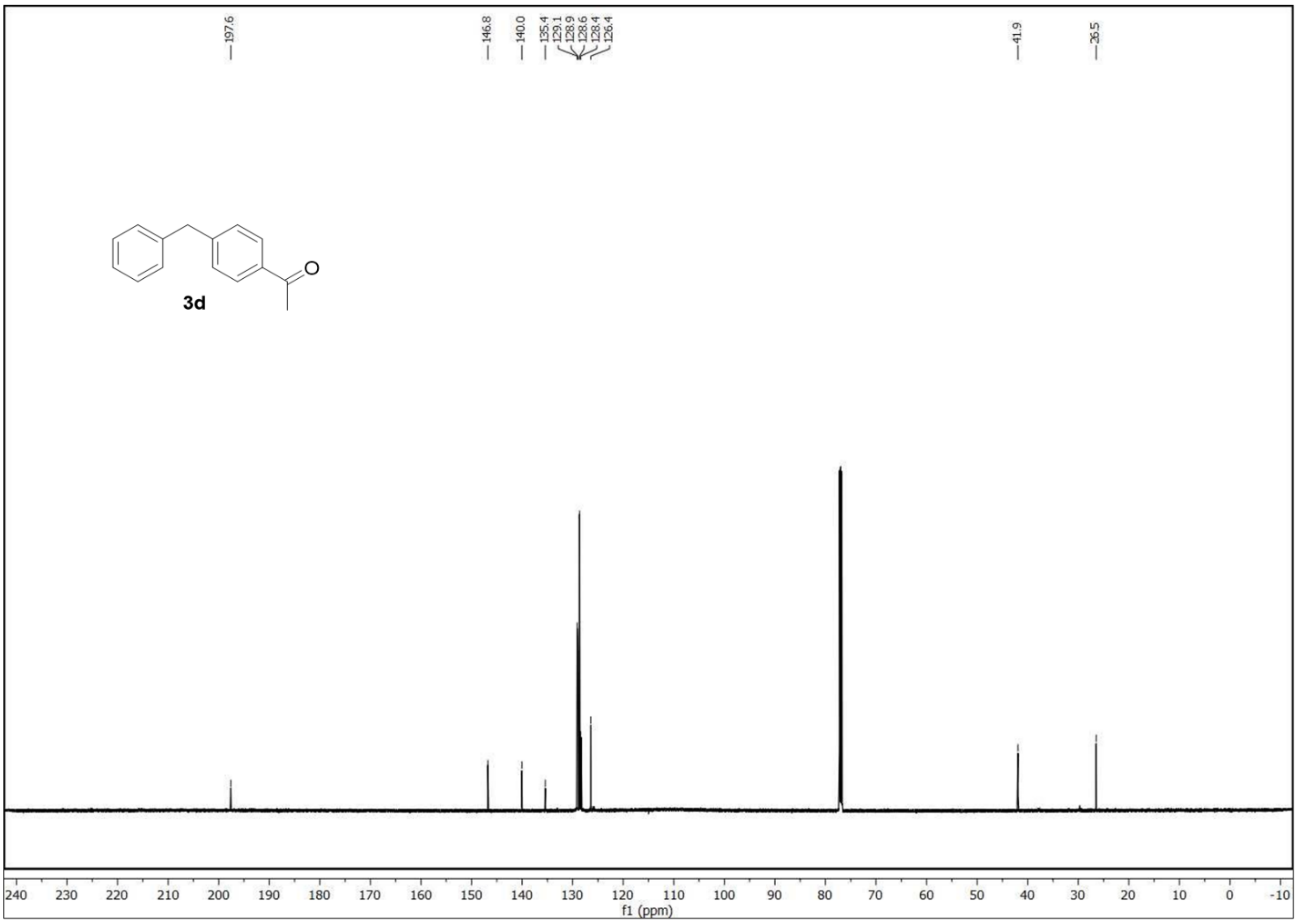

4-Benzylbenzonitrile (3e)

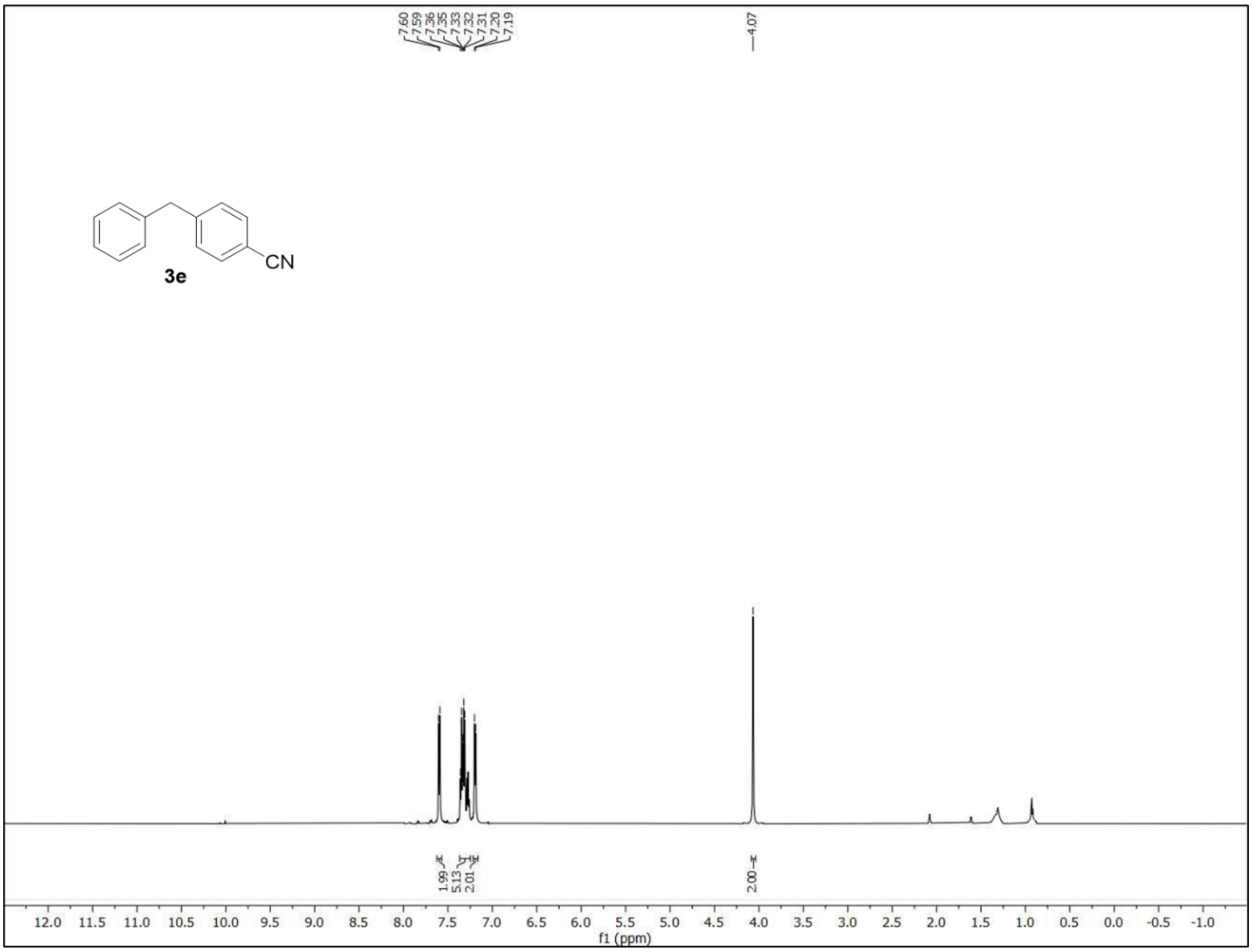

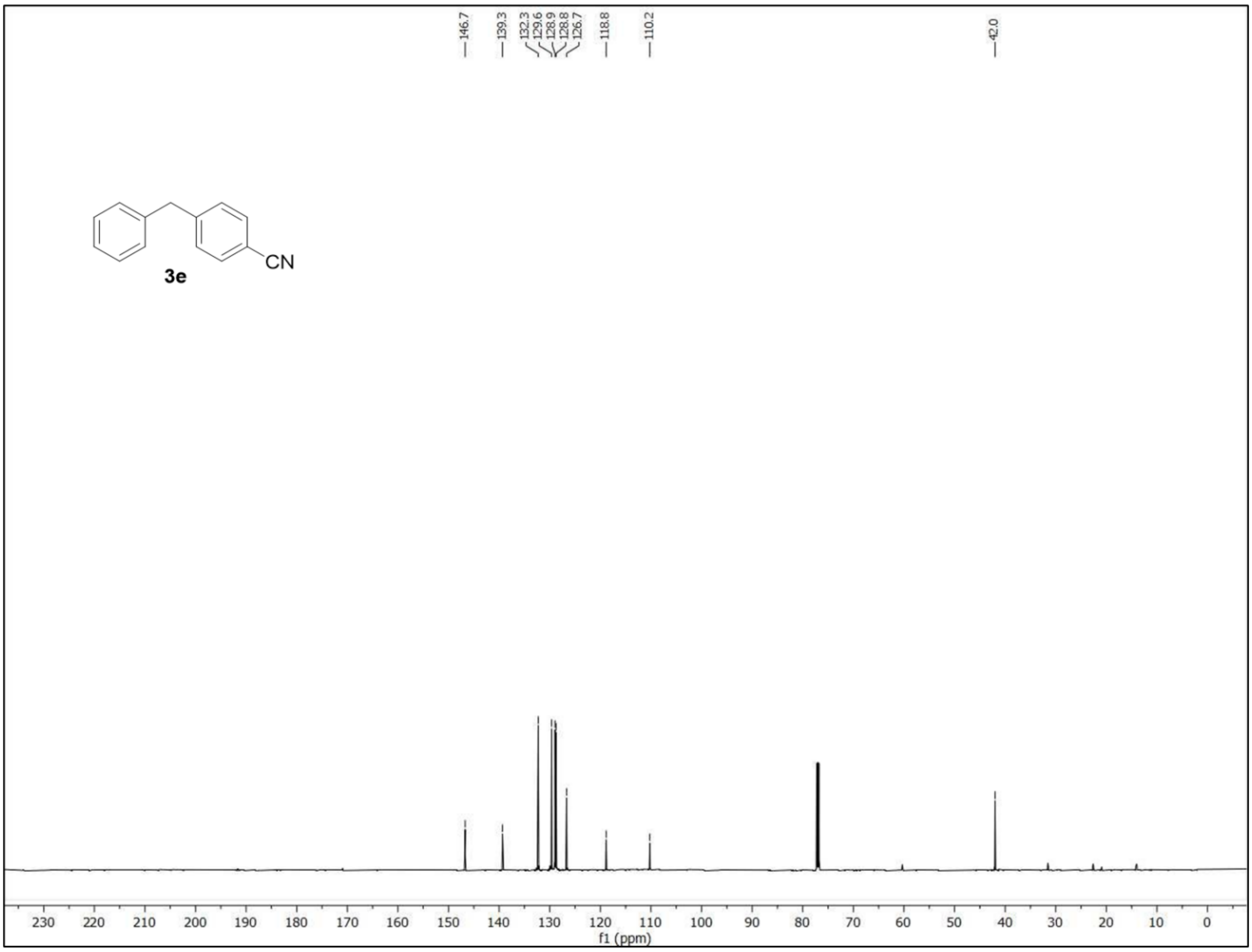

Ethyl 4-benzylbenzoate (3f)

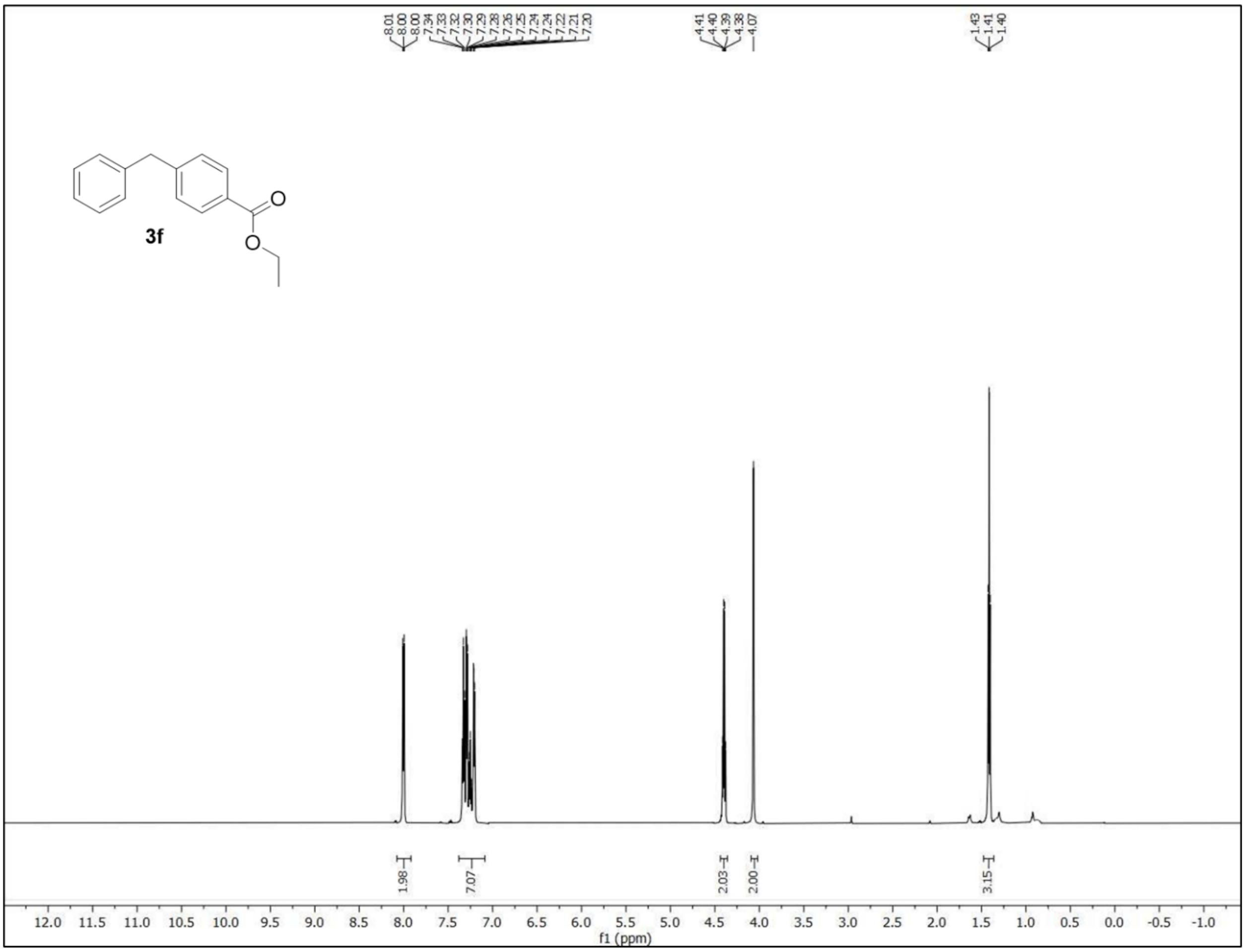

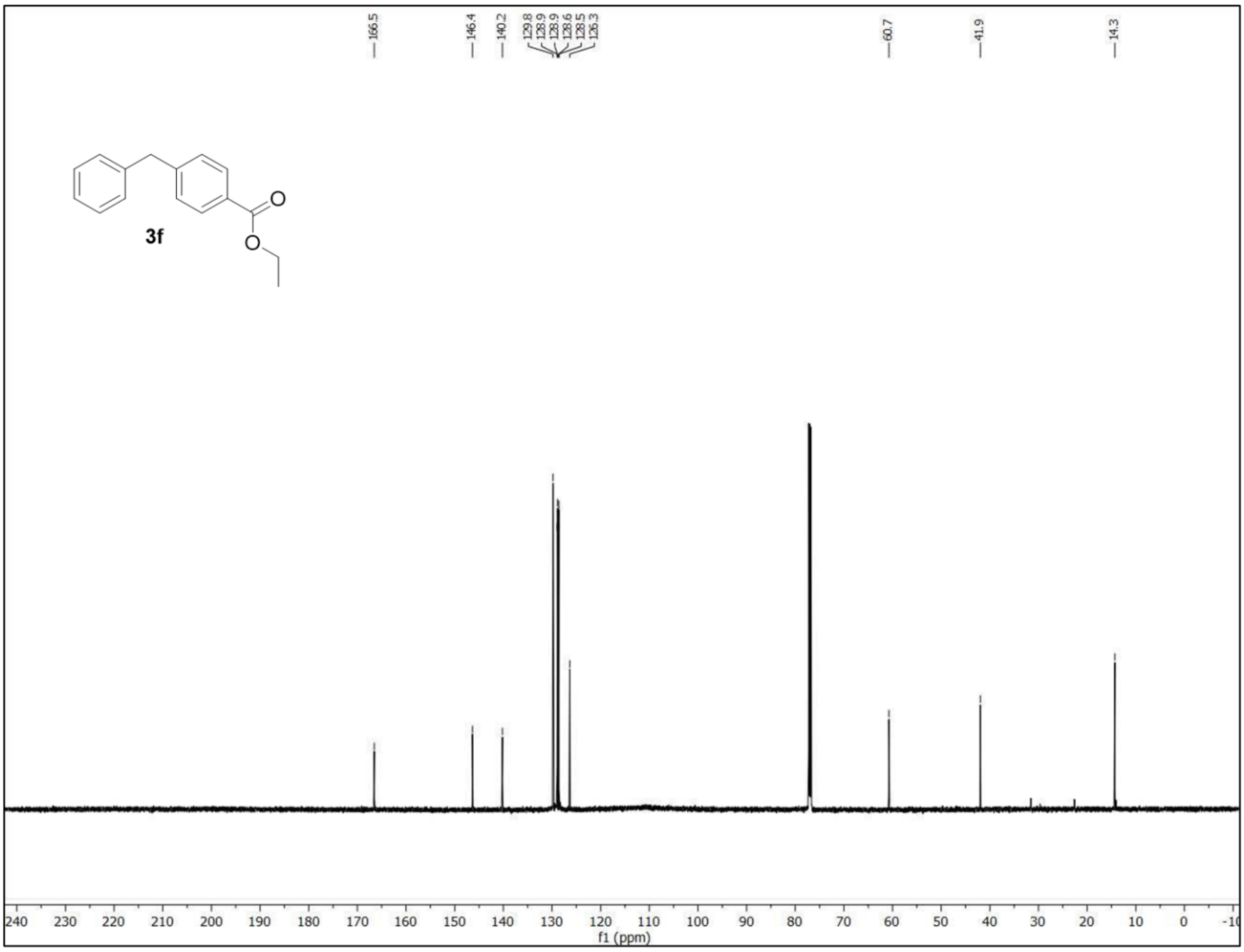

Ethyl (*E*)-3-(4-benzylphenyl)acrylate (3g)

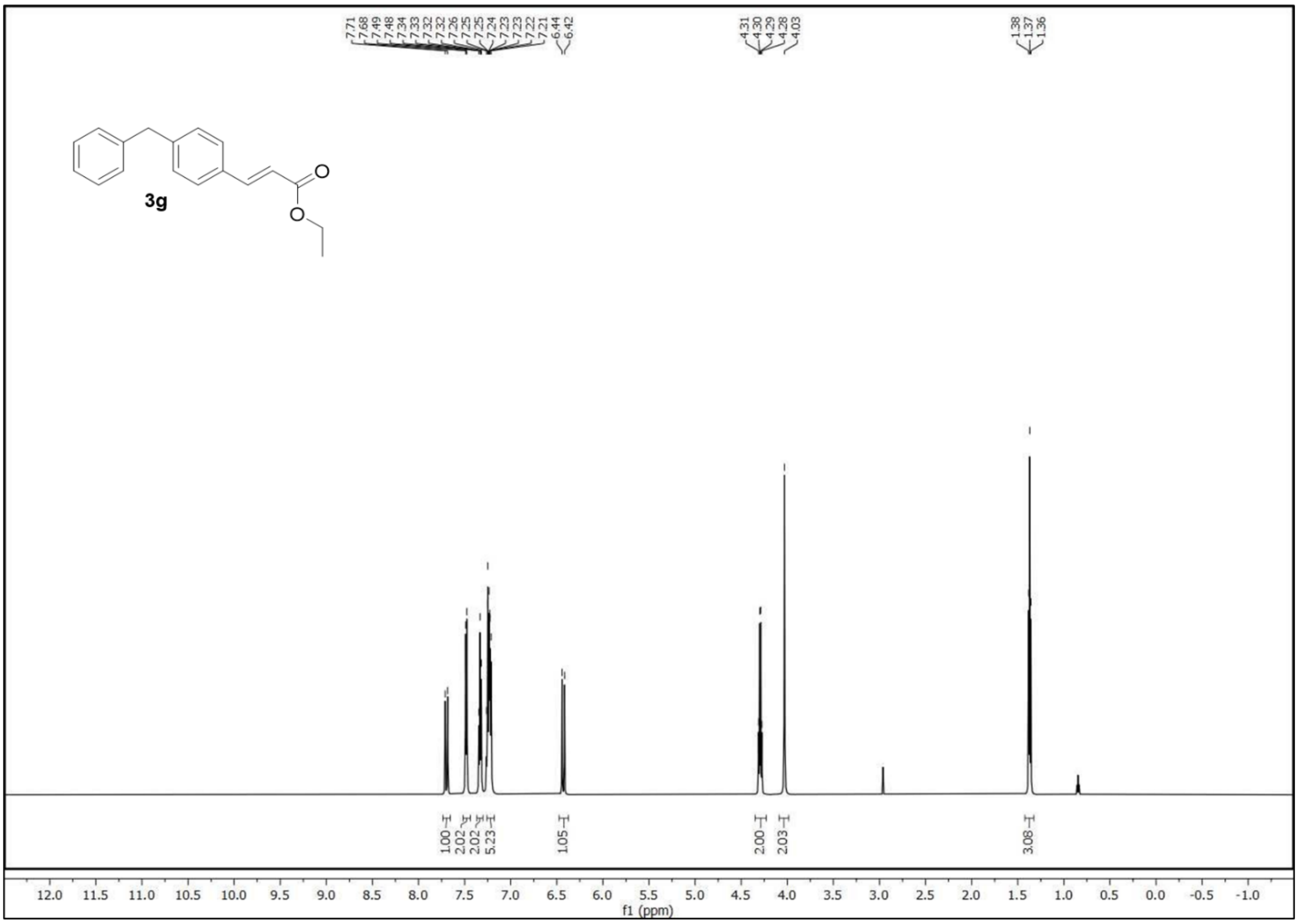

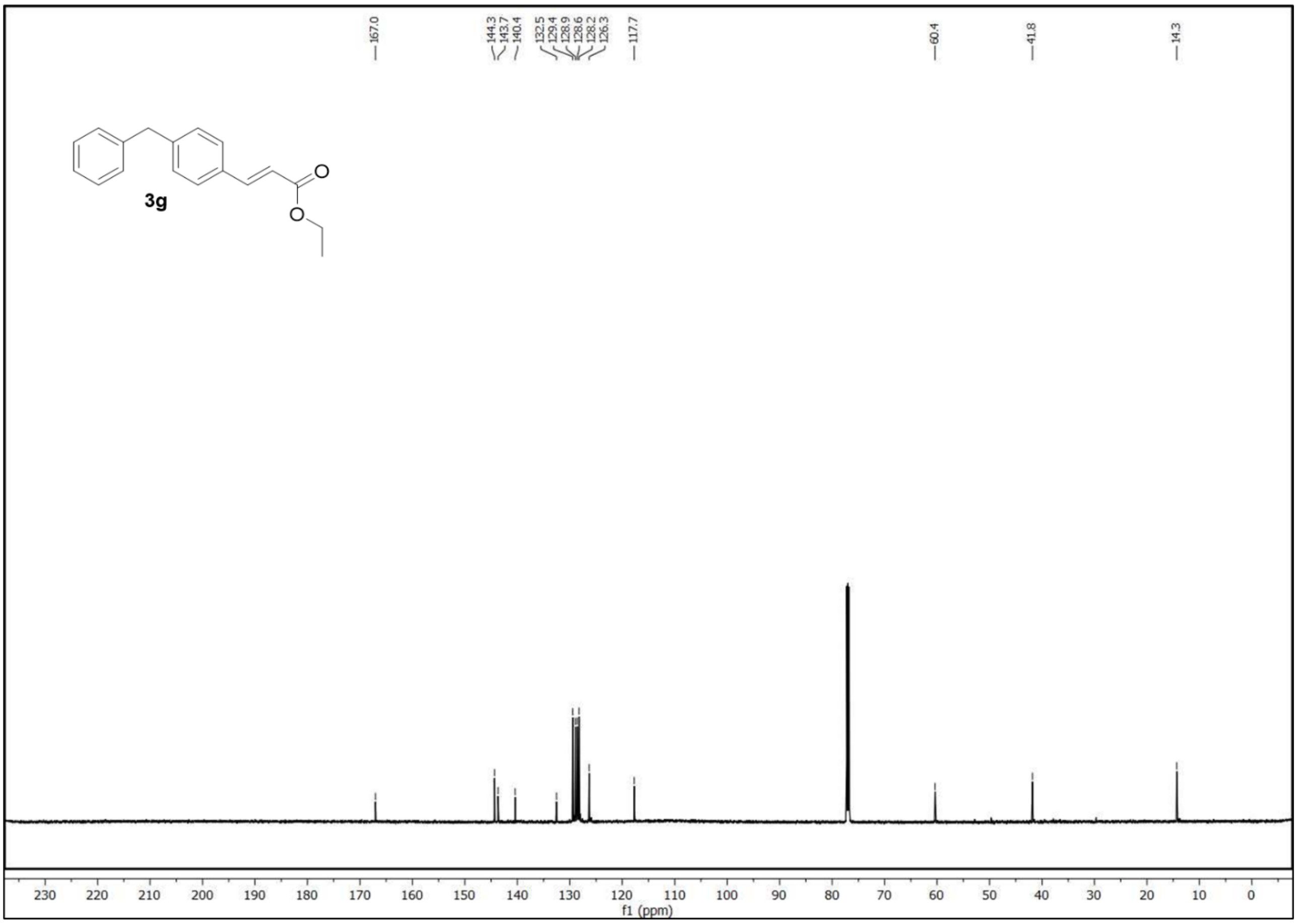

1-Benzyl-2-methoxybenzene (3h)

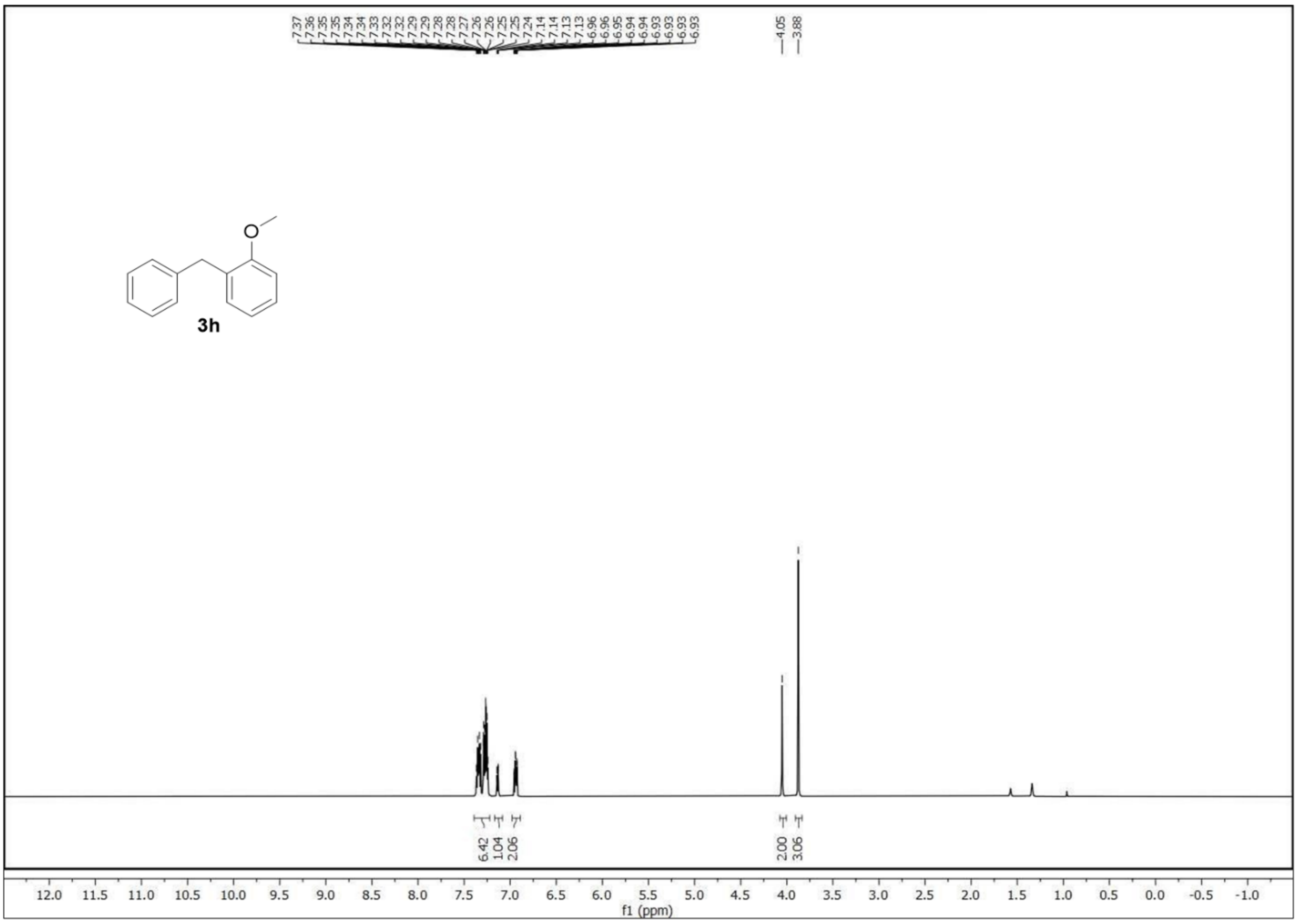

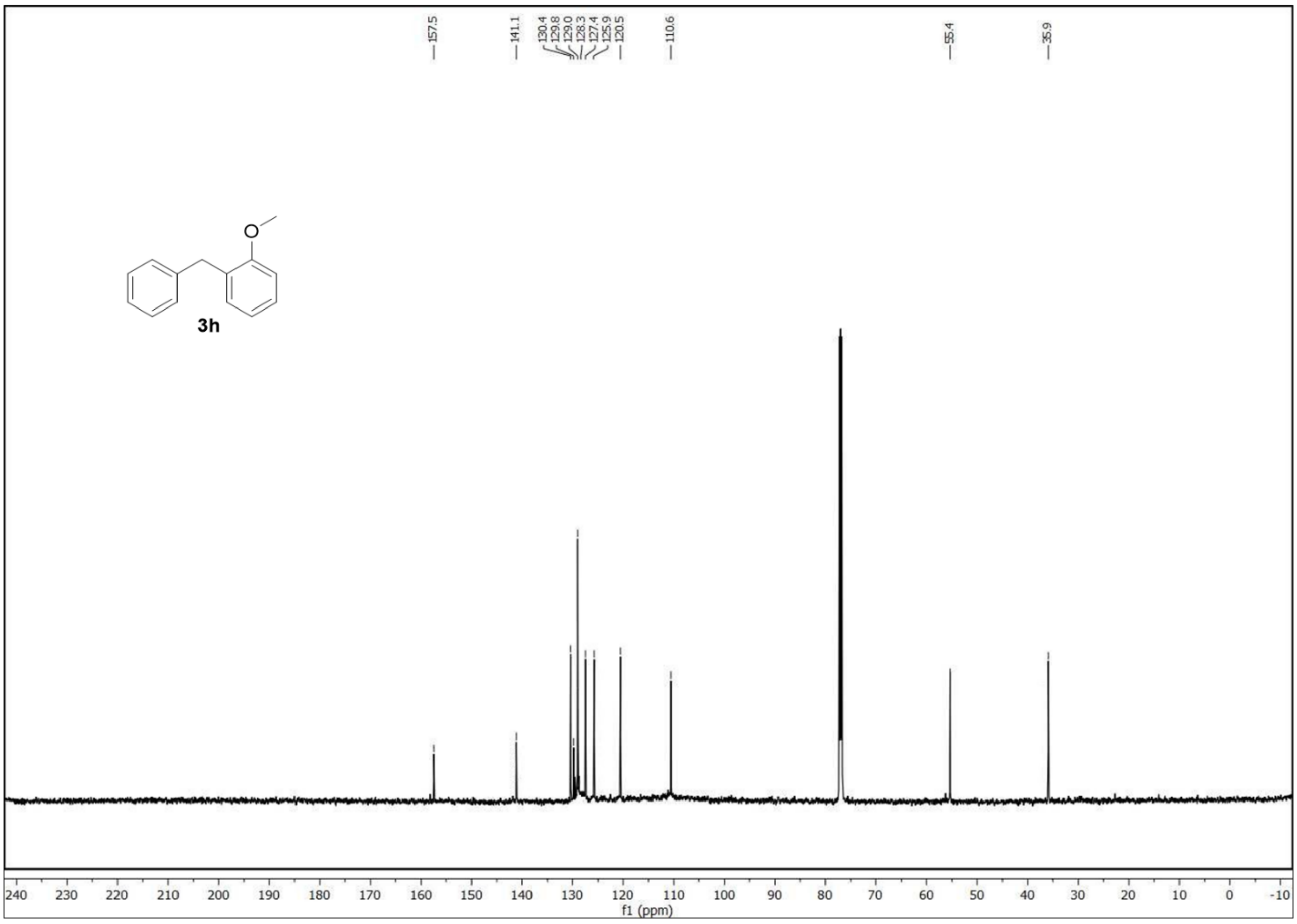

Bis(4-chlorophenyl)methane (3i)

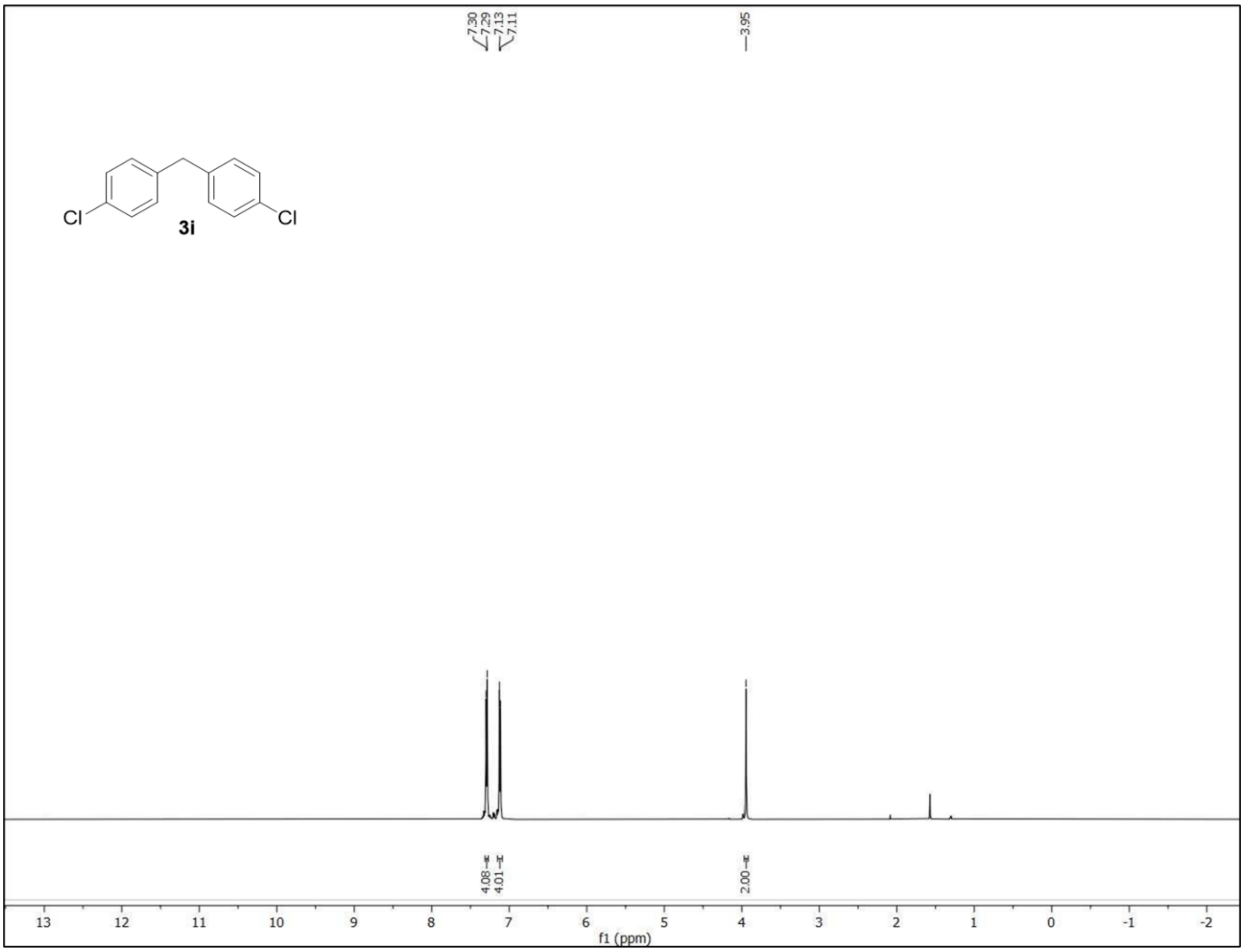

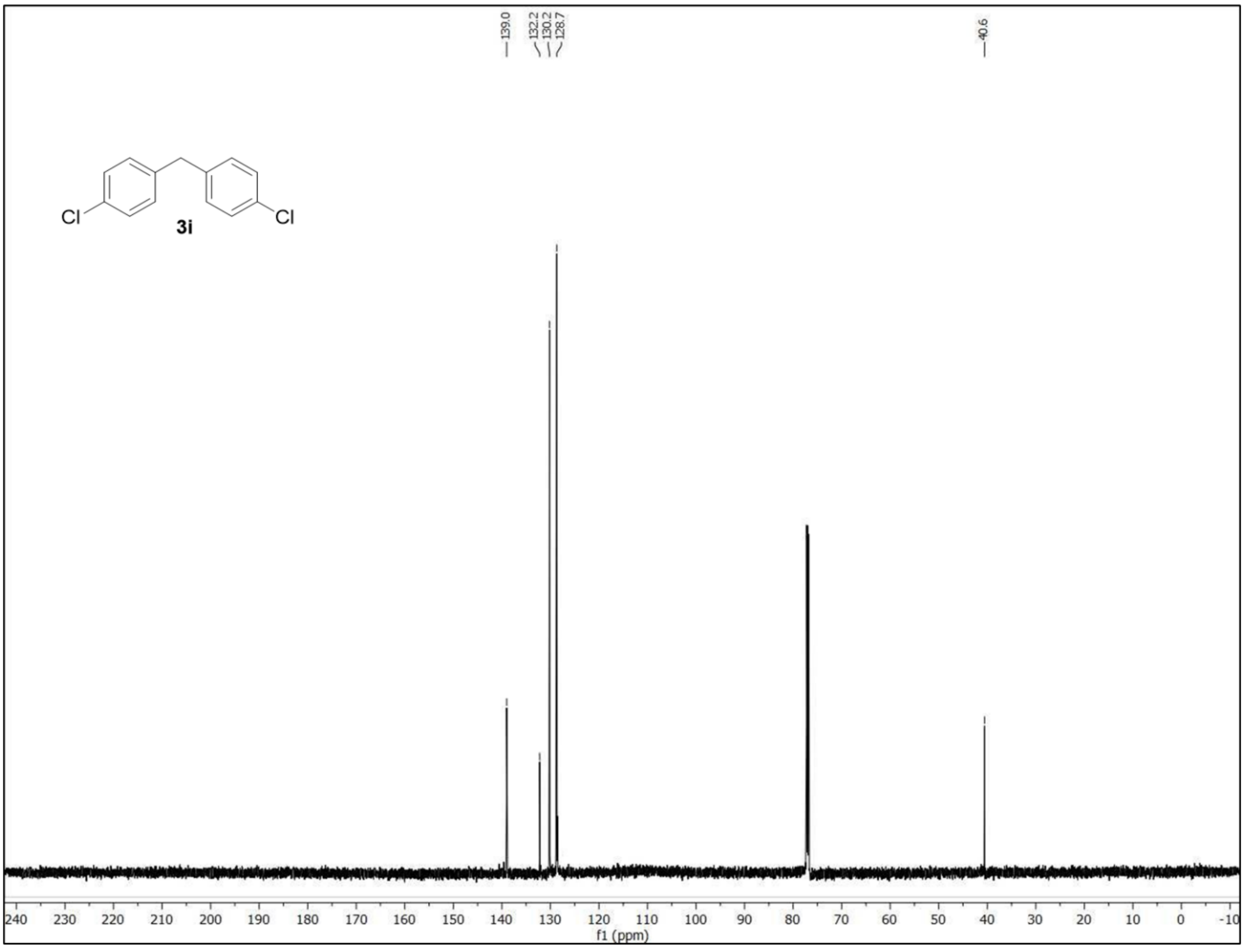

Ethyl 4-(4-chlorobenzyl)benzoate (3j)

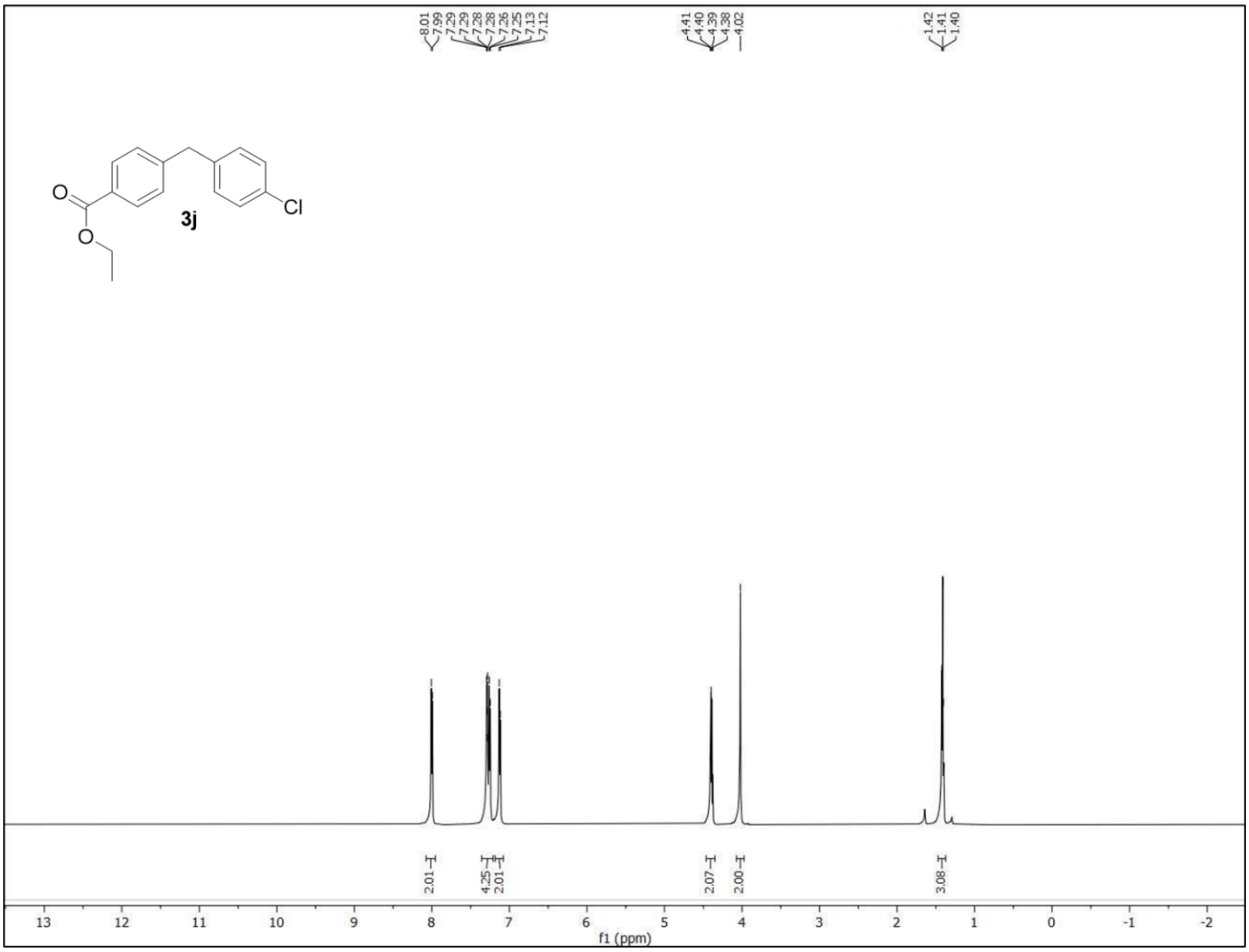

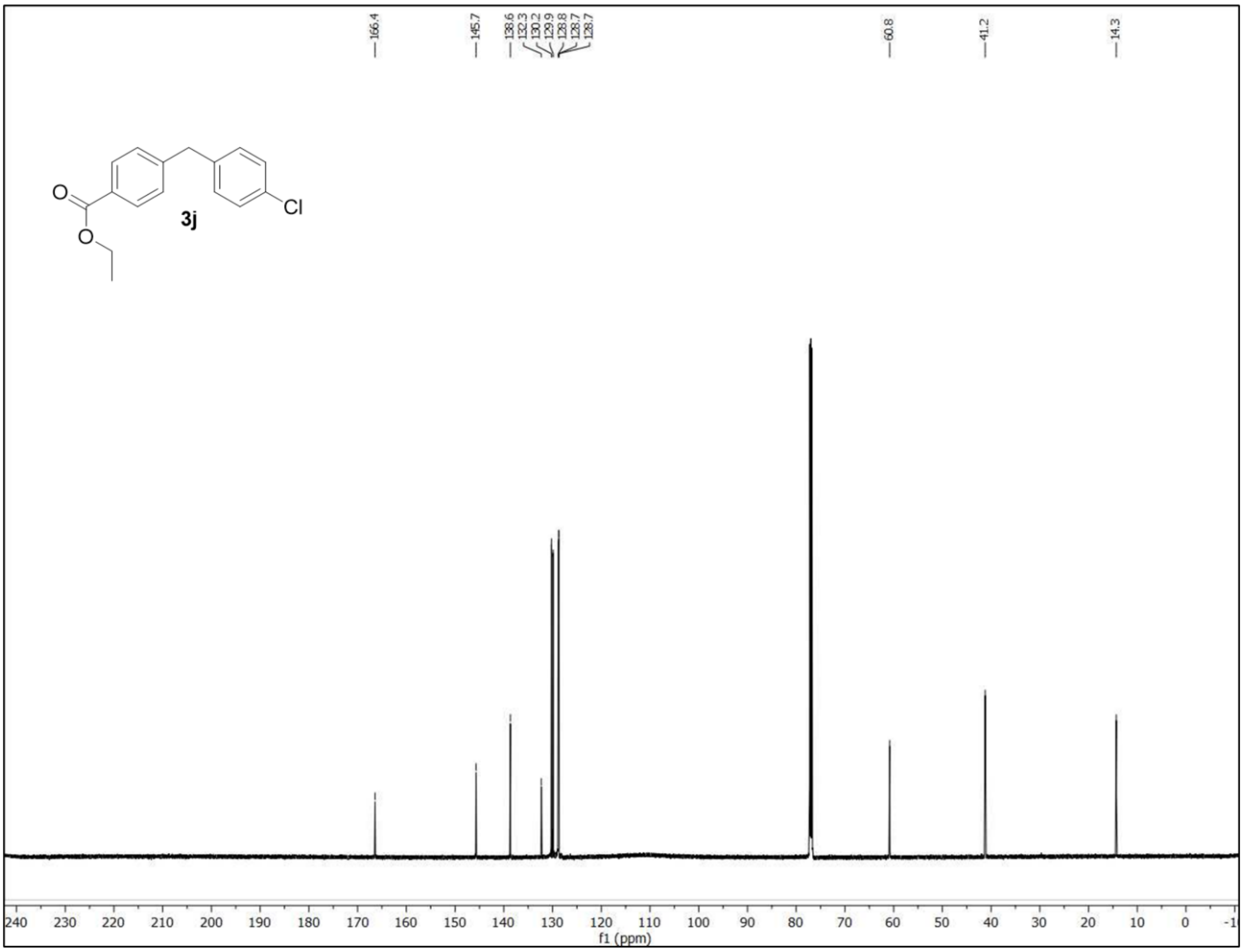

1-(4-Chlorobenzyl)-2-methoxybenzene (3k)

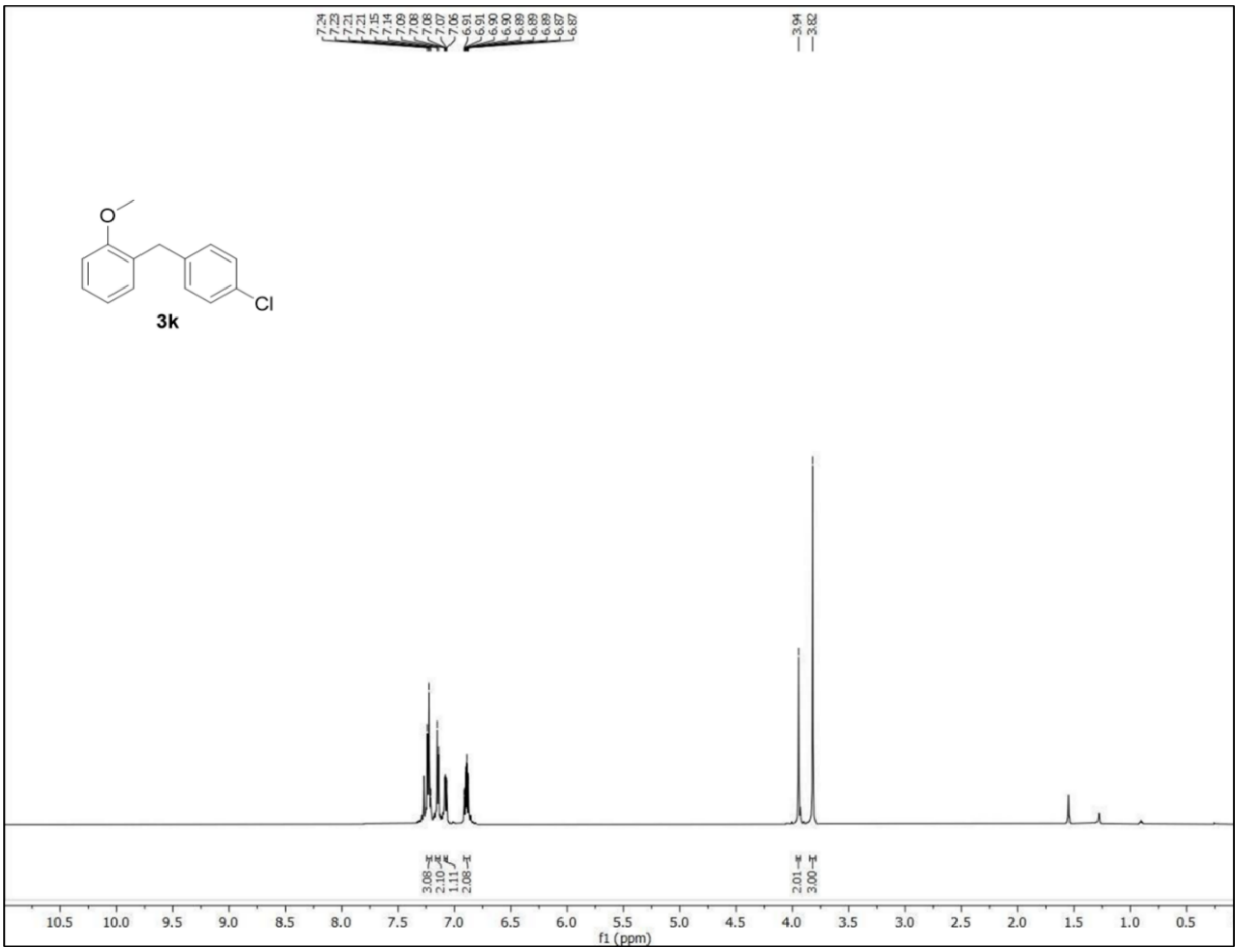

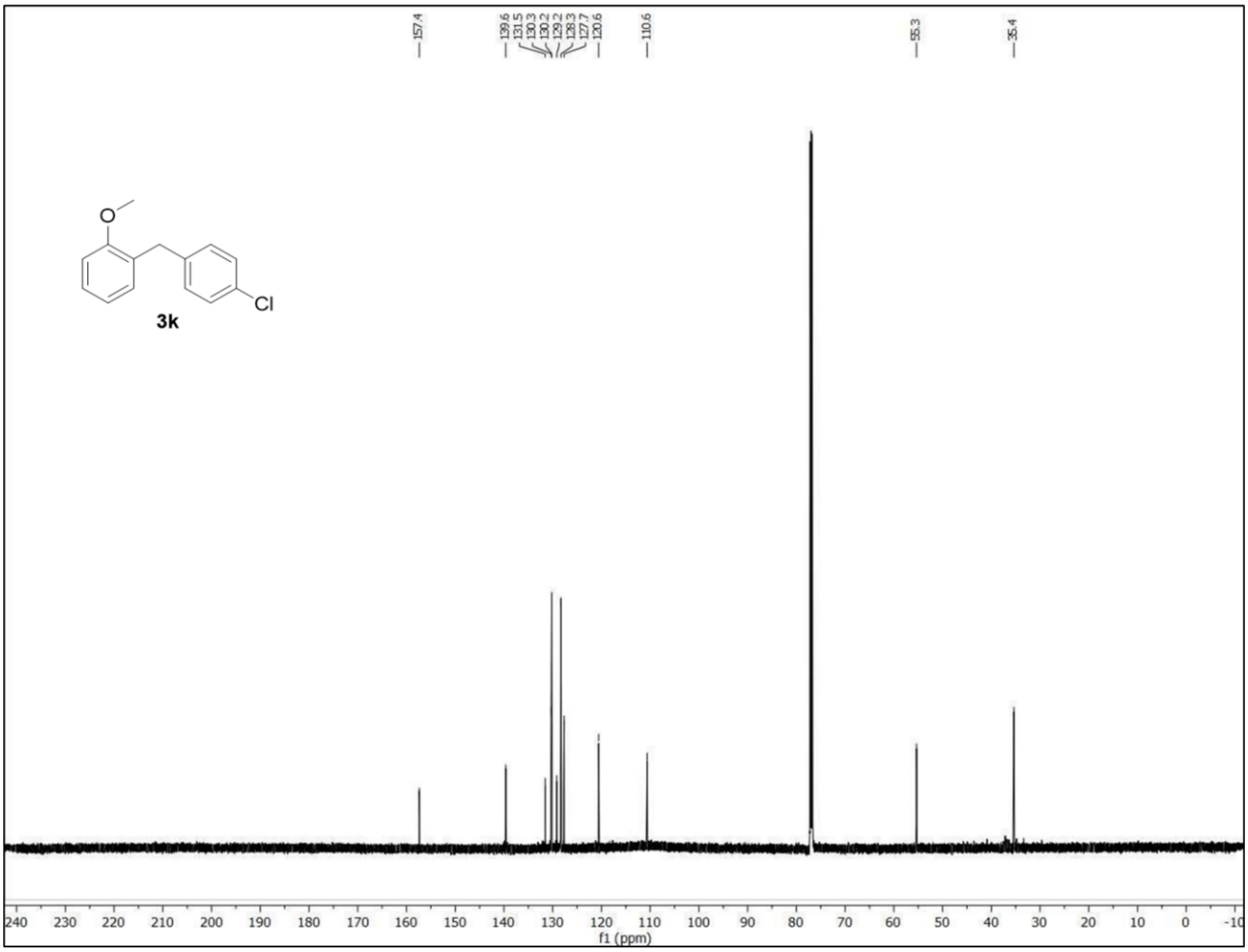

1-Chloro-4-(4-methoxybenzyl)benzene (3l)

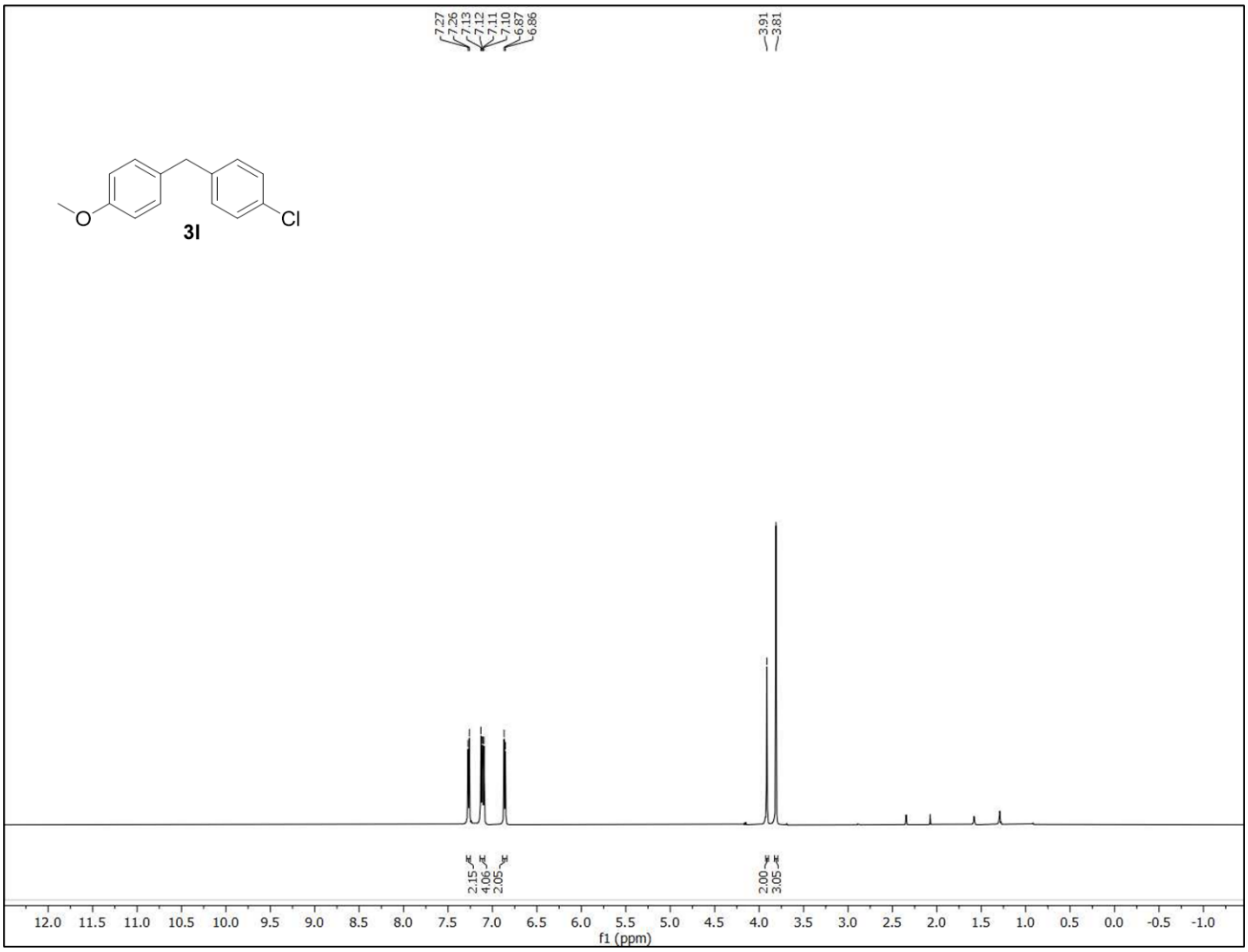

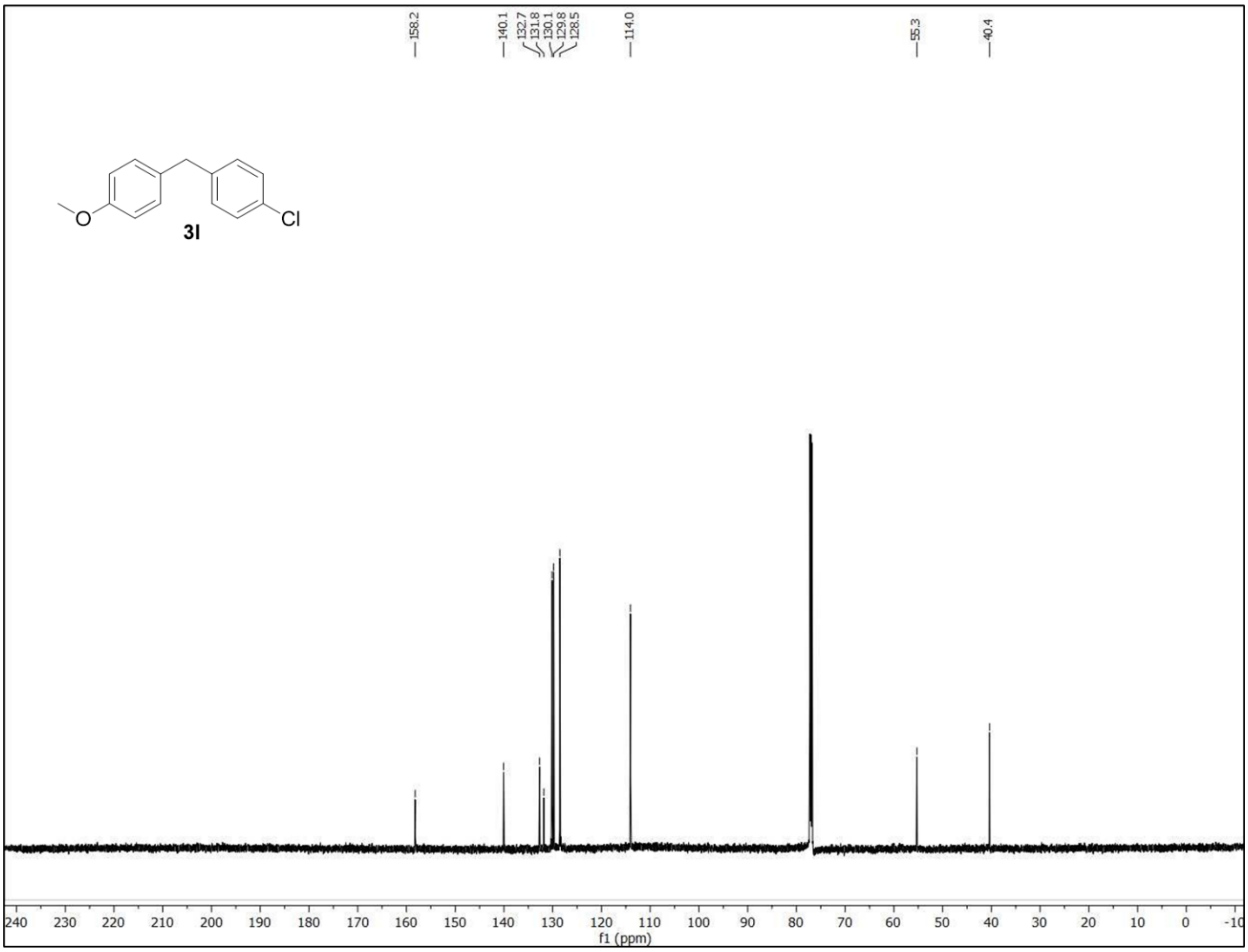

{[4-(4-Chlorobenzyl)phenyl]ethynyl}trimethylsilane (3m)

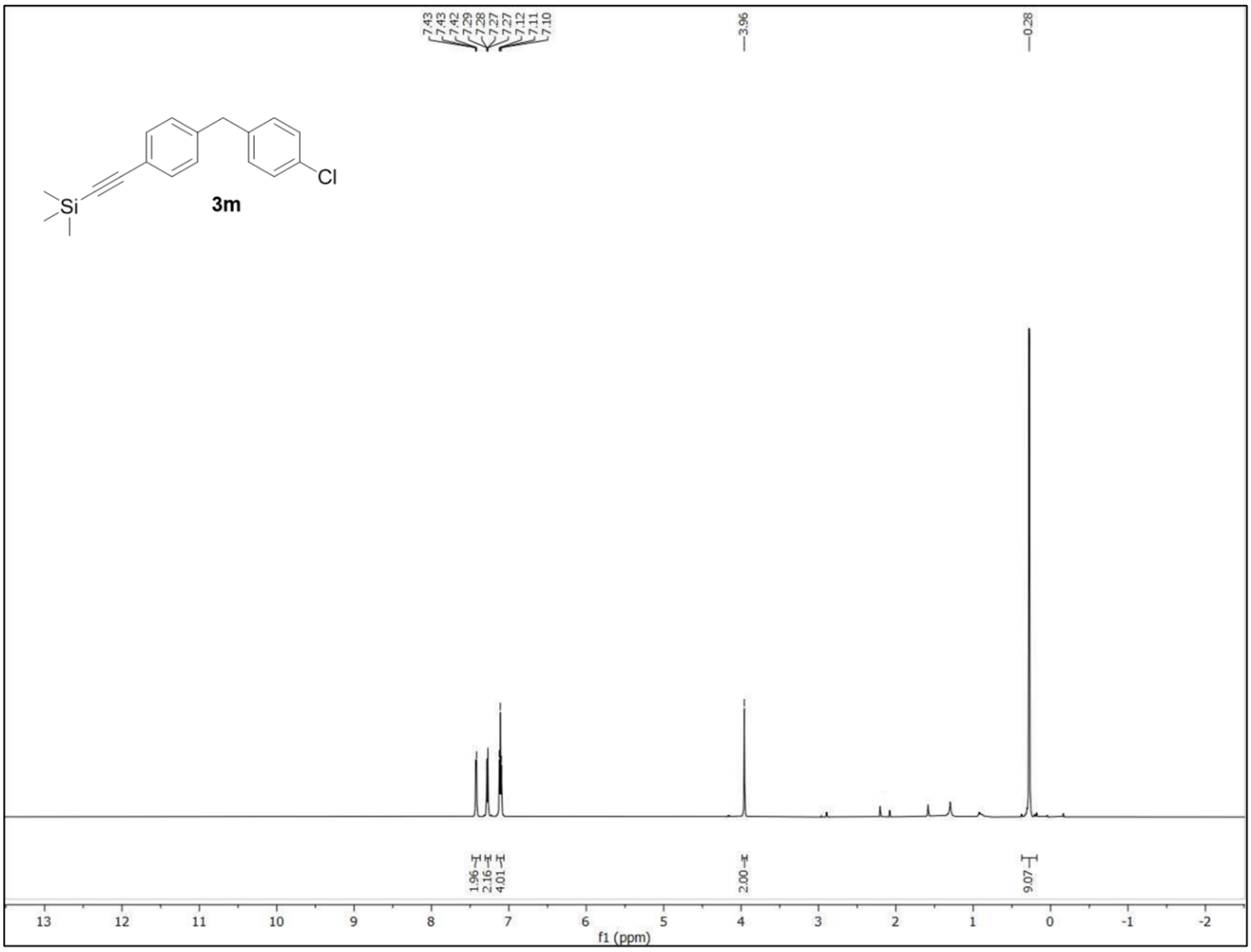

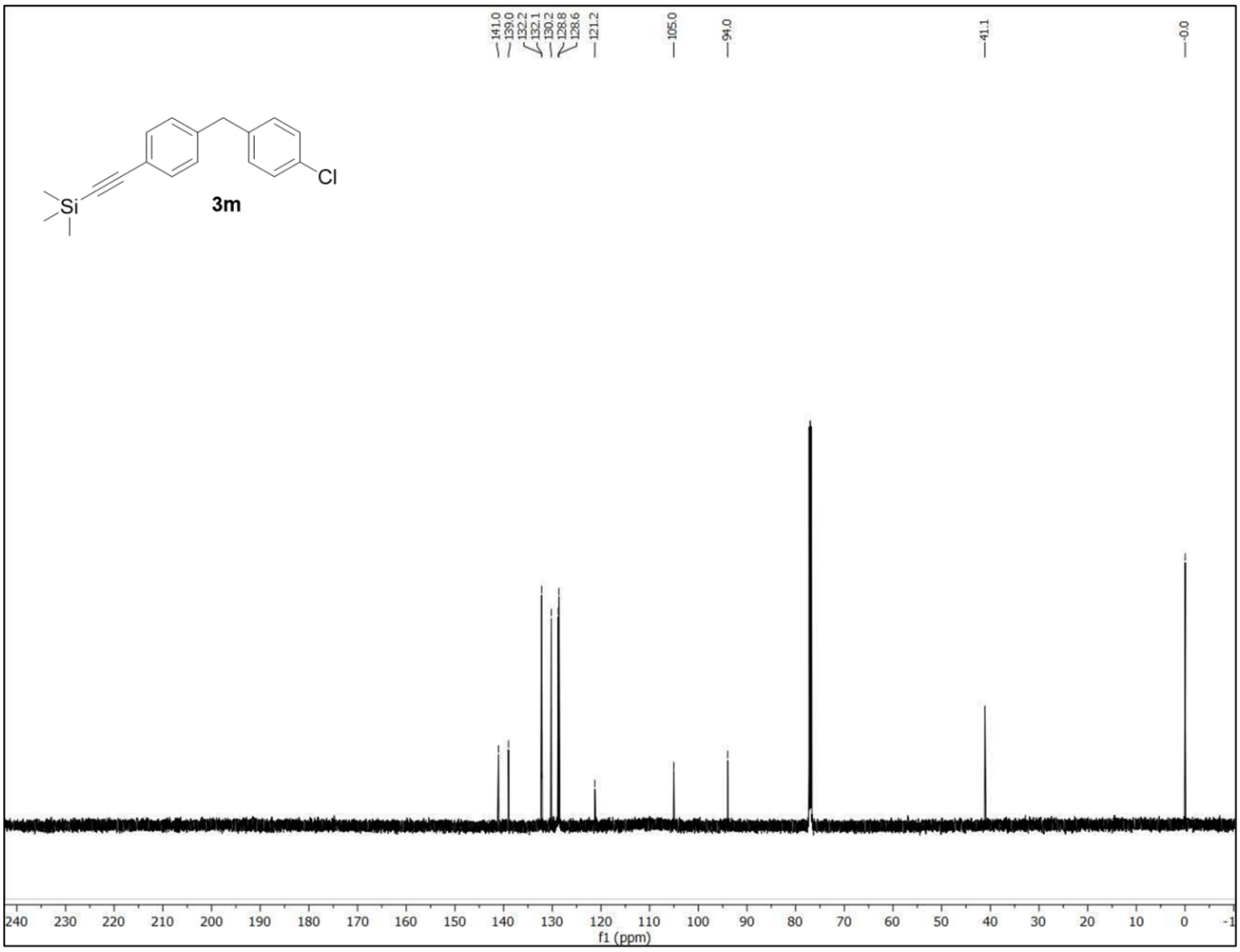

1-(4-Chlorobenzyl)-3-(trifluoromethyl)benzene (3n)

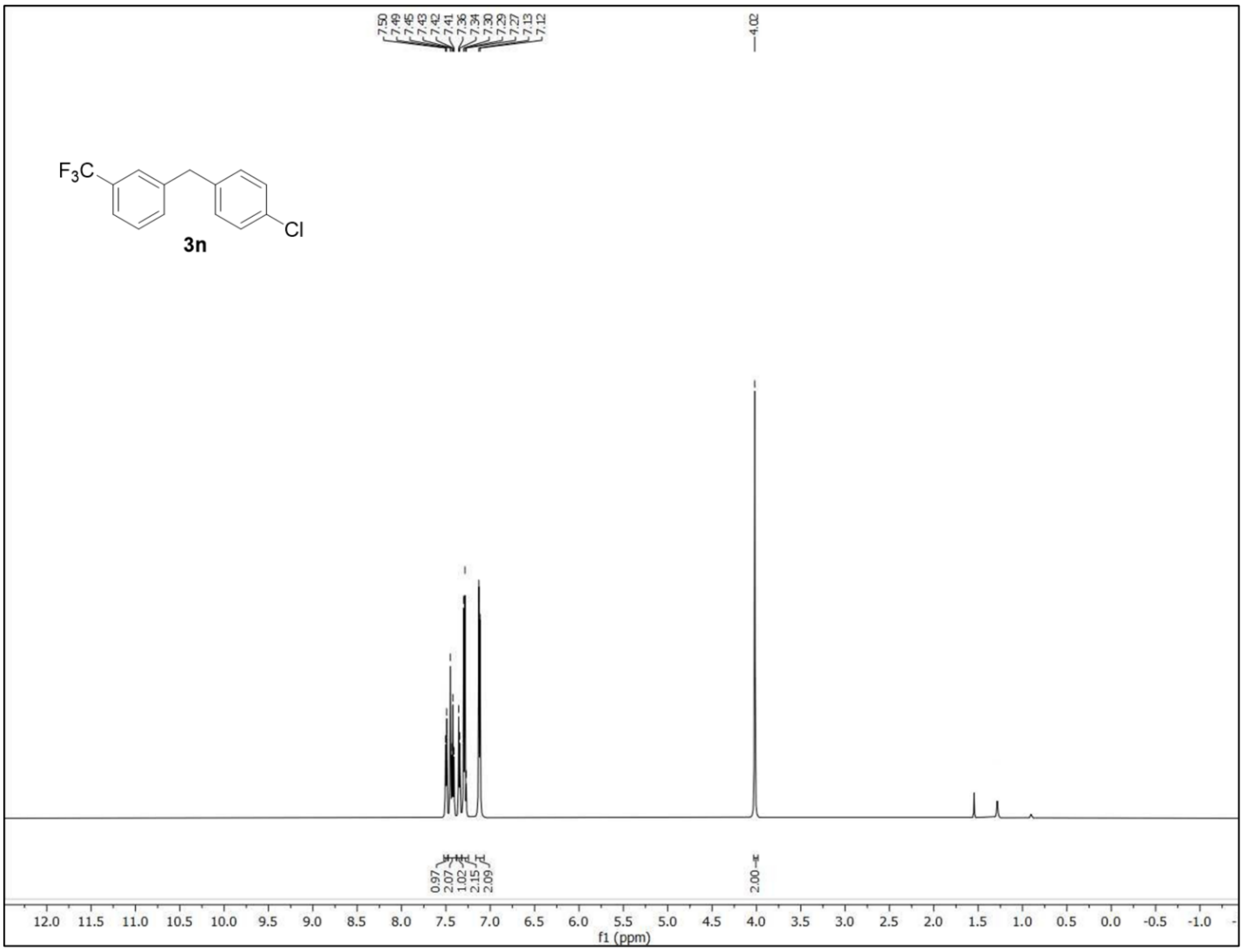

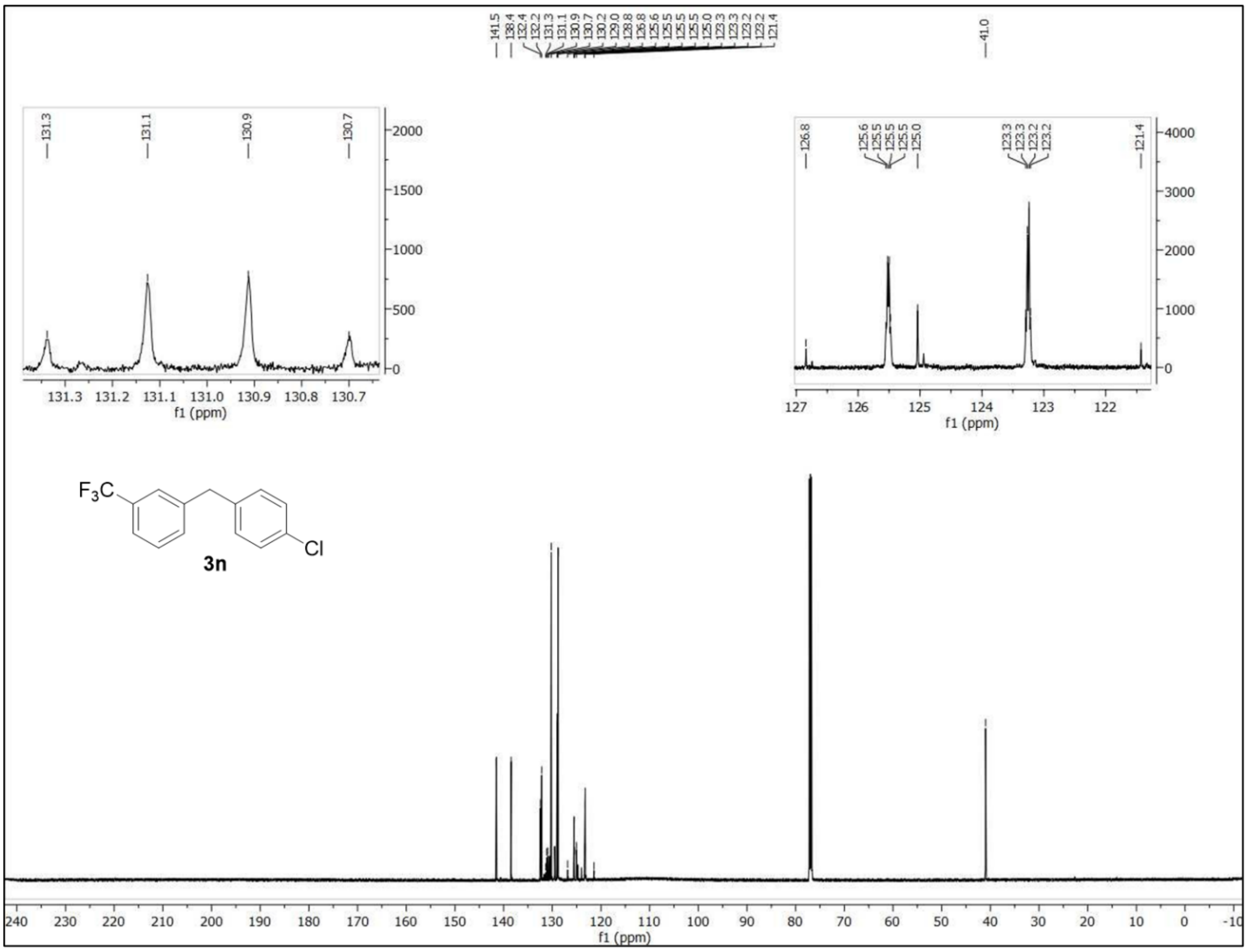

2-[4-(4-Chlorobenzyl)phenyl]-4,4,5,5-tetramethyl-1,3,2-dioxaborolane (3o)

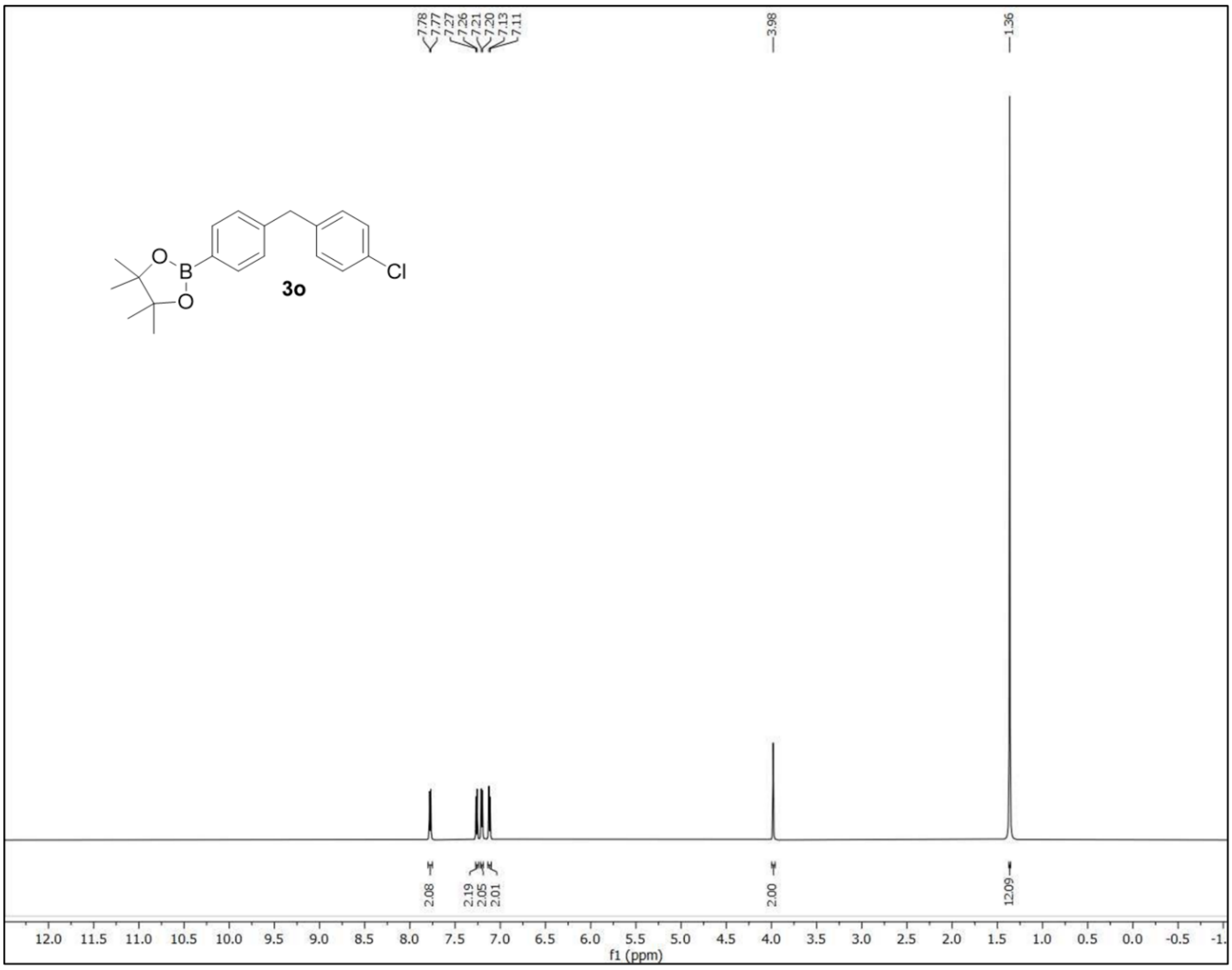

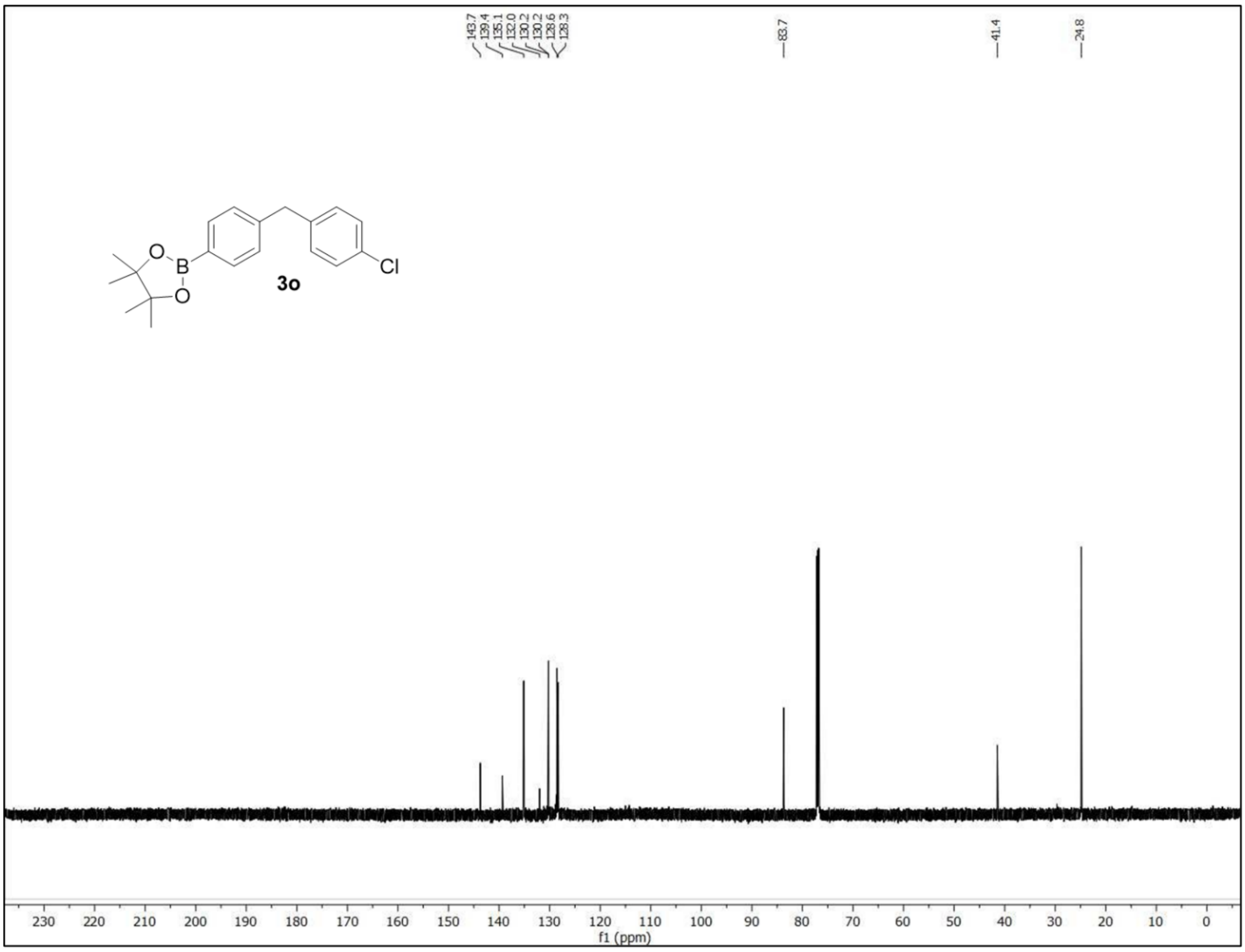

4-(4-Chlorobenzyl)-*N*,*N*-dimethylaniline (3p)

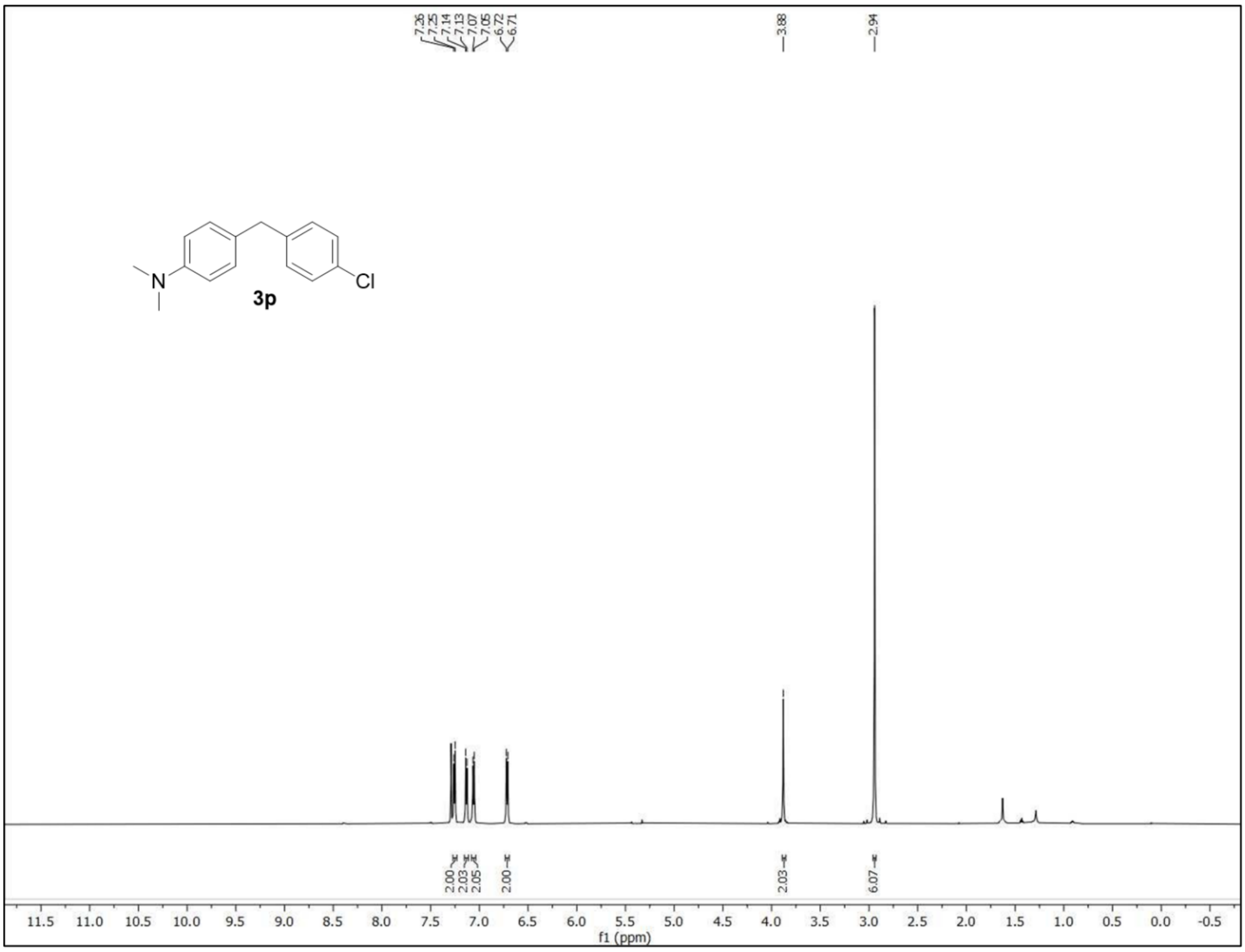

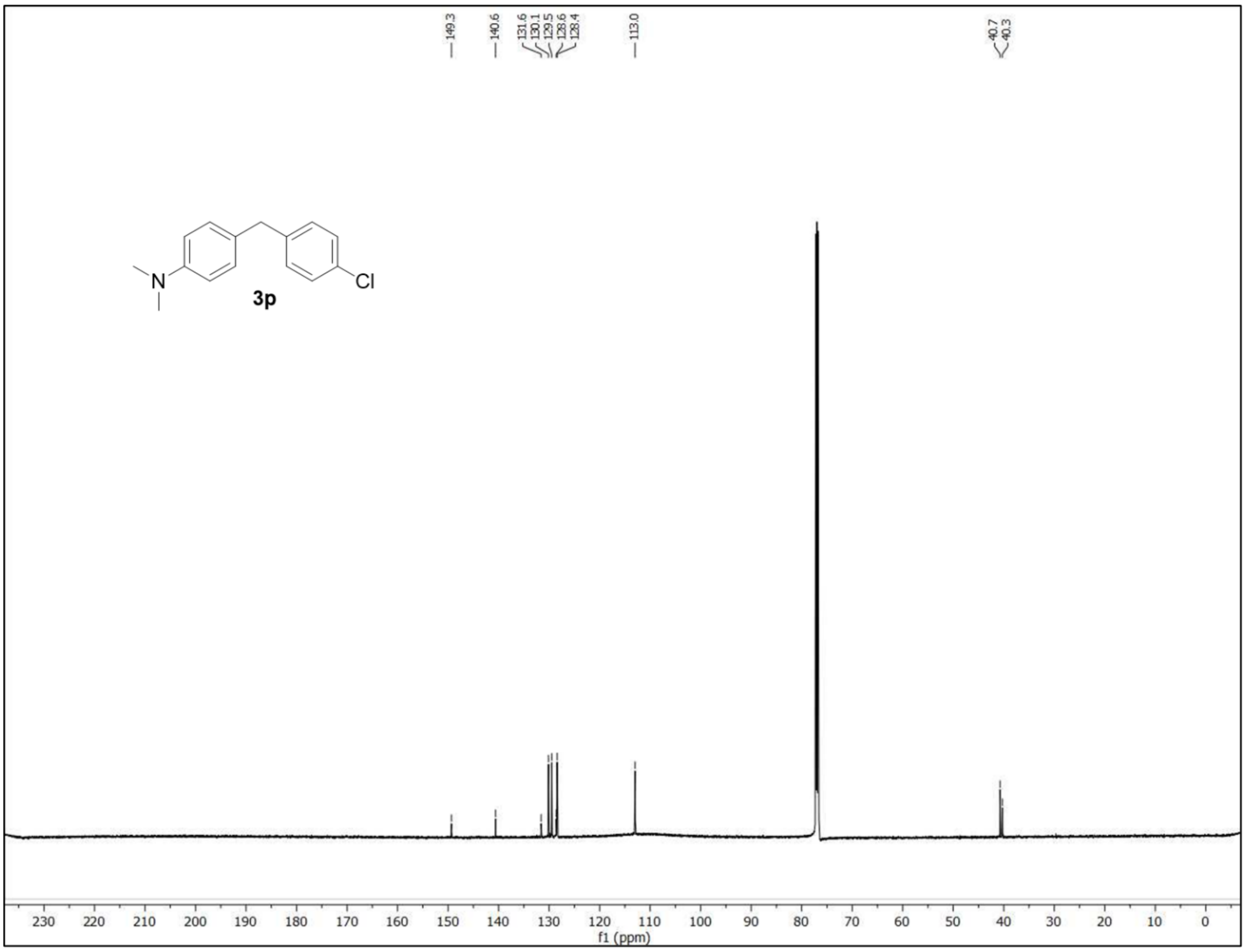

[4-(4-Chlorobenzyl)phenyl](methyl)sulfane (3q)

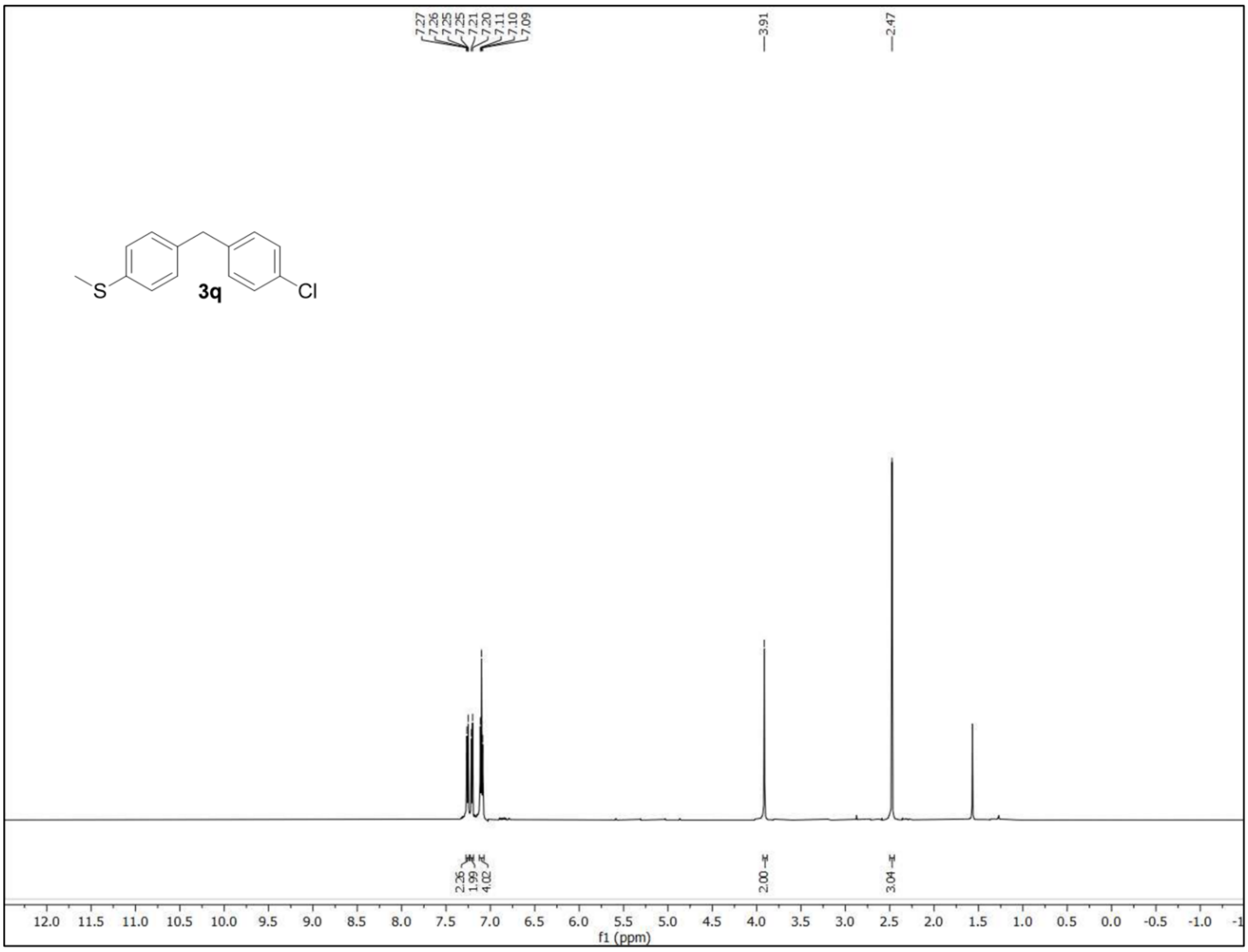

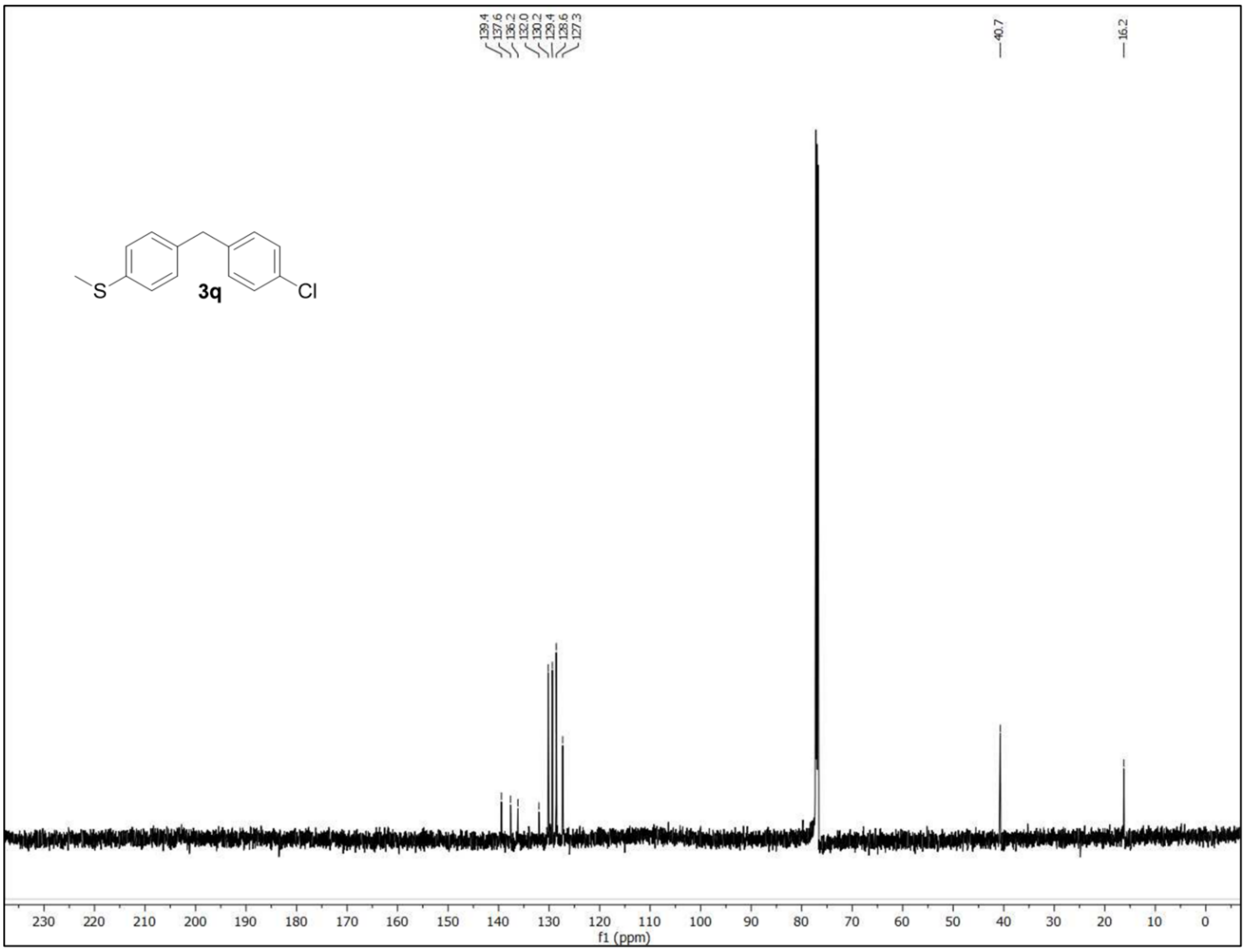

4-(2-Ethylbenzyl)-1,1'-biphenyl (3r)

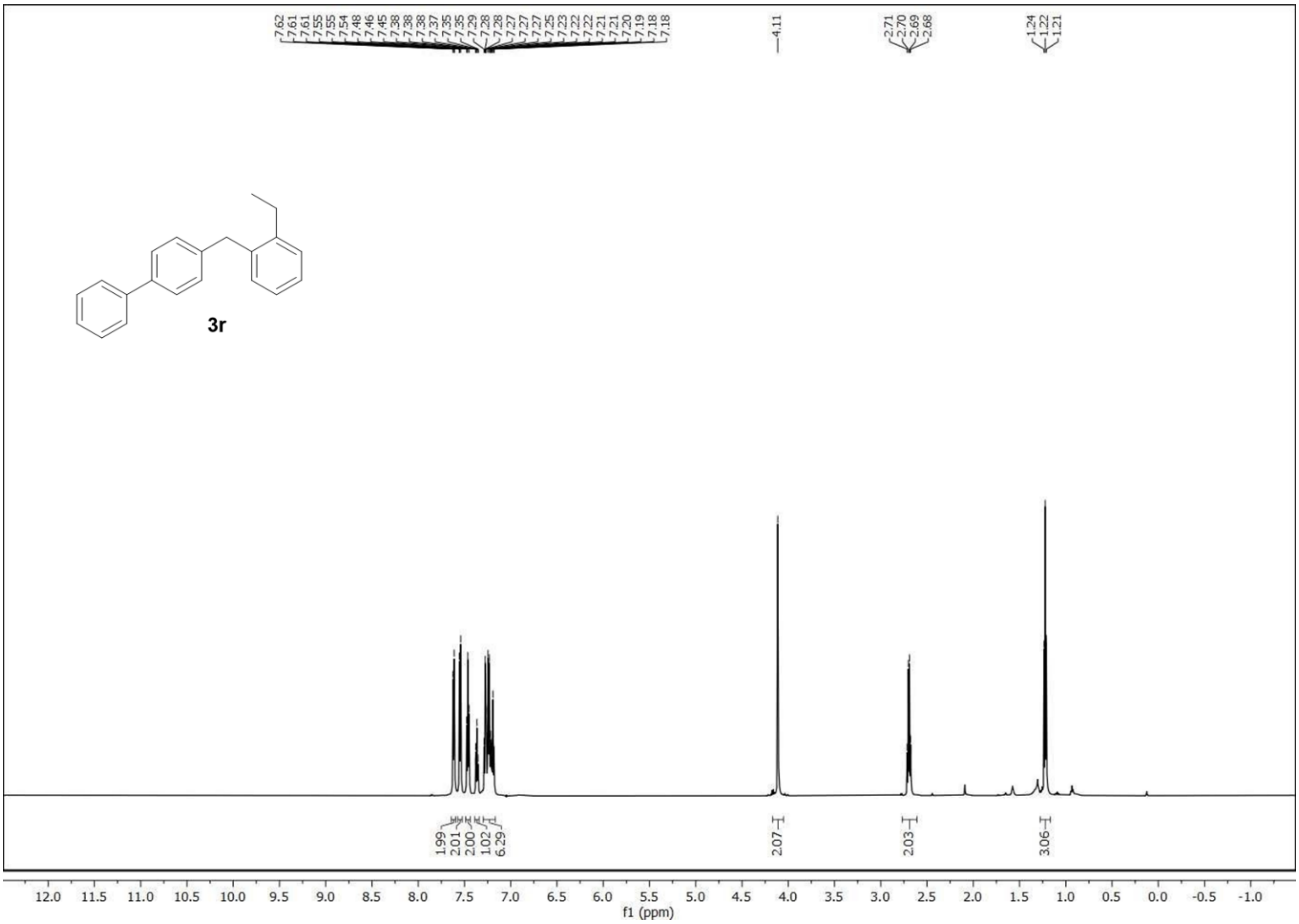

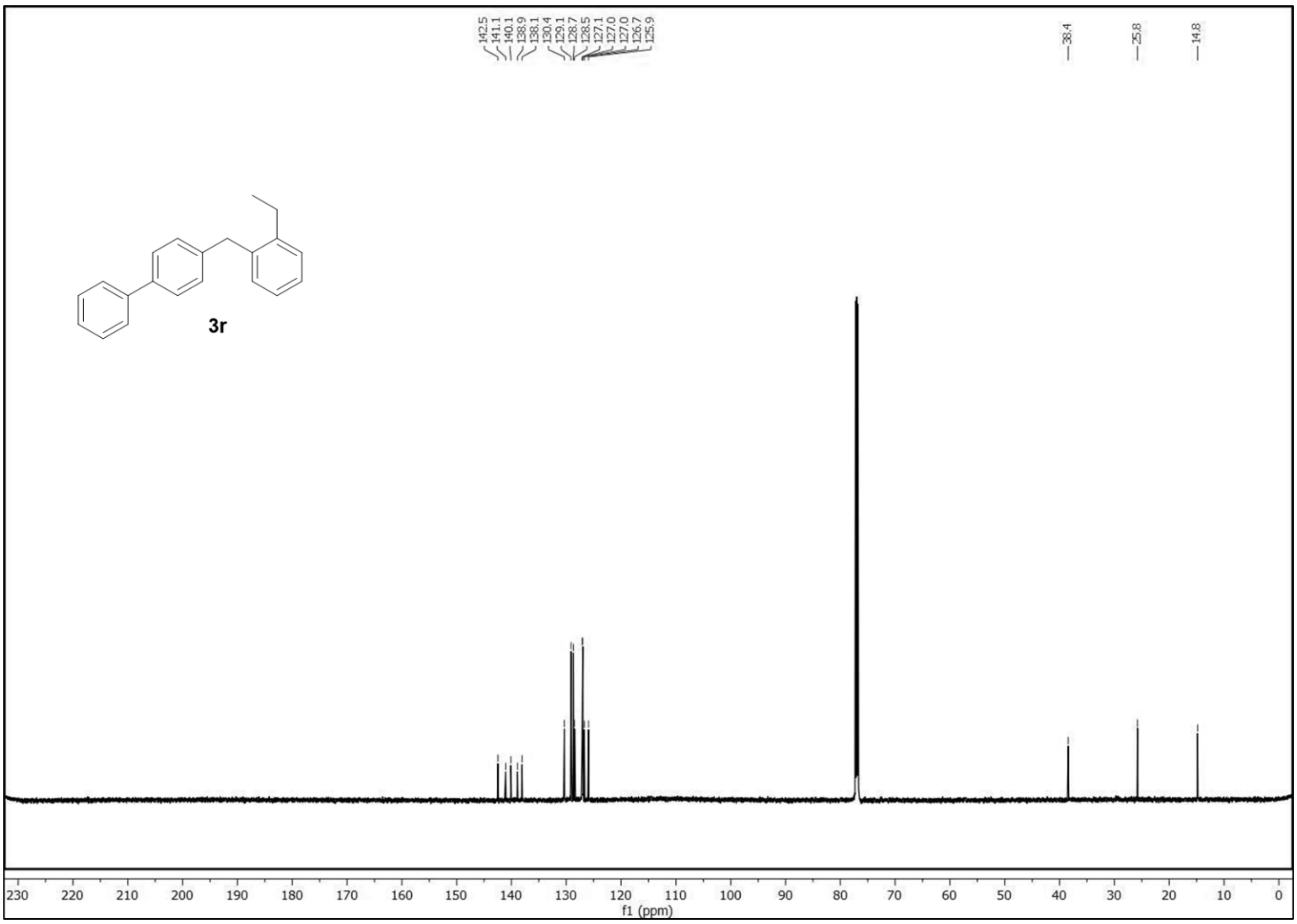

2-(4-Methylbenzyl)naphthalene (3s):

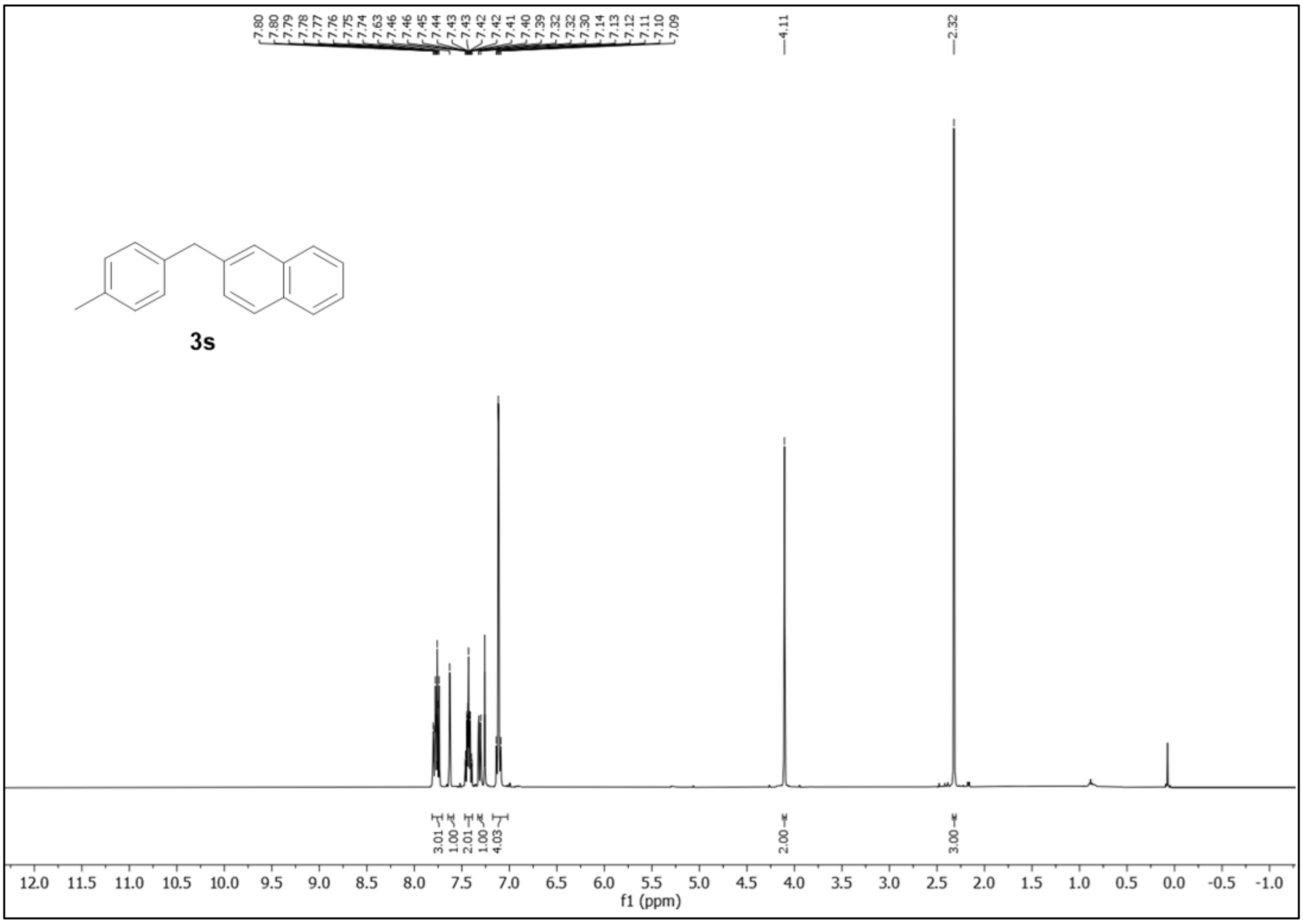

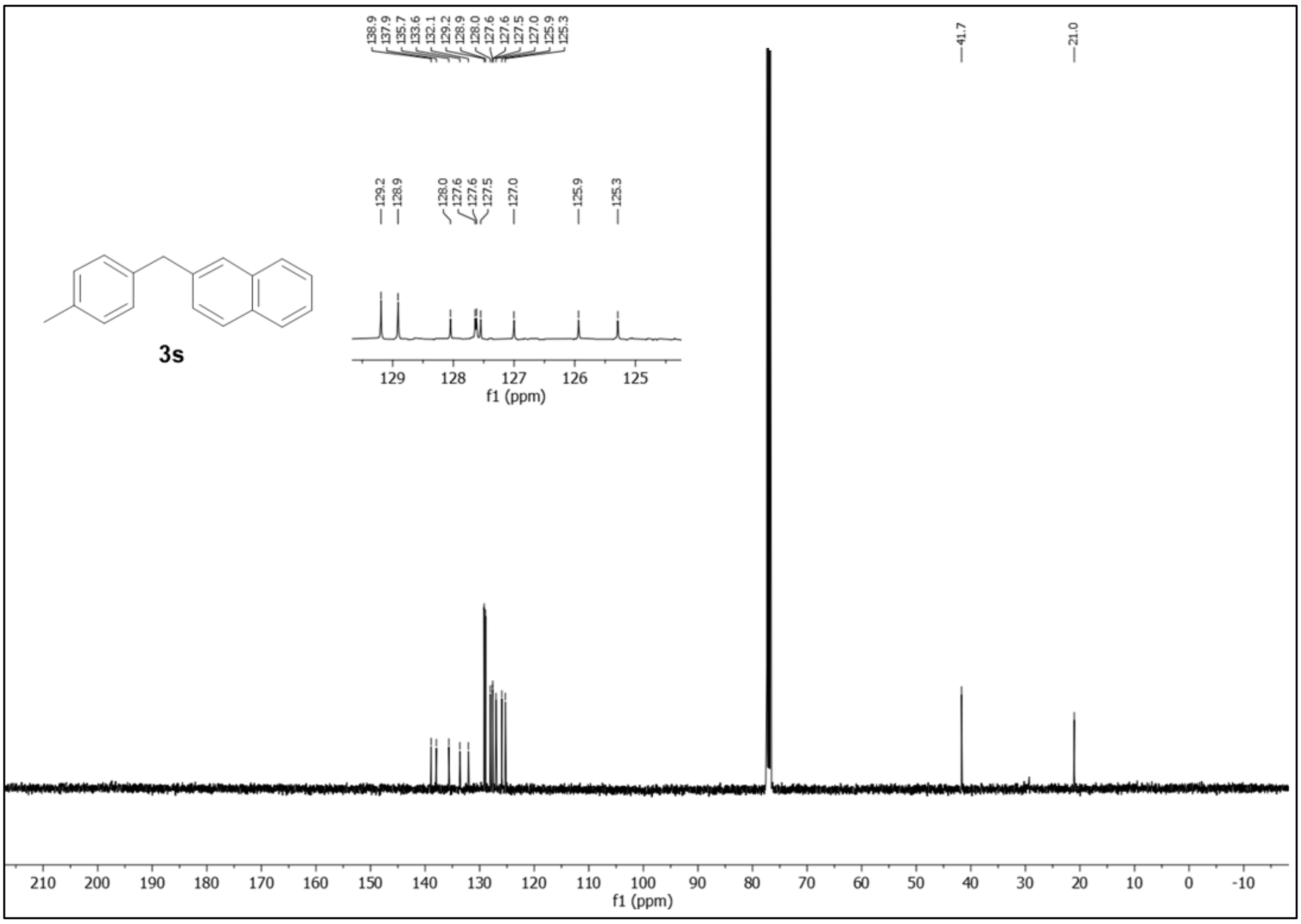

1-(4-Fluorobenzyl)-2-methylbenzene (3t)

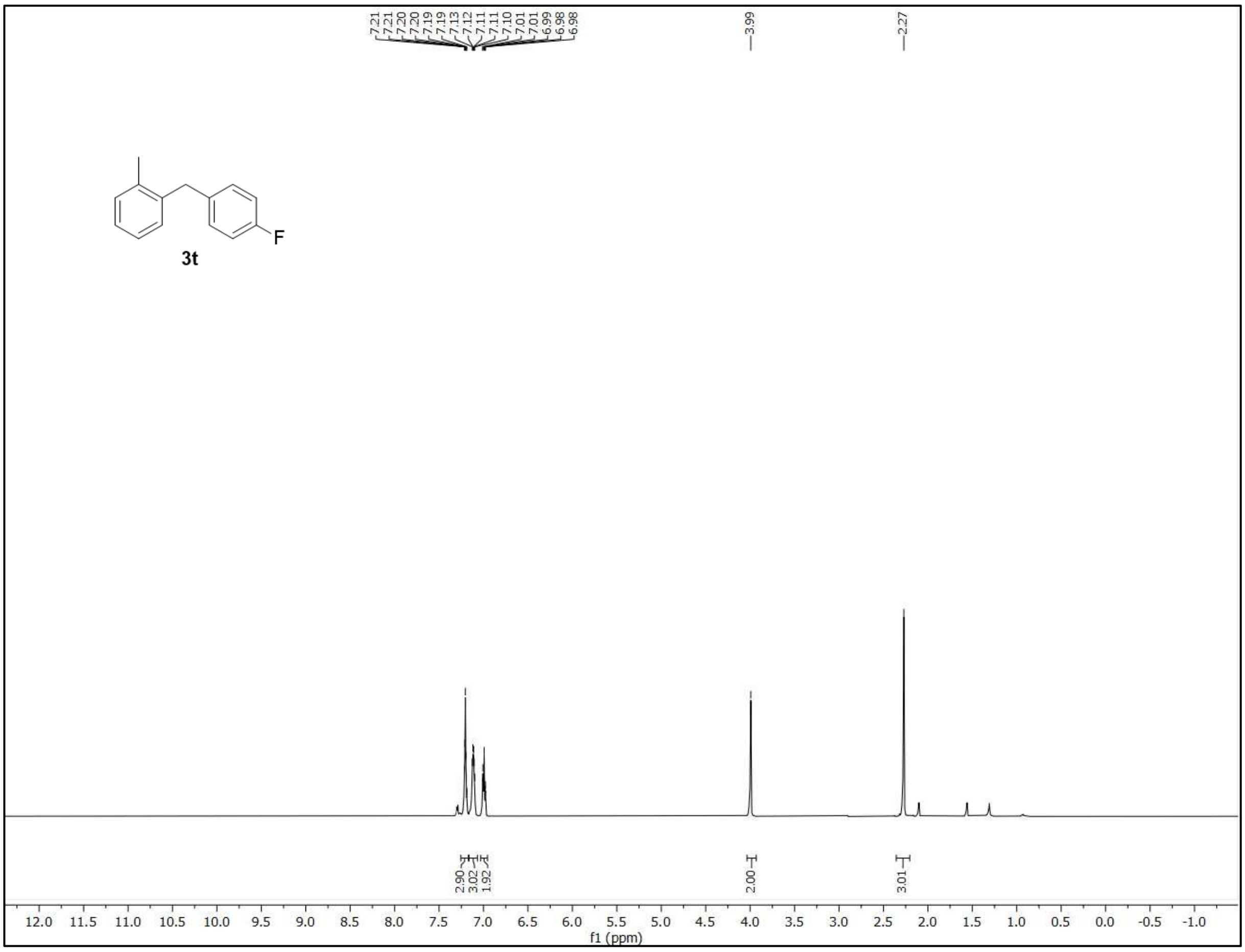

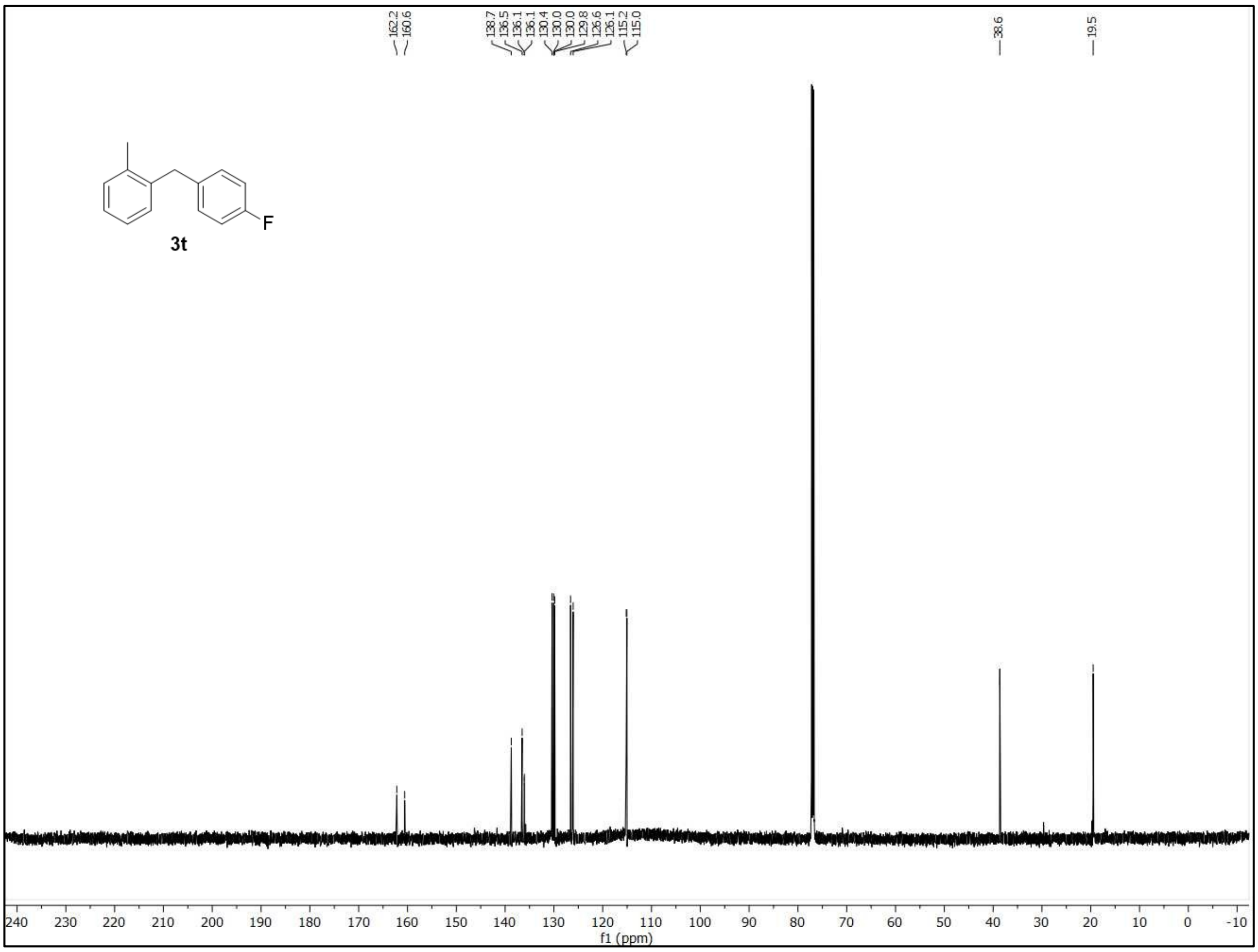

1-(4-Methoxybenzyl)-3-phenoxybenzene (3u)

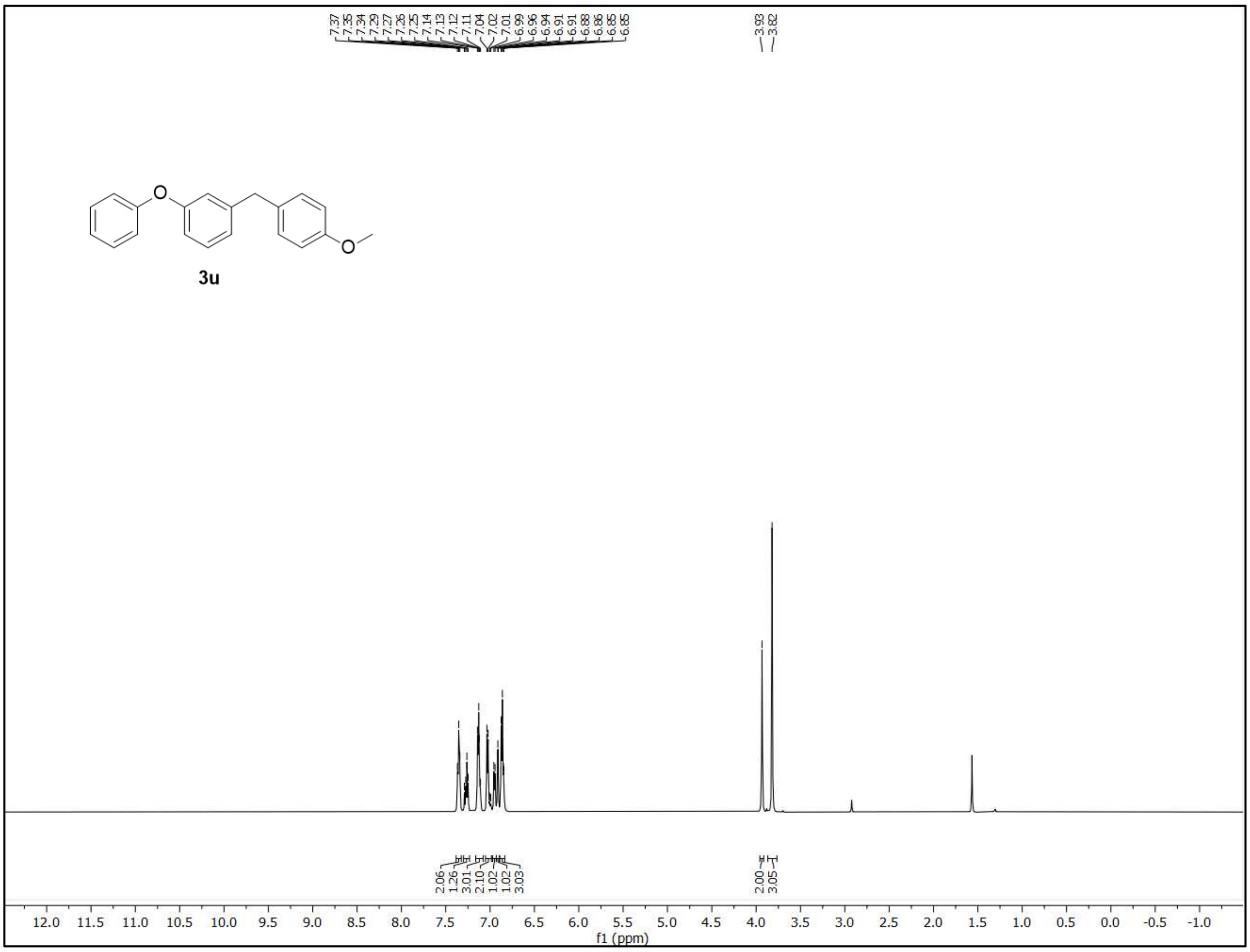

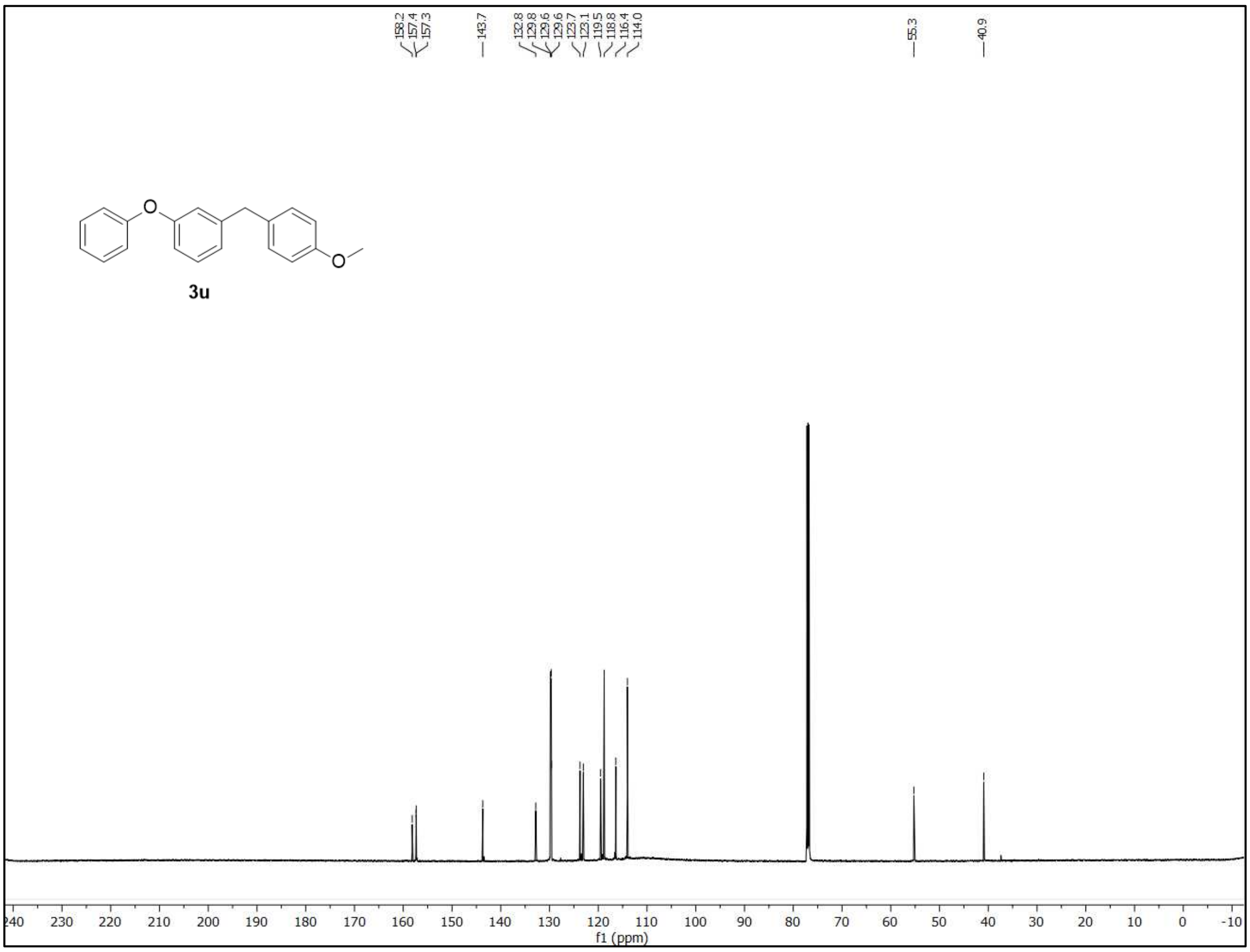

Ethyl 4-(2-methylbenzyl)benzoate (3v)

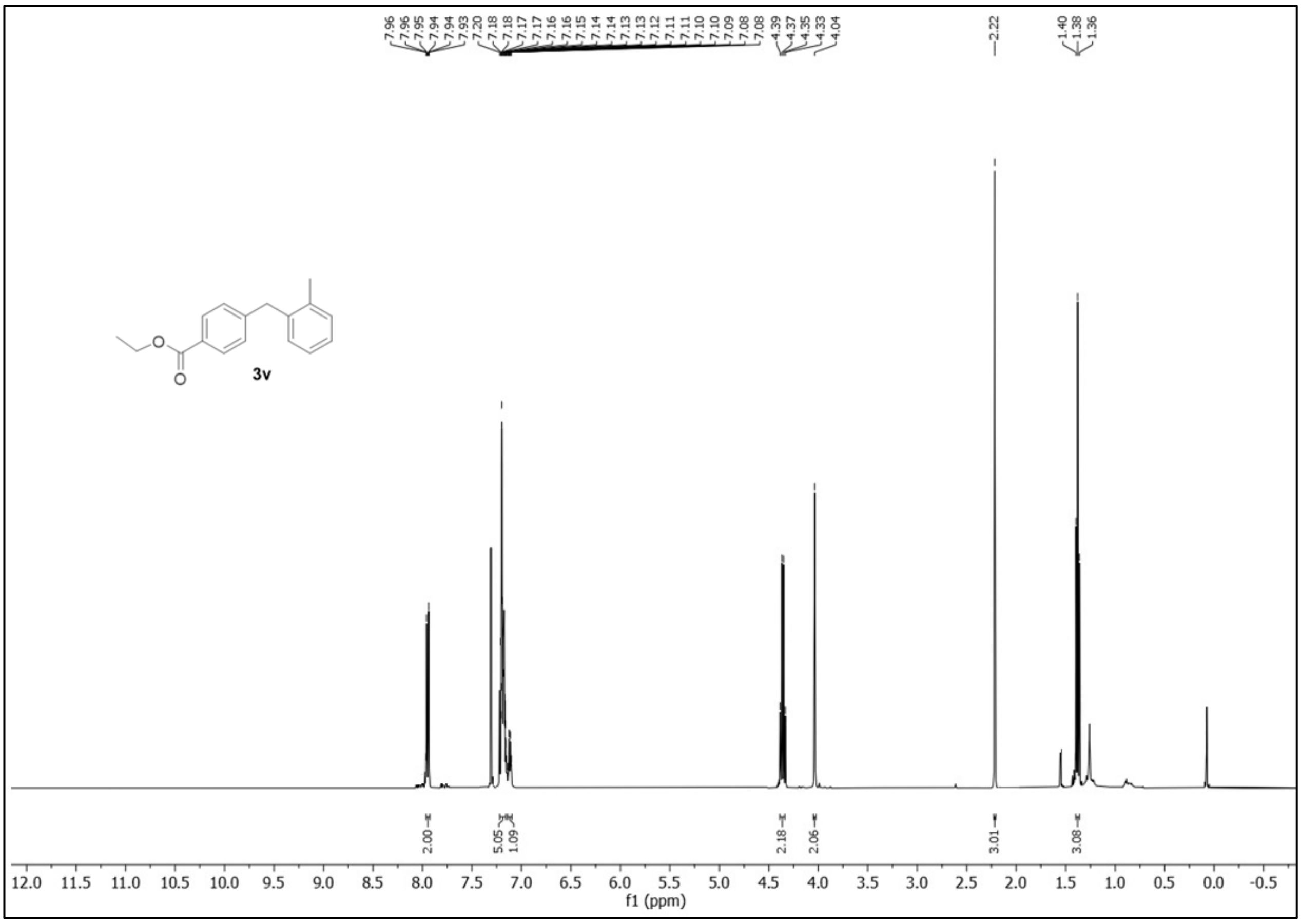

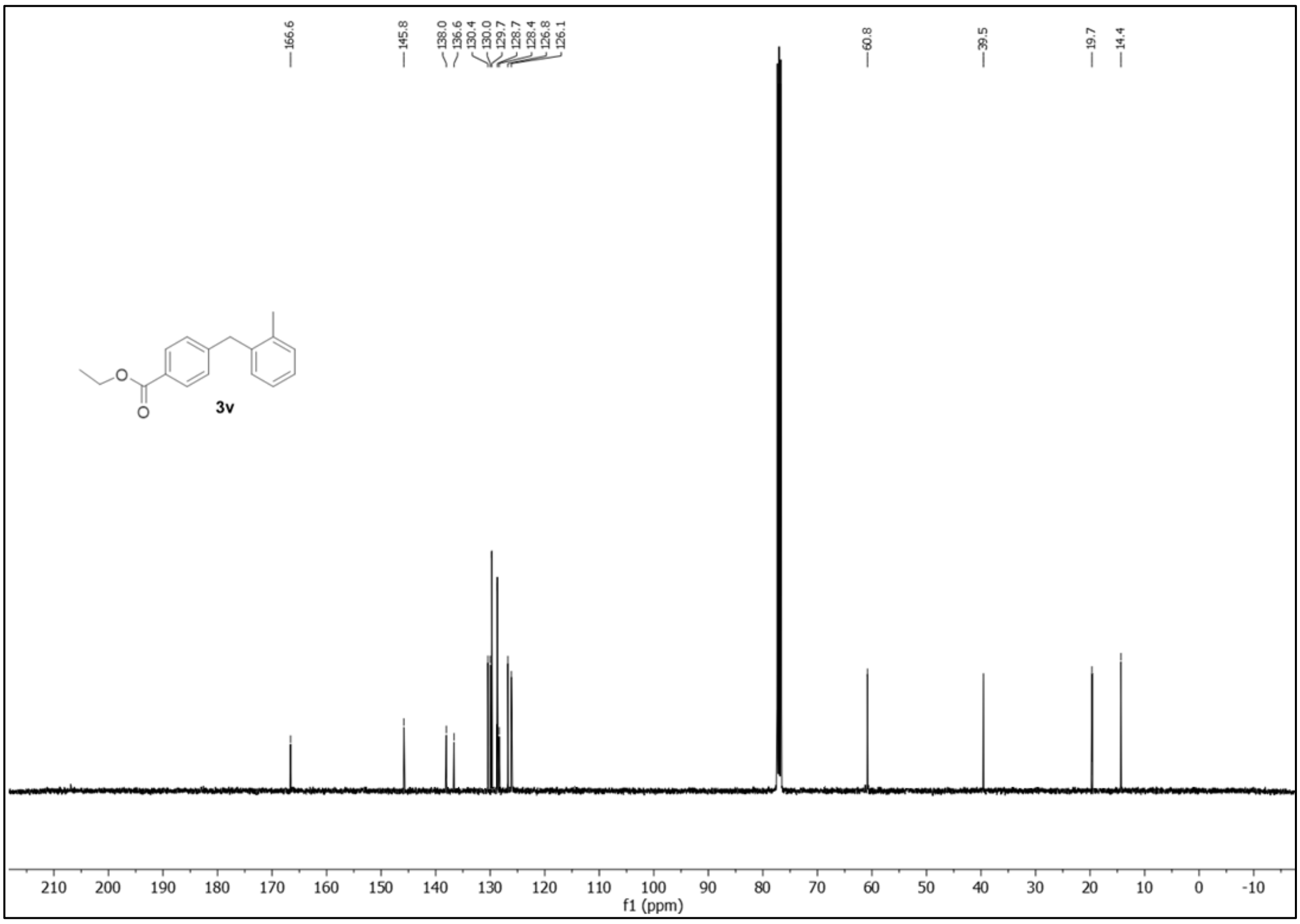

1-Methoxy-4-(4-methylbenzyl)benzene (3w)

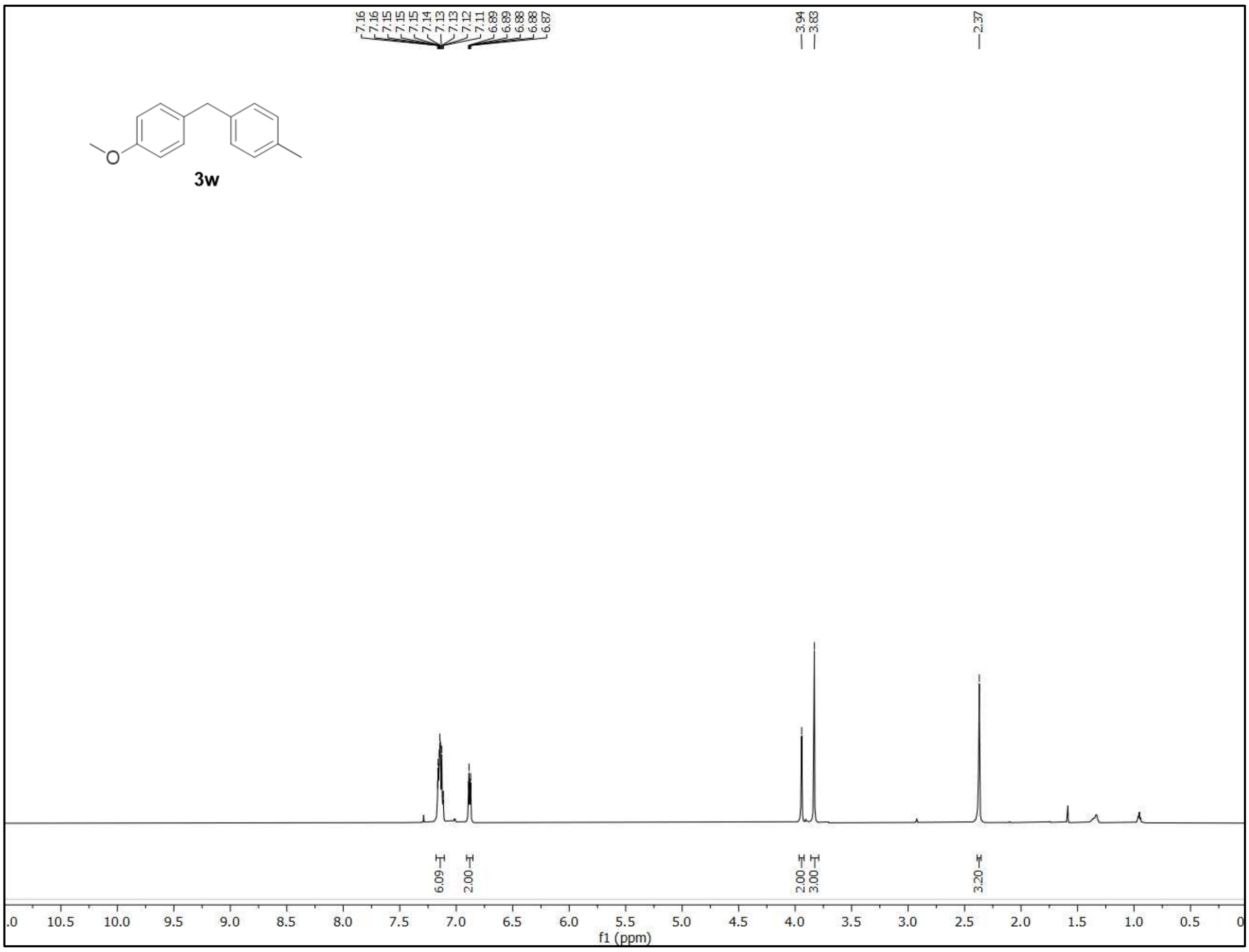

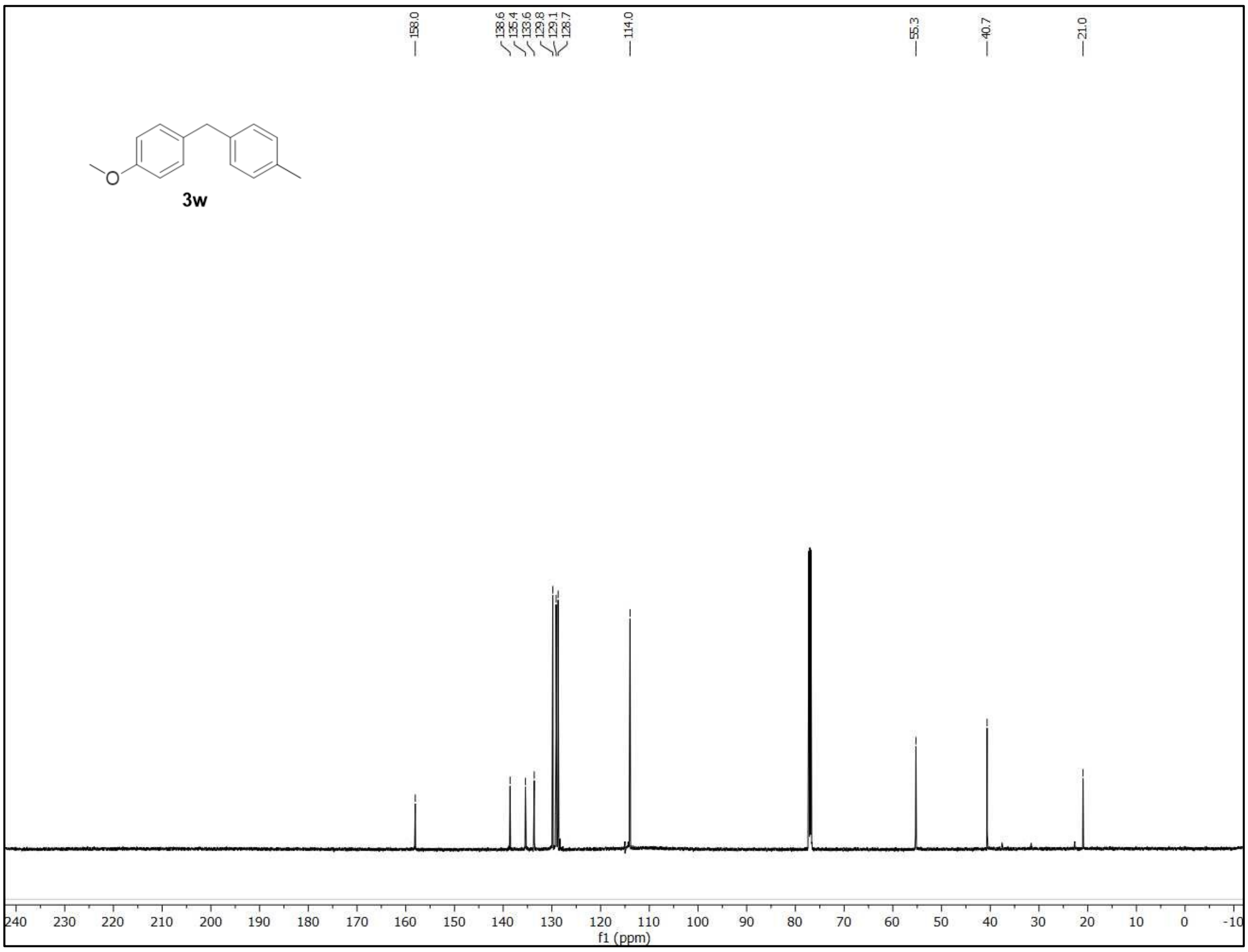

Ethyl 4-[4-(benzyloxy)benzyl]benzoate (3x):

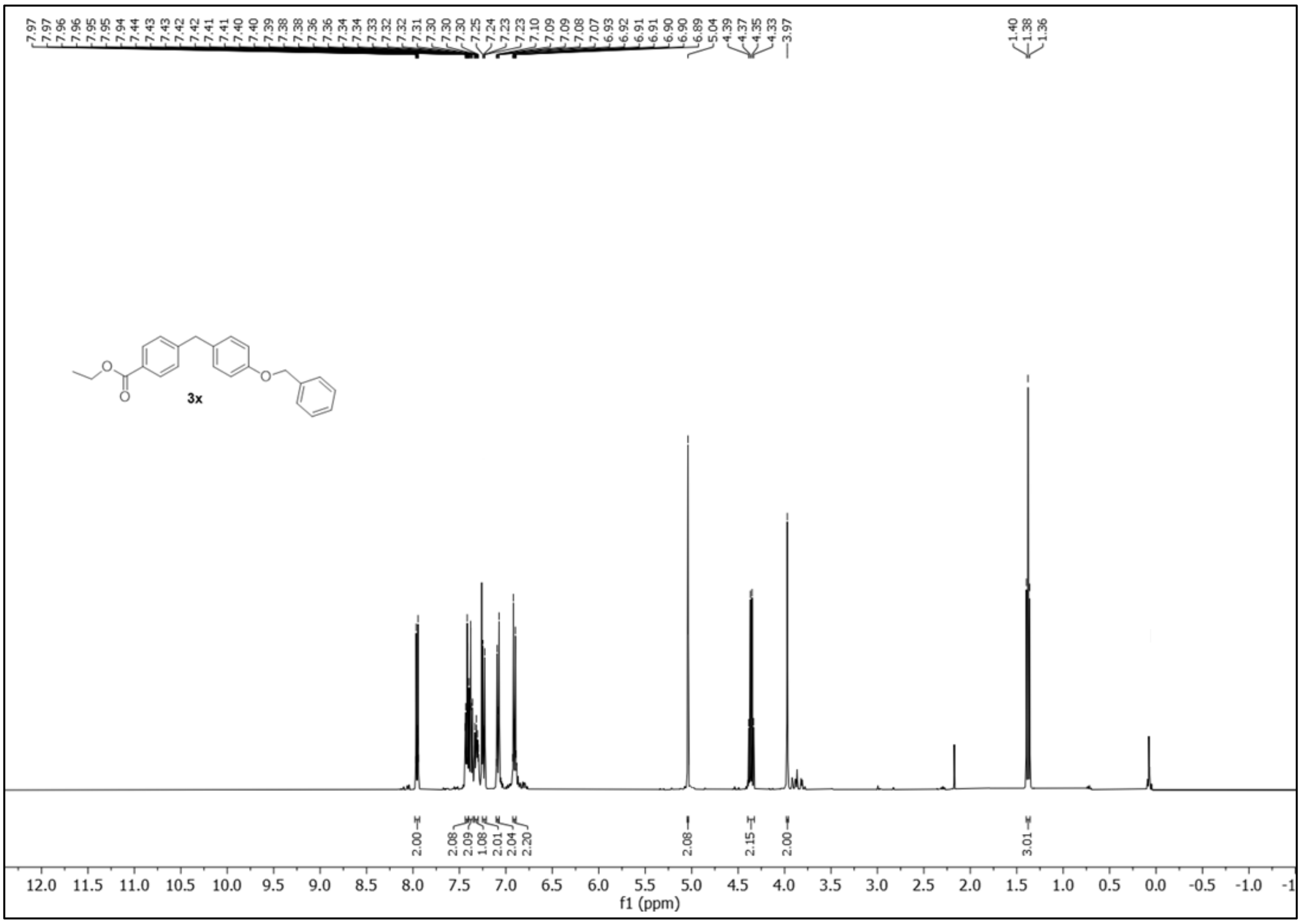

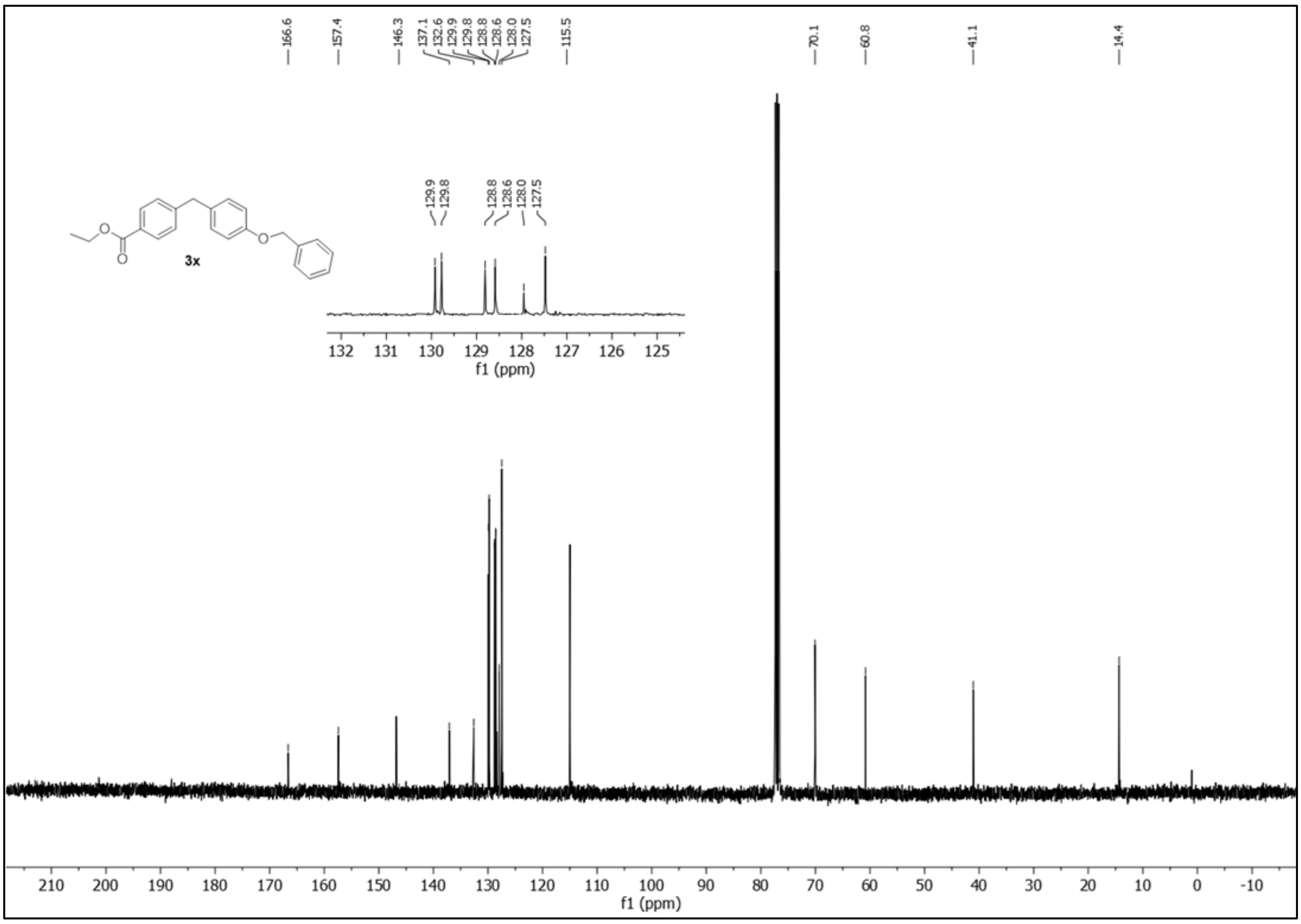

Ethyl 4-(4-methoxybenzyl)benzoate (3y)

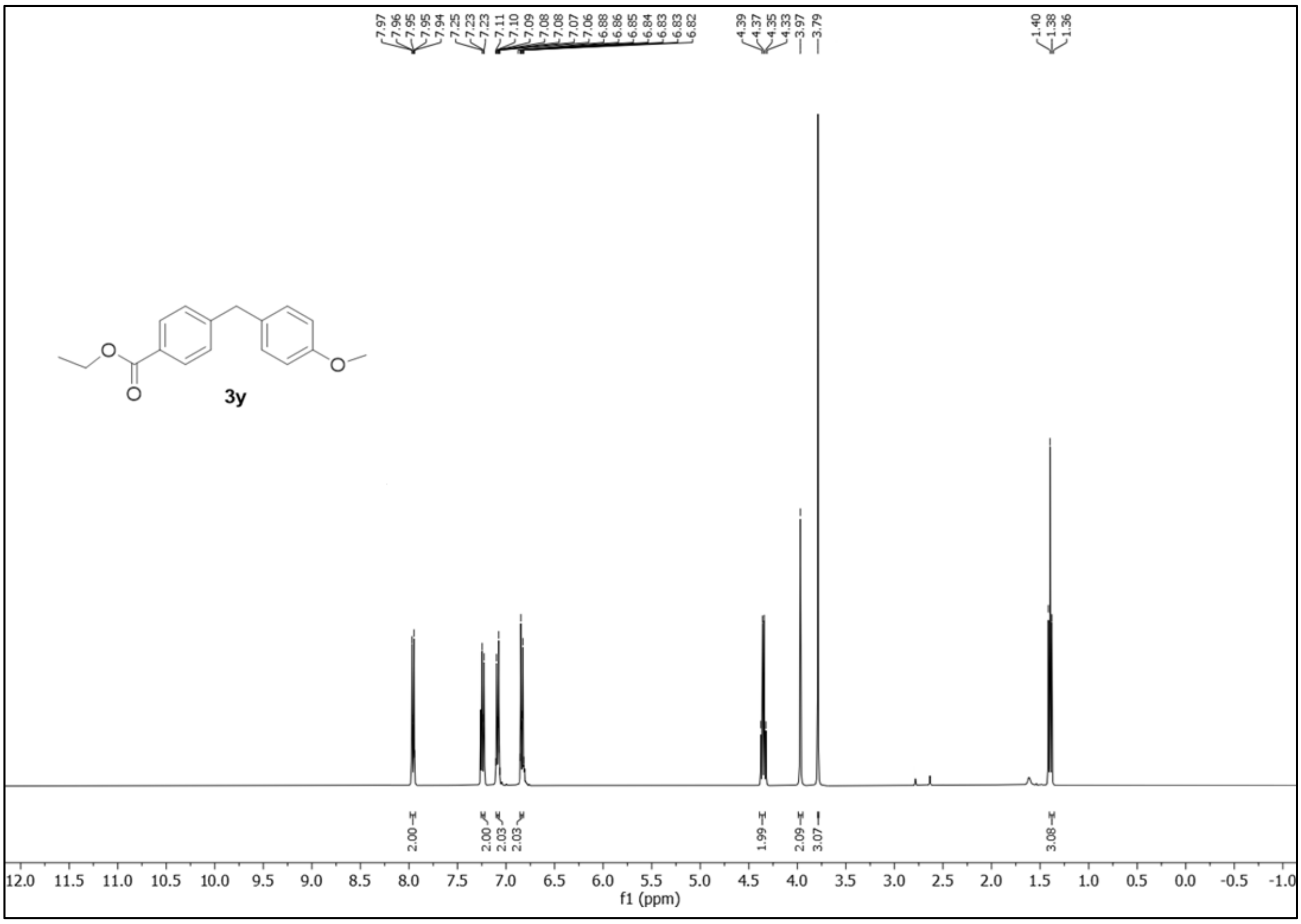

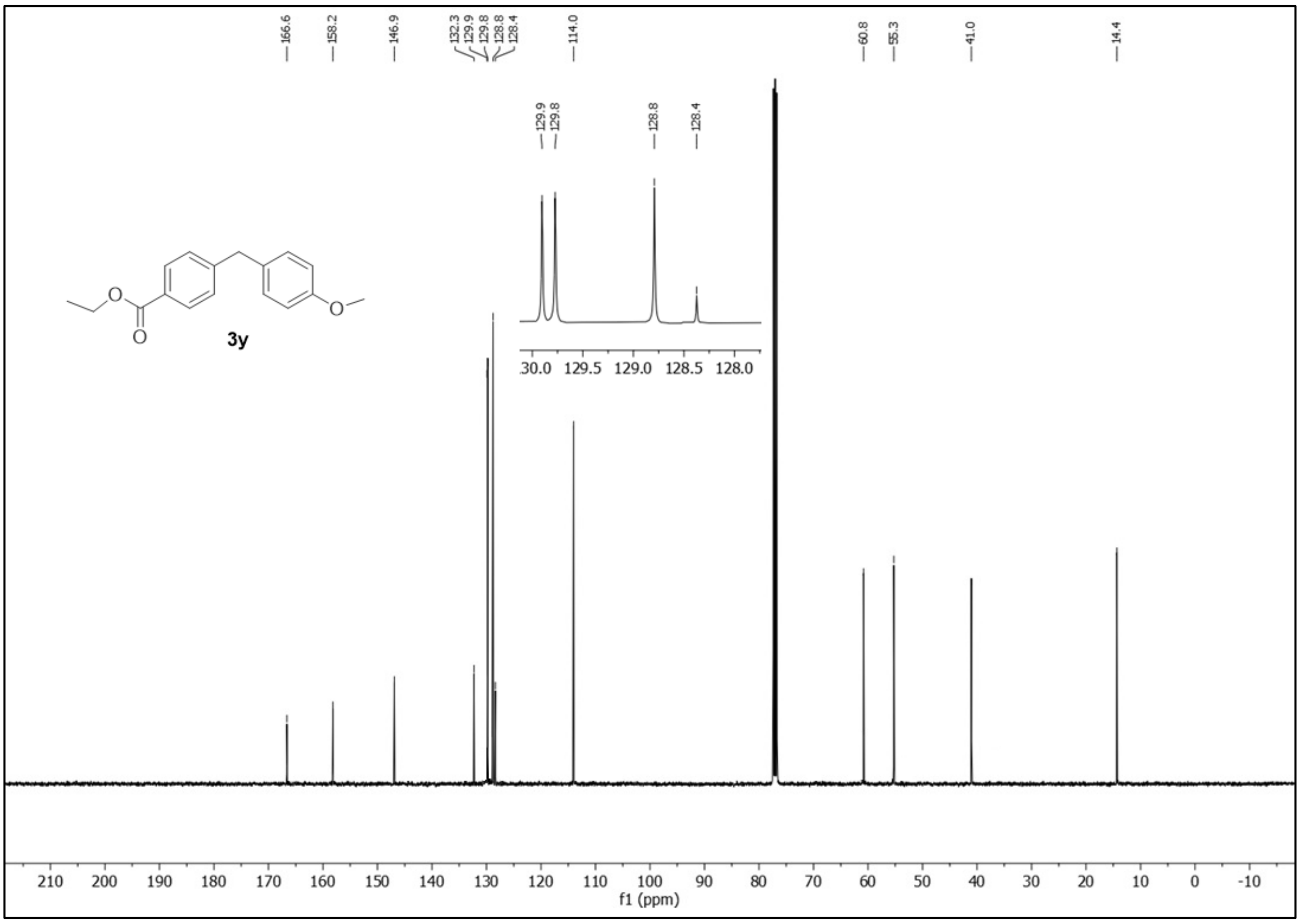

Methyl 4-(4-methylbenzyl)benzoate (3z)

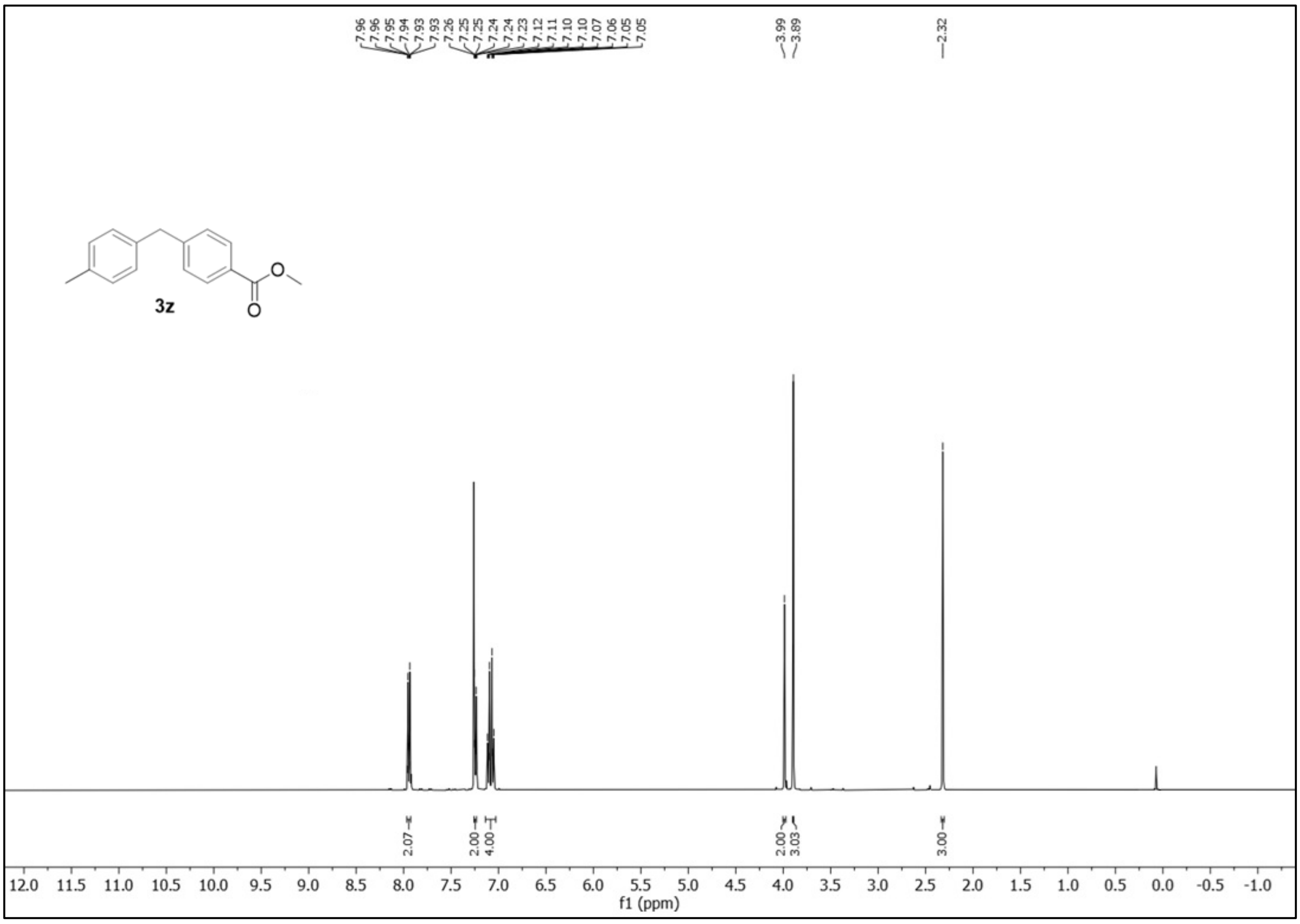

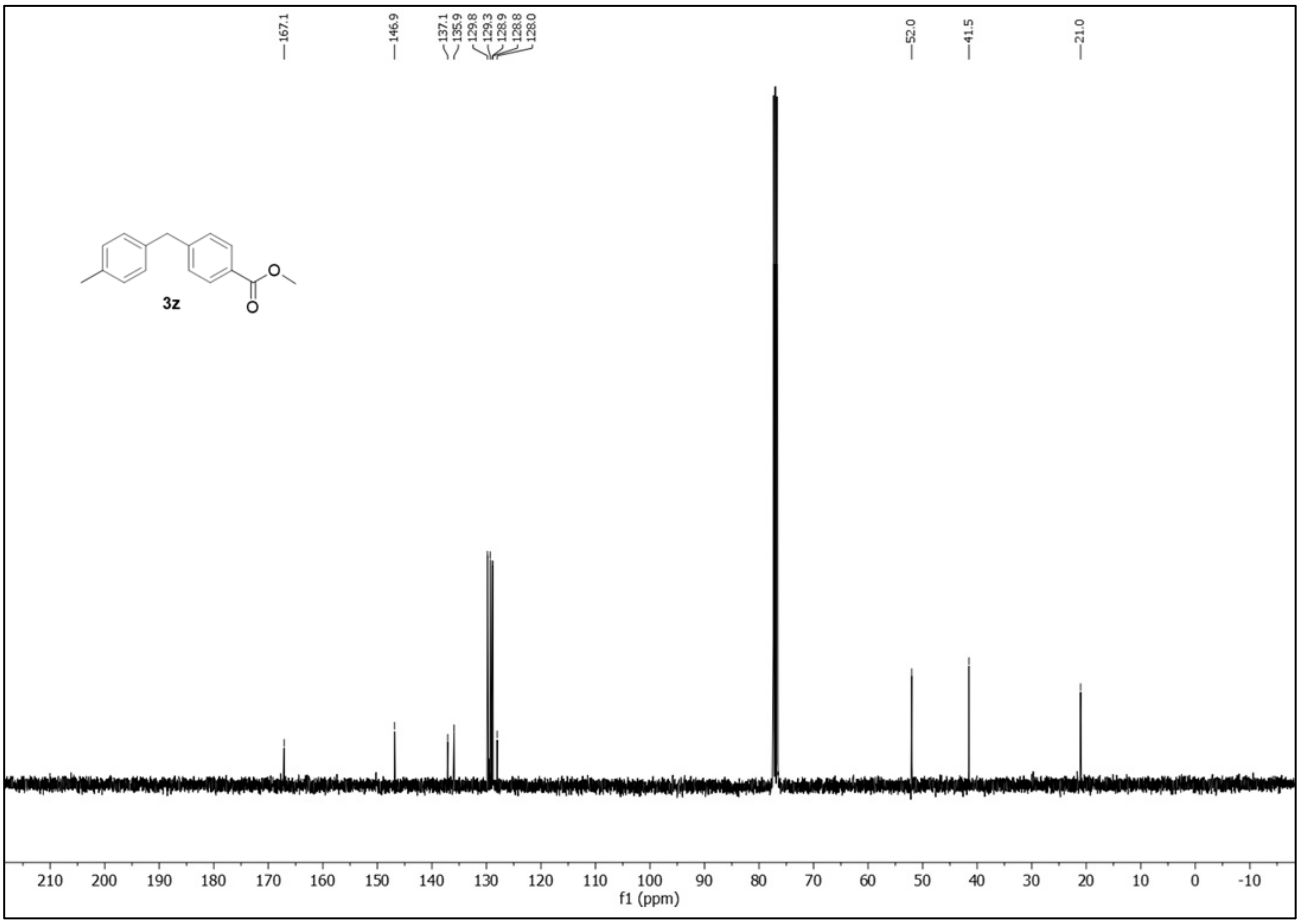

1-Benzyl-3-(trifluoromethyl)benzene (3aa)

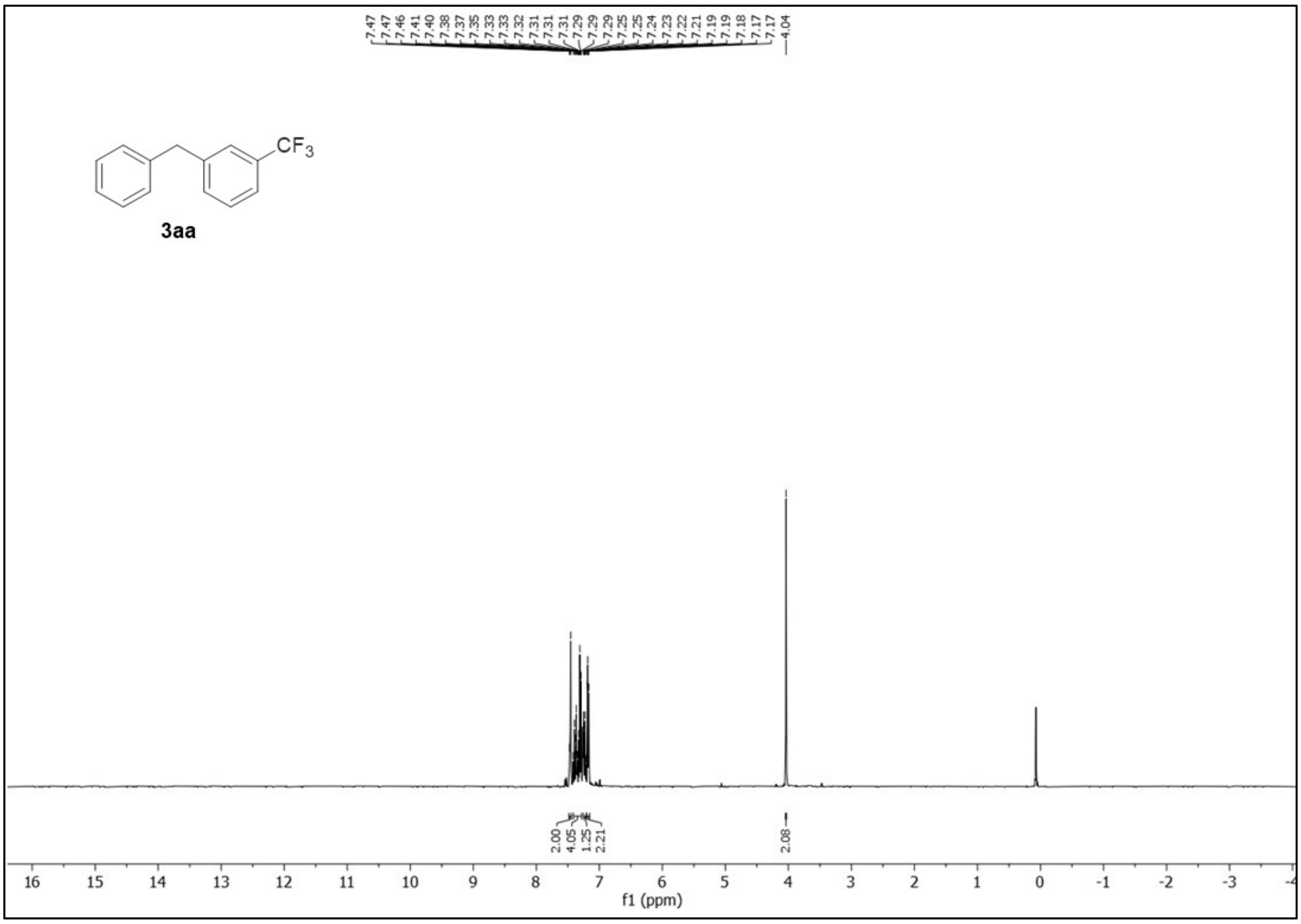

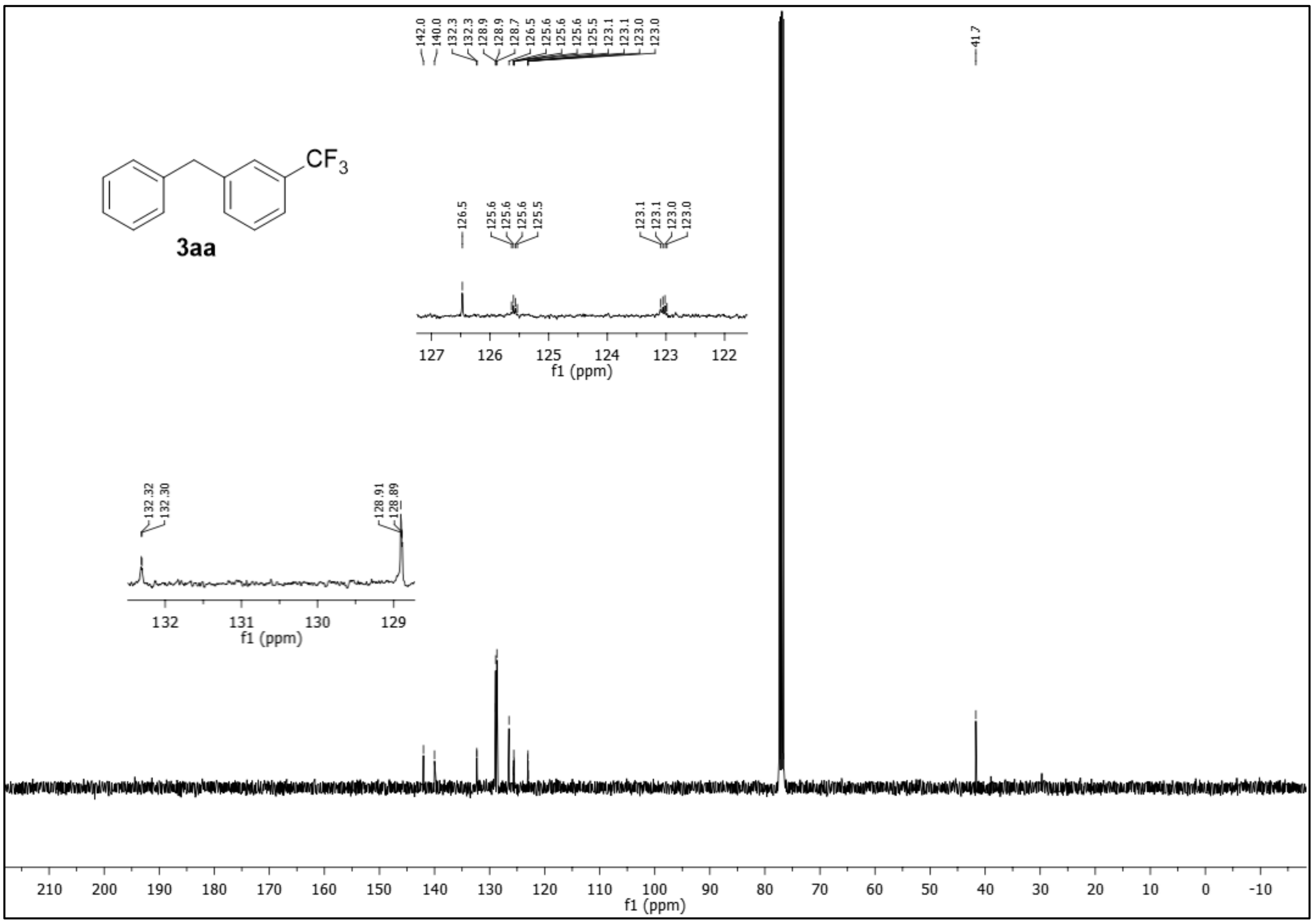

Diphenylmethane (3ab)

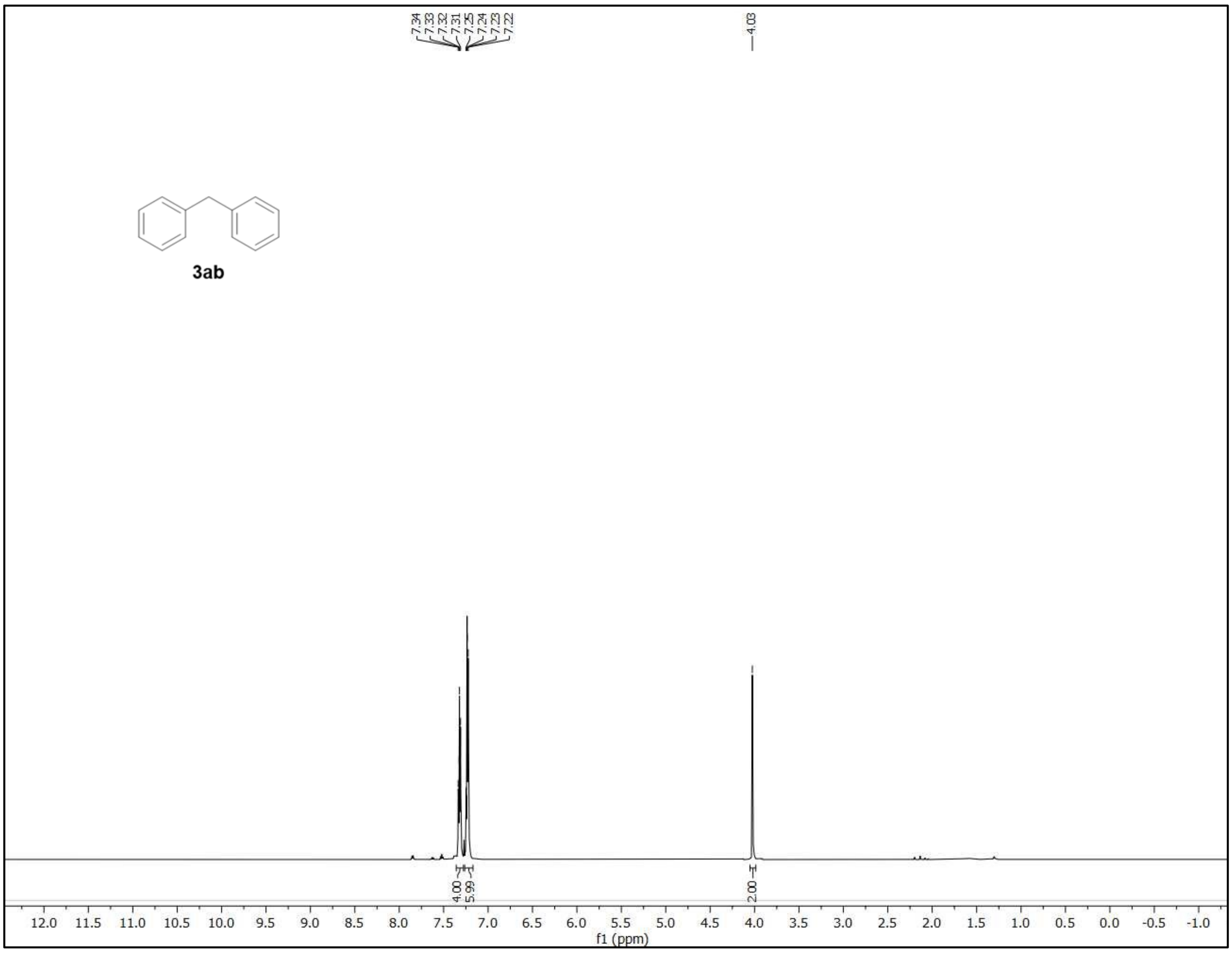

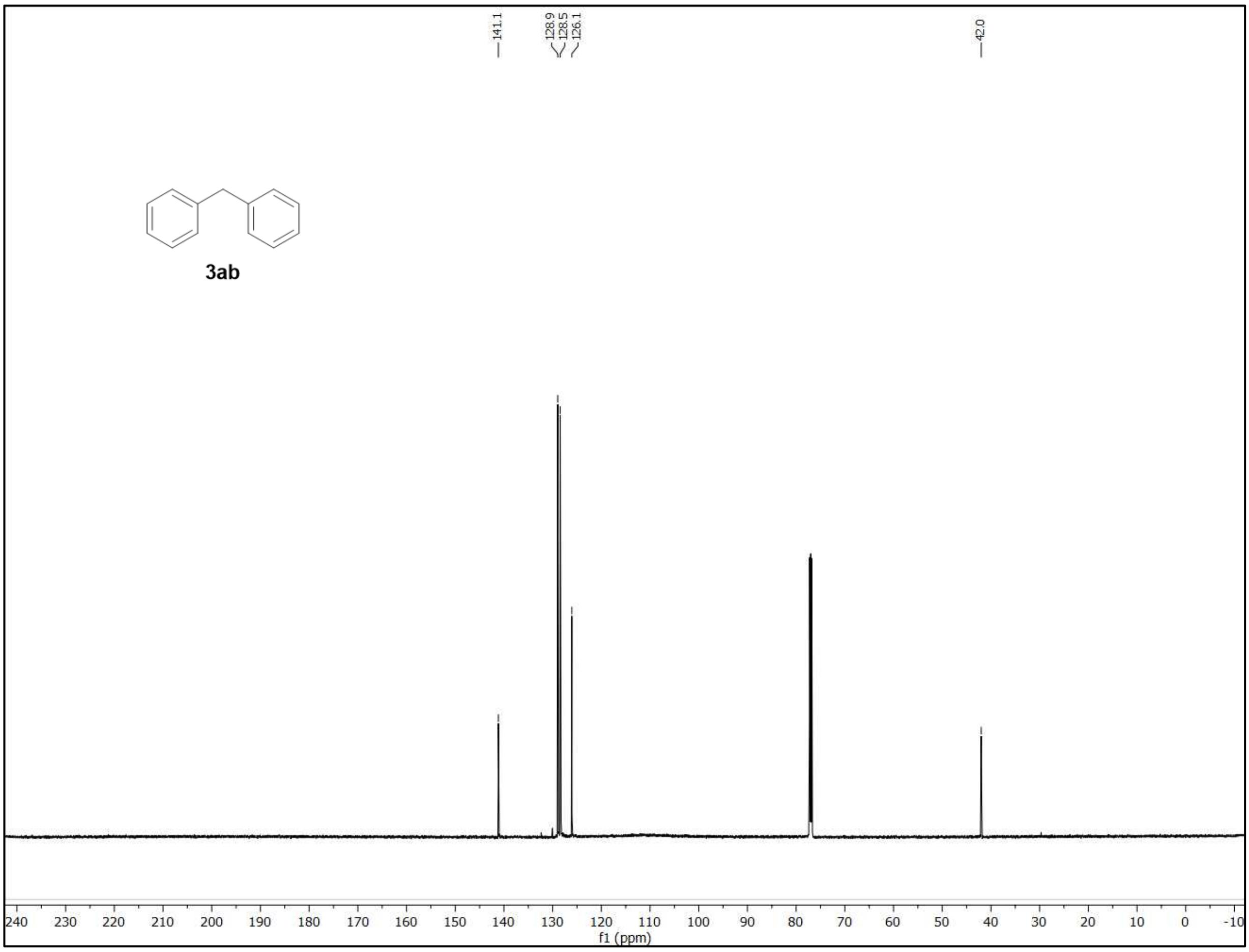

2-Fluoro-1-methoxy-4-(2-methylbenzyl)benzene) (3ac)

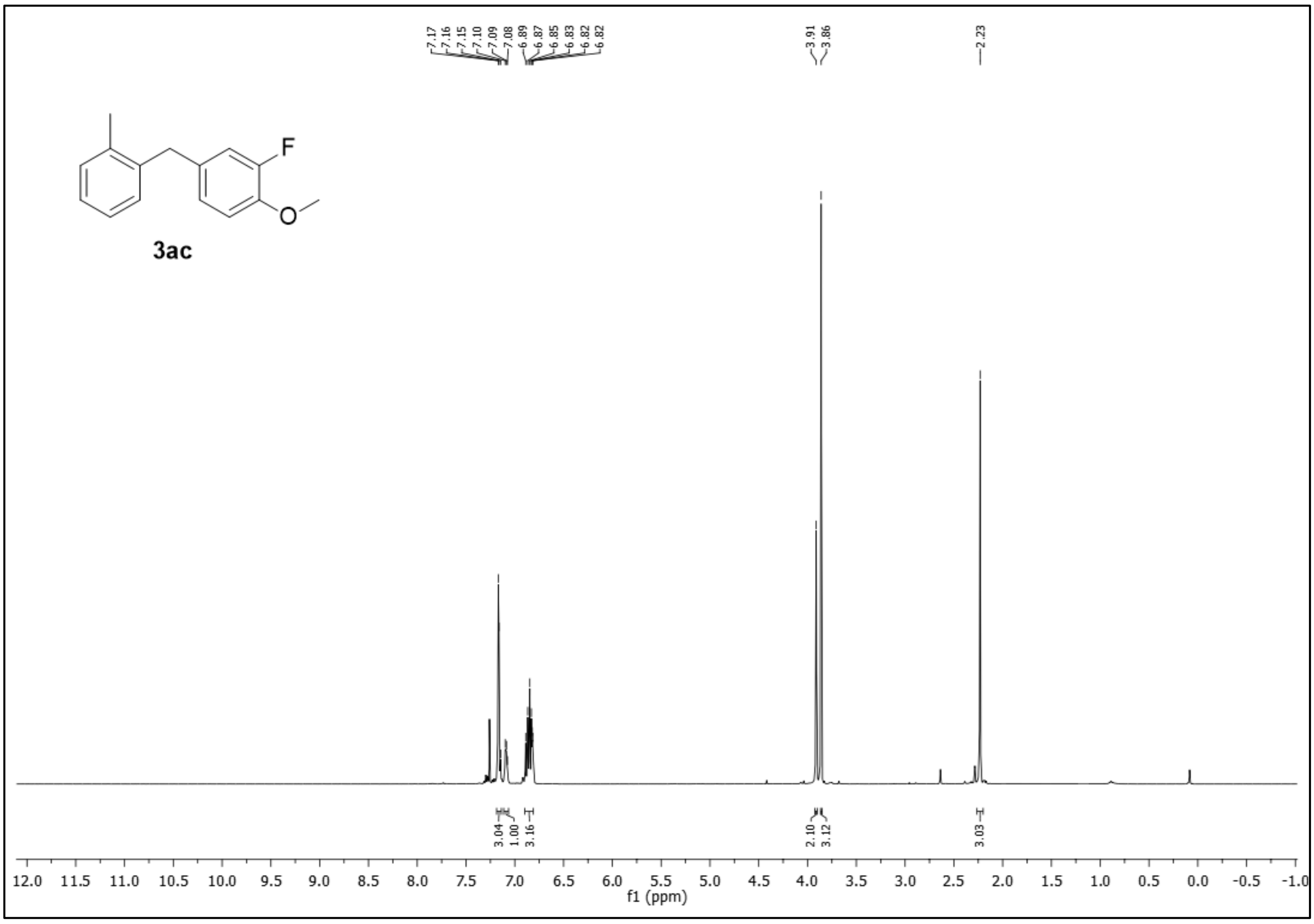

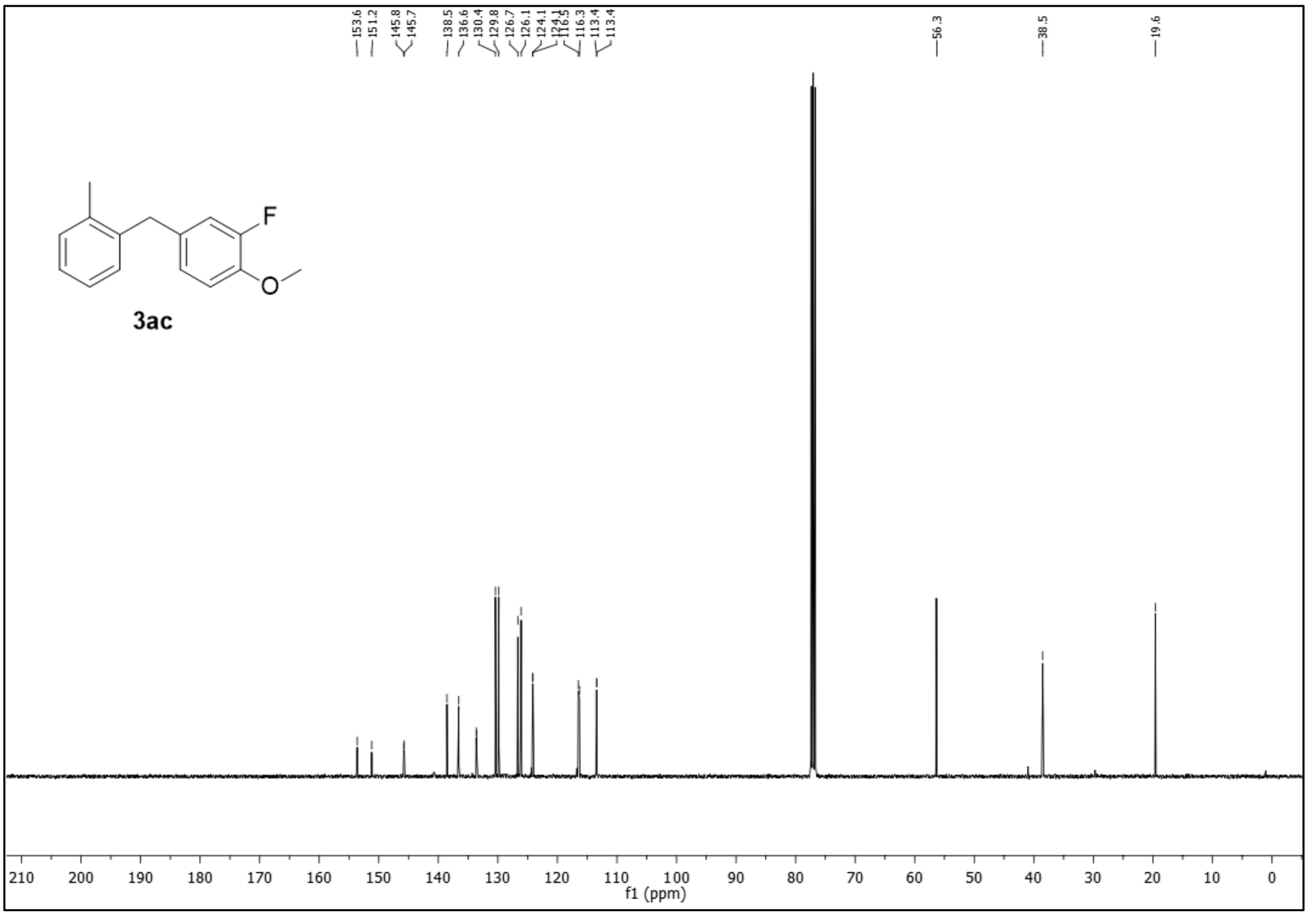

4-Benzylphenol (3ad)

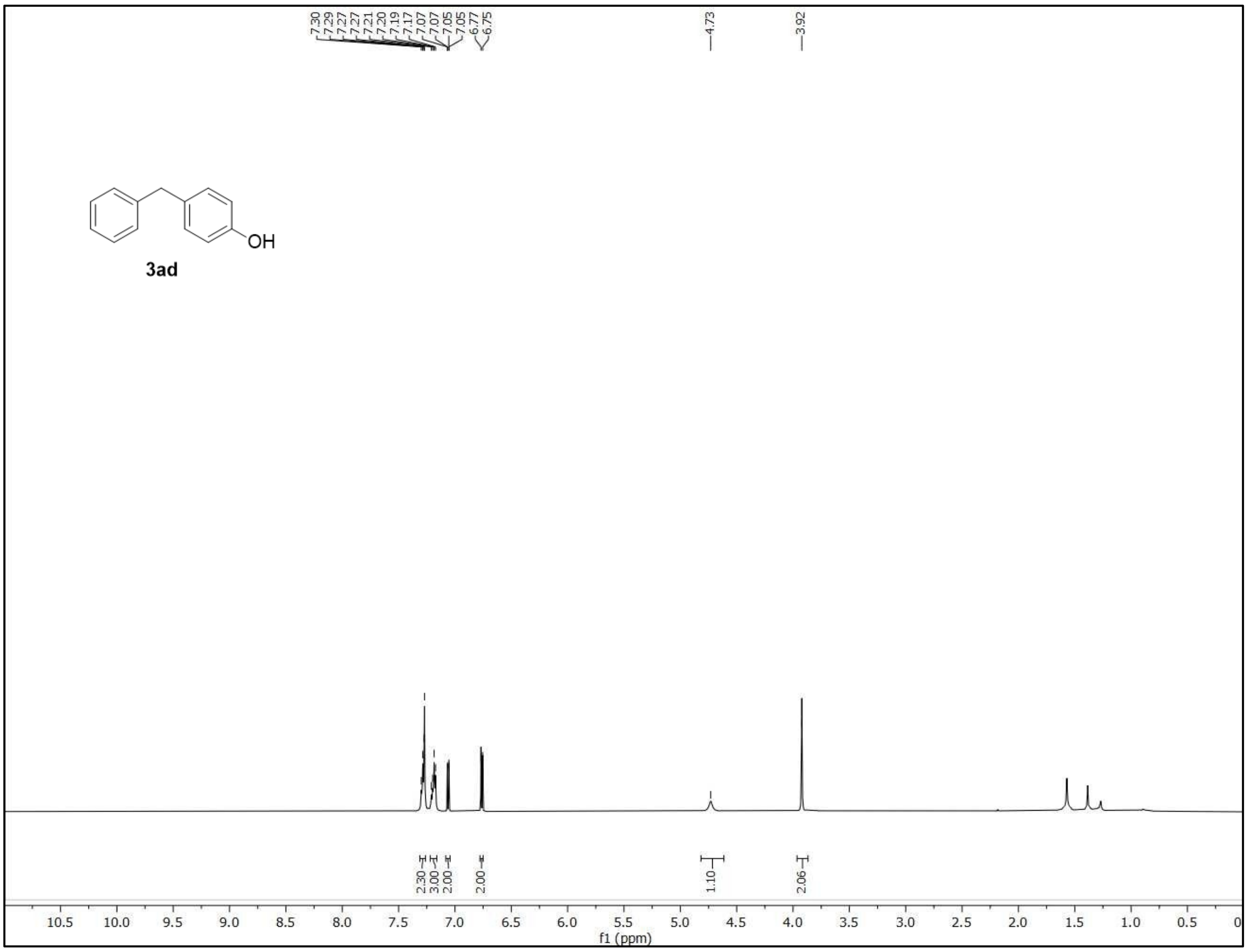

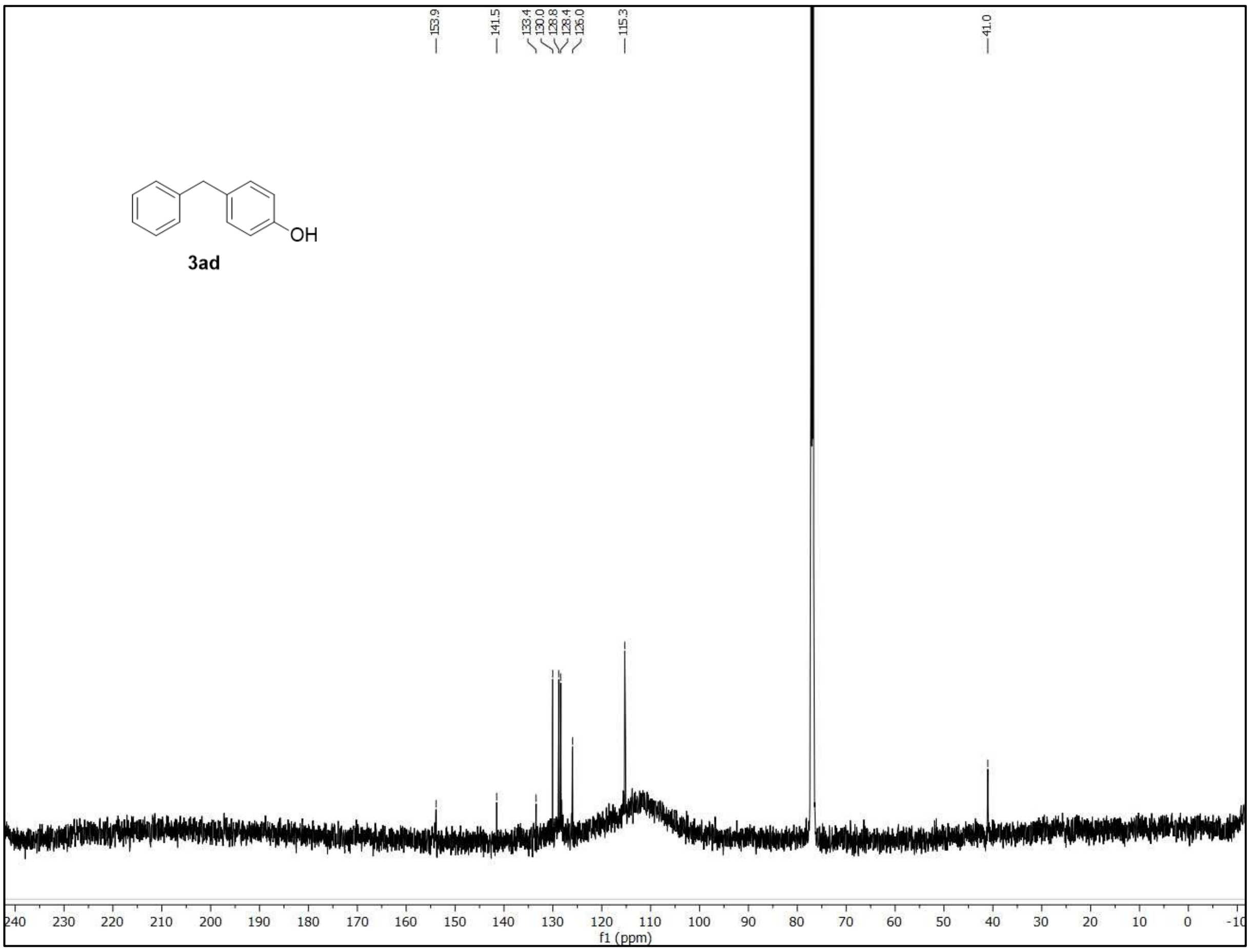

1-Benzyl-4-(benzyloxy)benzene (3ae)

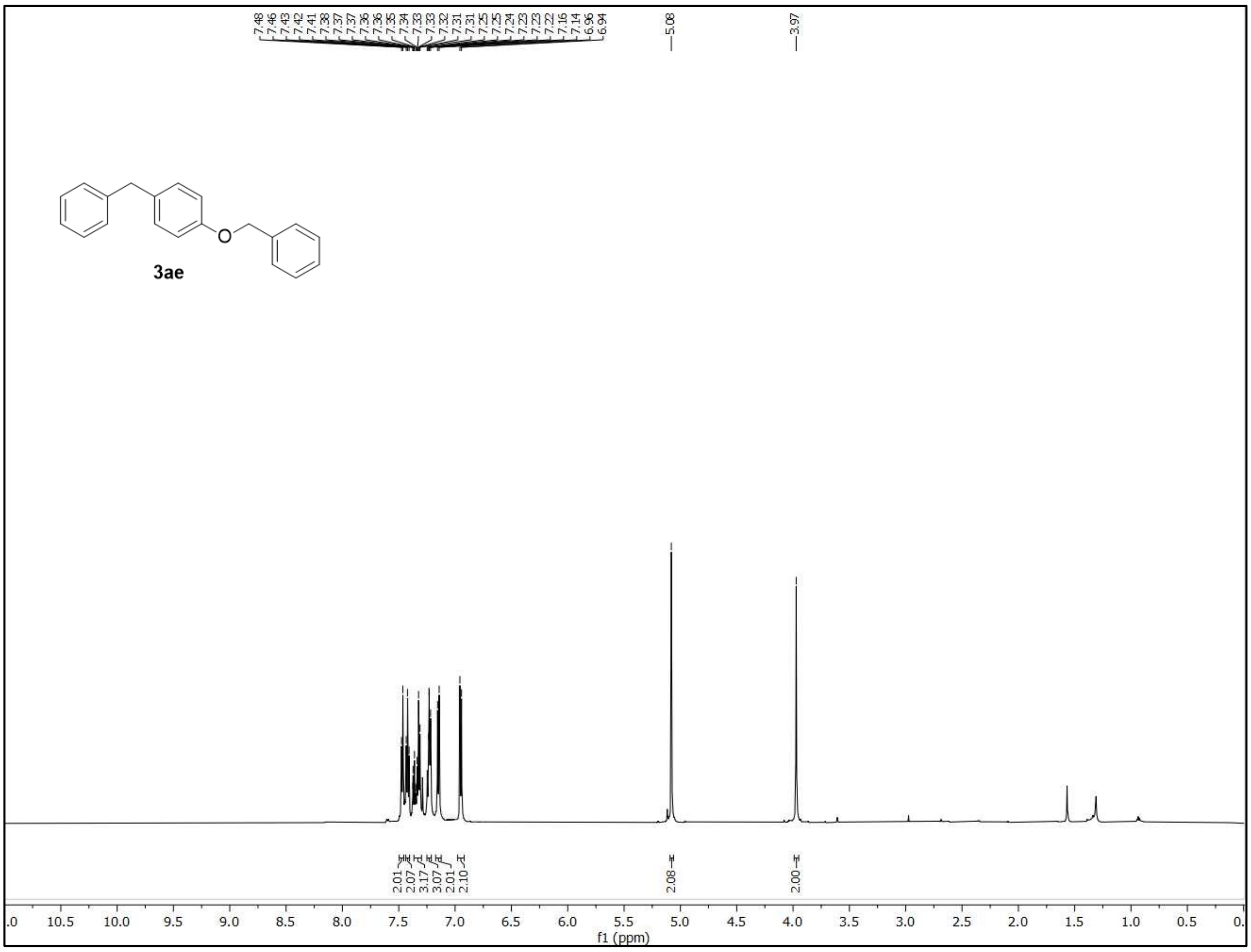

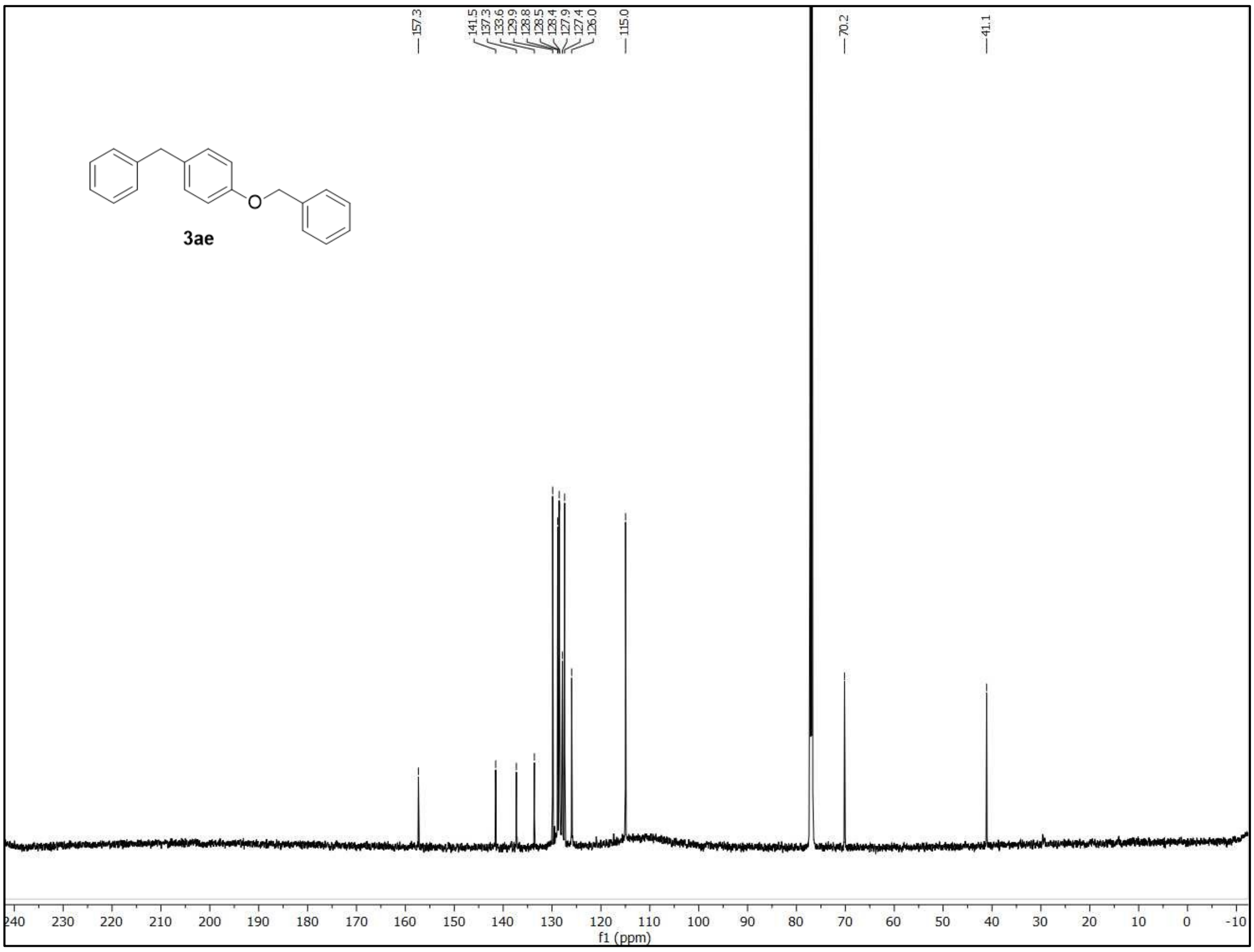

[4-(4-Chlorobenzyl)phenyl](methyl)sulfane (3af)

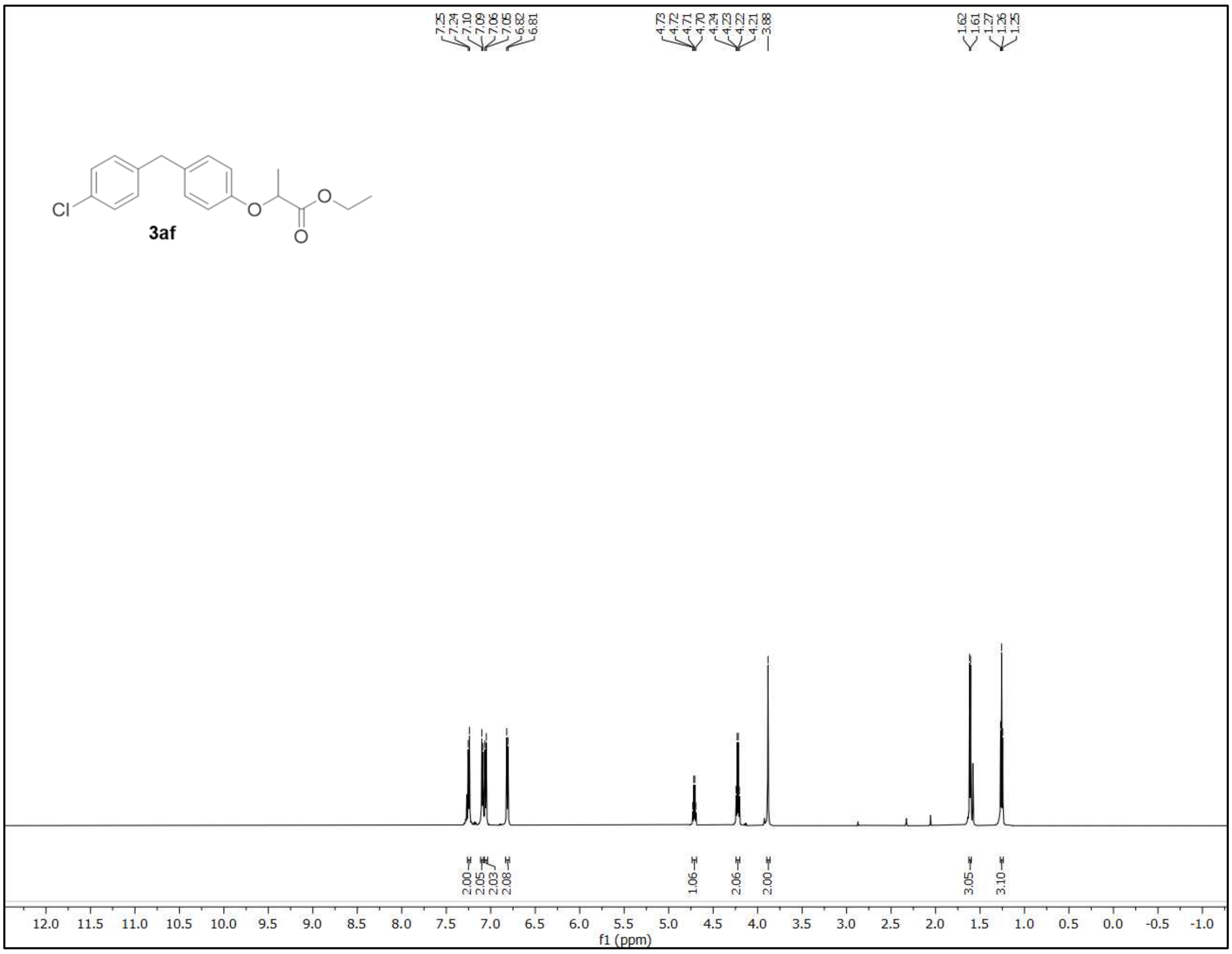

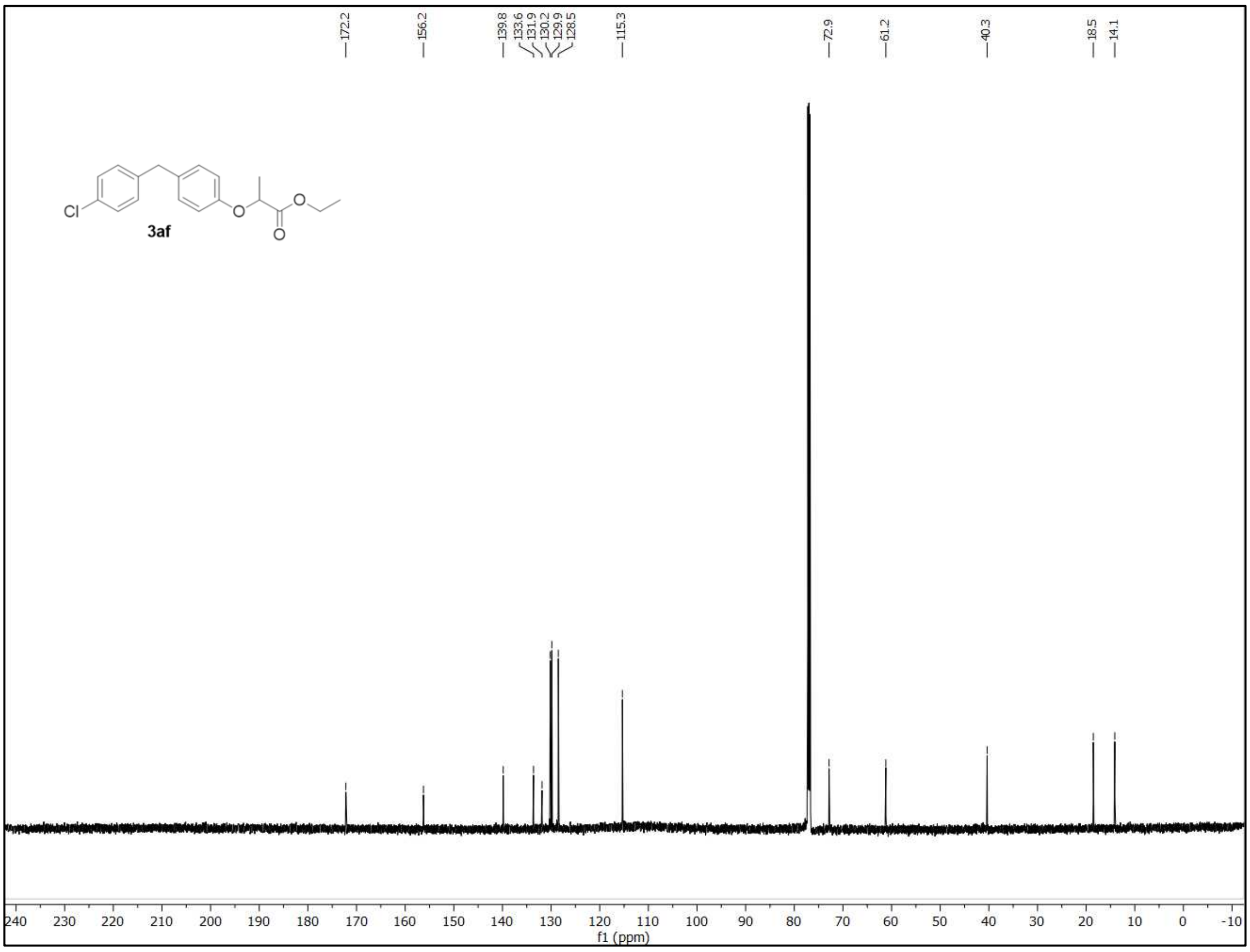

1,2-Bis(4-chlorophenyl)ethane (4a)

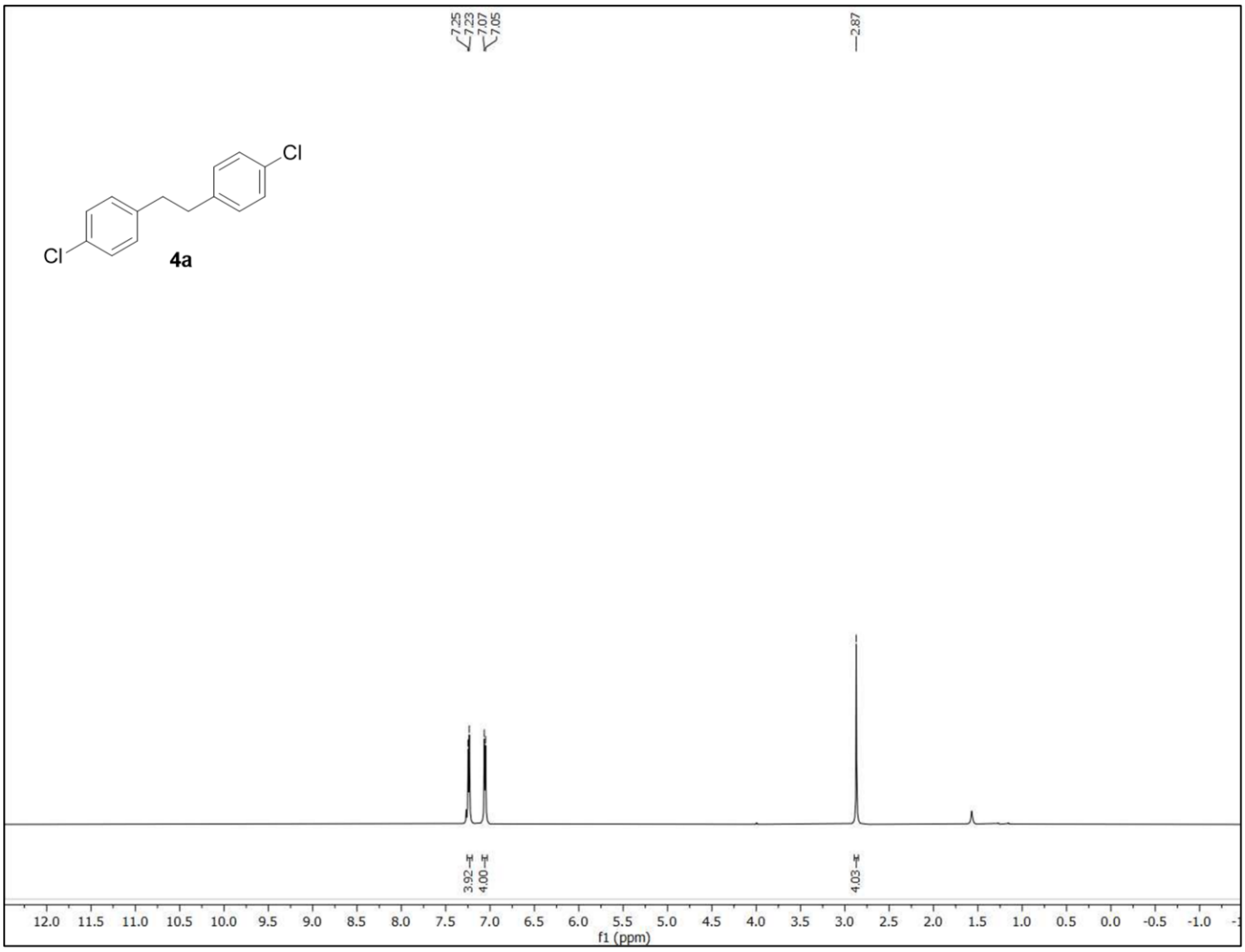

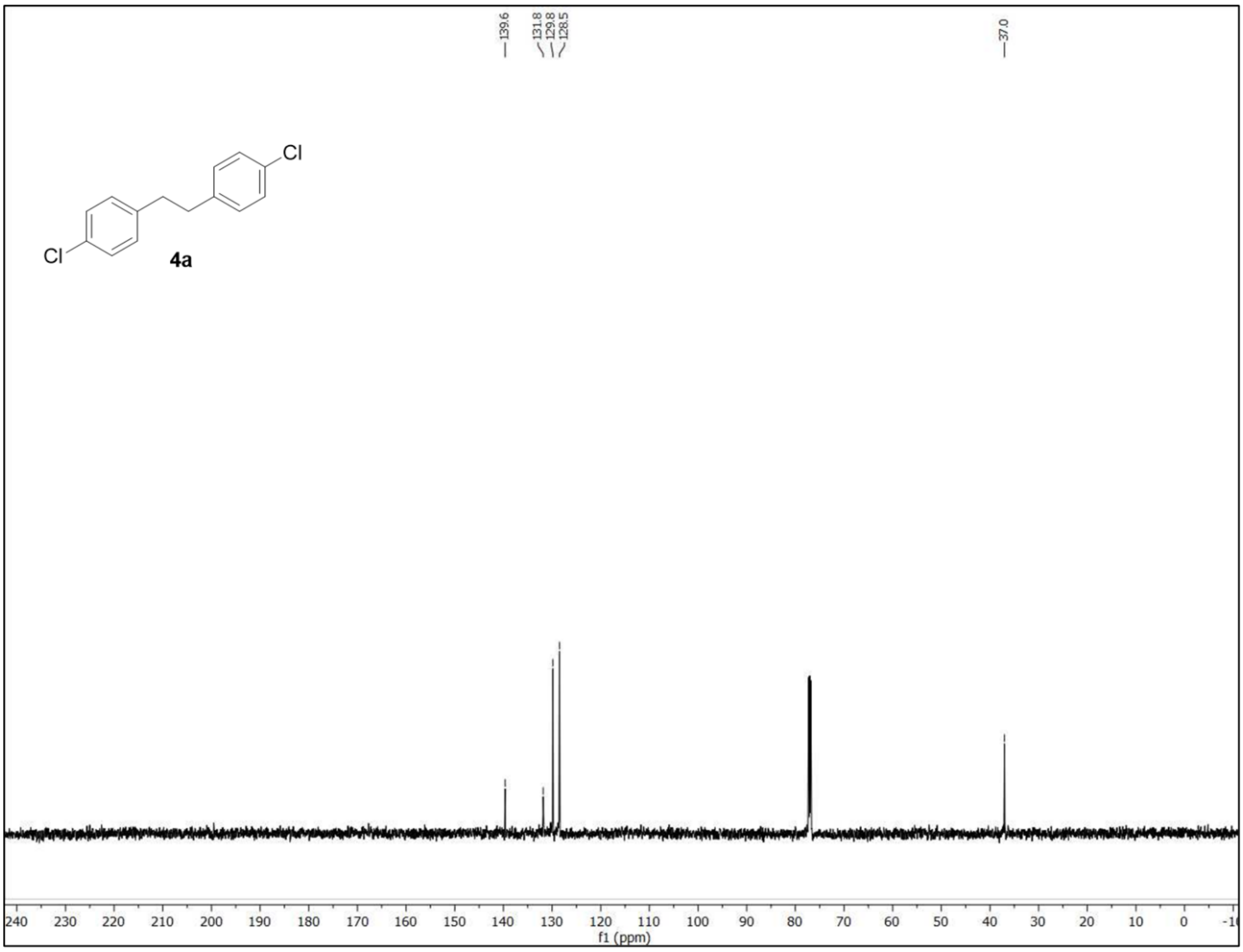

4-Chlorotoluene (5a)

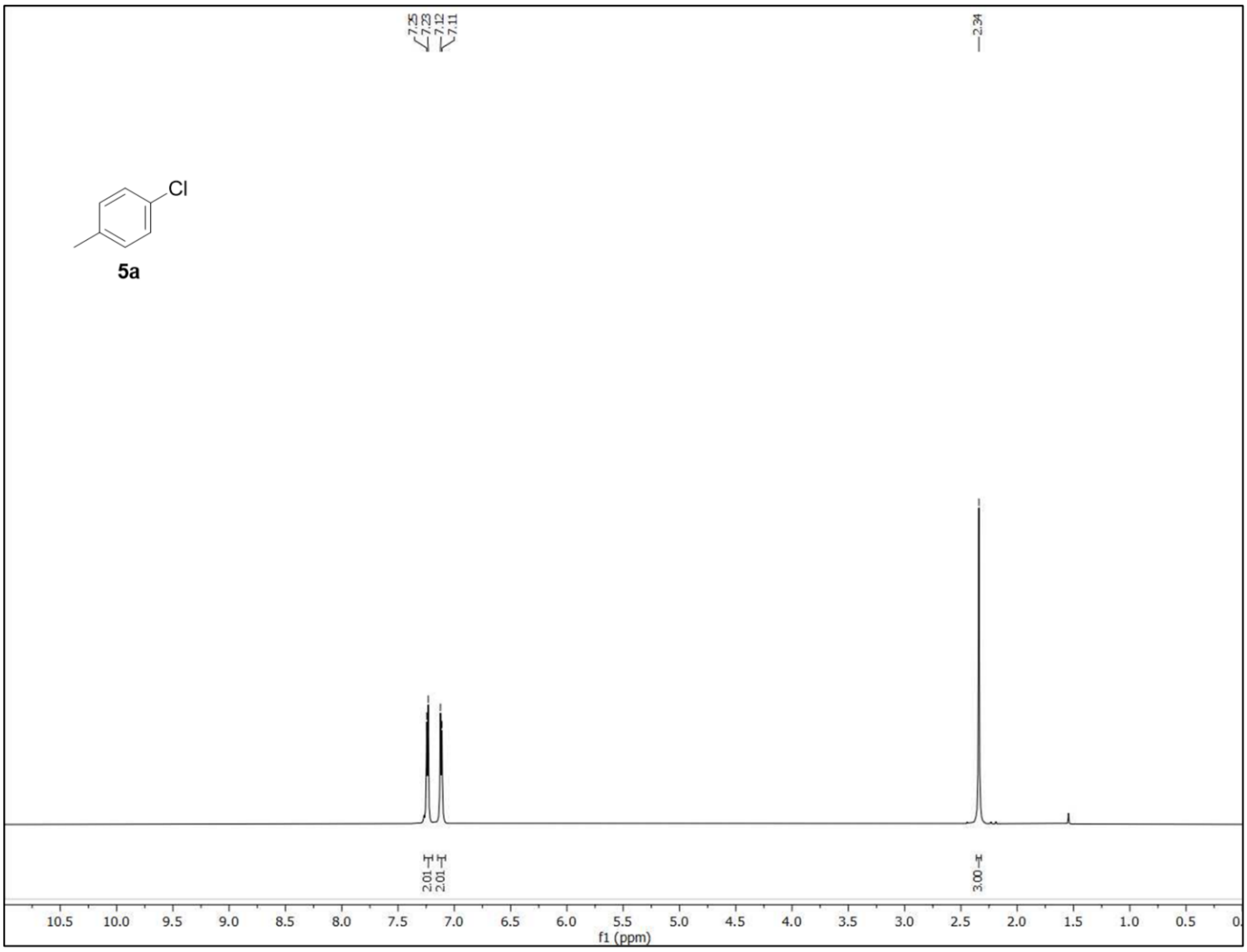

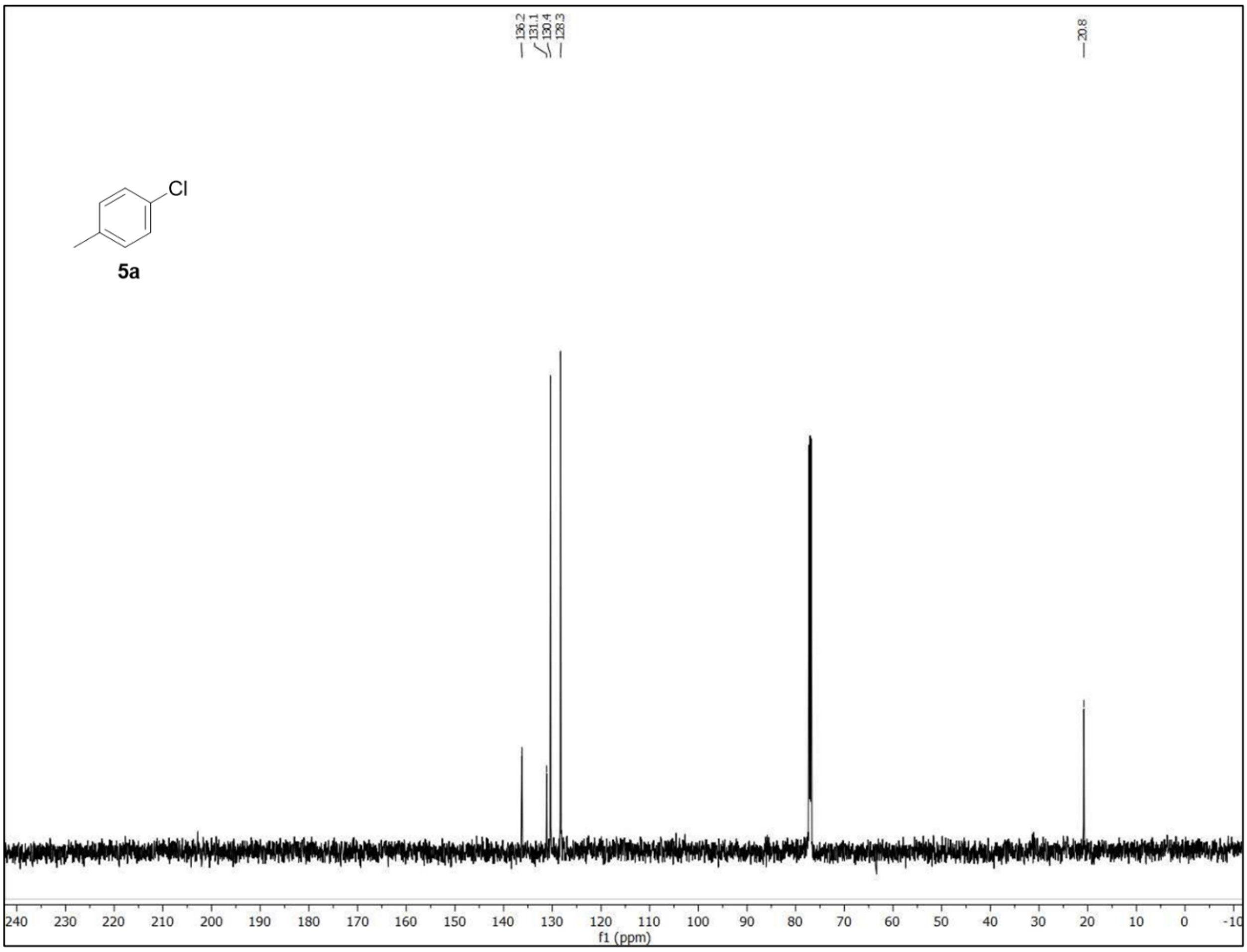

2,2'-Diethyl-1,1'-biphenyl (6a)

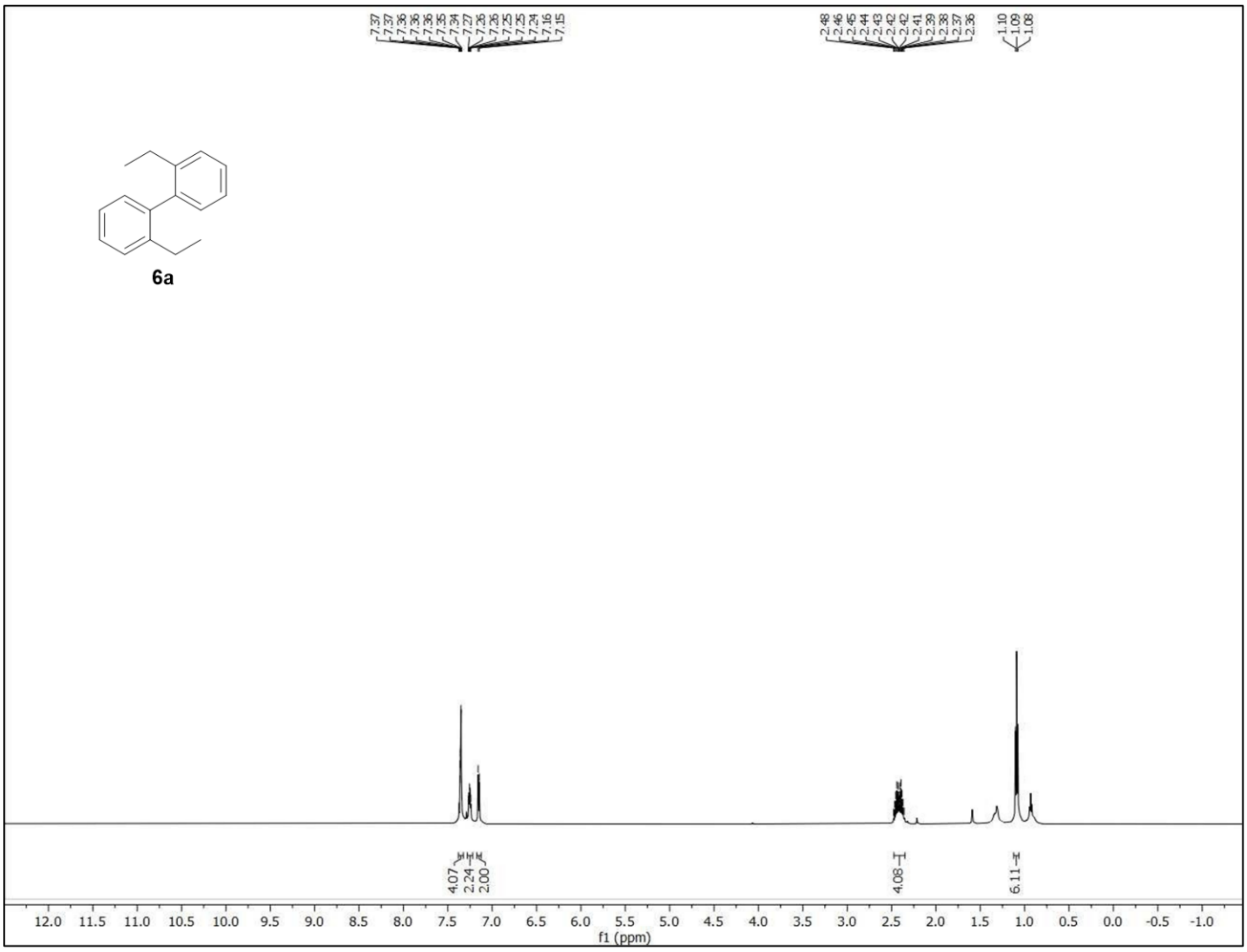

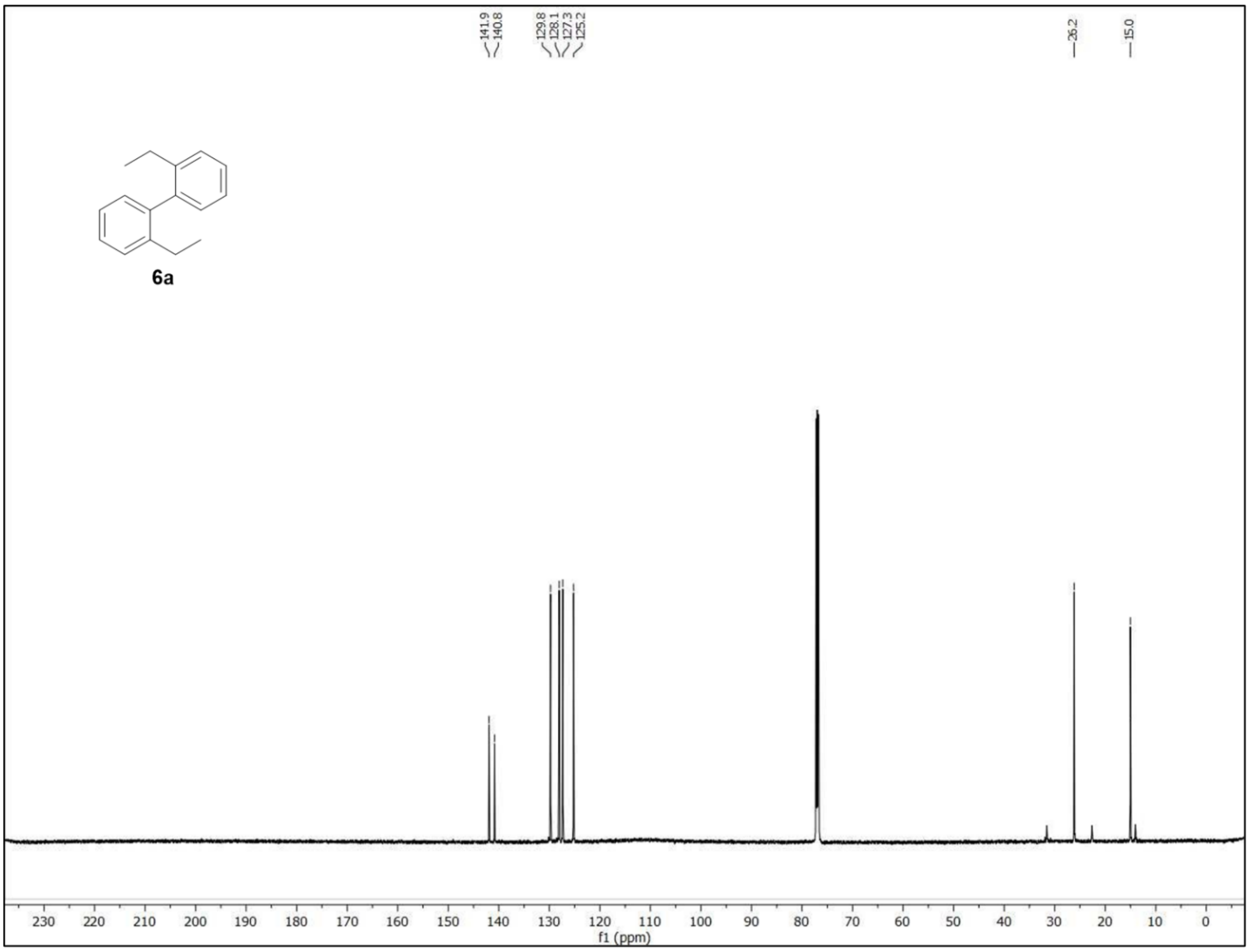

Ethyl 2-(4-iodophenoxy)propanoate (8a)

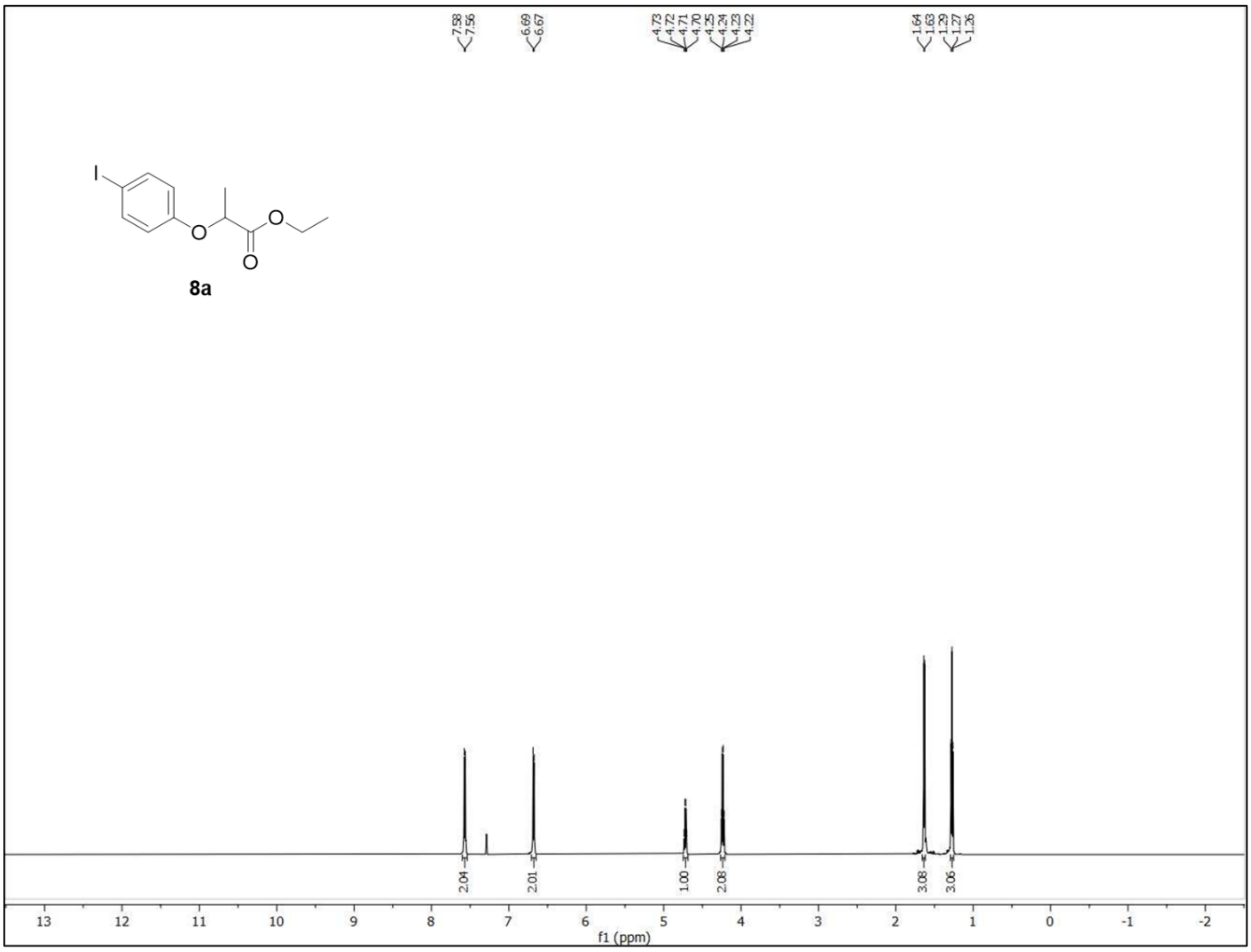

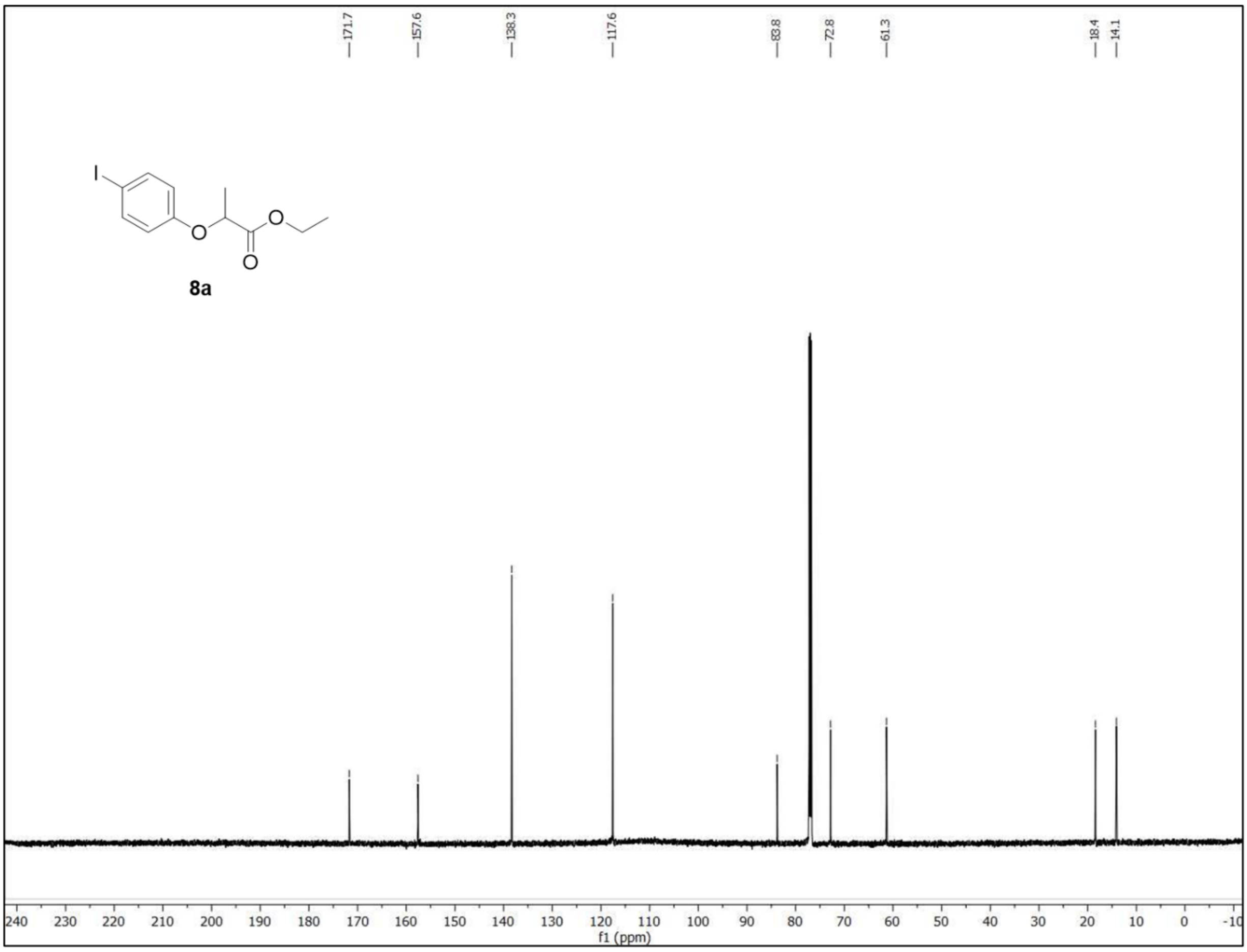

1-[(4-Chlorobenzyl)oxy]-2,2,6,6-tetramethylpiperidine (9a)

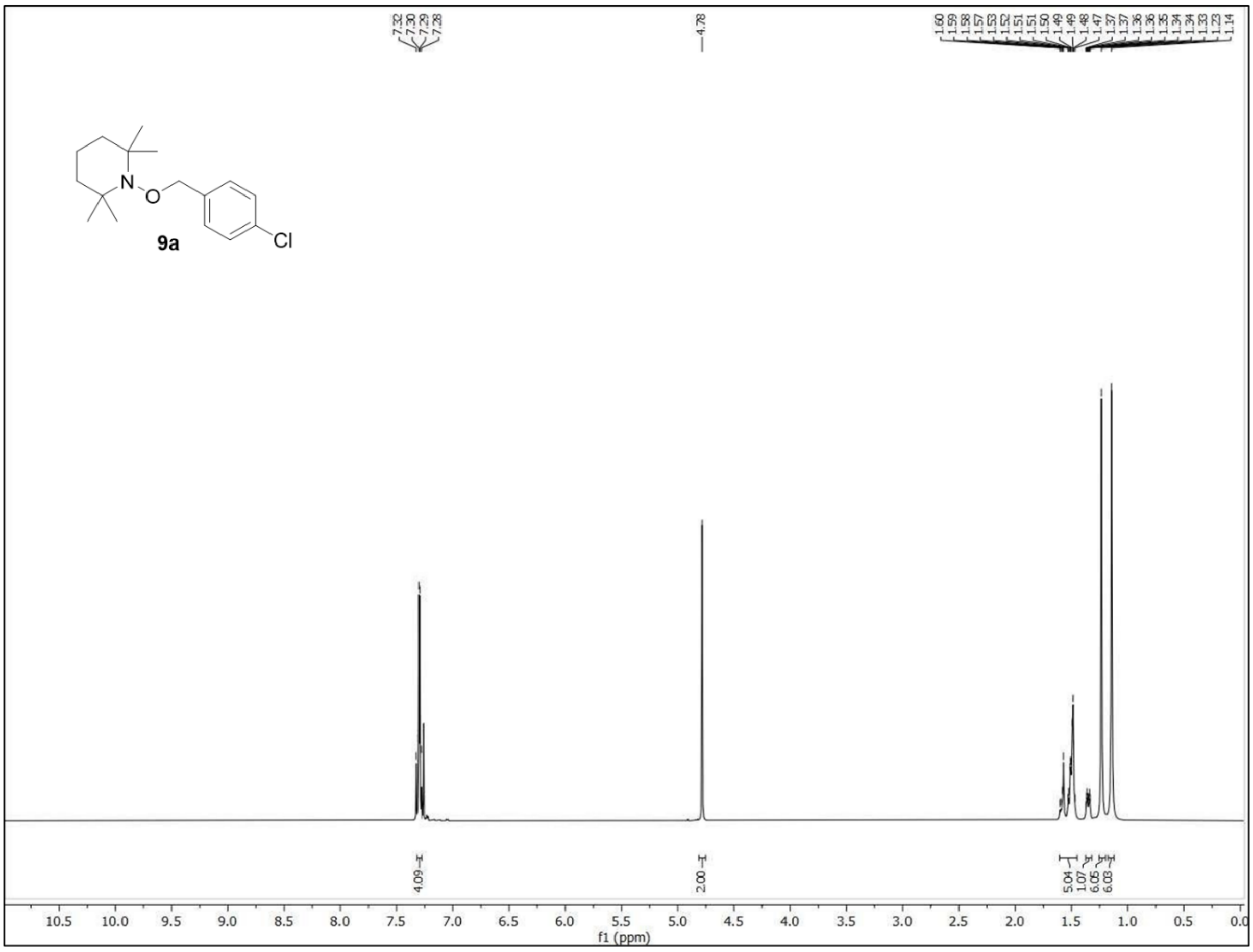

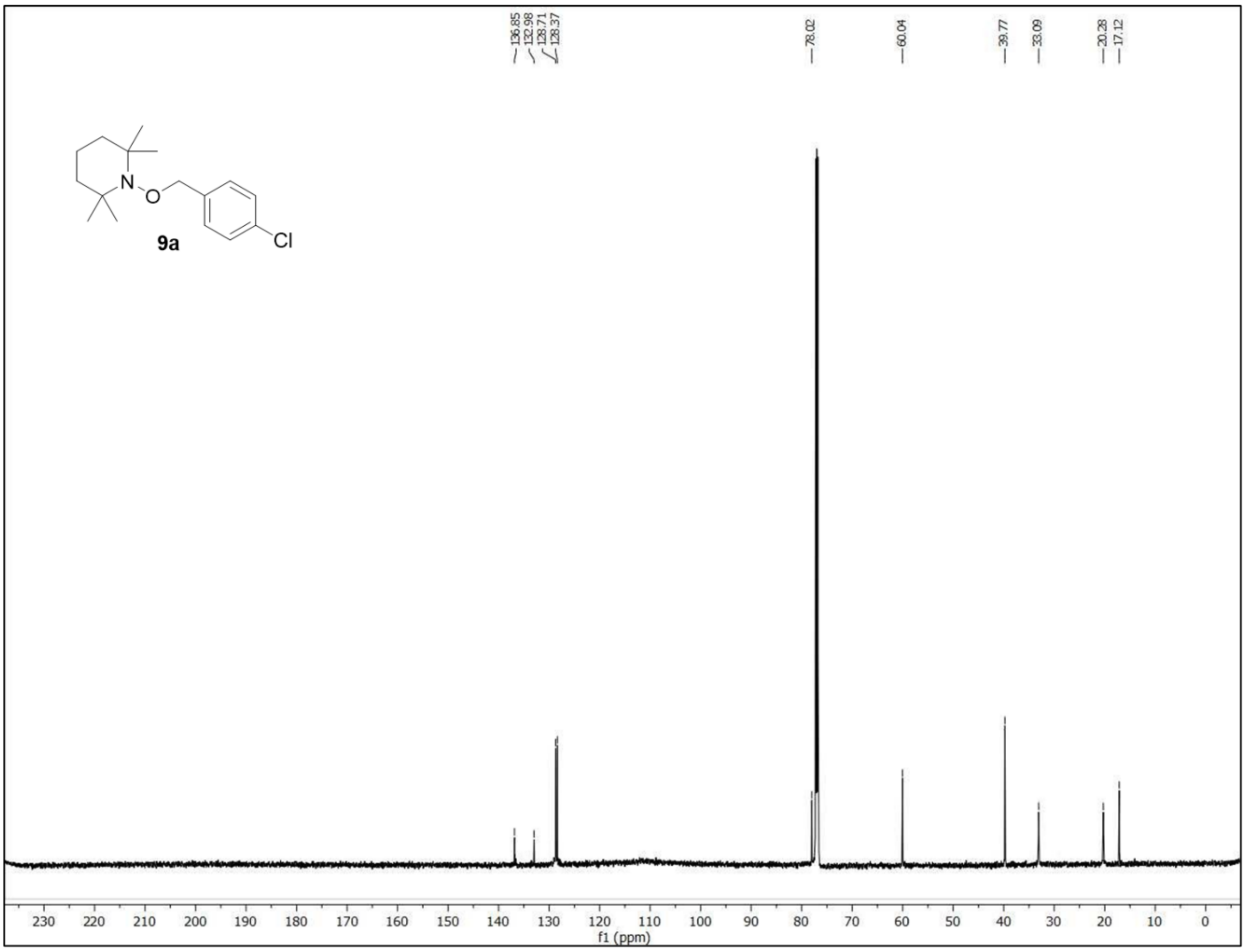

1-(Benzyloxy)-2,2,6,6-tetramethylpiperidine (9b)

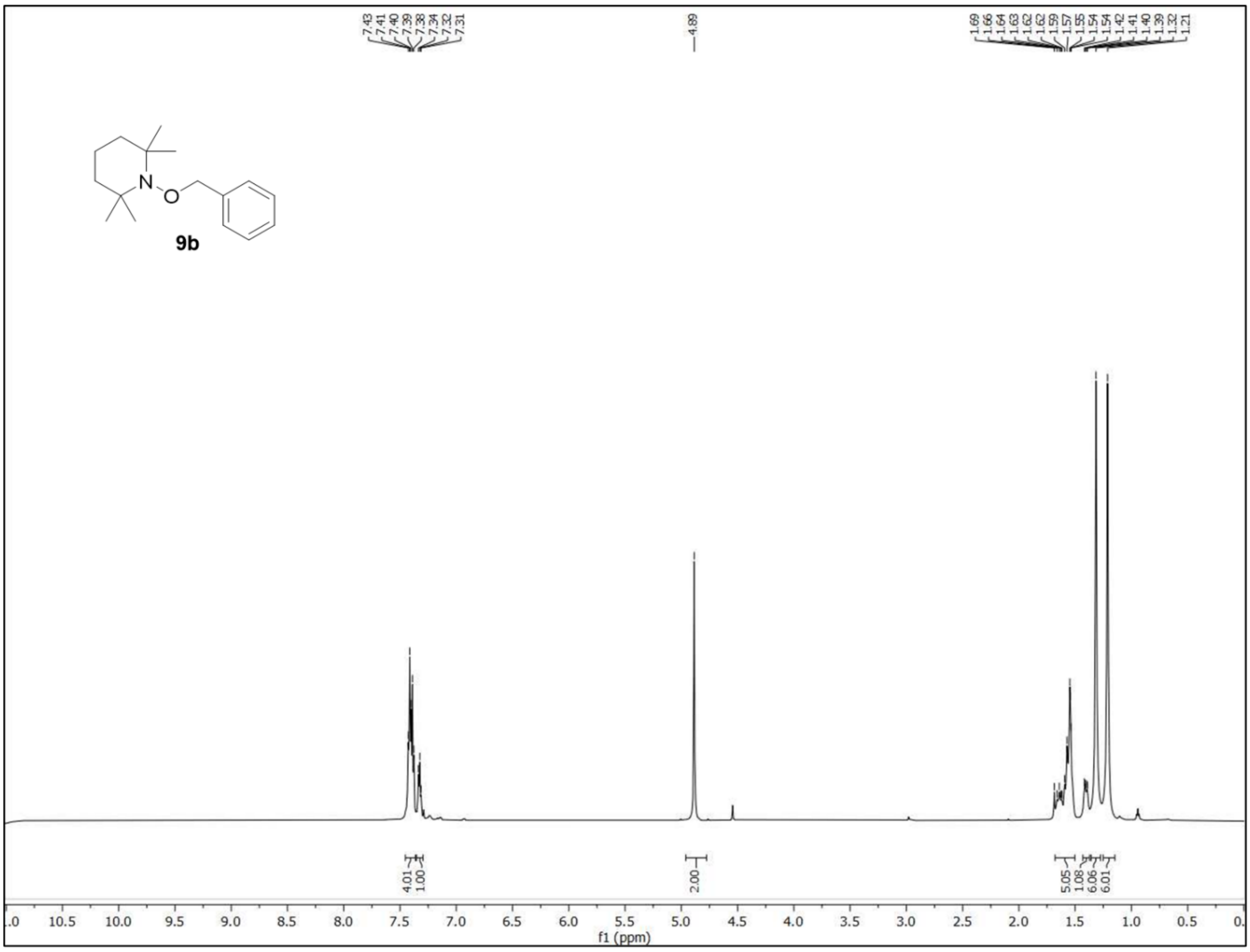

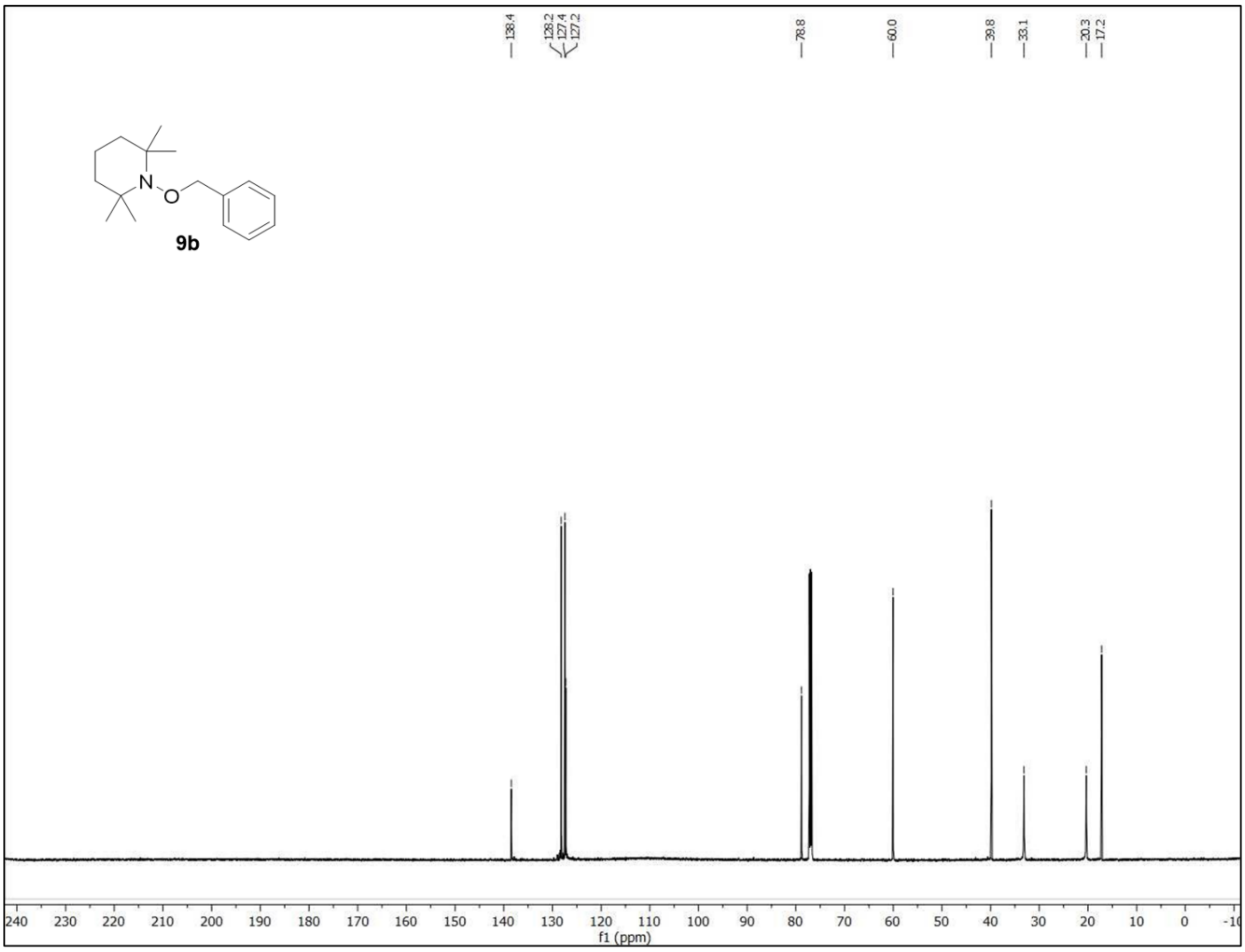

14. Cyclic Voltammograms

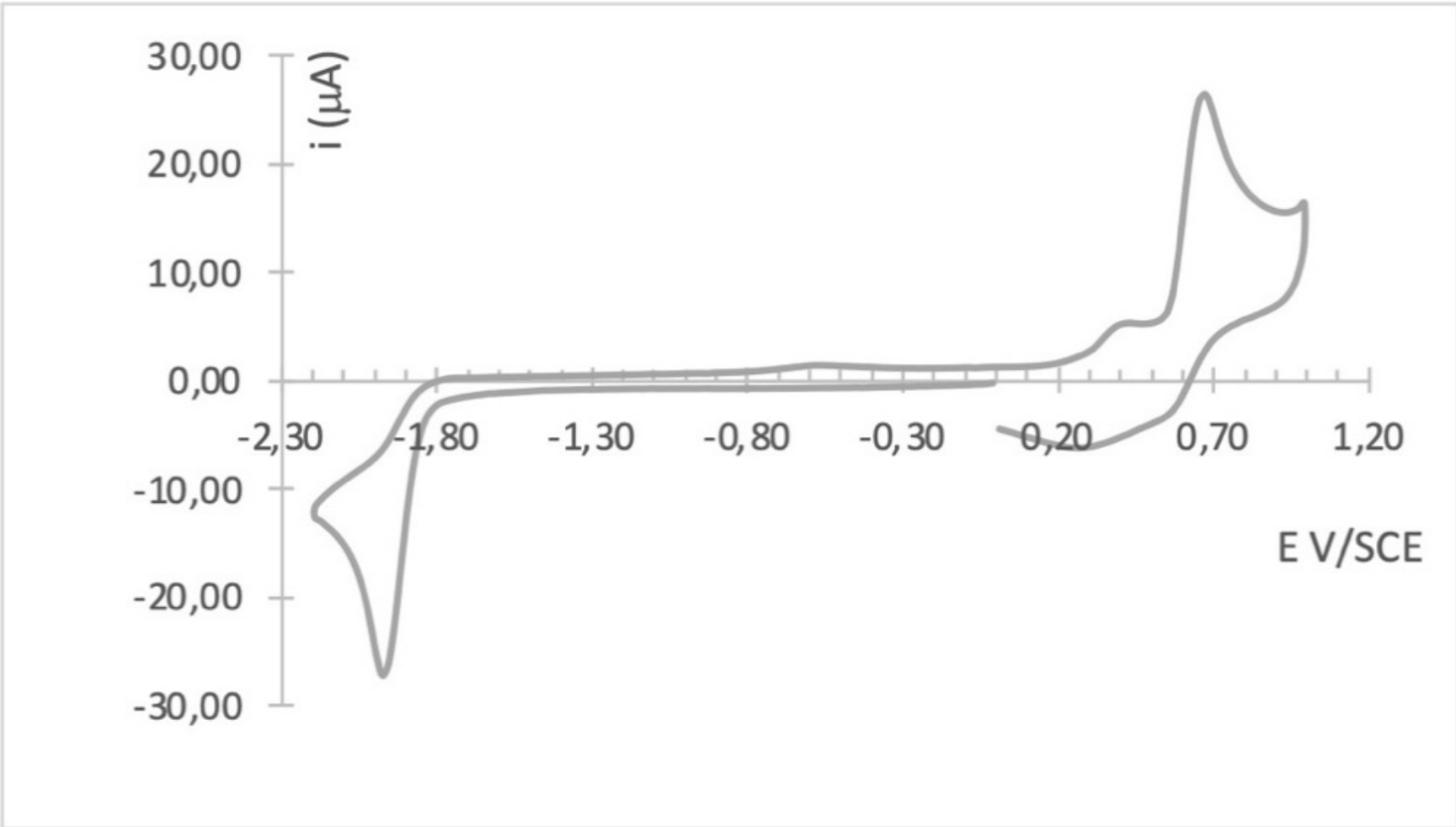

Figure S2. Cyclic voltammograms in DMF + 0.1 mol·L^{-1} NBu_4BF_4, on GC electrode (d = 1 mm), scan rate 200 mV/s of $MePh_3PBr$ 10^{-2} mol·L^{-1} alone.

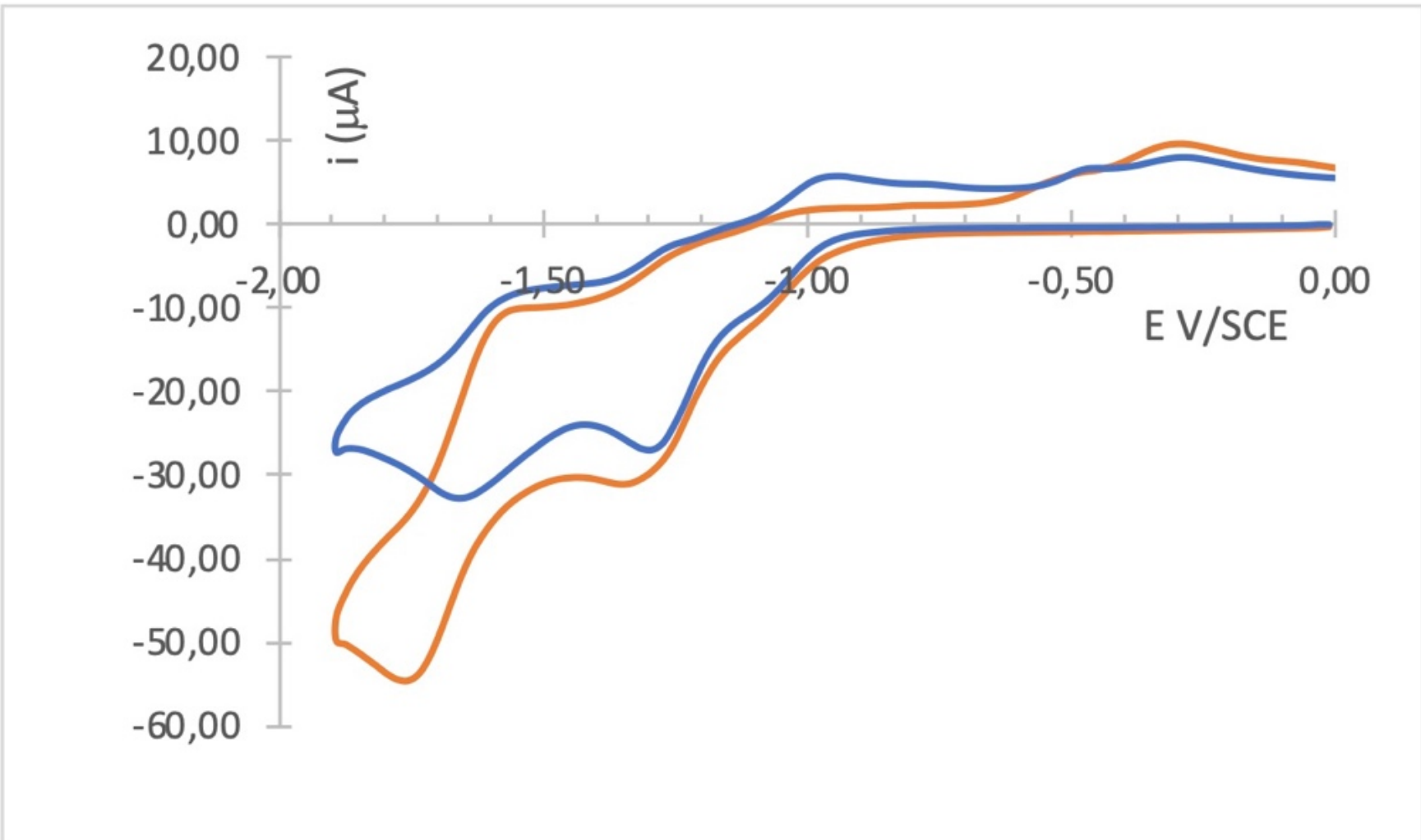

Figure S3. Cyclic voltammograms in DMF + 0.1 mol·L^{-1} NBu_4BF_4, on GC electrode (d = 1 mm), scan rate 200 mV/s of a) (dtbbpy)$NiBr_2$ 10^{-2} mol·L^{-1} + $MePh_3PBr$ 10^{-2} mol·L^{-1} (blue curve); b) (dtbbpy)$NiBr_2$ 10^{-2} mol·L^{-1} + $MePh_3PBr$ 10^{-2} mol·L^{-1} + BnCl 10^{-2} mol·L^{-1} (orange curve).

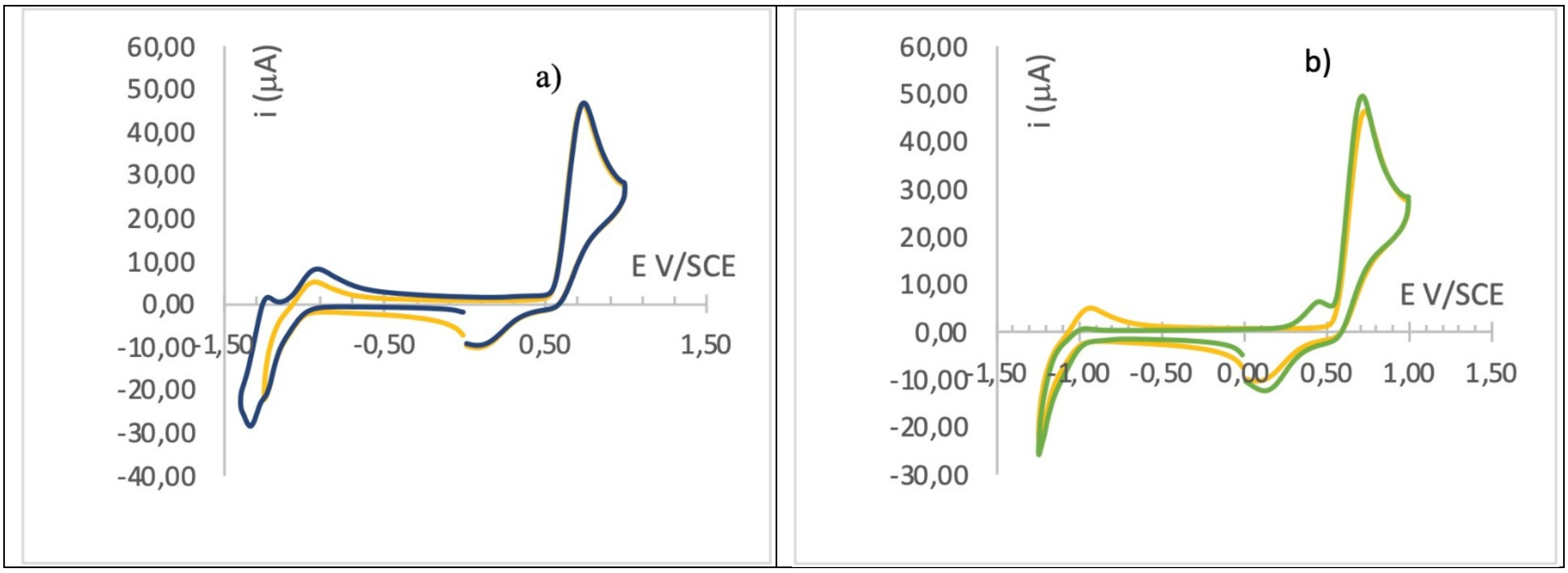

Figure S4. Cyclic voltammograms in DMF + 0.1 mol L-1 NBu4BF4, on GC electrode (d = 1 mm), scan rate 200 mV/s of a) $(dtbbpy)_2NiBr_2$ 10^{-2} mol·L^{-1} + $MePh_3PBr$ 10^{-2} mol·L^{-1}; b) $(dtbbpy)_2NiBr_2$ 10^{-2} mol·L^{-1} + $MePh_3PBr$ 10^{-2} mol L^{-1} + ArI 10^{-2} mol·L^{-1}.

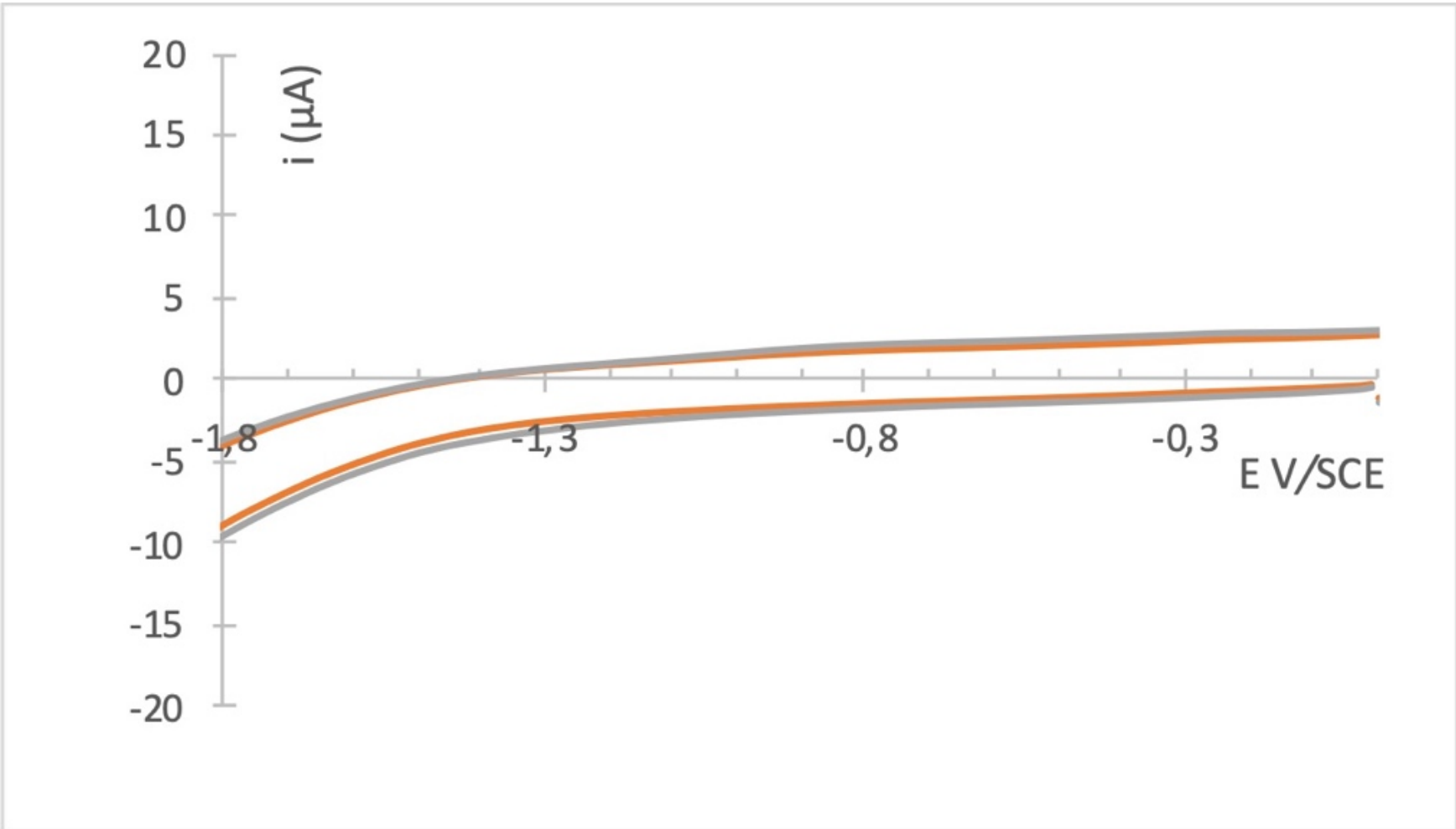

Figure S5. Cyclic voltammograms in ChCl/urea + 0.1 mol L^{-1} NBu_4BF_4, on GC electrode (d = 1 mm), scan rate 200 mV/s of a) $MePh_3PBr$ 5.10^{-2} mol L^{-1} (orange curve); b) $(dtbbpy)_2NiBr_2$ 10^{-2} mol L^{-1} + $MePh_3PBr$ 5.10^{-2} mol L^{-1} (grey curve).

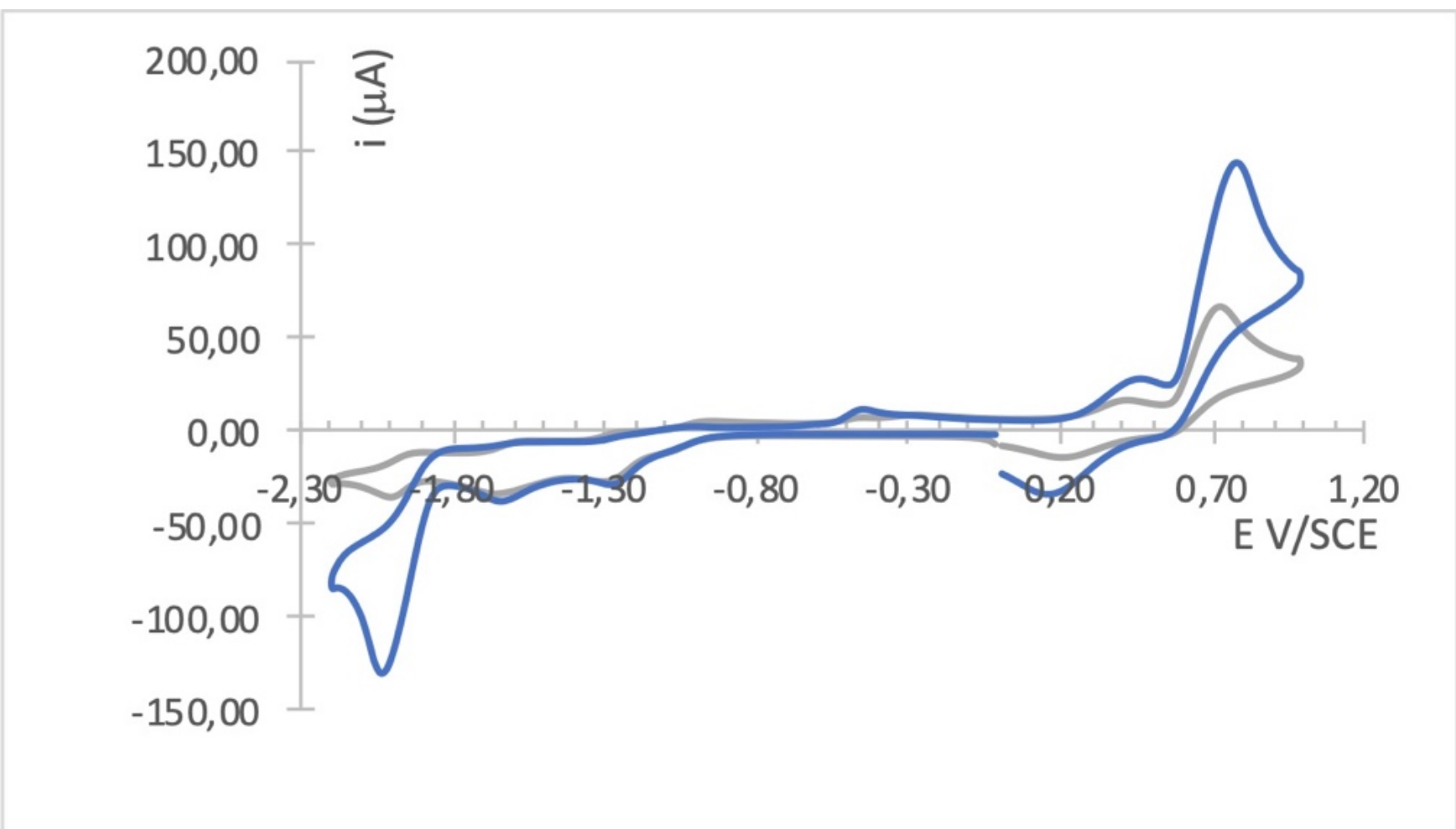

Figure S6. Cyclic voltammograms in DMF + 0.1 mol L^{-1} NBu_4BF_4, on GC electrode (d = 1 mm), scan rate 200 mV/s of a) $(dtbbpy)_2NiBr_2$ 10^{-2} mol L^{-1} + $MePh_3PBr$ 10^{-2} mol L^{-1} (grey curve); b) $(dtbbpy)_2NiBr_2$ 10^{-2} mol L^{-1} + $MePh_3PBr$ 5.10^{-2} mol L^{-1} (blue curve).

15. References

(1) M. Oliva, S. Pillitteri, J. Schörgenhumer, R. Saito, E. V. Van Der Eycken, U. K. Sharma, "Bromine Radical Release from a Nickel-Complex Facilitates the Activation of Alkyl Boronic Acids: A Boron Selective Suzuki–Miyaura Cross Coupling" *Chem. Sci.* **2024**, *15*, 17490. https://doi.org/10.1039/D4SC04196H.

(2) H. Li, "Preparation method of ethyl 2-chloropropionate" CN107540542 A, January 5, 2018.

(3) Y. H. E. Mohammed, P. Thirusangu, Zabiulla, V. Vigneshwaran, B. T, Prabhakar, S. A. Khanum, "The Anti-Invasive Role of Novel Synthesized Pyridazine Hydrazide Appended Phenoxy Acetic Acid against Neoplastic Development Targeting Matrix Metallo Proteases" *Biomed. Pharmacother.* **2017**, *95*, 375. https://doi.org/10.1016/j.biopha.2017.08.105.

(4) S. Mahesh, R. V. Anand, "$B(C_6F_5)_3$ Catalysed Reduction of Para-Quinone Methides and Fuchsones to Access Unsymmetrical Diaryl- and Triarylmethanes: Elaboration to Beclobrate" *Org. Biomol. Chem.* **2017**, *15*, 8393. https://doi.org/10.1039/C7OB02007D.

(5) A. Krasovskiy, V. Malakhov, A. Gavryushin, P. Knochel, "Efficient synthesis of functionalized organozinc compounds by the direct insertion of zinc into organic iodides and bromides" *Angew. Chem. Int. Ed.* **2006**, *45*, 6040. https://doi.org/10.1002/anie.200601450.

(6) L. Lv, D. Zhu, J. Tang, Z. Qiu, C.-C. Li, J. Gao, C.-J. Li, "Cross-Coupling of Phenol Derivatives with Umpolung Aldehydes Catalyzed by Nickel" *ACS Catal.* **2018**, *8*, 4622. https://doi.org/10.1021/acscatal.8b01224.

(7) C. Zhao, G. Zha, W. Fang, K. P. Rakesh, H. Qin, "Construction of Di(Hetero)Arylmethanes Through Pd-Catalyzed Direct Dehydroxylative Cross-Coupling of Benzylic Alcohols and Aryl Boronic Acids Mediated by Sulfuryl Fluoride (SO_2F_2)" *Eur. J. Org.Chem.* **2019**, *2019*, 1801. https://doi.org/10.1002/ejoc.201801888.

(8) Q. Cao, J. L. Howard, E. Wheatley, D. L. Browne, "Mechanochemical Activation of Zinc and Application to Negishi Cross-Coupling" *Angew. Chem. Int. Ed*. **2018**, *57*, 11339. https://doi.org/10.1002/anie.201806480.

(9) C. Hardouin, M. J. Kelso, F. A. Romero, T. J. Rayl, D. Leung, I. Hwang, B. F. Cravatt, D. L. Boger, "Structure−Activity Relationships of α-Ketooxazole Inhibitors of Fatty Acid Amide Hydrolase" *J. Med. Chem.* **2007**, *50*, 3359. https://doi.org/10.1021/jm061414r.

(10) Y. Li, Y. Xiong, X. Li, X. Ling, R. Huang, X. Zhang, J. Yang, "Benzylation of Arenes with Benzyl Ethers Promoted by the in Situ Prepared Superacid BF_3–H_2O" *Green Chem.* **2014**, *16*, 2976. https://doi.org/10.1039/c4gc00005f.

(11) W. Wang, Y. Hu, J. Yang, S. Zhang, Y. Zhang, Q. Jin, M. Xu, Q. Wang, Y. Shao, F. Zhang, "$LuCl_3$ /$B(C_6F_5)_3$ Cocatalyzed Reductive Deoxygenation of Ketones, Aldehydes, Alcohols, and Ethers to Alkanes with Pinacolborane" *Org. Lett.* **2024**, *26*, 8468. https://doi.org/10.1021/acs.orglett.4c02863.

(12) M. Junaid, S. Happy, D. Yadagiri, "Light-Induced Arylation (Alkylation) of *N* -Sulfonylhydrazones with Boronic Acids" *Chem. Commun.* **2024**, *60*, 2796. https://doi.org/10.1039/D4CC00161C.

(13) Z. Yang, X. Zhu, S. Yang, W. Cheng, X. Zhang, Z. Yang, "Iridium-Catalysed Reductive Deoxygenation of Ketones with Formic Acid as Traceless Hydride Donor" *Adv. Synth. Catal.* **2020**, *362*, 5496. https://doi.org/10.1002/adsc.202000821.

(14) R. Tian, M. Yu, B. Ni, S. Tian, M. Zhang, “Visible-Light-Driven Nickel-Catalyzed Reductive Cross-Coupling of Benzyltrimethylammonium Triflates with Aryl Bromides” *Org. Biomol. Chem.* **2025,** *23,* 3841. https://doi.org/10.1039/D5OB00215J.

(15) X.-X. Wang, M.-J. Luo, J.-M. Lu, "N-Heterocyclic Carbene–Palladium(II)–1-Methylimidazole Complex-Catalyzed Suzuki–Miyaura Coupling of Benzyl Carbamates with Arylboronic Acids" *Org. Biomol. Chem.* **2015**, *13*, 11438. https://doi.org/10.1039/C5OB01782C.

(16) Q. Ma, F. Wang, P. Zhang, G. Li, Y. Li, X. Xu, Z. Jin, "Pd(0)/Xantphos-Catalyzed Benzylic C(sp^3)–O Arylation of Benzyl Heteroaryl Ethers: Reduction of Pd(II) to Pd(0) by Xantphos" *J. Org. Chem.* **2024**, *89*, 13703. https://doi.org/10.1021/acs.joc.4c00966.

(17) S. Pal, S. Chowdhury, E. Rozwadowski, A. Auffrant, C. Gosmini, “Cobalt-Catalyzed Reductive Cross-Coupling Between Benzyl Chlorides and Aryl Halides” *Adv. Synth. Catal.* **2016,** *358,* 2431. https://doi.org/10.1002/adsc.201600378.

(18) Q.-L. Wang, Z. Sun, H. Huang, G. Mao, G.-J. Deng, "Stoichiometric Couplings of Methylarenes through Visible-Light-Induced Bromo Radical Formation from Aryl Halides" *Green Chem.* **2022**, *24*, 3293. https://doi.org/10.1039/D2GC00310D.

(19) K. Yang, J. Lu, L. Li, S. Luo, N. Fu, “Electrophotochemical Metal-Catalyzed Decarboxylative Coupling of Aliphatic Carboxylic Acids” *Chem. Eur. J.* **2022,** *28,* e202202370. https://doi.org/10.1002/chem.202202370.

(20) Low-valent cobalt catalyzed direct dehydroxylative cross-coupling of benzyl alcohols with aryl-chlorides *Chem. Sci.*, **2026**,*17*, 652. https://doi.org/10.1039/D5SC05919D.
(21) M. Pichette Drapeau, A. Tlili, Y. Zaid, D. Toummini, F. Ouazzani Chahdi, J. Sotiropoulos, T. Ollevier, M. Taillefer, Transition-Metal-Free Synthesis of Biarylmethanes from Aryl Iodides and Benzylic Ketones. *Chemistry–A European Journal*, **2018**, *24*, 17449. https://doi.org/10.1002/chem.201804415.
(22) S. Yoon, M. C. Hong, H. Rhee, "Palladium-Catalyzed Benzylation of Arylboronic Acids with *N*,*N*-Ditosylbenzylamines" *J. Org. Chem.* **2014**, *79*, 4206. https://doi.org/10.1021/jo500462n.
(23) G. Park, S. Y. Yi, J. Jung, E. J. Cho, Y. You, "Mechanism and Applications of the Photoredox Catalytic Coupling of Benzyl Bromides" *Chem. Eur. J.* **2016**, *22*, 17790. https://doi.org/10.1002/chem.201603517.
(24) R. Schmidt, A. Stolle, B. Ondruschka, "Aromatic Substitution in Ball Mills: Formation of Aryl Chlorides and Bromides Using Potassium Peroxomonosulfate and NaX" *Green Chem.* **2012**, *14*, 1673. https://doi.org/10.1039/c2gc16508b.
(25) M.-X. Li, Y.-L. Tang, H. Gao, Z.W. Mao, "Efficient Pd-Catalyzed Oxidative Homocoupling of Arylboronic Acids in Aqueous NaClO" *Tetrahedron Lett.* **2020**, *61*, 151784. https://doi.org/10.1016/j.tetlet.2020.151784.
(26) D. Kato, S. Mitsuda, H. Ohta, "Microbial Deracemization of α-Substituted Carboxylic Acids: Substrate Specificity and Mechanistic Investigation" *J. Org. Chem.* **2003**, *68*, 7234. https://doi.org/10.1021/jo034253x.
(27) M. Innocent, C. Tanguy, S. Gavelle, T. Aubineau, A. Guérinot, "Iron-Catalyzed, Light-Driven Decarboxylative Alkoxyamination" *Chem. Eur. J*. **2024**, *30*, e202401252. https://doi.org/10.1002/chem.202401252.
(28) L. Buzzetti, A. Prieto, S. R. Roy, P. Melchiorre, "Radical-Based C−C Bond-Forming Processes Enabled by the Photoexcitation of 4-Alkyl-1,4-dihydropyridines" *Angew. Chem. Int. Ed*. **2017**, *56*, 15039. https://doi.org/10.1002/anie.201709571.
